# The global burden and associated factors of ovarian cancer in 1990–2019: findings from the Global Burden of Disease Study 2019

**DOI:** 10.1186/s12889-022-13861-y

**Published:** 2022-07-30

**Authors:** Shiwen Zhang, Chen Cheng, Zejian Lin, Linzi Xiao, Xin Su, Lu Zheng, Yingjun Mu, Minqi Liao, Ruiqing Ouyang, Wanlin Li, Junrong Ma, Jun Cai, Lu Liu, Donghong Wang, Fangfang Zeng, Jun Liu

**Affiliations:** 1 Department of Public Health and Preventive , of Medicine, Jinan University, Guangzhou, China; 2 Department of Preventive Medicine Laboratory, School of Public Health, Zunyi Medical University, Zunyi, China; 3 Department of Gynaecology and Obstetrics, Affiliated Hospital of Zunyi Medical University, Guizhou, China

**Keywords:** Ovarian cancer, Risk factors, Global burden of disease study 2019

## Abstract

**Background:**

Ovarian cancer (OC) is a major cause of cancer-related deaths among women. The aim of this study was to estimate and report data on the current burden of ovarian cancer worldwide over the past 30 years.

**Method:**

Based on the data provided by GBD 2019, we collected and interpreted the disease data of ovarian cancer by incidence, mortality, disability-adjusted life-years (DALYs), and used corresponding age-standardized rates as indicators. Also, we categorized the data by attributed risk factors and captured deaths due to high fasting plasma glucose, occupational exposure to asbestos and high body-mass index, respectively. All outcomes in the study were reported using mean values and corresponding 95% uncertainty intervals (95% UI).

**Results:**

Globally, there were 294422 (260649 to 329727) incident cases in 2019, and the number of deaths and DALYs were 198412 (175357 to 217665) and 5.36 million (4.69 to 5.95). The overall burden was on the rise, with a percentage change of 107.8% (76.1 to 135.7%) for new cases, 103.8% (75.7 to 126.4%) for deaths and 96.1% (65.0 to 120.5%) for DALYs. Whereas the age-standardized rates kept stable during 1990–2019. The burden of ovarian cancer increased with age. and showed a totally different trends among SDI regions. Although high SDI region had the declining rates, the burden of ovarian cancer remained stable in high-middle and low SDI regions, and the middle and low-middle SDI areas showed increasing trends. High fasting plasma glucose was estimated to be the most important attributable risk factor for ovarian cancer deaths globally, with a percentage change of deaths of 7.9% (1.6 to 18.3%), followed by occupational exposure to asbestos and high body mass index.

**Conclusions:**

Although the age-standardized rates of ovarian cancer didn’t significantly change at the global level, the burden still increased, especially in areas on the lower end of the SDI range. Also, the disease burden due to different attributable risk factors showed heterogeneous, and it became more severe with age.

**Supplementary Information:**

The online version contains supplementary material available at 10.1186/s12889-022-13861-y.

## Introduction

Ovarian cancer (OC) is a type of aggressive gynecologic malignancy disease. In 2020, the latest global cancer burden data showed that it ranked eighth among female cancer deaths, the fifth most common cause of cancer death in women in Australia, North America, and Western Europe, accounting for 5% of female oncology deaths worldwide and more than any other gynecologic cancer [1]. Ovarian cancer has an insidious onset, and the prognosis is often poor because it is usually difficult to treat with conventional therapies due to recurrence and drug resistance [2]. For example, in Europe, the average five-year survival rate was only 29% [3].

Today, in a global perspective, the number of people with the disease varies greatly from country to country. Many variabilities add to the fact that ovarian cancer is a complex disease that has emerged as major global public health concern. In order to better understand the enormous public health impact of this disease, it is necessary to identify relevant global trends through statistics and analysis.

Ovarian cancer can be attributed to multiple risk factors (e.g., a history of smoking, nulliparity, and so on) [4]. But few comprehensive reviews have been conducted on ovarian cancer risk factors in the context of global data, and some studies that showed no association were included at the same time [5]. Several studies on ovarian cancer about GBD 2017 have been published before [6–8]. Besides the data version need to be updated, whether a step-change in the disease burden is meaningful remains to be determined [8]. Also, one study did not address changes in disability-adjusted life years [7], and it can be seen that there was no overall grasp of age-standardized rates changes over the past 30 years [6, 7]. The same as this study, some researches show that the trend in some absolute numbers may be opposite to the trend in related ASRs during past 30 years, such as cardiovascular diseases [9], so it is necessary to discuss absolute numbers in conjunction with ASRs.

As part of GBD 2019, this study provided the information of distribution and trends in the burden of ovarian cancer globally, regionally, and in 204 countries, between 1990 and 2019. Different from previous GBD studies on ovarian cancer [6–8], in this study we focus not only on the details of geographic and long-term patterns of OC incidence and mortality over the past 30 years. Our objectives were to estimate the number of ovarian cancer new cases, deaths, and corresponding disability-adjusted life years (DALYs) between 1990 and 2019 based on country, age, and sociodemographic status, to explore age trends in ovarian cancer and to analyze the major risk factors that contributed to the deaths of OC.

## Methods and materials

### Overview

Data for this study was obtained from the Global Burden of Diseases, Injuries, and Risk Factors Study 2019 (GBD 2019). The GBD study provides a tool to quantify the health losses from hundreds of diseases, injuries, and risk factors to improve health systems and eliminate disparities [10]. GBD is now widely used to understand the global burden of diseases. We searched the GBD database for global female ovarian cancer data from 1990 to 2019 through the GBD Results Tool of IHME (GHDx, a large database of health-related data maintained by the Institute for Health Metrics and Evaluation, http://ghdx.healthdata.org).

The codes corresponding to cancer in the GBD etiology hierarchy were derived from the ICD-9 and ICD-10 code books of the International Classification of Diseases, which is consistent with Guidelines for Accurate and Transparent Health Estimates Reporting (GATHER) [11], and could be used to identify OC data. Incidence, mortality, and disability-adjusted life years and their corresponding age-standardized rates were selected as disease burden evaluation parameters, and the data obtained were subdivided according to geographic location, age groups, and attributable risk factors. The specific GBD study design and methods have been described in detail in previous literature [10, 12], and here we briefly review the methods used to assess the burden of ovarian cancer and its attributable risk factors.

### Geographic units and age groups

In addition to natural geographic location, these countries and regions were divided into five regions based on different Socio-demographic indexes (SDI): high, high-middle, middle, low-middle, and low SDI regions. The SDI is a composite indicator of national and regional development status, expressed on a scale of 0 to 1, and is a measure of per capita income, average educational attainment (15 years and older), and total fertility (25 years and older) for all regions in the GBD study, and is strongly correlated with health outcomes [13, 14].

GBD provides multiple age groupings, and in this study, we focused on data on the burden of ovarian cancer in three groups: 15–49, 50–69, and ≥ 70 years.

### Statistical analyses

The first step in estimating cancer burden calculations is modeling for specific mortality rates [15]. Although mortality data are available from a wide range of sources (vital registration, verbal autopsies, mortality surveillance, census, etc.), there are still instances where mortality data are not available for certain locations and time points [16, 17]. Accordingly, in GBD study, separately modeled mortality-to-incidence ratios (MIRs) was generated to maximize the availability of data obtained from cancer registries. Subsequently, the mortality estimates were used as inputs to Cause of Death Ensemble model (CODEm), which predicts single-cause mortality based on available data and causal covariates, allowing estimation of the number of deaths due to ovarian cancer by location, age, sex, and year. The specific modeling strategy has been described in detail in other literature [16].

Methods for estimating disease incidence in GBD 2019 study have been described in detail in previous study [10]. Ovarian cancer incidence data in GBD study was determined by using a literature review and studies jointly generated from a wide range of population representative data sources covering microdata, such as scientific reports from a large number of cohorts and registries, as well as macro-administrative data from health systems administrative. According to related studies, the estimation of all available data on incidence was calculated by Bayesian meta-regression software DisMod-MR 2.1 [18], and the values were estimated by dividing the mortality estimates of OC by the corresponding MIRs [15].

Disability is widely used in burden of disease analysis nowadays and refers to deviations from good or ideal health in any important area of health. Disability-adjusted life years (DALY) is a composite measure of time lost due to premature death and time spent living in less-than-optimal health, can fully analyze the population impact in of disease burden [19]. It is measured by summing the number of years of life lost (YLL) due to premature death, and the number of years of life disabled (YLD) due to nonfatal health loss [20]. In this case, YLL is estimated using each death multiplied by the standard life expectancy at each age, while YLD is estimated from the prevalence of sequelae and disability weights derived from population-based surveys [21]. For most sequelae, the same as the estimation of incidence, GBD 2019 study used the Bayesian meta-regression method DisMod MR 2.1 [18], to address some of the limitations in descriptive epidemiological data, such as missing, or inconsistent data.

### Estimation of risk factors

Since 2002, the GBD has followed a comparative risk assessment (CRA) approach to quantify the attributable burden [22, 23], in which risk factors are classified into four tiers, ranging from the broadest risk category (e.g. behavioral, environmental and occupational, and metabolic) to the most detailed classification (e.g. discontinued and non-exclusive breastfeeding). 87 risks or risk clusters were provided in GBD 2019, and to ensure the persuasiveness of risk-outcomes on the relevant evidence, researchers excluded risk-outcome pairs with correlation results *p*-value > 0.1 from the existing studies. And ultimately 12 risk-outcome pairs in GBD 2017 were excluded from GBD 2019 after final reviews and meta-analysis [24]. This study mainly focused on the change in mortality, and related age-standardized rate by the most prominent risk factors for ovarian cancer, and included all risk factors, and top three most detailed risk factors.

## Results

### Burden of incidence

**Fig. 1:**
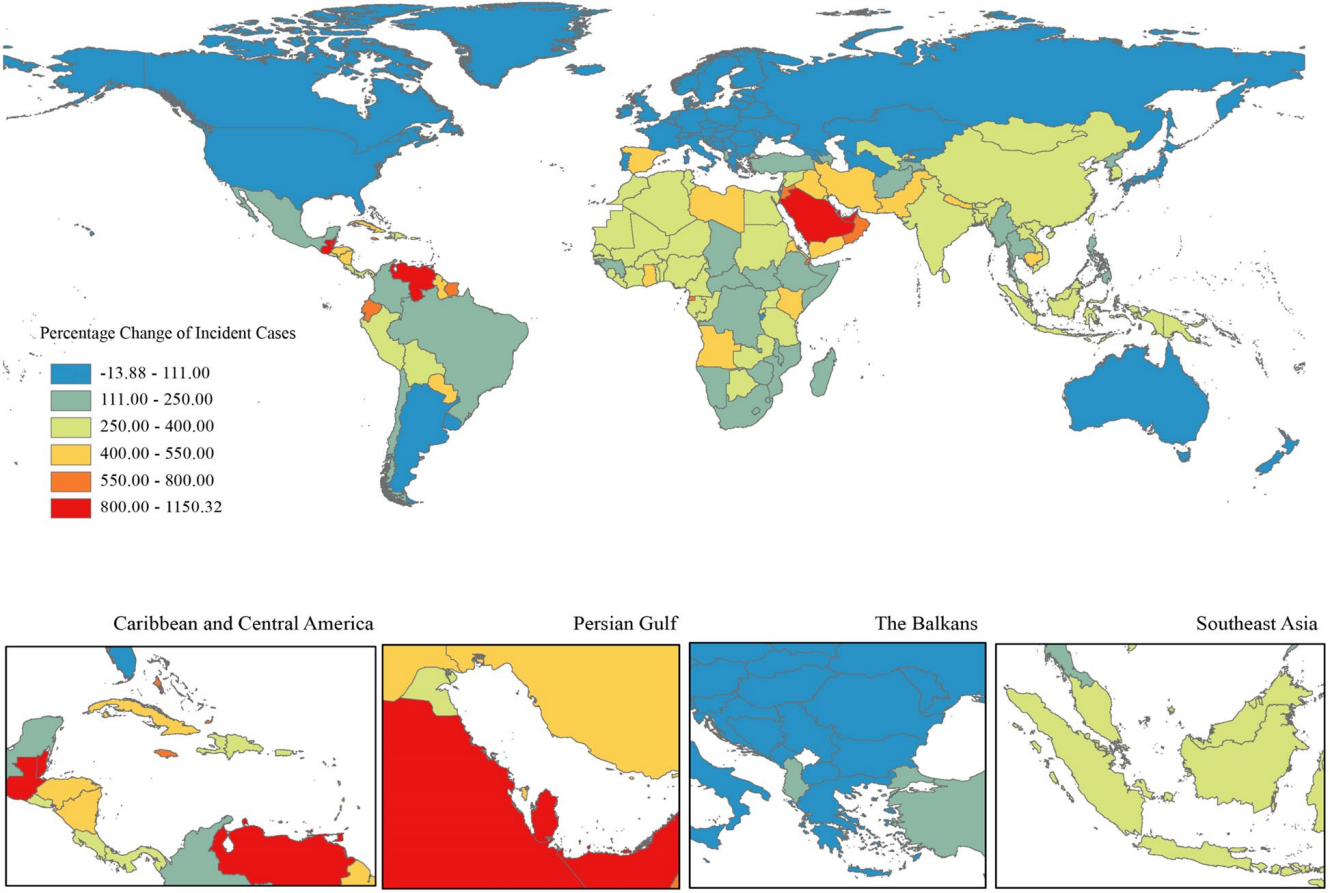
Map of percentage change of incident cases due to ovarian cancer, 1990–2019

**Fig. 2:**
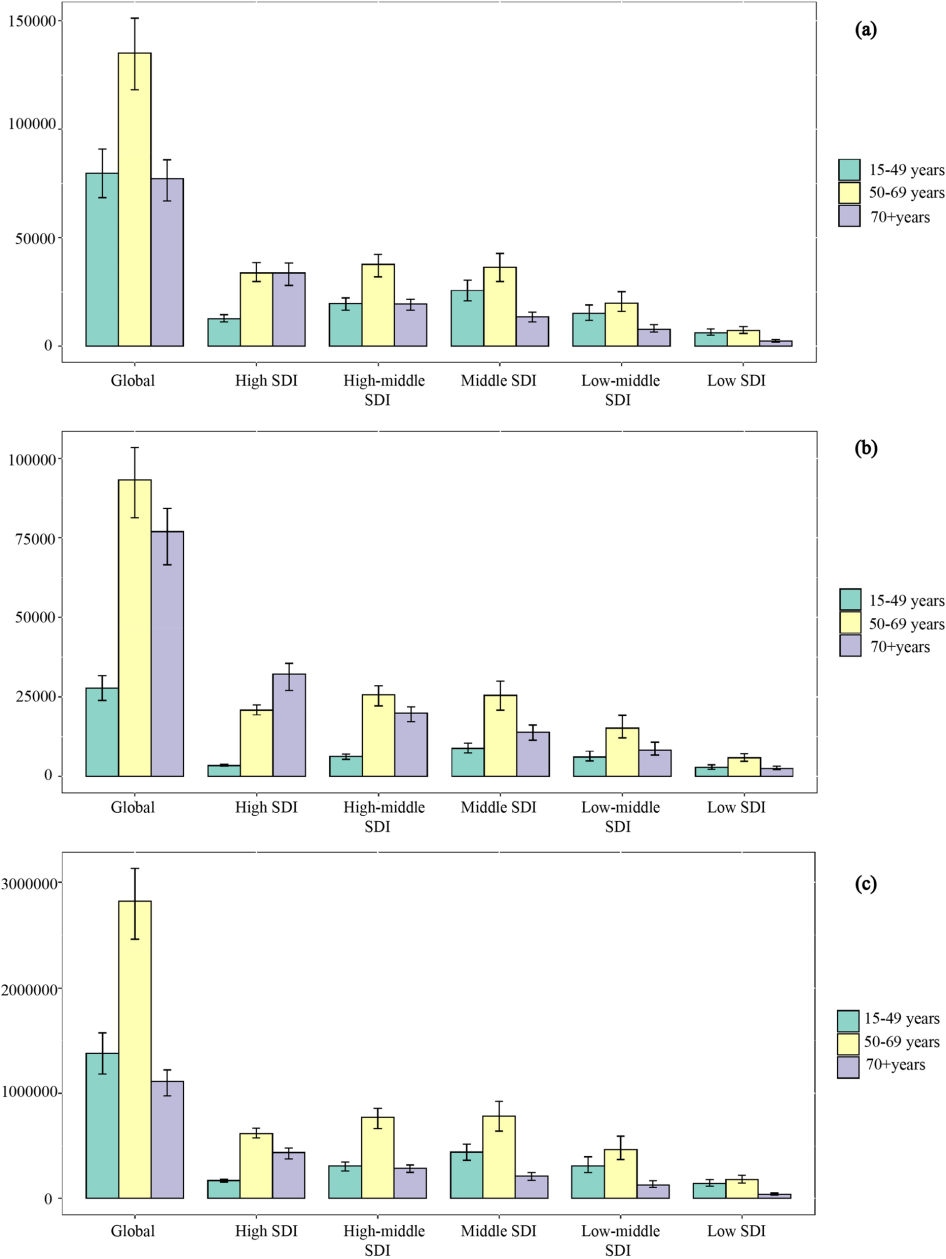
Incident cases (**a**), Deaths (**b**) and DALYs (**c**) of three age groups in different SDI regions in 2019

In 2019, the number of ovarian cancer incident cases was 294422 (260649 to 329727). The overall burden of ovarian cancer was on the rise, especially in the number of cases, with a percentage change of 107.8% (76.1 to 135.7%) compared to 1990 (Table 1, Fig. 1). But the percentage changes of global age-standardized incidence rate kept stable during the same time period. All three groups (15–49, 50–69, and 70 + age groups) showed an increasing trend in the number of cases between 1990 and 2019, with the highest cases number in 2019 in the 50–69 age group. The age group of largest percentage change in the number of incident cases was 70 + age group, were 119.9% (92.9 to 143.9%) (Table 1, Fig. 2a).

**Table 1:** Incident cases, Deaths, DALYs in 1990 and 2019, percentage change for ovarian cancer during 1990–2019, by age groups, global, SDI regions, world regions and countries

**Characteristics**	**Incident cases (95% UI)**			Deaths (95% UI)			DALYs (95% UI)		
	**1990**	**2019**	Percentage change in cases, 1990–2019	**1990**	**2019**	**Percentage change in cases, 1990–2019**	**1990**	**2019**	**Percentage change in cases, 1990–2019**
Global	141706 (130,541 to 160779)	294422 (260649 to 329727)	107.8% (76.1 to 135.7%)	97363 (89703 to 109761)	198412 (175357 to 217665)	103.8% (75.7 to 126.4%)	2,732666 (2,493732 to 3,165170)	5,359737 (4,692949 to 5954993)	96.1% (65.0 to 120.5%)
**Age (years)**									
15 ~ 49	39872 (34418 to 48766)	79672 (68573 to 90845)	99.8% (55.7 to 138.0%)	15139 (12927 to 18976)	27759 (23923 to 31722)	83.4% (40.4 to 116.9%)	760870 (645441 to 960213)	1379355 (1183284 to 1574162)	81.3% (37.8 to 115.7%)
50 ~ 69	65290 (60965 to 73424)	135107 (118168 to 151221)	106.9% (75.4 to 134.6%)	47082 (43811 to 53461)	93173 (81,327 to 103381)	97.9% (67.1 to 122.8%)	1420153 (1321775 to 1614600)	2823174 (2459433 to 3132271)	98.8% (67.9 to 124.4%)
> 70	35083 (32165 to 37477)	77131 (66946 to 85958)	119.9% (92.9 to 143.9%)	34774 (31793 to 37331)	76943 (66525 to 84280)	121.3% (95.7 to 140.3%)	522102 (482096 to 560730)	1113871 (975511 to 1221853)	113.3% (87.7 to 132.6%)
**SDI regions**									
High SDI	62463 (56715 to 64466)	80454 (70504 to 91461)	28.8% (13.9 to 49.8%)	43458 (39182 to 45018)	56639 (50391 to 61318)	30.3% (19.1 to 47.3%)	1061103 (956786 to 1094307)	1229123 (1125703 to 1323417)	15.8% (6.5 to 35.4%)
High-middle SDI	43567 (40298 to 46702)	77286 (65885 to 86459)	77.4% (54.3 to 100.0%)	29784 (27764 to 31762)	51967 (44998 to 57246)	74.5% (52.6 to 92.9%)	864811 (793572 to 925109)	1378231 (1191048 to 1526401)	59.4% (38.7 to 76.9%)
Middle SDI	21289 (18298 to 27014)	76545 (63249 to 88974)	259.6% (152.7 to 331.7%)	13706 (12089 to 17148)	48485 (39894 to 56526)	253.8% (155.6 to 324.8%)	462013 (401880 to 592519)	1453634 (1199319 to 1696724)	214.6% (124.0 to 277.4%)
Low-middle SDI	10234 (8031 to 16701)	43595 (35303 to 54683)	326.0% (158.8 to 470.5%)	7311 (5765 to 11582)	29874 (24421 to 37624)	308.6% (151.3 to 445.8%)	241438 (188017 to 394770)	922653 (740013 to 1169375)	282.1% (129.6 to 412.9%)
Low SDI	4095 (2808 to 8460)	16389 (13486 to 20299)	300.2% (126.2 to 485.4%)	3065 (2119 to 6167)	11346 (9551 to 13928)	270.2% (115.2 to 433.2%)	102198 (69309 to 215443)	373324 (311090 to 462226)	265.3% (104.7 to 436.5%)
High-income North America	23115 (21222 to 23959)	29785 (24355 to 36248)	28.9% (4.9 to 60.6%)	15850 (14330 to 16508)	21631 (19536 to 23591)	36.5% (25.4 to 61.3%)	379316 (348905 to 393040)	472990 (439017 to 512580)	24.7% (15.1 to 49.6%)
Canada	2067 (1920 to 2195)	2994 (2239 to 4011)	44.9% (7.4 to 100.0%)	1419 (1318 to 1501)	2125 (1759 to 2521)	49.7% (25.4 to 81.6%)	35555 (33131 to 37449)	46390 (39180 to 54672)	30.5% (9.8 to 57.4%)
Greenland	3 (3 to 4)	4 (3 to 6)	41.6% (-1.9 to 95.3%)	2 (2 to 3)	3 (2 to 4)	40.0% (-4.6 to 93.2%)	68 (55 to 85)	89 (64 to 115)	29.8% (-11.0 to 80.7%)
United States of America	21044 (19305 to 21824)	26786 (21456 to 32815)	27.3% (2.0 to 60.9%)	14428 (12981 to 15032)	19503 (17644 to 21126)	35.2% (25.0 to 62.3%)	343684 (314204 to 356277)	426504 (397117 to 461998)	24.1% (14.8 to 51.4%)
**Australasia**	1315 (1145 to 1386)	1931 (1524 to 2472)	46.9% (15.1 to 98.1%)	906 (794 to 955)	1384 (1182 to 1592)	52.8% (30.0 to 102.0%)	22733 (19655 to 23917)	29637 (25738 to 34301)	30.4% (12.0 to 78.5%)
Australia	1066 (930 to 1131)	1598 (1216 to 2097)	49.9% (12.4 to 108.4%)	738 (646 to 781)	1146 (970 to 1334)	55.2% (31.5 to 106.6%)	18512 (16071 to 19498)	24264 (20875 to 28371)	31.1% (11.8 to 82.0%)
New Zealand	248 (220 to 269)	333 (257 to 439)	34.1% (0.7 to 79.7%)	168 (148 to 181)	238 (204 to 270)	42.0% (19.8 to 76.4%)	4221 (3694 to 4540)	5373 (4682 to 6090)	27.3% (7.5 to 61.9%)
**High-income Asia Pacific**	6620 (6311 to 6965)	11882 (9655 to 14073)	79.5% (44.6 to 112.7%)	4133 (3913 to 4418)	7341 (6205 to 8064)	77.6% (52.2 to 94.6%)	122455 (117885 to 129485)	167714 (147322 to 181069)	37.0% (19.8 to 47.5%)
Brunei Darussalam	7 (5 to 11)	31 (24 to 38)	327.2% (172.4 to 509.4%)	4 (3 to 7)	17 (13 to 20)	291.8% (145.9 to 447.7%)	154 (112 to 239)	562 (440 to 689)	265.2% (126.9 to 421.3%)
Japan	5950 (5627 to 6177)	9198 (7192 to 11347)	54.6% (21.1 to 92.8%)	3723 (3525 to 3850)	5791 (4858 to 6400)	55.6% (35.7 to 69.8%)	108566 (103437 to 112022)	127087 (112556 to 137754)	17.1% (5.3 to 27.7%)
Singapore	106 (96 to 116)	289 (225 to 368)	172.9% (107.4 to 255.1%)	65 (60 to 71)	163 (140 to 185)	149.1% (112.9 to 187.4%)	2084 (1908 to 2270)	4478 (3858 to 5086)	114.9% (83.3 to 150.3%)
Republic of Korea	557 (484 to 923)	2364 (1376 to 2981)	324.7% (33.9 to 477.8%)	341 (291 to 600)	1370 (823 to 1608)	301.7% (17.2 to 429.2%)	11651 (10139 to 19198)	35587 (20988 to 41442)	205.4% (-7.4 to 297.5%)
**Western Europe**	37386 (33294 to 38634)	42235 (36189 to 49048)	13.0% (-2.6 to 33.9%)	26356 (23607 to 27318)	30612 (27024 to 33277)	16.1% (4.9 to 30.2%)	624896 (553975 to 645271)	626364 (569657 to 678617)	0.2% (-8.8 to 18.7%)
Andorra	2 (1 to 3)	5 (4 to 7)	140.5% (43.2 to 265.0%)	1 (1 to 2)	3 (2 to 4)	151.8% (49.6 to 288.3%)	34 (24 to 53)	77 (55 to 104)	123.9% (33.3 to 246.2%)
Austria	929 (718 to 988)	801 (637 to 1093)	-13.7% (-32.4 to 52.8%)	664 (513 to 706)	576 (497 to 746)	-13.2% (-25.0 to 47.0%)	15294 (11756 to 16241)	12027 (10486 to 16923)	-21.4% (-31.8 to 44.7%)
Belgium	1150 (959 to 1237)	1022 (797 to 1347)	-11.1% (-32.8 to 26.1%)	867 (712 to 935)	796 (678 to 909)	-8.3% (-23.9 to 19.7%)	19555 (16565 to 20965)	15700 (13454 to 18539)	-19.7% (-33.5 to 13.2%)
Cyprus	38 (29 to 55)	94 (68 to 119)	150.6% (30.9 to 275.3%)	28 (21 to 40)	65 (46 to 80)	134.4% (18.7 to 250.9%)	707 (546 to 1036)	1508 (1105 to 1853)	113.5% (13.7 to 212.7%)
Denmark	490 (459 to 521)	626 (485 to 812)	27.7% (-1.3 to 68.2%)	391 (367 to 414)	489 (427 to 564)	25.0% (8.6 to 46.7%)	9428 (8850 to 9988)	10204 (9019 to 11775)	8.2% (-5.4 to 26.5%)
Finland	484 (442 to 515)	630 (494 to 795)	30.0% (1.2 to 69.2%)	331 (302 to 352)	450 (388 to 511)	35.8% (18.4 to 56.3%)	7823 (7169 to 8267)	8924 (7812 to 10077)	14.1% (-0.6 to 36.7%)
France	4947 (4326 to 5237)	5922 (4540 to 7637)	19.7% (-7.8 to 57.8%)	3687 (3255 to 3917)	4608 (3851 to 5319)	25.0% (7.3 to 47.2%)	84437 (73390 to 89061)	90047 (77790 to 102831)	6.6% (-8.1 to 34.5%)
Germany	9480 (7778 to 9982)	9125 (7002 to 11727)	-3.7% (-26.9 to 26.9%)	6657 (5399 to 7044)	6636 (5769 to 7449)	-0.3% (-12.6 to 15.6%)	153634 (125334 to 161598)	132650 (117611 to 147636)	-13.7% (-24.8 to 5.7%)
Greece	720 (675 to 779)	1023 (797 to 1305)	42.2% (8.8 to 81.1%)	492 (462 to 532)	756 (666 to 835)	53.5% (34.7 to 72.6%)	12432 (11690 to 13388)	15641 (14155 to 17405)	25.8% (12.4 to 41.7%)
Iceland	20 (18 to 23)	23 (19 to 30)	15.8% (-6.1 to 60.7%)	14 (12 to 15)	15 (13 to 19)	13.7% (-5.7 to 54.8%)	332 (296 to 368)	346 (294 to 449)	4.2% (-13.0 to 45.0%)
Ireland	318 (289 to 346)	459 (336 to 616)	44.6% (2.6 to 103.7%)	222 (204 to 241)	311 (238 to 374)	39.7% (1.4 to 76.0%)	5598 (5148 to 6057)	7112 (5534 to 8621)	27.0% (-6.0 to 61.5%)
Israel	289 (247 to 312)	537 (407 to 692)	86.1% (39.5 to 148.0%)	209 (177 to 225)	376 (328 to 423)	79.5% (57.0 to 116.3%)	5308 (4457 to 5694)	8646 (7645 to 9714)	62.9% (42.0 to 102.3%)
Italy	4679 (4479 to 4854)	5816 (4498 to 7405)	24.3% (-3.6 to 58.4%)	3220 (3074 to 3345)	4279 (3727 to 4712)	32.9% (19.3 to 44.4%)	79549 (76253 to 82084)	88236 (79937 to 95792)	10.9% (1.1 to 21.9%)
Luxembourg	46 (42 to 51)	54 (43 to 72)	17.9% (-8.5 to 56.7%)	34 (31 to 37)	40 (32 to 50)	18.8% (-5.0 to 52.3%)	794 (725 to 870)	866 (702 to 1114)	9.2% (-11.8 to 41.4%)
Malta	30 (26 to 33)	46 (37 to 57)	55.9% (23.4 to 99.0%)	21 (18 to 23)	33 (27 to 40)	58.6% (28.0 to 99.5%)	530 (463 to 585)	715 (590 to 867)	35.0% (10.3% to 70.1%)
Monaco	7 (5 to 9)	9 (6 to 11)	35.8% (-9.8 to 95.6%)	5 (4 to 7)	7 (5 to 8)	33.8% (-9.4 to 90.8%)	106 (77 to 144)	137 (98 to 173)	29.4% (-14.6 to 88.2%)
Netherlands	1507 (1328 to 1599)	1825 (1407 to 2305)	21.0% (-4.9 to 52.4%)	1063 (933 to 1123)	1350 (1164 to 1503)	27.1% (12.3 to 41.9%)	25511 (22440 to 26867)	27530 (24372 to 30407)	7.9% (-3.6 to 22.0%)
Norway	455 (422 to 480)	534 (424 to 664)	17.4% (-6.6 to 47.9%)	334 (309 to 349)	387 (344 to 429)	15.6% (4.0 to 35.2%)	7758 (7264 to 8068)	8345 (7570 to 9287)	7.6% (-3.5 to 28.7%)
Portugal	469 (438 to 502)	601 (459 to 806)	28.2% (-4.0 to 71.5%)	350 (328 to 387)	491 (426 to 613)	40.1% (19.9 to 64.3%)	9135 (8587 to 9713)	10544 (9228 to 13065)	15.4% (-1.5 to 39.1%)
San Marino	1 (1 to 1)	2 (1 to 3)	90.1% (32.9 to 172.3%)	1 (1 to 1)	1 (1 to 2)	94.5% (21.1 to 204.4%)	17 (13 to 23)	31 (19 to 49)	85.4% (14.6 to 205.5%)
Spain	2852 (2474 to 3034)	3894 (2956 to 5040)	36.5% (4.1 to 77.4%)	1596 (1403 to 1694)	2378 (1978 to 2675)	49.0% (29.6 to 68.0%)	41485 (35069 to 43893)	52223 (45154 to 58448)	25.9% (10.9 to 43.4%)
Sweden	984 (801 to 1050)	847 (674 to 1075)	-13.9% (-31.6 to 21.7%)	724 (597 to 772)	681 (602 to 833)	-6.0% (-16.2 to 41.8%)	16583 (13407 to 17587)	13938 (12524 to 17273)	-16.0% (-24.6 to 32.2%)
Switzerland	417 (386 to 448)	696 (524 to 888)	66.9% (23.8 to 116.9%)	286 (263 to 306)	509 (409 to 580)	77.9% (43.1 to 103.6%)	6541 (6065 to 6986)	10200 (8703 to 11431)	55.9% (30.1 to 78.6%)
United Kingdom	7041 (6466 to 7288)	7604 (6017 to 9532)	8.0% (-15.8 to 39.8%)	5135 (4745 to 5319)	5350 (4824 to 5819)	4.2% (-4.4 to 20.1%)	121788 (109997 to 125931)	110172 (101732 to 120850)	-9.5% (-17.1 to 9.7%)
**Southern Latin America**	1968 (1733 to 2328)	3631 (2833 to 4644)	84.5% (37.8 to 150.3%)	1469 (1289 to 1731)	2553 (2344 to 2877)	73.8% (40.2 to 113.7%)	40143 (35416 to 47236)	65518 (60158 to 73658)	63.2% (32.5 to 100.2%)
Argentina	1410 (1217 to 1707)	2485 (1924 to 3177)	76.3% (29.8 to 140.6%)	1070 (920 to 1291)	1760 (1592 to 2007)	64.4% (31.1 to 105.6%)	28974 (24943 to 34665)	45319 (41123 to 51209)	56.4% (25.2 to 94.3%)
Chile	397 (350 to 448)	896 (688 to 1161)	126.0% (67.3 to 209.2%)	275 (247 to 307)	604 (539 to 695)	119.6% (81.1 to 176.9%)	8002 (7122 to 8984)	15761 (13938 to 18121)	97.0% (59.6 to 154.1%)
Uruguay	161 (142 to 182)	250 (195 to 316)	54.9% (15.7 to 103.6%)	123 (109 to 139)	189 (167 to 210)	53.1% (27.1 to 79.7%)	3166 (2787 to 3605)	4435 (3960 to 4926)	40.1% (15.5 to 65.7%)
**Eastern Europe**	15646 (13500 to 16556)	18954 (15904 to 22903)	21.1% (3.1 to 50.9%)	11130 (9995 to 11728)	13285 (11081 to 15918)	19.4% (2.0 to 45.5%)	323951 (276933 to 342997)	360688 (299272 to 431833)	11.3% (-5.8 to 38.5%)
Belarus	690 (591 to 749)	699 (483 to 945)	1.3% (-28.3 to 42.1%)	545 (459 to 592)	518 (362 to 692)	-5.1% (-32.8 to 33.1%)	15155 (12787 to 16477)	14037 (9724 to 18895)	-7.4% (-35.0 to 30.7%)
Estonia	159 (136 to 172)	143 (106 to 190)	-9.8% (-33.3 to 33.3%)	123 (98 to 133)	115 (86 to 147)	-6.6% (-30.0 to 32.6%)	3196 (2763 to 3472)	2450 (1807 to 3281)	-23.3% (-43.2 to 16.6%)
Latvia	245 (224 to 265)	239 (175 to 315)	-2.7% (-28.8 to 29.4%)	202 (184 to 218)	207 (154 to 272)	2.3% (-24.1 to 34.8%)	5593 (5070 to 6037)	4789 (3460 to 6436)	-14.4% (-37.5 to 15.4%)
Lithuania	339 (308 to 364)	343 (272 to 425)	1.2% (-20.4 to 27.4%)	273 (245 to 292)	292 (234 to 357)	7.1% (-14.8 to 33.3%)	7470 (6831 to 8005)	6877 (5424 to 8598)	-7.9% (-28.2 to 17.2%)
Republic of Moldova	194 (133 to 213)	193 (147 to 234)	-0.9% (-23.7 to 47.9%)	141 (104 to 155)	144 (111 to 174)	2.3% (-20.3 to 46.1%)	4498 (3014 to 4942)	4134 (3159 to 5064)	-8.1% (-29.1 to 37.9%)
Russian Federation	10617 (8901 to 11134)	13197 (10458 to 16810)	24.3% (-0.1 to 68.0%)	7552 (6704 to 7897)	9379 (7369 to 11726)	24.2% (0.4 to 60.6%)	220045 (182905 to 230859)	251988 (196012 to 318184)	14.5% (-8.3 to 54.7%)
Ukraine	3402 (2861 to 3897)	4140 (3174 to 5285)	21.7% (-10.2 to 60.3%)	2295 (1952 to 2627)	2631 (2050 to 3285)	14.7% (-13.8 to 50.2%)	67994 (56561 to 77888)	76412 (58973 to 95883)	12.4% (-16.6 to 49.3%)
**Central Europe**	8726 (8283 to 9012)	11713 (9997 to 13583)	34.2% (15.7 to 55.7%)	6108 (5834 to 6322)	8650 (7446 to 10044)	41.6% (22.7 to 63.8%)	174509 (166029 to 180625)	210935 (181004 to 245579)	20.9% (4.8 to 40.0%)
Albania	40 (33 to 46)	93 (65 to 130)	131.1% (58.6 to 220.8%)	24 (21 to 30)	60 (42 to 84)	155.0% (75.0 to 251.7%)	790 (673 to 910)	1656 (1138 to 2314)	109.7% (42.9 to 194.7%)
Bosnia and Herzegovina	174 (153 to 218)	322 (241 to 427)	85.7% (34.1 to 143.3%)	116 (102 to 153)	232 (175 to 306)	100.5% (42.2 to 163.5%)	3591 (3160 to 4517)	6012 (4465 to 8029)	67.4% (20.7 to 120.6%)
Bulgaria	554 (509 to 610)	760 (553 to 979)	37.1% (-3.6 to 79.4%)	367 (338 to 412)	537 (399 to 681)	46.4% (2.6 to 89.3%)	10995 (10089 to 12152)	14000 (10105 to 18071)	27.3% (-10.7 to 67.8%)
Croatia	510 (447 to 567)	505 (383 to 653)	-1.0% (-25.6 to 33.6%)	303 (265 to 336)	318 (245 to 407)	4.9% (-20.4 to 38.7%)	8213 (7182 to 9101)	7302 (5510 to 9511)	-11.1% (-33.9 to 20.2%)
Czechia	1034 (886 to 1091)	1210 (961 to 1522)	17.1% (-7.8 to 51.5%)	704 (596 to 744)	825 (658 to 1024)	17.2% (-7.4 to 48.6%)	18570 (15699 to 19615)	18883 (14883 to 23768)	1.7% (-20.3 to 32.6%)
Hungary	939 (885 to 995)	1051 (859 to 1291)	11.9% (-9.4 to 39.0%)	674 (632 to 714)	786 (643 to 953)	16.5% (-5.1 to 42.5%)	17657 (16637 to 18756)	18316 (14796 to 22678)	3.7% (-17.2 to 29.2%)
Montenegro	28 (22 to 37)	44 (32 to 59)	58.5% (14.5 to 102.3%)	17 (14 to 24)	30 (21 to 40)	69.8% (22.6 to 116.4%)	513 (400 to 678)	790 (571 to 1060)	54.0% (11.1 to 97.9%)
North Macedonia	84 (71 to 98)	168 (121 to 221)	100.3% (43.4 to 164.6%)	55 (48 to 66)	114 (84 to 148)	106.8% (47.5 to 172.2%)	1727 (1454 to 2016)	3139 (2280 to 4113)	81.8% (30.0 to 140.7%)
Poland	2972 (2809 to 3074)	4292 (3297 to 5473)	44.4% (10.1 to 84.4%)	2230 (2129 to 2299)	3415 (2649 to 4322)	53.1% (18.9 to 94.0%)	63820 (60104 to 65744)	82012 (63412 to 104731)	28.5% (-1.7 to 64.1%)
Romania	1337 (1239 to 1420)	1700 (1373 to 2075)	27.2% (1.5 to 55.6%)	874 (822 to 926)	1216 (983 to 1470)	39.1% (11.6 to 70.7%)	27476 (25055 to 29039)	31036 (24980 to 37886)	13.0% (-9.7 to 38.5%)
Serbia	526 (439 to 645)	850 (625 to 1090)	61.6% (9.1 to 115.7%)	382 (316 to 471)	639 (476 to 819)	67.3% (14.4 to 123.5%)	11261 (9350 to 13942)	16125 (11864 to 20721)	43.2% (-3.5 to 93.1%)
Slovakia	357 (269 to 400)	533 (364 to 720)	49.1% (-1.0 to 103.6%)	228 (171 to 256)	327 (223 to 437)	43.3% (-5.5 to 92.7%)	6409 (4756 to 7215)	8291 (5635 to 11251)	29.4% (-14.5 to 78.0%)
Slovenia	170 (125 to 224)	183 (135 to 243)	7.5% (-30.3 to 58.9%)	133 (100 to 174)	151 (112 to 199)	13.4% (-25.3 to 65.1%)	3486 (2554 to 4627)	3371 (2485 to 4529)	-3.3% (-37.9 to 45.4%)
**Central Asia**	1440 (1217 to 1591)	3187 (2759 to 3567)	121.4% (87.3 to 165.1%)	953 (803 to 1056)	2044 (1768 to 2287)	114.5% (81.0 to 156.3%)	30232 (25688 to 33516)	64750 (56175 to 72871)	114.2% (80.7 to 157.3%)
Armenia	103 (80 to 148)	168 (134 to 204)	62.8% (3.3 to 127.4%)	71 (55 to 98)	121 (98 to 147)	71.0% (12.2 to 136.3%)	2142 (1675 to 3042)	3228 (2560 to 3951)	50.7% (-5.3 to 110.6%)
Azerbaijan	108 (78 to 144)	327 (210 to 451)	202.0% (89.3 to 333.1%)	69 (47 to 93)	201 (131 to 274)	192.2% (92.6 to 323.7%)	2342 (1680 to 3102)	6779 (4315 to 9260)	189.5% (81.1 to 315.6%)
Georgia	157 (132 to 196)	282 (187 to 349)	79.9% (1.5% to 141.2%)	105 (89 to 132)	210 (135 to 259)	99.3% (10.0 to 163.4%)	3191 (2678 to 4035)	5668 (3732 to 7032)	77.6% (-0.1% to 136.8%)
Kazakhstan	623 (486 to 716)	1048 (852 to 1254)	68.1% (35.8 to 111.3%)	411 (328 to 474)	668 (552 to 799)	62.6% (31.4 to 104.0%)	13047 (10168 to 15048)	20673 (16793 to 24821)	58.4% (28.0% to 98.6%)
Kyrgyzstan	100 (88 to 111)	205 (159 to 247)	105.9% (61.0 to 156.1%)	70 (61 to 78)	131 (102 to 157)	87.4% (47.6 to 134.9%)	2127 (1869 to 2382)	4228 (3268 to 5081)	98.8% (55.8 to 149.7%)
Mongolia	24 (16 to 45)	97 (68 to 143)	303.5% (139.5 to 543.7%)	17 (11 to 30)	63 (45 to 88)	276.0% (124.0 to 506.8%)	558 (368 to 1043)	2138 (1501 to 3097)	282.9% (130.5 to 513.1%)
Tajikistan	66 (45 to 90)	174 (133 to 229)	165.6% (79.3 to 298.9%)	48 (30 to 66)	117 (90 to 148)	141.1% (65.5 to 276.6%)	1403 (973 to 1962)	3814 (2896 to 5076)	171.8% (81.6 to 313.0%)
Turkmenistan	63 (48 to 71)	133 (94 to 173)	109.3% (48.1 to 193.9%)	36 (30 to 41)	74 (53 to 97)	104.6% (46.2 to 180.0%)	1338 (1023 to 1503)	2691 (1898 to 3497)	101.1% (42.2 to 178.8%)
Uzbekistan	196 (102 to 245)	753 (585 to 921)	284.9% (173.8 to 559.2%)	125 (62 to 158)	459 (356 to 560)	265.9% (153.6 to 550.1%)	4083 (2143 to 5168)	15532 (11987 to 19026)	280.4% (169.5 to 535.3%)
**Central Latin America**	2642 (2555 to 2752)	9795 (8127 to 11851)	270.8% (207.5 to 343.9%)	1690 (1626 to 1800)	6141 (5096 to 7294)	263.3% (200.9 to 329.5%)	55783 (54063 to 58415)	183814 (152067 to 220400)	229.5% (171.3 to 292.8%)
Colombia	752 (709 to 801)	2117 (1609 to 2888)	181.5% (113.0 to 286.1%)	488 (460 to 517)	1328 (1018 to 1815)	172.1% (108.0 to 266.4%)	15926 (14979 to 16964)	38219 (29230 to 52363)	140.0% (82.0 to 228.2%)
Costa Rica	36 (32 to 40)	171 (128 to 222)	380.2% (231.5 to 540.8%)	22 (20 to 25)	110 (83 to 144)	392.1% (245.2 to 547.0%)	688 (628 to 766)	2984 (2201 to 3905)	333.5% (206.3 to 479.4%)
El Salvador	42 (36 to 62)	210 (132 to 291)	399.1% (148.8 to 642.2%)	28 (24 to 43)	134 (87 to 181)	373.1% (132.5 to 586.3%)	933 (798 to 1373)	3946 (2473 to 5467)	322.9% (109.1 to 523.8%)
Guatemala	27 (22 to 34)	335 (196 to 440)	1150.3% (469.1 to 1652.0%)	18 (15 to 22)	208 (127 to 270)	1069.6% (446.2 to 1516.1%)	662 (547 to 807)	6718 (3987 to 8848)	914.9% (415.5 to 1324.7%)
Honduras	39 (29 to 60)	204 (95 to 441)	425.0% (172.3 to 812.3%)	27 (21 to 40)	138 (68 to 295)	403.7% (180.4 to 836.6%)	902 (676 to 1398)	4233 (1982 to 9179)	369.2% (144.5 to 718.8%)
Mexico	1587 (1533 to 1642)	5341 (4256 to 6599)	236.5% (165.0 to 316.4%)	1007 (971 to 1051)	3340 (2688 to 4120)	231.6% (164.1 to 304.0%)	33320 (32278 to 34443)	101537 (81437 to 126005)	204.7% (141.3 to 274.1%)
Nicaragua	25 (19 to 35)	151 (102 to 193)	493.7% (249.5 to 776.0%)	16 (11 to 20)	92 (61 to 116)	491.8% (237.9 to 838.2%)	547 (403 to 752)	2809 (1918 to 3601)	413.0% (200.0 to 664.9%)
Panama	28 (26 to 32)	130 (97 to 171)	359.2% (226.2 to 521.6%)	19 (17 to 21)	84 (63 to 109)	350.1% (221.1 to 498.4%)	577 (518 to 653)	2334 (1719 to 3060)	304.8% (187.7 to 448.7%)
Venezuela (Bolivarian Republic of)	106 (92 to 184)	1143 (654 to 1542)	981.6% (240.3 to 1466.3%)	66 (57 to 119)	711 (413 to 953)	986.1% (230.8 to 1457.8%)	2251 (1957 to 3890)	21182 (12247 to 28850)	844.3% (199.0 to 1281.4%)
**Andean Latin America**	471 (378 to 693)	2153 (1522 to 2768)	357.2% (179.6 to 556.6%)	320 (258 to 465)	1370 (968 to 1735)	328.7% (170.7 to 500.0%)	10643 (8517 to 15737)	40930 (29298 to 52538)	284.6% (135.1 to 448.0%)
Bolivia	64 (40 to 154)	315 (213 to 505)	389.9% (185.0 to 674.5%)	48 (29 to 114)	217 (148 to 360)	354.6% (165.5 to 606.9%)	1554 (966 to 3761)	6571 (4407 to 10490)	322.9% (144.2 to 577.6%)
Ecuador	71 (57 to 122)	601 (353 to 811)	743.6% (224.2 to 1222.9%)	48 (38 to 83)	383 (221 to 513)	705.0% (201.3 to 1151.7%)	1627 (1296 to 2782)	11369 (6729 to 15407)	598.9% (173.2 to 996.3%)
Peru	335 (251 to 466)	1237 (843 to 1682)	268.8% (117.2 to 467.0%)	224 (170 to 300)	771 (534 to 1044)	243.4% (108.8 to 414.4%)	7463 (5545 to 10295)	22990 (15753 to 31437)	208.1% (82.7 to 373.8%)
**Caribbean**	287 (252 to 441)	1527 (1102 to 2044)	432.7% (152.6 to 569.4%)	191 (167 to 296)	1039 (734 to 1396)	444.5% (152.5 to 582.7%)	5993 (5185 to 9690)	29259 (21130 to 40851)	388.3% (138.4 to 518.2%)
Antigua and Barbuda	1 (1 to 1)	5 (3 to 6)	778.8% (170.4 to 1067.8%)	0 (0 to 1)	4 (2 to 4)	773.9% (159.6% to 1062.4%)	11 (10 to 21)	98 (60 to 120)	768.8% (167.0 to 1063.4%)
Bahamas	4 (3 to 6)	26 (17 to 33)	597.8% (193.7 to 836.7%)	2 (2 to 4)	16 (11 to 20)	606.8% (198.3 to 839.1%)	75 (65 to 119)	495 (337 to 637)	558.3% (182.0 to 782.6%)
Barbados	3 (3 to 5)	23 (11 to 29)	669.4% (107.3 to 904.7%)	2 (2 to 4)	17 (8 to 21)	664.1% (105.8 to 888.5%)	58 (50 to 103)	429 (211 to 537)	644.5% (103.4 to 876.2%)
Belize	1 (1 to 1)	7 (5 to 8)	1002.2% (458.9 to 1330.9%)	0 (0 to 1)	4 (3 to 5)	932.9% (396.3 to 1231.3%)	14 (12 to 18)	140 (98 to 169)	903.7% (412.3 to 1191.2%)
Bermuda	4 (3 to 5)	6 (5 to 8)	70.5% (13.6 to 133.8%)	3 (2 to 3)	4 (4 to 6)	74.3% (18.6 to 137.4%)	67 (55 to 89)	97 (78 to 123)	45.9% (-3.5 to 101.0%)
Cuba	97 (87 to 152)	534 (303 to 673)	450.6% (103.2 to 628.1%)	60 (54 to 97)	356 (197 to 447)	492.1% (107.4 to 683.3%)	1895 (1704 to 2970)	9702 (5502 to 12316)	412.0% (90.7 to 580.7%)
Dominica	1 (0 to 1)	2 (1 to 3)	230.2% (27.4 to 409.5%)	0 (0 to 1)	1 (1 to 2)	243.1% (23.5% to 431.6%)	12 (10 to 19)	39 (21 to 53)	217.5% (27.6 to 391.8%)
Dominican Republic	32 (26 to 46)	129 (83 to 194)	301.4% (156.4 to 501.4%)	21 (17 to 31)	84 (54 to 125)	296.3% (154.1 to 501.8%)	722 (573 to 1032)	2571 (1654 to 3889)	256.1% (125.8 to 441.5%)
Grenada	1 (1 to 2)	6 (4 to 8)	577.6% (143.2 to 766.1%)	1 (1 to 1)	4 (3 to 5)	530.7% (120.3 to 705.1%)	19 (16 to 33)	126 (80 to 150)	552.8% (135.0 to 740.8%)
Guyana	7 (6 to 13)	44 (29 to 59)	511.6% (123.2 to 805.9%)	5 (4 to 9)	28 (19 to 37)	489.7% (119.2 to 762.6%)	162 (128 to 293)	929 (628 to 1251)	473.9% (110.4 to 752.1%)
Haiti	45 (23 to 187)	202 (101 to 647)	351.4% (129.6 to 723.7%)	32 (17 to 129)	138 (69 to 429)	327.6% (111.1 to 693.3%)	1132 (573 to 4741)	4709 (2335 to 15181)	316.2% (111.7 to 673.7%)
Jamaica	17 (15 to 28)	127 (73 to 163)	640.9% (158.7 to 907.2%)	12 (11 to 19)	85 (50 to 108)	608.3% (149.8 to 839.0%)	344 (302 to 549)	2389 (1391 to 3089)	594.4% (146.1 to 843.9%)
Puerto Rico	46 (42 to 66)	214 (154 to 277)	360.4% (138.9 to 516.7%)	32 (28 to 43)	157 (111 to 201)	391.7% (155.8 to 550.7%)	872 (780 to 1237)	3591 (2551 to 4656)	311.5% (113.5 to 451.1%)
Saint Kitts and Nevis	1 (1 to 1)	4 (2 to 5)	450.6% (178.7 to 680.1%)	0 (0 to 1)	2 (2 to 3)	423.6% (146.9 to 603.3%)	13 (11 to 18)	69 (46 to 91)	445.8% (179.8 to 662.3%)
Saint Lucia	1 (1 to 2)	10 (7 to 13)	643.0% (226.3 to 903.5%)	1 (1 to 1)	7 (5 to 9)	620.4% (203.4 to 867.0%)	29 (25 to 43)	198 (134 to 250)	588.5% (200.3 to 834.6%)
Saint Vincent and the Grenadines	1 (1 to 2)	6 (4 to 7)	480.6% (134.8 to 634.2%)	1 (1 to 1)	4 (3 to 5)	480.5% (132.0 to 632.8%)	20 (17 to 36)	116 (87 to 138)	473.0% (134.1 to 634.1%)
Suriname	4 (3 to 7)	28 (15 to 37)	665.6% (173.2 to 1108.9%)	3 (2 to 5)	20 (10 to 26)	659.9% (167.4 to 1070.0%)	82 (61 to 144)	581 (303 to 757)	609.9% (152.5 to 1019.8%)
Trinidad and Tobago	9 (8 to 15)	90 (39 to 123)	896.2% (143.7 to 1349.7%)	6 (5 to 10)	63 (27 to 86)	920.0% (155.8 to 1360.7%)	188 (166 to 319)	1752 (754 to 2421)	832.4% (131.3 to 1258.7%)
United States Virgin Islands	4 (3 to 6)	13 (9 to 16)	235.2% (86.5 to 393.6%)	3 (2 to 4)	10 (7 to 12)	274.8% (108.9 to 443.6%)	78 (60 to 121)	236 (171 to 301)	202.5% (70.1 to 348.2%)
**Tropical Latin America**	3120 (2976 to 3245)	8058 (7382 to 8806)	158.2% (135.6 to 184.5%)	2089 (1986 to 2177)	5436 (4963 to 5914)	160.3% (137.0 to 185.1%)	66749 (63747 to 69433)	153726 (140925 to 167334)	130.3% (109.4 to 152.6%)
Brazil	3089 (2940 to 3213)	7885 (7228 to 8641)	155.3% (132.6 to 182.0%)	2068 (1965 to 2155)	5325 (4864 to 5804)	157.5% (135.3% to 182.6%)	66081 (63024 to 68762)	150454 (138189 to 163533)	127.7% (107.3 to 150.2%)
Paraguay	31 (25 to 40)	173 (96 to 239)	452.5% (165.6 to 727.9%)	21 (16 to 27)	111 (61 to 153)	439.7% (148.6 to 719.5%)	668 (528 to 838)	3273 (1820 to 4560)	389.7% (137.5 to 639.7%)
**East Asia**	13419 (10535 to 18334)	47852 (35082 to 59600)	256.6% (117.7 to 398.2%)	8433 (6502 to 12138)	30350 (22124 to 38066)	259.9% (114.2 to 416.7%)	288347 (222940 to 393416)	871645 (645106 to 1098317)	202.3% (87.6 to 329.2%)
China	12680 (9905 to 17511)	45482 (33113 to 57376)	258.7% (113.9 to 406.7%)	8035 (6182 to 11673)	29092 (20956 to 36860)	262.0% (109.9 to 426.4%)	275060 (212261 to 377972)	835056 (612557 to 1063247)	203.6% (85.7 to 335.2%)
Democratic People's Republic of Korea	328 (162 to 706)	810 (536 to 1310)	147.0% (36.6 to 392.8%)	190 (95 to 392)	484 (329 to 762)	155.4% (44.2 to 410.2%)	6402 (3115 to 13618)	14647 (9589 to 23982)	128.8% (25.8 to 370.9%)
Taiwan (province of China)	411 (384 to 436)	1560 (1137 to 2078)	279.7% (170.7 to 407.9%)	208 (196 to 220)	774 (567 to 1022)	272.7% (166.2 to 394.6%)	6885 (6475 to 7290)	21942 (15920 to 29388)	218.7% (127.0 to 331.3%)
**Southeast Asia**	8175 (6454 to 12130)	28151 (21777 to 38998)	244.3% (108.9 to 334.9%)	5094 (4105 to 7253)	16187 (12722 to 22681)	217.8% (100.9 to 296.3%)	174387 (136906 to 257864)	517385 (404021 to 726116)	196.7% (78.7 to 275.4%)
Cambodia	119 (62 to 353)	647 (421 to 1091)	443.2% (161.2 to 924.5%)	78 (43 to 221)	382 (257 to 644)	387.2% (139.6 to 856.8%)	2774 (1438 to 8309)	12508 (8109 to 21122)	350.8% (112.5 to 757.2%)
Indonesia	2961 (2077 to 5708)	10543 (6547 to 19733)	256.1% (89.5 to 414.5%)	1841 (1371 to 3407)	6115 (3870 to 11649)	232.1% (81.4 to 369.4%)	65692 (47198 to 124857)	202839 (127003 to 381129)	208.8% (59.5% to 346.4%)
Lao People's Democratic Republic	57 (28 to 179)	225 (144 to 363)	297.4% (65.7 to 671.3%)	39 (20 to 117)	130 (86 to 206)	231.8% (47.0 to 537.7%)	1368 (668 to 4368)	4551 (2887 to 7336)	232.7% (38.9% to 555.5%)
Malaysia	277 (213 to 415)	1182 (874 to 1612)	327.4% (148.1 to 529.4%)	174 (136 to 252)	656 (493 to 880)	278.1% (123.8 to 459.4%)	5641 (4330 to 8508)	20093 (14850 to 27396)	256.2% (102.3 to 431.9%)
Maldives	4 (2 to 13)	17 (13 to 23)	353.9% (11.3 to 840.4%)	2 (1 to 7)	8 (6 to 11)	264.8% (-2.4 to 625.3%)	80 (42 to 266)	262 (202 to 353)	228.3% (-19.3 to 589.3%)
Mauritius	24 (21 to 26)	85 (66 to 107)	259.4% (180.4 to 362.2%)	13 (12 to 15)	49 (38 to 62)	264.2% (186.1 to 369.3%)	443 (396 to 489)	1449 (1129 to 1851)	227.4% (154.7 to 322.4%)
Myanmar	783 (480 to 1831)	2299 (1600 to 3283)	193.5% (37.1 to 396.3%)	527 (334 to 1181)	1392 (994 to 1935)	164.1% (24.9 to 333.8%)	18024 (10903 to 42917)	44905 (31561 to 63587)	149.1% (8.7 to 318.0%)
Philippines	1517 (1188 to 1958)	4805 (3553 to 6526)	216.8% (113.6 to 365.4%)	862 (665 to 1122)	2604 (1926 to 3594)	202.1% (106.7 to 358.4%)	30877 (24260 to 39020)	88930 (65579 to 119141)	188.0% (95.9 to 329.8%)
Sri Lanka	215 (181 to 269)	790 (529 to 1086)	268.0% (145.3 to 418.0%)	124 (103 to 161)	451 (306 to 618)	264.6% (139.1 to 416.5%)	4245 (3581 to 5364)	12888 (8556 to 17753)	203.6% (101.1 to 331.3%)
Seychelles	3 (2 to 4)	9 (7 to 11)	233.0% (132.8 to 336.5%)	2 (1 to 2)	5 (4 to 6)	201.8% (108.1 to 294.7%)	52 (43 to 73)	155 (121 to 189)	197.0% (108.5 to 291.3%)
Thailand	1254 (1011 to 1577)	3619 (2510 to 5360)	188.7% (79.6 to 344.4%)	751 (609 to 949)	2068 (1463 to 2977)	175.3% (77.4 to 306.1%)	25173 (20454 to 31774)	59805 (41070 to 88579)	137.6% (49.3 to 263.0%)
Timor-Leste	6 (3 to 16)	26 (15 to 38)	338.8% (84.7 to 778.9%)	4 (2 to 10)	15 (10 to 22)	299.2% (92.4 to 669.7%)	142 (68 to 380)	510 (290 to 740)	258.3% (52.9 to 616.3%)
Viet Nam	947 (719 to 1251)	3867 (2814 to 5026)	308.4% (168.5 to 486.0%)	669 (506 to 864)	2291 (1630 to 2982)	242.1% (129.5 to 380.1%)	19644 (14865 to 26289)	67812 (48461 to 89349)	245.2% (124.6 to 400.9%)
**Oceania**	48 (34 to 104)	184 (121 to 362)	281.1% (156.0 to 443.5%)	31 (22 to 64)	110 (74 to 209)	254.1% (134.2 to 408.9%)	1072 (746 to 2321)	3792 (2478 to 7547)	253.8% (134.4 to 411.6%)
American Samoa	1 (1 to 2)	4 (3 to 6)	208.7% (95.6 to 380.7%)	1 (1 to 1)	2 (2 to 3)	218.0% (99.8 to 386.1%)	26 (19 to 38)	74 (55 to 105)	185.5% (81.9 to 354.2%)
Cook Islands	0 (0 to 0)	1 (0 to 1)	232.2% (81.4 to 440.1%)	0 (0 to 0)	0 (0 to 0)	245.6% (93.5 to 446.3%)	3 (2 to 5)	10 (7 to 13)	199.0% (62.7 to 389.1%)
Micronesia(Federated States of)	1 (1 to 3)	4 (2 to 7)	242.2% (60.6 to 599.6%)	1 (0 to 2)	2 (1 to 4)	215.9% (54.2 to 542.5%)	23 (14 to 59)	71 (38 to 138)	207.2% (44.6% to 534.0%)
Fiji	5 (4 to 7)	13 (9 to 17)	143.2% (61.2 to 258.0%)	3 (2 to 4)	8 (6 to 10)	147.0% (62.9 to 260.0%)	112 (85 to 154)	250 (179 to 336)	124.1% (46.0 to 232.0%)
Guam	2 (1 to 3)	6 (5 to 8)	235.4% (116.5 to 364.2%)	1 (1 to 2)	4 (3 to 5)	267.7% (140.3 to 407.5%)	34 (27 to 52)	107 (84 to 137)	216.2% (104.3 to 338.8%)
Kiribati	0 (0 to 0)	1 (0 to 1)	216.1% (72.8 to 384.0%)	0 (0 to 0)	0 (0 to 0)	199.1% (68.0 to 352.1%)	3 (2 to 5)	10 (7 to 13)	192.9% (61.3 to 346.7%)
Marshall Islands	1 (0 to 1)	2 (1 to 3)	318.3% (141.9 to 638.2%)	0 (0 to 1)	1 (1 to 2)	289.7% (124.8 to 589.6%)	13 (10 to 18)	38 (24 to 56)	298.3% (132.0 to 598.0%)
Nauru	0 (0 to 1)	2 (1 to 3)	127.9% (25.3 to 365.0%)	0 (0 to 1)	1 (0 to 2)	104.7% (12.3 to 323.5%)	8 (5 to 19)	32 (17 to 67)	105.7% (13.0 to 327.3%)
Niue	0 (0 to 0)	0 (0 to 0)	66.2% (-5.1 to 170.1%)	0 (0 to 0)	0 (0 to 0)	47.4% (-14.1 to 133.9%)	2 (1 to 5)	5 (3 to 9)	49.6% (-13.6 to 143.5%)
Northern Mariana Islands	0 (0 to 0)	0 (0 to 0)	296.1% (116.3 to 549.2%)	0 (0 to 0)	0 (0 to 0)	392.9% (169.4 to 694.0%)	1 (1 to 2)	2 (1 to 2)	295.5% (117.8 to 551.6%)
Palau	1 (0 to 1)	2 (2 to 3)	206.9% (84.3 to 365.6%)	0 (0 to 0)	1 (1 to 2)	193.5% (75.8 to 341.8%)	9 (6 to 17)	37 (28 to 49)	189.4% (71.6 to 338.3%)
Papua New Guinea	26 (16 to 64)	112 (66 to 242)	329.9% (172.1 to 576.8%)	17 (10 to 40)	66 (40 to 140)	288.7% (140.5 to 509.9%)	599 (362 to 1470)	2379 (1410 to 5179)	297.3% (147.4 to 531.1%)
Samoa	4 (3 to 5)	11 (6 to 19)	167.8% (50.6 to 349.8%)	3 (2 to 4)	6 (3 to 11)	146.7% (39.9 to 305.0%)	81 (57 to 112)	198 (102 to 354)	143.5% (36.4 to 312.6%)
Solomon Islands	2 (1 to 11)	13 (6 to 44)	466.9% (195.1 to 1096.1%)	1 (1 to 6)	7 (4 to 22)	408.4% (167.6 to 963.5%)	51 (23 to 244)	256 (130 to 859)	398.6% (162.6 to 941.8%)
Tokelau	0 (0 to 0)	0 (0 to 0)	78.4% (-3.8 to 231.1%)	0 (0 to 0)	0 (0 to 0)	52.8% (-17.2 to 186.4%)	0 (0 to 1)	1 (0 to 1)	55.0% (-17.5 to 194.8%)
Tonga	1 (1 to 2)	3 (2 to 4)	125.5% (43.6 to 232.2%)	1 (0 to 1)	2 (1 to 3)	118.7% (39.8 to 217.5%)	25 (16 to 33)	50 (31 to 73)	99.9% (25.4 to 196.4%)
Tuvalu	0 (0 to 0)	0 (0 to 1)	129.9% (30.7 to 281.4%)	0 (0 to 0)	0 (0 to 0)	117.1% (24.5 to 257.4%)	4 (2 to 9)	7 (4 to 13)	101.2% (14.0 to 232.4%)
Vanuatu	1 (0 to 3)	4 (2 to 10)	446.5% (202.0 to 987.1%)	0 (0 to 2)	3 (1 to 6)	428.9% (192.2 to 928.2%)	17 (8 to 57)	87 (42 to 214)	407.2% (176.2 to 920.8%)
**North Africa and Middle East**	6589 (4722 to 12191)	25753 (20789 to 30142)	290.8% (100.8 to 438.2%)	4582 (3322 to 8255)	15651 (12975 to 18273)	241.6% (81.9 to 371.2%)	147202 (104972 to 277066)	487631 (396614 to 568566)	231.3% (72.8 to 362.3%)
Afghanistan	102 (37 to 565)	331 (162 to 1304)	224.7% (110.0 to 476.4%)	79 (29 to 416)	218 (105 to 849)	177.0% (80.1 to 380.0%)	2613 (942 to 14789)	7970 (3863 to 32130)	205.0% (98.1 to 451.4%)
Algeria	191 (136 to 260)	776 (511 to 1025)	306.4% (150.8 to 487.4%)	125 (90 to 170)	466 (303 to 614)	273.5% (135.0 to 427.1%)	4130 (2916 to 5613)	14712 (9617 to 19399)	256.2% (120.0 to 408.1%)
Bahrain	9 (6 to 13)	50 (34 to 81)	490.9% (201.6 to 953.2%)	6 (4 to 8)	29 (20 to 44)	410.7% (165.6 to 813.3%)	182 (137 to 265)	920 (624 to 1467)	406.2% (157.1 to 804.2%)
Egypt	312 (236 to 565)	1205 (660 to 2119)	286.6% (92.2 to 502.2%)	207 (150 to 416)	707 (389 to 1354)	242.0% (84.4 to 434.3%)	7172 (5431 to 13032)	24106 (13213 to 43379)	236.1% (69.3% to 422.7%)
Iran	369 (243 to 693)	1944 (1318 to 2216)	426.3% (143.6 to 676.9%)	223 (152 to 412)	1148 (818 to 1302)	415.4% (146.1 to 659.7%)	7744 (5050 to 14528)	35213 (24068 to 40121)	354.7% (113.2 to 568.5%)
Iraq	156 (88 to 273)	931 (647 to 1313)	496.5% (157.8 to 1131.2%)	102 (56 to 189)	498 (357 to 683)	388.6% (126.6 to 912.4%)	3454 (1956 to 6028)	17602 (12223 to 24826)	409.6% (124.9 to 982.5%)
Jordan	30 (21 to 48)	210 (148 to 276)	600.5% (238.8 to 1009.9%)	19 (13 to 31)	118 (85 to 154)	512.3% (206.9 to 888.7%)	647 (443 to 1026)	3808 (2685 to 4991)	488.6% (190.4 to 861.9%)
Kuwait	23 (20 to 31)	87 (54 to 134)	268.7% (138.0 to 511.0%)	12 (10 to 16)	43 (27 to 63)	249.9% (121.7 to 473.5%)	429 (354 to 566)	1430 (897 to 2178)	233.5% (115.0 to 447.5%)
Lebanon	87 (61 to 134)	334 (228 to 444)	286.1% (80.5 to 510.1%)	60 (43 to 91)	214 (145 to 285)	254.7% (65.2 to 460.6%)	1829 (1283 to 2827)	5709 (3866 to 7641)	212.0% (47.4 to 398.5%)
Libya	41 (26 to 70)	214 (150 to 311)	417.6% (162.5 to 890.6%)	29 (18 to 48)	135 (96 to 191)	371.6% (141.7 to 819.0%)	876 (547 to 1489)	4186 (2926 to 6103)	378.0% (142.7 to 824.8%)
Morocco	309 (228 to 454)	1350 (893 to 1924)	336.6% (112.0 to 596.8%)	225 (168 to 324)	896 (607 to 1237)	297.9% (99.8 to 522.8%)	7175 (5284 to 10477)	27877 (18349 to 39840)	288.6% (88.6 to 523.9%)
Palestine	18 (10 to 37)	100 (58 to 126)	442.3% (133.8 to 996.2%)	12 (7 to 24)	59 (34 to 74)	380.4% (111.4 to 862.5%)	388 (204 to 794)	1874 (1085 to 2347)	382.9% (107.0 to 877.4%)
Oman	10 (5 to 17)	67 (42 to 88)	585.9% (148.5 to 1328.3%)	6 (3 to 12)	36 (23 to 47)	448.7% (106.8 to 1018.1%)	205 (99 to 368)	1159 (724 to 1548)	466.4% (103.7 to 1070.6%)
Qatar	3 (2 to 5)	39 (26 to 55)	1094.0% (394.3 to 2013.6%)	2 (1 to 3)	20 (12 to 27)	846.1% (315.0 to 1551.4%)	70 (45 to 118)	673 (430 to 936)	867.5% (290.3 to 1617.3%)
Saudi Arabia	72 (47 to 130)	735 (509 to 1047)	917.4% (373.8 to 1695.7%)	58 (37 to 106)	351 (243 to 497)	506.9% (172.6 to 989.1%)	1952 (1257 to 3484)	13042 (9054 to 18662)	568.1% (202.3 to 1109.7%)
Sudan	84 (34 to 331)	358 (209 to 639)	327.6% (87.3 to 844.5%)	60 (23 to 228)	211 (132 to 368)	252.3% (65.6 to 663.8%)	2002 (826 to 8094)	7301 (4393 to 13100)	264.7% (63.1 to 724.8%)
Syrian Arab Republic	62 (41 to 100)	248 (159 to 373)	302.8% (70.0 to 685.7%)	38 (25 to 61)	147 (95 to 222)	284.7% (70.3 to 638.5%)	1348 (878 to 2201)	4760 (3039 to 7205)	253.1% (49.5 to 587.4%)
Tunisia	93 (69 to 128)	358 (237 to 496)	285.2% (111.3 to 505.3%)	63 (47 to 87)	228 (154 to 314)	260.5% (101.4 to 467.2%)	1944 (1437 to 2707)	6523 (4355 to 9043)	235.6% (82.6 to 431.6%)
Turkey	1265 (715 to 2302)	3137 (1946 to 4122)	147.9% (21.5 to 288.4%)	925 (532 to 1629)	2067 (1280 to 2693)	123.6% (12.4 to 244.9%)	28082 (15827 to 50877)	56538 (35034 to 74620)	101.3% (-3.0 to 215.7%)
United Arab Emirates	14 (8 to 27)	169 (102 to 305)	1127.0% (476.8 to 2208.0%)	7 (4 to 16)	82 (49 to 155)	998.7% (401.4 to 1992.2%)	296 (162 to 584)	3304 (1982 to 6091)	1016.6% (423.8 to 2002.0%)
Yemen	42 (13 to 184)	220 (129 to 529)	424.1% (169.6 to 1241.1%)	31 (9 to 128)	144 (85 to 336)	367.9% (145.5 to 1096.9%)	1014 (302 to 4516)	4862 (2837 to 11815)	379.2% (146.1 to 1132.4%)
**South Asia**	9996 (7382 to 15060)	45756 (34899 to 56554)	357.7% (176.0 to 547.3%)	7328 (5505 to 10799)	32105 (24894 to 39896)	338.1% (166.3 to 510.7%)	240840 (175112 to 367236)	982473 (748576 to 1238008)	307.9% (144.7 to 477.9%)
Bangladesh	639 (377 to 1176)	3166 (1980 to 6892)	395.7% (104.9 to 738.4%)	471 (280 to 850)	2193 (1360 to 4702)	365.9% (102.7 to 664.6%)	15508 (9140 to 28619)	66485 (41904 to 145576)	328.7% (73.6 to 633.2%)
Bhutan	4 (2 to 9)	20 (11 to 46)	375.2% (75.2 to 819.5%)	3 (2 to 7)	14 (8 to 32)	335.9% (73.2 to 710.3%)	105 (50 to 225)	419 (234 to 946)	299.4% (45.5 to 691.4%)
India	7307 (4821 to 11444)	31024 (23462 to 38479)	324.6% (172.8 to 507.0%)	5335 (3587 to 8281)	22350 (16820 to 28024)	318.9% (177.6 to 503.3%)	175429 (115375 to 277756)	657740 (492035 to 826775)	274.9% (146.3 to 444.6%)
Nepal	131 (66 to 261)	764 (465 to 1743)	482.1% (133.7 to 957.4%)	99 (50 to 196)	546 (333 to 1227)	453.2% (139.9 to 895.7%)	3260 (1665 to 6554)	16425 (9944 to 37176)	403.8% (102.3% to 814.7%)
Pakistan	1915 (1481 to 2418)	10783 (5511 to 17344)	463.0% (157.2 to 887.8%)	1420 (1104 to 1762)	7002 (3672 to 11477)	393.0% (130.9 to 781.6%)	46538 (35377 to 57205)	241404 (122744 to 390862)	418.7% (139.2% to 823.9%)
**Southern sub-Saharan Africa**	817 (700 to 975)	2323 (1903 to 2786)	184.2% (116.0 to 238.0%)	581 (488 to 695)	1687 (1380 to 2011)	190.2% (128.3 to 248.0%)	17900 (15425 to 21180)	48660 (39766 to 58352)	171.8% (105.9 to 230.1%)
Botswana	15 (10 to 23)	75 (47 to 114)	382.8% (173.0 to 680.6%)	11 (7 to 16)	48 (31 to 72)	333.8% (149.4 to 584.7%)	351 (229 to 516)	1527 (945 to 2362)	335.3% (141.6 to 608.7%)
Lesotho	20 (13 to 30)	61 (33 to 117)	205.7% (61.2 to 503.6%)	16 (11 to 24)	47 (25 to 90)	193.9% (55.8 to 471.5%)	458 (295 to 692)	1385 (745 to 2708)	202.4% (58.4 to 501.5%)
Namibia	14 (9 to 19)	43 (29 to 63)	222.1% (86.5 to 429.2%)	11 (7 to 14)	31 (21 to 43)	193.5% (79.5 to 362.0%)	316 (214 to 445)	920 (614 to 1349)	190.9% (67.0 to 382.2%)
South Africa	616 (525 to 723)	1650 (1278 to 2052)	167.9% (91.0 to 224.0%)	439 (365 to 515)	1222 (934 to 1484)	178.5% (117.9 to 227.3%)	13455 (11429 to 15831)	33930 (25497 to 42265)	152.2% (80.7 to 206.5%)
Eswatini	11 (6 to 22)	31 (17 to 54)	193.8% (54.0 to 456.5%)	8 (4 to 15)	22 (12 to 38)	189.7% (55.5 to 437.9%)	247 (132 to 506)	685 (360 to 1188)	177.4% (43.9 to 430.2%)
Zimbabwe	142 (109 to 185)	461 (312 to 620)	225.9% (105.5 to 383.8%)	97 (75 to 127)	317 (215 to 433)	225.8% (102.6 to 388.8%)	3072 (2341 to 4006)	10212 (6807 to 14000)	232.4% (106.2 to 403.8%)
**Western sub-Saharan Africa**	1130 (841 to 1629)	4961 (3595 to 6761)	339.0% (158.2 to 567.2%)	849 (644 to 1214)	3406 (2427 to 4570)	301.4% (136.6 to 491.7%)	26988 (20466 to 38668)	111798 (80399 to 150518)	314.2% (148.5 to 511.4%)
Benin	25 (19 to 33)	110 (78 to 155)	335.1% (184.3 to 516.4%)	19 (14 to 24)	75 (55 to 103)	301.8% (171.1 to 451.1%)	615 (459 to 799)	2517 (1768 to 3542)	309.1% (173.2 to 486.1%)
Burkina Faso	55 (37 to 86)	209 (152 to 288)	277.8% (153.5 to 447.1%)	41 (28 to 65)	142 (106 to 194)	244.6% (132.7 to 396.2%)	1355 (917 to 2133)	4823 (3478 to 6699)	256.1% (137.0 to 416.2%)
Cameroon	87 (65 to 124)	411 (248 to 657)	370.2% (188.8 to 610.0%)	64 (47 to 90)	270 (160 to 430)	325.4% (161.2 to 552.3%)	2143 (1569 to 3027)	9223 (5500 to 14839)	330.5% (165.2 to 560.4%)
Cabo Verde	1 (1 to 2)	10 (5 to 14)	611.6% (177.7 to 1091.2%)	1 (1 to 1)	6 (3 to 9)	565.7% (138.4 to 1131.0%)	31 (21 to 45)	192 (98 to 268)	528.5% (147.1 to 951.5%)
Chad	26 (17 to 40)	76 (49 to 122)	195.2% (108.4 to 309.0%)	20 (13 to 30)	53 (35 to 83)	167.3% (89.4 to 266.7%)	638 (421 to 994)	1831 (1173 to 2944)	186.9% (101.3 to 297.6%)
Côte d’Ivoire	62 (46 to 82)	270 (181 to 386)	338.7% (177.8 to 570.8%)	42 (32 to 55)	180 (122 to 255)	329.8% (175.3 to 549.7%)	1520 (1141 to 2004)	6250 (4129 to 9009)	311.2% (161.6 to 534.7%)
Gambia	4 (3 to 5)	26 (15 to 41)	530.8% (224.7 to 1017.4%)	3 (2 to 4)	18 (10 to 29)	504.1% (216.9 to 963.4%)	96 (68 to 131)	576 (324 to 933)	498.1% (200.1 to 967.1%)
Ghana	108 (76 to 151)	561 (312 to 924)	419.2% (181.2 to 714.4%)	74 (52 to 105)	366 (204 to 612)	393.7% (167.5 to 676.3%)	2596 (1793 to 3644)	12288 (6777 to 20199)	373.3% (155.5 to 647.7%)
Guinea	59 (46 to 74)	174 (101 to 261)	192.5% (64.8 to 349.3%)	46 (36 to 58)	123 (71 to 185)	165.5% (47.1 to 313.4%)	1465 (1142 to 1834)	4101 (2357 to 6206)	179.9% (53.8 to 328.0%)
Guinea-Bissau	7 (4 to 12)	22 (14 to 36)	233.2% (96.5 to 418.3%)	5 (3 to 8)	15 (9 to 24)	210.6% (80.9 to 384.7%)	166 (107 to 293)	515 (327 to 841)	211.3% (86.4 to 385.9%)
Liberia	14 (10 to 21)	57 (34 to 94)	317.9% (126.1 to 586.1%)	11 (8 to 16)	39 (23 to 64)	265.9% (98.7 to 500.8%)	336 (248 to 502)	1322 (773 to 2148)	293.6% (113.2 to 545.2%)
Mali	39 (31 to 48)	135 (77 to 190)	250.1% (90.3 to 407.6%)	29 (23 to 36)	91 (52 to 126)	212.9% (70.1 to 347.9%)	972 (768 to 1199)	3148 (1777 to 4477)	223.9% (76.4 to 373.9%)
Mauritania	17 (13 to 23)	61 (30 to 94)	253.7% (71.7 to 460.2%)	13 (10 to 18)	42 (21 to 65)	218.6% (57.7 to 394.9%)	415 (323 to 555)	1333 (657 to 2078)	221.4% (53.2 to 415.2%)
Niger	27 (18 to 45)	111 (64 to 176)	309.3% (173.0 to 489.9%)	19 (13 to 32)	77 (46 to 122)	296.8% (163.3 to 464.9%)	693 (465 to 1140)	2621 (1514 to 4162)	278.2% (150.9 to 445.1%)
Nigeria	512 (317 to 902)	2353 (1495 to 3597)	359.7% (119.4 to 800.5%)	399 (250 to 696)	1646 (1060 to 2450)	312.5% (99.8 to 686.3%)	11832 (7338 to 21447)	52503 (34227 to 79231)	343.7% (110.9 to 735.7%)
São Tomé and Príncipe	1 (1 to 2)	6 (3 to 11)	328.5% (116.8 to 618.3%)	1 (1 to 1)	4 (2 to 7)	258.9% (88.5 to 506.3%)	35 (26 to 45)	138 (69 to 238)	294.7% (101.0 to 573.5%)
Senegal	44 (32 to 58)	190 (117 to 281)	332.1% (147.9 to 577.9%)	32 (23 to 42)	135 (84 to 197)	323.9% (143.8 to 560.4%)	1071 (784 to 1407)	4310 (2659 to 6387)	302.4% (130.1 to 536.1%)
Sierra Leone	21 (14 to 31)	81 (51 to 127)	283.9% (136.7 to 486.1%)	16 (11 to 23)	55 (35 to 85)	248.4% (111.2 to 422.1%)	514 (346 to 757)	1849 (1179 to 2895)	259.5% (117.4 to 451.7%)
Togo	20 (15 to 27)	99 (63 to 148)	384.9% (185.8 to 651.4%)	14 (10 to 18)	68 (42 to 102)	385.3% (183.6 to 658.2%)	494 (357 to 654)	2253 (1423 to 3395)	356.2% (168.7 to 620.1%)
**Eastern sub-Saharan Africa**	1770 (1097 to 3939)	6349 (5046 to 7696)	258.8% (77.9 to 470.6%)	1322 (829 to 2862)	4456 (3629 to 5408)	237.1% (69.6 to 421.4%)	44065 (27025 to 100756)	147571 (117400 to 181455)	234.9% (59.2 to 437.9%)
Burundi	61 (31 to 164)	129 (84 to 207)	111.1% (10.6 to 263.0%)	47 (24 to 122)	90 (58 to 143)	91.9% (0.9 to 224.4%)	1529 (757 to 4100)	3064 (1990 to 4955)	100.4% (2.8 to 252.6%)
Comoros	5 (2 to 8)	23 (14 to 36)	337.4% (107.3 to 918.2%)	4 (2 to 7)	18 (10 to 27)	316.6% (107.1 to 773.5%)	131 (55 to 214)	534 (307 to 820)	307.1% (91.6 to 899.5%)
Djibouti	3 (2 to 5)	26 (14 to 41)	647.2% (264.3 to 1298.4%)	2 (1 to 4)	18 (10 to 28)	621.1% (264.6 to 1228.5%)	87 (49 to 135)	605 (334 to 961)	597.2% (236.2 to 1213.5%)
Eritrea	22 (12 to 43)	119 (75 to 183)	450.4% (182.8 to 923.4%)	16 (9 to 33)	86 (53 to 133)	421.8% (164.0 to 864.7%)	573 (314 to 1148)	2867 (1770 to 4439)	400.3% (148.5 to 826.8%)
Ethiopia	431 (168 to 1923)	1298 (723 to 2123)	201.0% (7.1 to 593.2%)	328 (129 to 1365)	912 (493 to 1498)	178.3% (1.8 to 520.9%)	11188 (4304 to 50237)	29774 (16115 to 49909)	166.1% (-6.6 to 518.2%)
Kenya	137 (73 to 227)	785 (554 to 1068)	471.4% (174.5 to 958.4%)	99 (51 to 163)	591 (420 to 797)	496.9% (193.6 to 997.2%)	3218 (1674 to 5306)	19465 (13634 to 26642)	504.9% (190.0 to 1034.7%)
Madagascar	116 (72 to 191)	366 (225 to 562)	215.4% (90.5 to 401.0%)	83 (52 to 132)	251 (155 to 387)	201.8% (87.0 to 372.1%)	2892 (1785 to 4779)	8809 (5456 to 13687)	204.6% (84.6 to 381.9%)
Malawi	82 (60 to 109)	249 (136 to 389)	202.8% (41.9 to 441.9%)	60 (45 to 78)	174 (98 to 265)	189.2% (39.3 to 397.3%)	2012 (1463 to 2647)	5663 (3110 to 8896)	181.4% (30.9 to 407.5%)
Mozambique	141 (90 to 211)	451 (295 to 638)	220.8% (96.7 to 391.3%)	108 (71 to 159)	317 (215 to 448)	192.5% (85.6 to 347.2%)	3569 (2275 to 5365)	10555 (6794 to 15028)	195.8% (80.8 to 356.7%)
Rwanda	90 (50 to 210)	325 (223 to 450)	260.7% (33.4 to 628.5%)	68 (38 to 155)	228 (161 to 312)	236.4% (29.6 to 557.1%)	2274 (1241 to 5357)	7347 (4998 to 10138)	223.1% (18.6 to 561.8%)
Somalia	54 (28 to 112)	179 (102 to 334)	230.4% (100.7 to 416.3%)	41 (21 to 84)	136 (80 to 258)	232.6% (112.6 to 407.5%)	1432 (743 to 3028)	4609 (2668 to 8752)	221.7% (101.0 to 399.0%)
South Sudan	49 (31 to 76)	107 (65 to 157)	118.3% (27.8 to 242.4%)	37 (24 to 58)	78 (47 to 114)	109.1% (23.7 to 220.5%)	1207 (741 to 1858)	2654 (1544 to 3968)	119.9% (26.3 to 256.0%)
United Republic of Tanzania	303 (183 to 440)	1065 (782 to 1372)	251.6% (133.4 to 453.7%)	223 (137 to 321)	737 (555 to 933)	229.7% (124.3 to 396.6%)	7272 (4401 to 10669)	24102 (17581 to 31512)	231.4% (118.0 to 423.6%)
Uganda	183 (127 to 242)	874 (576 to 1330)	376.6% (185.7 to 693.3%)	139 (98 to 181)	592 (399 to 887)	326.6% (159.6 to 590.2%)	4401 (3015 to 5882)	19594 (12711 to 29927)	345.3% (161.9 to 656.6%)
Zambia	320 (186 to 677)	1120 (728 to 1809)	287.1% (113.9 to 577.0%)	242 (142 to 501)	800 (522 to 1299)	251.1% (100.5 to 499.2%)	8064 (4673 to 17296)	26272 (17015 to 43122)	247.5% (89.1 to 514.8%)
**Central sub-Saharan Africa**	90 (53 to 146)	348 (249 to 471)	249.6% (119.9 to 472.7%)	64 (38 to 103)	225 (160 to 304)	231.1% (108.3 to 433.2%)	2247 (1319 to 3715)	7809 (5448 to 10637)	225.8% (104.4 to 431.3%)
Angola	46 (24 to 109)	254 (145 to 431)	452.3% (222.2 to 830.3%)	34 (18 to 78)	174 (98 to 297)	407.3% (201.5 to 733.9%)	1191 (627 to 2877)	5927 (3328 to 10192)	397.7% (186.7 to 738.3%)
Central African Republic	17 (10 to 46)	38 (22 to 88)	119.3% (47.8 to 237.1%)	13 (8 to 34)	29 (16 to 64)	113.2% (42.2 to 226.5%)	452 (262 to 1203)	977 (553 to 2253)	116.2% (44.6 to 233.6%)
Congo	21 (14 to 44)	81 (51 to 130)	277.2% (119.0 to 521.5%)	16 (11 to 33)	56 (36 to 89)	243.3% (103.9 to 456.8%)	527 (348 to 1102)	1865 (1171 to 3012)	253.7% (104.9 to 490.4%)
Democratic Republic of the Congo	222 (124 to 460)	689 (437 to 1130)	210.6% (89.8 to 429.3%)	167 (94 to 337)	501 (312 to 830)	200.4% (82.4 to 407.8%)	5569 (3087 to 11645)	16243 (10252 to 27031)	191.6% (75.1 to 401.0%)
Equatorial Guinea	2 (1 to 7)	18 (10 to 30)	673.5% (113.9 to 1762.0%)	2 (1 to 5)	12 (7 to 19)	542.7% (86.1 to 1419.0%)	59 (27 to 173)	383 (218 to 653)	547.7% (77.1 to 1489.9%)
Gabon	11 (8 to 21)	40 (25 to 64)	253.9% (69.4 to 528.7%)	9 (6 to 16)	28 (18 to 44)	216.0% (54.5 to 441.6%)	266 (177 to 494)	877 (550 to 1414)	229.9% (55.7 to 492.2%)

Data in parentheses are 95% uncertainty intervals. *DALYs*   disability-adjusted life-years, *SDI * Sociodemographic index

The disease burden of ovarian cancer varies significantly across SDI regions. In 2019, the number of ovarian cancer incident cases was highest in the high SDI region, corresponding to value of 80454 (70504 to 91461), and lowest in the low SDI region. All regions showed an increasing trend during 1990–2019, while the high SDI region was the region with the lowest increase in morbidity burden, but the low-middle SDI region was the highest one, increased by 326.0% (158.8 to 470.5%) (Table 1) for new cases.

**Fig. 3:**
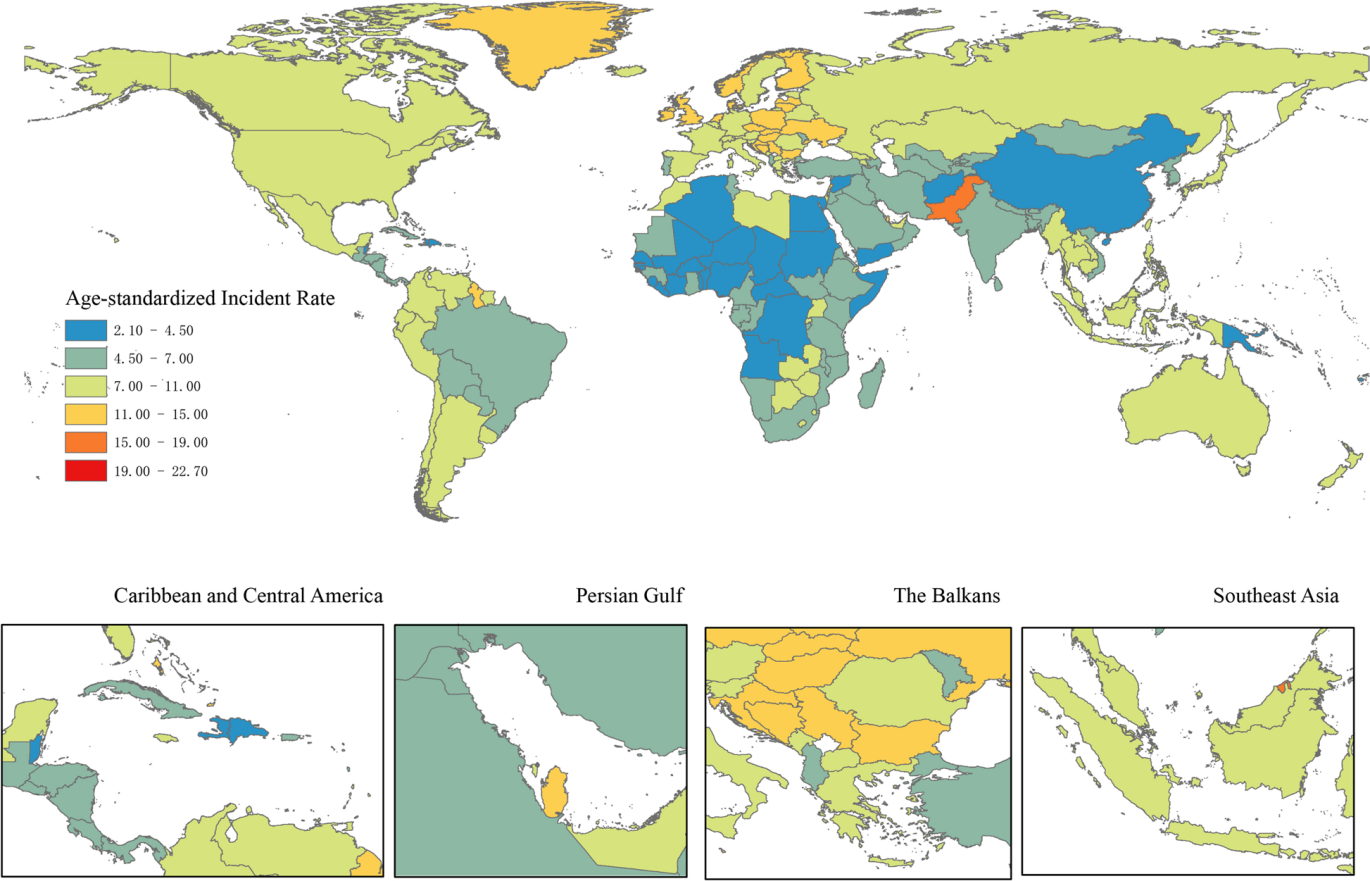
Map of age-standardized incidence rate due to ovarian cancer in 2019

Similarly, the high SDI quintile had a negative change of ASIR during this period, accounting for 18.8% (-28.6 to -3.8%). In contrast, the disease burden in low-middle SDI quintile increased substantially (91.3% (17.8 to 157.1%) for ASIR) (Table 2, Fig. 3).

**Table 2:** Age-standardized rates of incidence, death and DALY per 100,000 population in 1990 and 2019, percentage change for ovarian cancer during 1990–2019, by global, SDI regions, world regions and countries

	ASIR (95% Uncertainty interval)			ASDR (95% Uncertainty interval)			ASDALYR (95% Uncertainty interval)		
	1990 ASIR per 100,000	2019 ASIR per 100,000	Percentage change in age-standardized rates,1990–2019	1990 ASDR per 100,000	2019 ASDR per 100,000	Percentage change in age-standardized rates,1990–2019	1990 ASDALY per 100,000	2019 ASDALY per 100,000	Percentage change in age-standardized rates,1990–2019
**Global**	6.5 (6.0 to 7.3)	6.9 (6.1 to 7.7)	6.3% (-10.1 to 20.4%)	4.6 (4.2 to 5.2)	4.6 (4.0 to 5.0)	-0.5% (-14.0 to 10.5%)	124.1 (113.7 to 143.0)	124.7 (109.1 to 138.7)	0.5% (-15.4 to 12.9%)
**SDI regions**									
High SDI	11.5 (10.4 to 11.8)	9.3 (8.2 to 10.6)	-18.8% (-28.6 to -3.8%)	7.5 (6.7 to 7.7)	5.7 (5.2 to 6.1)	-24.0% (-30.3 to -12.3%)	198.3 (178.8 to 204.3)	143.8 (132.6 to 154.5)	-27.5% (-33.1 to -14.2%)
High-middle SDI	7.3 (6.7 to 7.8)	7.6 (6.4 to 8.5)	3.4% (-9.8 to 16.4%)	5.0 (4.6 to 5.3)	3.7 (3.0 to 4.3)	-4.5% (-16.4 to 5.7%)	145.1 (132.9 to 155.3)	133.0 (114.8 to 147.5)	-8.3% (-20.1 to 1.9%)
Middle SDI	3.4 (3.0 to 4.3)	5.7 (4.7 to 6.6)	67.0% (18.7 to 100.6%)	2.4 (2.2 to 3.0)	4.7 (4.1 to 5.2)	49.9% (9.2 to 79.4%)	73.9 (64.7 to 93.5)	106.4 (87.7 to 124.0)	44.0% (3.5% to 73.5%)
Low-middle SDI	3.0 (2.3 to 4.7)	5.6 (4.6 to 7.1)	91.3% (17.8 to 157.1%)	2.3 (1.8 to 3.6)	4.1 (3.4 to 5.1)	75.1% (10.1 to 134.4%)	67.6 (53.3 to 107.7)	118.4 (95.4 to 150.2)	75.1% (6.9 to 134.6%)
Low SDI	3.0 (2.0 to 5.9)	5.1 (4.3 to 6.3)	73.9% (1.8 to 149.2%)	2.4 (1.7 to 4.7)	4.0 (3.4 to 4.9)	63.8% (-2.1 to 134%)	71.0 (48.9 to 144.9)	115.2 (96.4 to 141.8)	62.1% (-6.4 to 134.4%)
**High-income North America**	6.1 (5.8 to 6.4)	6.8 (5.5 to 8.2)	11.7% (-10.2 to 33.0%)	3.7 (3.5 to 4.0)	3.5 (3.0 to 3.7)	-7.5% (-19.9 to -0.7%)	112.9 (108.8 to 119.1)	101.2 (89.5 to 107.9)	-10.4% (-21.6 to -4.3%)
Canada	12.3 (11.3 to 13.0)	9.4 (7.0 to 12.5)	-23.6% (-43.6 to 6.1%)	8.1 (7.5 to 8.5)	5.9 (4.9 to 6.9)	-27.2% (-38.8 to -12.3%)	214.3 (200.3 to 225.9)	144.5 (122.3 to 170.4)	-32.6% (-43.4 to -18.5%)
Greenland	16.6 (13.4 to 22.0)	13.6 (10.1 to 17.6)	-18.1% (-45.8 to 12.3%)	12.7 (10.1 to 18.0)	9.5 (7.1 to 12.8)	-24.9% (-49.9 to 3.0%)	350.6 (281.0 to 450.3)	258.9 (190.6 to 337.1)	-26.2% (-50.6 to 2.6%)
United States of America	12.7 (11.7 to 13.2)	9.9 (7.9 to 12.2)	-22.2% (-38.1 to -0.7%)	8.1 (7.4 to 8.4)	6.5 (5.9 to 7.0)	-20.1% (-26.1 to -3.1%)	213.1 (195.9 to 220.5)	157.7 (147.8 to 171.5)	-26.0% (-31.5 to -9.3%)
**Australasia**	10.9 (9.5 to 11.5)	8.2 (6.5 to 10.6)	-24.4% (-40.8 to 2.6%)	7.2 (6.3 to 7.6)	5.3 (4.6 to 6.1)	-26.5% (-36.9 to -1.3%)	191.7 (166.0 to 201.8)	128.1 (111.5 to 149.4)	-33.2% (-42.9 to -7.7%)
Australia	10.6 (9.2 to 11.2)	8.1 (6.1 to 10.8)	-24.0% (-43.4 to 6.4%)	7.0 (6.1 to 7.4)	5.2 (4.4 to 6.0)	-26.4% (-37.5 to 0.0%)	187.1 (162.3 to 197.4)	124.3 (107.0 to 146.6)	-33.6% (-43.6 to -6.9%)
New Zealand	12.4 (11.0 to 13.5)	9.2 (7.0 to 12.2)	-26.1% (-44.8 to -0.7%)	8.0 (7.1 to 8.6)	5.9 (5.1 to 6.6)	-26.6% (-38.0 to -8.0%)	214.7 (188.5 to 232.0)	148.0 (129.4 to 167.8)	-31.1% (-41.4 to -12.5%)
**High-income Asia Pacific**	6.1 (5.8 to 6.4)	6.8 (5.5 to 8.2)	11.7% (-10.2 to 33.0%)	3.7 (3.5 to 4.0)	3.5 (3.0 to 3.7)	-7.5% (-19.9 to -0.7%)	112.9 (108.8 to 119.1)	101.2 (89.5 to 107.9)	-10.4% (-21.6 to -4.3%)
Brunei Darussalam	10.9 (8.3 to 16.0)	16.1 (12.7 to 19.4)	47.4% (-4.9 to 103.6%)	7.9 (6.0 to 11.5)	10.1 (8.0 to 11.9)	28.2% (-18.3 to 77.1%)	227.1 (169.5 to 342.4)	282.0 (224.6 to 338.6)	24.2% (-22.6 to 74.0%)
Japan	6.9 (6.5 to 7.1)	7.4 (5.8 to 9.3)	8.3% (-14.9 to 36.9%)	4.1 (3.9 to 4.2)	3.7 (3.3 to 4.0)	-9.9% (-18.7 to -2.9%)	124.9 (119.0 to 128.8)	109.9 (99.4 to 118.3)	-12.0% (-21.2 to -2.7%)
Singapore	7.7 (7.0 to 8.5)	7.4 (5.8 to 9.4)	-4.0% (-26.6 to 24.4%)	5.1 (4.7 to 5.6)	4.1 (3.5 to 4.6)	-21.0% (-32.1 to -9.0%)	149.7 (136.9 to 162.8)	113.1 (97.2 to 128.0)	-24.5% (-35.8 to -11.9%)
Republic of Korea	2.8 (2.4 to 4.8)	5.5 (3.3 to 6.9)	95.3% (-37.8 to 170.1%)	1.9 (1.6 to 3.4)	2.9 (1.8 to 3.4)	54.9% (-55.1 to 104.0%)	57.9 (50.1 to 98.1)	81.4 (49.3 to 94.6)	40.6% (-57.1 to 81.6%)
**Western Europe**	12.9 (11.4 to 13.3)	10.4 (9.0 to 12.1)	-19.6% (-31.3 to -2.0%)	8.2 (7.3 to 8.5)	6.3 (5.7 to 6.8)	-23.5% (-30.2 to -10.9%)	218.6 (192.3 to 225.7)	154.8 (142.2 to 167.8)	-29.2% (-35.7 to -13.3%)
Andorra	7.4 (5.2 to 11.3)	7.5 (5.5 to 10.1)	1.8% (-38 to 53.7%)	4.8 (3.3 to 7.4)	4.5 (3.2 to 6.1)	-5.5% (-43.5 to 0.5%)	123.6 (86.6 to 189.9)	116.1 (83.5 to 157.7)	-6.1% (-42.7 to 44.4%)
Austria	15.0 (11.7 to 16.0)	9.6 (7.5 to 13.8)	-36.0% (-50.8 to 18.8%)	9.6 (7.4 to 10.2)	6.0 (5.2 to 8.1)	-5.5% (-43.5 to 45.5%)	252.1 (194.3 to 267.8)	146.0 (126.9 to 214.9)	-42.1% (-50.0 to 11.0%)
Belgium	14.8 (12.4 to 15.9)	9.6 (7.4 to 13.1)	-34.9% (-51.5 to -4.3%)	10.1 (8.4 to 10.8)	6.3 (5.4 to 7.3)	-37.9% (-46.4 to 10.8%)	256.7 (218.5 to 276.0)	150.4 (128.7 to 183.6)	-41.4% (-51.7 to -13.5%)
Cyprus	8.8 (6.8 to 12.7)	9.8 (7.0 to 12.3)	11.4% (-42.3 to 65.1%)	6.5 (5.0 to 9.5)	6.4 (4.6 to 7.9)	-37.0% (-47.7 to -12.4%)	163.9 (127.3 to 238.5)	155.7 (113.9 to 191.9)	-5.0% (-48.6 to 38.2%)
Denmark	12.5 (11.7 to 13.3)	11.8 (9.1 to 15.3)	-5.9% (-27.8 to 24.7%)	9.3 (8.7 to 9.8)	8.1 (7.2 to 9.4)	-0.9% (-48.8 to 47.8%)	250.8 (232.4 to 266.5)	195.2 (173.7 to 225.2)	-22.2% (-31.8 to -8.6%)
Finland	12.7 (11.6 to 13.5)	11.5 (8.9 to 14.8)	-9.3% (-30.2 to 21.6%)	7.9 (7.3 to 8.4)	6.7 (5.9 to 7.6)	-12.4% (-23.1 to 2.0%)	208.5 (191.8 to 220.3)	163.0 (142.1 to 186.4)	-21.8% (-32.5 to -1.8%)
France	11.9 (10.2 to 12.6)	9.5 (7.2 to 12.5)	-20.6% (-39.2 to 7.6%)	8.0 (7.0 to 8.4)	6.1 (5.2 to 6.9)	-15.1% (-26.0 to 0.9%)	209.2 (180.1 to 220.3)	146.7 (128.6 to 169.1)	-29.9% (-39.2 to -6.1%)
Germany	14.2 (11.7 to 15.0)	10.7 (8.1 to 14.0)	-25.0% (-43.5 to 2.2%)	8.9 (7.3 to 9.4)	6.4 (5.7 to 7.2)	-24.1% (-34.5 to -6.0%)	233.7 (191.7 to 245.8)	156.7 (140.1 to 174.8)	-33.0% (-41.4 to -14.7%)
Greece	9.7 (9.1 to 10.5)	10.2 (7.8 to 13.1)	4.8% (-20.5 to 34.3%)	6.2 (5.8 to 6.6)	6.2 (5.5 to 6.8)	-27.8% (-36.4 to -12.7%)	166.0 (156.3 to 178.4)	158.1 (142.7 to 176.3)	-4.7% (-15.2 to 7.2%)
Iceland	14.4 (12.8 to 16.1)	9.4 (7.8 to 12.0)	-34.5% (-46.7 to -9.4%)	9.1 (8.2 to 10.1)	5.4 (4.6 to 6.9)	0.0% (-9.8 to 12.3%)	240.2 (213.9 to 266.1)	138.3 (118.1 to 179.6)	-42.4% (-51.9 to -20.4%)
Ireland	15.7 (14.3 to 17.2)	12.8 (9.4 to 17.3)	-18.4% (-42.3 to 15.8%)	10.4 (9.6 to 11.2)	8.0 (6.1 to 9.7)	-40.5% (-50.4 to -17.8%)	282.1 (258.9 to 305.6)	197.9 (154.2 to 239.7)	-29.8% (-47.9 to -11.1%)
Israel	11.5 (9.8 to 12.4)	9.5 (7.1 to 12.3)	-18.0% (-38.8 to 10.5%)	8.2 (6.9 to 8.8)	6.1 (5.4 to 6.9)	-23.0% (-43.7 to -3.0%)	214.6 (179.8 to 230.2)	153.7 (135.5 to 172.6)	-28.3% (-37.4 to -10.4%)
Italy	10.8 (10.3 to 11.3)	9.7 (7.5 to 12.3)	-10.6% (-30.7 to 15.7%)	6.7 (6.4 to 6.9)	5.7 (5.1 to 6.2)	-25.0% (-33.9 to -8.8%)	182.2 (174.1 to 188.2)	145.9 (133.6 to 157.3)	-19.9% (-26.3 to -11.6%)
Luxembourg	16.1 (14.7 to 17.6)	11.4 (9.0 to 15.1)	-29.0% (-44.9 to -4.7%)	10.9 (9.9 to 11.9)	7.5 (6.0 to 9.4)	-14.5% (-22.1 to -7.6%)	280.2 (256.5 to 306.1)	182.6 (148.3 to 234.1)	-34.8% (-47.4 to -15.0%)
Malta	12.8 (11.2 to 14.3)	11.4 (9.4 to 14.2)	-10.9% (-29.3 to 15.1%)	8.9 (7.6 to 9.8)	6.9 (5.7 to 8.4)	-30.8% (-44.6 to -10.2%)	230.0 (201.3 to 253.9)	176.1 (147.0 to 211.5)	-23.5% (-37.6 to -1.6%)
Monaco	20.9 (15.7 to 28.2)	22.7 (16.7 to 28.9)	8.6% (-27.6 to 56.6%)	13.2 (9.6 to 17.9)	13.7 (9.8 to 17.2)	3.6% (-30.3 to 49.1%)	342.2 (252.9 to 464.8)	342.1 (248.9 to 436.0)	0.0% (-33.9 to 46.2%)
Netherlands	14.8 (13.0 to 15.7)	11.6 (8.9 to 14.8)	-21.9% (-39.4 to 0.0%)	9.7 (8.4 to 10.2)	7.4 (6.5 to 8.2)	-23.5% (-32.0 to -14.5%)	255.0 (223.2 to 268.6)	173.5 (154.6 to 190.6)	-32.0% (-39.1 to -22.2%)
Norway	14.6 (13.7 to 15.4)	12.3 (9.7 to 15.5)	-15.7% (-33.5 to 7.8%)	9.5 (8.9 to 9.9)	7.8 (7.1 to 8.7)	-18.0% (-26.1 to -2.5%)	259.2 (243.8 to 269.6)	194.3 (176.0 to 216.6)	-25.0% (-32.8 to -9.2%)
Portugal	6.8 (6.3 to 7.3)	5.8 (4.4 to 7.8)	-14.2% (-36.9 to 16.3%)	4.6 (4.4 to 5.0)	3.9 (3.4 to 4.8)	-16.4% (-28.3 to -1.3%)	131.3 (123.2 to 140.0)	102.2 (89.4 to 123.9)	-22.2% (-34.1 to -3.7%)
San Marino	6.3 (5.0 to 8.3)	7.2 (5.2 to 10.0)	14.5% (-21.3 to 67.2%)	4.1 (3.3 to 5.5)	4.4 (2.8 to 6.7)	5.6% (-34.5 to 69.6%)	102.2 (79.3 to 138.8)	112.9 (67.7 to 177.4)	10.4% (-33.8 to 83.2%)
Spain	10.9 (9.3 to 11.6)	9.3 (7.0 to 12.0)	-14.3% (-35.1 to 11.7%)	5.5 (4.8 to 5.8)	4.7 (4.0 to 5.3)	-13.6% (-23.5 to -3.3%)	156.8 (130.9 to 166.0)	125.1 (109.2 to 140.1)	-20.2% (-29.4 to -8.6%)
Sweden	14.5 (11.7 to 15.5)	9.5 (7.6 to 12.3)	-34.3% (-48.3 to -6%)	9.4 (7.6 to 10.0)	6.4 (5.7 to 7.9)	-31.9% (-39.2 to 6.7%)	250.4 (199.4 to 265.3)	157.2 (142.2 to 195.3)	-37.2% (-43.8 to 0.7%)
Switzerland	8.0 (7.4 to 8.6)	8.7 (6.6 to 11.1)	8.6% (-19.4 to 42.2%)	4.9 (4.6 to 5.3)	5.4 (4.5 to 6.1)	9.5% (-9.4 to 24.9%)	127.8 (119.1 to 137.3)	128.3 (112.0 to 144.0)	0.4% (-15.1 to 14.7%)
United Kingdom	16.2 (14.5 to 16.8)	13.2 (10.4 to 16.9)	-18.4% (-37 to 7.9%)	10.6 (9.7 to 11.0)	8.0 (7.3 to 8.7)	-24.8% (-31.0 to -9.8%)	287.0 (252.8 to 296.7)	191.4 (178.2 to 211.8)	-33.3% (-38.8 to -16.5%)
**Southern Latin America**	7.8 (6.9 to 9.2)	8.5 (6.6 to 10.9)	9.4% (-18.8 to 48.9%)	5.8 (5.1 to 6.8)	5.6 (5.2 to 6.4)	-2.3% (-21.0 to 19.6%)	159.6 (140.7 to 187.6)	154.9 (142.6 to 173.7)	-2.9% (-21.2 to 18.5%)
Argentina	8.1 (7.0 to 9.8)	9.0 (6.9 to 11.5)	11.2% (-18.5 to 51.8%)	6.0 (5.2 to 7.2)	6.0 (5.4 to 6.8)	-0.3% (-20.2 to 24.0%)	166.3 (143.3 to 199.5)	165.4 (150.1 to 187.1)	-0.6% (-20.6 to 24.7%)
Chile	6.9 (6.1 to 7.7)	7.3 (5.6 to 9.5)	6.5% (-21.8 to 45.0%)	5.0 (4.4 to 5.6)	4.7 (4.2 to 5.3)	-6.5% (-23.0 to 19.2%)	138.4 (123.7 to 155.1)	127.5 (112.3 to 146.5)	-7.9% (-25.5 to 18.1%)
Uruguay	8.1 (7.1 to 9.2)	9.6 (7.5 to 12.3)	18.9% (-12.0 to 57.9%)	5.8 (5.2 to 6.6)	6.4 (5.7 to 7.1)	10.7% (-8.1 to 29.8%)	161.4 (142.0 to 183.5)	175.1 (156.0 to 194.6)	8.5% (-10.6 to 29.3%)
**Eastern Europe**	9.9 (8.4 to 10.5)	10.8 (9.0 to 13.1)	9.4% (-7.4 to 37.4%)	6.5 (5.8 to 6.9)	6.7 (5.6 to 8.0)	3.2% (-12.4 to 26.4%)	203.0 (170.9 to 215.7)	202.7 (167.4 to 242.9)	-0.1% (-15.7 to 25.0%)
Belarus	9.2 (8.0 to 9.9)	8.6 (5.9 to 11.6)	-6.2% (-34.2 to 30.7%)	6.9 (5.8 to 7.4)	5.7 (4.0 to 7.7)	-16.6% (-41.5 to 16.8%)	201.2 (172.8 to 219.5)	171.5 (118.5 to 231.5)	-14.8% (-40.3 to 19.4%)
Estonia	13.4 (11.9 to 14.6)	10.9 (8.0 to 15.6)	-18.9% (-40.8 to 28.9%)	9.6 (8.0 to 10.4)	7.4 (5.4 to 9.8)	-23.5% (-43.5 to 18.8%)	269.3 (240.4 to 293.2)	191.4 (139.6 to 276.3)	-28.9% (-48.0 to 15.0%)
Latvia	11.9 (10.9 to 12.9)	12.0 (8.6 to 16.2)	0.7% (-28.0 to 37.0%)	9.3 (8.5 to 10.1)	9.2 (6.7 to 12.4)	-1.3% (-28.1 to 31.9%)	277.1 (250.5 to 299.4)	256.6 (181.1 to 347.8)	-7.4% (-34.2 to 26.9%)
Lithuania	13.4 (12.2 to 14.4)	12.5 (9.8 to 15.9)	-6.5% (-27.8 to 20.2%)	10.2 (9.2 to 10.9)	9.1 (7.2 to 11.3)	-10.6% (-29.7 to 13.8%)	293.4 (268.5 to 315.0)	255.9 (199.4 to 324.9)	-12.8% (-32.8 to 12.3%)
Republic of Moldova	7.5 (5.1 to 8.3)	6.6 (5.0 to 8.0)	-12.7% (-32.8 to 27.2%)	5.3 (4.0 to 5.8)	4.5 (3.4 to 5.5)	-15.5% (-34.2 to 18.0%)	173.2 (115.1 to 190.3)	139.4 (105.7 to 170.5)	-19.5% (-38.0 to 18.1%)
Russian Federation	10.3 (8.4 to 10.8)	10.9 (8.6 to 14.0)	6.2% (-15.5 to 45.8%)	6.8 (5.9 to 7.1)	6.9 (5.4 to 8.7)	1.7% (-18.3 to 33.2%)	210.6 (172.3 to 221.4)	204.2 (157.0 to 258.2)	-3.0% (-23.0 to 33.2%)
Ukraine	8.7 (7.2 to 9.9)	11.1 (8.4 to 14.4)	27.9% (-7.3 to 69.5%)	5.4 (4.6 to 6.2)	6.3 (4.9 to 7.9)	16.5% (-13.6 to 54.3%)	173.8 (143.2 to 199.2)	203.8 (156.9 to 259.0)	17.3% (-14.0 to 56.6%)
**Central Europe**	11.2 (10.6 to 11.6)	11.7 (10.0 to 13.7)	4.7% (-10.1 to 22.0%)	7.5 (7.2 to 7.8)	7.6 (6.6 to 8.9)	1.6% (-11.7 to 17.9%)	223.7 (210.6 to 231.1)	212.3 (181.7 to 247.2)	-5.1% (-18.0 to 11.4%)
Albania	3.2 (2.8 to 3.8)	5.0 (3.4 to 6.9)	53.9% (5.3 to 120.2%)	2.1 (1.8 to 2.7)	2.8 (2.0 to 3.9)	37.2% (-6.6 to 92.5%)	64.2 (56.5 to 77.5)	85.5 (58.6 to 118.8)	33.3% (-9.0 to 91.2%)
Bosnia and Herzegovina	7.2 (6.3 to 9.1)	11.4 (8.5 to 15.2)	60.1% (13.6 to 110.9%)	4.9 (4.3 to 6.5)	7.4 (5.6 to 9.8)	52.0% (6.6 to 100.1%)	144.8 (127.3 to 183.6)	210.1 (154.8 to 283.0)	45.1% (2.8 to 92.1%)
Bulgaria	9.1 (8.4 to 10.0)	12.3 (8.7 to 16.0)	35.3% (-6.4 to 80.3%)	5.7 (5.2 to 6.3)	7.6 (5.5 to 9.7)	33.5% (-8.0 to 75.9%)	179.6 (164.9 to 198.1)	230.8 (162.9 to 301.7)	28.5% (-11.3 to 71.7%)
Croatia	14.7 (12.9 to 16.4)	12.8 (9.5 to 16.9)	-13.2% (-35.6 to 19.5%)	8.3 (7.2 to 9.1)	6.8 (5.1 to 8.8)	-17.9% (-38.4 to 10.7%)	233.7 (204.0 to 259.1)	183.0 (135.9 to 240.5)	-21.7% (-42.4 to 7.3%)
Czechia	14.3 (12.4 to 15.0)	12.4 (9.8 to 15.7)	-13.3% (-31.9 to 15.3%)	9.1 (7.7 to 9.6)	7.4 (5.9 to 9.3)	-18.7% (-36.2 to 4.9%)	259.2 (223.3 to 273.1)	195.2 (154.0 to 249.8)	-24.7% (-41.2 to 0.7%)
Hungary	12.2 (11.5 to 13.0)	11.4 (9.2 to 14.3)	-6.6% (-25.4 to 17.8%)	8.2 (7.7 to 8.7)	7.4 (6.0 to 9.0)	-9.7% (-26.9 to 11.9%)	230.3 (217.7 to 244.7)	200.7 (161.3 to 254.6)	-12.8% (-30.9 to 10.2%)
Montenegro	8.1 (6.5 to 10.7)	9.4 (6.8 to 12.5)	16.0% (-17.1 to 48.6%)	5.0 (3.9 to 6.7)	5.7 (4.2 to 7.8)	14.5% (-17.9 to 46.6%)	148.5 (116.4 to 195.5)	166.1 (120.3 to 221.7)	11.9% (-19.3 to 44.0%)
North Macedonia	8.1 (6.9 to 9.5)	10.8 (7.8 to 14.3)	33.1% (-5.8 to 77.7%)	5.4 (4.7 to 6.7)	6.9 (5.1 to 8.9)	27.4% (-9.7 to 68.9%)	164.7 (140.2 to 193.7)	198.1 (143.4 to 261.0)	20.2% (-14.8 to 58.8%)
Poland	12.7 (12.0 to 13.2)	12.8 (9.7 to 16.4)	0.6% (-23.7 to 30.2%)	9.1 (8.7 to 9.4)	9.1 (7.0 to 11.5)	-0.8% (-23.5 to 26.4%)	274.7 (254.5 to 283.0)	246.5 (189.9 to 317.6)	-10.3% (-31.8 to 15.5%)
Romania	9.3 (8.5 to 9.9)	10.3 (8.2 to 12.8)	11.1% (-11.8 to 36.5%)	5.8 (5.5 to 6.1)	6.4 (5.2 to 7.8)	10.8% (-11.6 to 36.1%)	189.1 (168.2 to 199.8)	188.3 (149.6 to 231.6)	-0.4% (-21.1 to 22.7%)
Serbia	8.8 (7.3 to 10.8)	11.8 (8.7 to 15.3)	35.1% (-8.9 to 83.0%)	6.2 (5.2 to 7.7)	7.9 (5.9 to 10.1)	27.7% (-13.6 to 71.6%)	183.3 (152.9 to 225.1)	223.7 (164.2 to 289.8)	22.1% (-17.7 to 67.5%)
Slovakia	11.2 (8.5 to 12.5)	11.8 (8.1 to 16.0)	5.5% (-29.4 to 45.0%)	6.9 (5.2 to 7.8)	6.5 (4.4 to 8.8)	-5.5% (-36.6 to 28.2%)	202.0 (150.7 to 227.2)	182.1 (124.4 to 249.3)	-9.8% (-40.1 to 23.7%)
Slovenia	12.6 (9.3 to 16.6)	9.6 (7.0 to 13.1)	-23.8% (-51.3 to 16.8%)	9.3 (7.0 to 12.1)	6.7 (5.0 to 9.0)	-27.4% (-52.7 to 8.4%)	257.5 (189.9 to 342.5)	178.9 (129.3 to 244.4)	-30.5% (-56.0 to 6.1%)
**Central Asia**	5.0 (4.3 to 5.6)	6.9 (6.0 to 7.8)	37.8% (17.0 to 65.1%)	3.4 (2.9 to 3.8)	4.7 (4.0 to 5.2)	37.0% (16.2 to 63.5%)	106.4 (90.4 to 117.9)	138.4 (119.9 to 155.6)	30.1% (9.7 to 56.0%)
Armenia	6.5 (5.1 to 9.2)	7.7 (6.1 to 9.4)	19.0% (-23.4 to 0.7%)	4.6 (3.6 to 6.2)	5.2 (4.2 to 6.4)	14.8% (-24.2 to 58.5%)	133.3 (104.6 to 189.7)	147.4 (116.5 to 181.6)	10.6% (-30.1 to 54.6%)
Azerbaijan	3.4 (2.4 to 4.6)	5.6 (3.7 to 7.6)	19.0% (-23.4 to 65.7%)	2.3 (1.6 to 3.0)	3.5 (2.3 to 4.8)	56.2% (5.1 to 127.7%)	74.6 (53.2 to 98.6)	112.7 (73.4 to 153.4)	51.1% (-2.7 to 116.3%)
Georgia	4.5 (3.8 to 5.7)	9.7 (6.7 to 12.0)	63.1% (5.3 to 132.7%)	2.9 (2.5 to 3.6)	6.5 (4.3 to 8.0)	124.9% (25.1 to 198.0%)	91.3 (76.9 to 115.2)	194.5 (133.1 to 240.5)	113.1% (22.3 to 183.9%)
Kazakhstan	7.9 (6.2 to 9.1)	10.0 (8.1 to 12.0)	113.9% (23.9 to 185.5%)	5.3 (4.2 to 6.1)	6.4 (5.3 to 7.6)	21.5% (-1.4 to 52.3%)	165.8 (128.7 to 191.2)	194.9 (157.8 to 233.2)	17.5% (-5.3 to 46.8%)
Kyrgyzstan	5.5 (4.8 to 6.1)	7.0 (5.5 to 8.4)	26.6% (2.3 to 58.5%)	3.9 (3.4 to 4.3)	4.7 (3.7 to 5.7)	22.4% (-3.7 to 53.8%)	118.2 (103.5 to 132.1)	143.3 (111.3 to 172.0)	21.2% (-4.9 to 53.4%)
Mongolia	3.8 (2.5 to 6.9)	6.1 (4.4 to 8.7)	28.2% (0.3 to 60.0%)	2.8 (1.9 to 5.0)	4.4 (3.2 to 5.9)	53.7% (-10.5 to 147.7%)	88.6 (58.9 to 162.3)	130.1 (92.6 to 185.7)	46.9% (-12.2 to 133.8%)
Tajikistan	4.0 (2.7 to 5.4)	5.5 (4.2 to 6.9)	60.7% (-4.1 to 154.4%)	3.1 (1.9 to 4.2)	4.2 (3.1 to 5.4)	37.2% (-5.4 to 112.5%)	86.2 (58.8 to 119.0)	115.8 (89.7 to 145.7)	34.3% (-8.6 to 103.1%)
Turkmenistan	4.7 (3.8 to 5.3)	5.4 (3.9 to 7.1)	37.3% (-5.5 to 108.0%)	3.0 (2.5 to 3.4)	3.1 (2.2 to 4.1)	5.0% (-24.5 to 41.3%)	102.4 (82.0 to 114.6)	108.6 (76.4 to 140.9)	6.1% (-25.0 to 45.6%)
Uzbekistan	2.7 (1.4 to 3.4)	5.4 (4.2 to 6.5)	14.5% (-18.7 to 56.8%)	1.9 (0.9 to 2.4)	3.7 (2.9 to 4.5)	99.2% (36.6 to 254.9%)	58.3 (30.5 to 73.5)	106.9 (82.8 to 130.0)	83.4% (28.1 to 207.1%)
**Central Latin America**	5.0 (4.8 to 5.3)	7.5 (6.2 to 9.0)	49.6% (24.4 to 79.2%)	3.6 (3.5 to 3.9)	4.8 (3.9 to 5.7)	31.1% (8.4 to 54.5%)	105.3 (101.7 to 112.1)	139.4 (115.4 to 167.1)	32.4% (8.5 to 57.5%)
Colombia	6.9 (6.5 to 7.3)	7.6 (5.8 to 10.4)	10.5% (-16.4 to 50.4%)	5.0 (4.7 to 5.4)	4.7 (3.6 to 6.4)	-7.8% (-29.4 to 24.1%)	146.0 (137.5 to 155.0)	136.9 (104.7 to 188.4)	-6.3% (-29.1 to 27.8%)
Costa Rica	3.5 (3.2 to 3.8)	6.2 (4.7 to 8.1)	78.2% (27.1 to 137.1%)	2.4 (2.2 to 2.6)	4.0 (3.0 to 5.2)	64.9% (17.6 to 116.2%)	68.5 (62.3 to 75.6)	107.9 (79.4 to 140.8)	57.4% (14.0 to 108.9%)
El Salvador	2.3 (2.0 to 3.5)	6.2 (3.9 to 8.6)	167.9% (29.3 to 299.6%)	1.7 (1.4 to 2.6)	3.9 (2.6 to 5.3)	132.5% (11.2 to 241.4%)	51.4 (43.5 to 77.6)	117.4 (73.8 to 162.4)	128.3% (11.3 to 239.8%)
Guatemala	1.1 (0.9 to 1.5)	4.7 (2.8 to 6.2)	322.3% (79.1 to 491.3%)	0.8 (0.7 to 1.1)	3.3 (2.0 to 4.2)	297.4% (65.3 to 449.9%)	27.2 (22.6 to 33.5)	96.4 (57.5 to 126.0)	255.0% (69.5 to 395.6%)
Honduras	3.1 (2.3 to 4.6)	5.6 (2.7 to 11.9)	81.4% (-1.3 to 230.0%)	2.4 (1.8 to 3.5)	4.2 (2.1 to 9.0)	73.0% (-2.1 to 233.4%)	70.5 (52.9 to 104.7)	115.5 (55.6 to 245.0)	63.8% (-11.8 to 196.8%)
Mexico	5.8 (5.6 to 6.0)	8.1 (6.5 to 10.0)	40.6% (10.6 to 73.7%)	4.2 (4.0 to 4.4)	5.2 (4.2 to 6.4)	23.4% (-1.7 to 50.6%)	121.1 (117.2 to 127.1)	153.2 (123.0 to 189.7)	26.5% (-0.4 to 55.0%)
Nicaragua	2.4 (1.6 to 3.1)	5.5 (3.6 to 6.9)	130.9% (30.6 to 254.3%)	1.7 (1.1 to 2.3)	3.7 (2.4 to 4.6)	115.6% (19.7 to 259.8%)	51.2 (35.6 to 67.9)	101.8 (69.0 to 129.6)	98.9% (12.1 to 203.7%)
Panama	3.4 (3.0 to 3.8)	6.2 (4.6 to 8.1)	82.2% (29.6 to 144.7%)	2.4 (2.1 to 2.7)	3.9 (2.9 to 5.1)	62.5% (16.3 to 115.8%)	69.1 (62.1 to 77.7)	110.4 (81.3 to 144.8)	59.7% (13.7 to 116.3%)
Venezuela (Bolivarian Republic of)	1.7 (1.4 to 3.0)	7.3 (4.2 to 9.8)	339.0% (33.0 to 535.6%)	1.2 (1.0 to 2.2)	4.5 (2.6 to 6.1)	290.1% (15.3 to 460.5%)	35.5 (30.7 to 63.5)	133.5 (76.9 to 181.6)	276.5% (15.3 to 449.0%)
**Andean Latin America**	3.8 (3.0 to 5.5)	7.1 (5.0 to 9.0)	88.0% (15.5 to 167.5%)	2.8 (2.3 to 4.1)	4.6 (3.3 to 5.9)	63.0% (0.9 to 127.9%)	83.9 (67.2 to 123.0)	134.2 (95.9 to 171.7)	59.9% (-1.4 to 127.1%)
Bolivia (Plurinational State of)	3.2 (2.0 to 7.8)	6.3 (4.3 to 10.2)	93.3% (12.5 to 206.6%)	2.6 (1.6 to 6.3)	4.6 (3.2 to 7.7)	73.7% (0.7 to 167.0%)	76.5 (47.3 to 184.3)	130.1 (87.5 to 210.0)	70.1% (-1.2 to 169.2%)
Ecuador	2.2 (1.7 to 3.8)	7.3 (4.2 to 9.8)	234.0% (26.2 to 425.7%)	1.6 (1.3 to 2.9)	4.8 (2.8 to 6.4)	197.0% (9.3 to 364.1%)	49.1 (38.9 to 84.8)	137.0 (80.9 to 186.2)	179.1% (8.1 to 336.6%)
Peru	4.6 (3.5 to 6.2)	7.2 (4.9 to 9.8)	57.0% (-5.6 to 104.6%)	3.4 (2.6 to 4.6)	4.6 (3.1 to 6.2)	33.2% (-18.3 to 98.3%)	101.6 (76.7 to 138.3)	134.3 (92.2 to 183.2)	32.2% (-21.7 to 101.5%)
**Caribbean**	2.0 (1.7 to 3.0)	5.7 (4.1 to 7.7)	188.8% (37.6 to 264.5%)	1.4 (1.2 to 2.1)	3.8 (2.7 to 5.1)	174.9% (27.8 to 244.8%)	41.6 (36.0 to 66.5)	109.4 (78.9 to 153.6)	163.3% (28.0 to 232.7%)
Antigua and Barbuda	2.0 (1.7 to 3.8)	9.5 (5.7 to 11.5)	366.6% (43.0 to 519.4%)	1.4 (1.2 to 2.6)	6.7 (3.8 to 8.1)	379.9% (42.5 to 535.3%)	41.0 (34.4 to 76.4)	176.2 (106.8 to 214.4)	330.3% (32.8 to 474.9%)
Bahamas	3.6 (3.1 to 5.8)	11.5 (7.8 to 14.6)	219.1% (32.4 to 326.7%)	2.5 (2.1 to 4.1)	7.3 (5.2 to 9.2)	198.0% (25.2 to 294.3%)	75.0 (65.0 to 120.3)	218.3 (149.5 to 280.6)	191.0% (22.1 to 288.7%)
Barbados	2.0 (1.8 to 3.7)	10.0 (4.8 to 12.3)	386.3% (29.4 to 542.9%)	1.4 (1.2 to 2.5)	6.6 (3.2 to 8.1)	366.2% (24.9 to 504.4%)	40.8 (35.6 to 73.2)	180.5 (88.6 to 224.9)	343.0% (20.0 to 486.5%)
Belize	1.2 (1.0 to 1.6)	4.2 (3.0 to 5.1)	251.0% (80.4 to 352.0%)	0.8 (0.7 to 1.2)	2.8 (2.0 to 3.4)	239.4% (64.1 to 339.2%)	26.8 (23.2 to 34.8)	84.0 (59.2 to 101.3)	213.0% (62.1 to 301.7%)
Bermuda	10.1 (8.4 to 13.3)	10.3 (8.2 to 13.0)	1.6% (-32.5 to 0.4%)	7.3 (6.1 to 9.5)	6.3 (5.1 to 8.0)	-13.2% (-41.6 to 18.6%)	191.1 (158.2 to 255.6)	164.2 (130.9 to 209.4)	-14.1% (-43.3 to 18.2%)
Cuba	1.8 (1.6 to 2.8)	6.2 (3.6 to 7.8)	238.2% (27.6 to 347.1%)	1.2 (1.0 to 1.9)	3.7 (2.1 to 4.7)	222.2% (13.9 to 326.1%)	36.0 (32.2 to 56.6)	109.2 (63.1 to 137.8)	203.6% (15.7 to 306.3%)
Dominica	1.7 (1.4 to 2.6)	4.9 (2.6 to 6.6)	180.9% (12.9 to 333.9%)	1.1 (0.9 to 1.9)	3.3 (1.8 to 4.5)	189.6% (9.8 to 345.1%)	36.2 (28.4 to 54.5)	94.6 (51.2 to 128.7)	161.5% (8.8 to 302.7%)
Dominican Republic	1.4 (1.1 to 2.0)	2.5 (1.6 to 3.8)	81.9% (17.7 to 175.5%)	1.0 (0.8 to 1.5)	1.7 (1.1 to 2.5)	64.3% (6.3 to 152.5%)	30.8 (24.4 to 44.8)	50.3 (32.5 to 76.2)	63.1% (4.8 to 151.2%)
Grenada	2.5 (2.1 to 4.4)	11.2 (7.1 to 13.3)	343.3% (58.5 to 466.2%)	1.8 (1.5 to 3.2)	7.6 (4.8 to 9.0)	325.0% (49.2 to 441.1%)	54.0 (45.6 to 92.8)	216.5 (136.7 to 256.2)	301.0% (43.6 to 417.2%)
Guyana	3.0 (2.4 to 5.5)	11.8 (8.0 to 15.8)	300.0% (47.1 to 488.5%)	2.2 (1.8 to 4.2)	8.1 (5.7 to 10.7)	260.8% (35.7 to 423.1%)	66.4 (52.9 to 121.1)	247.6 (168.3 to 330.8)	272.8% (37.8 to 449.3%)
Haiti	2.2 (1.2 to 8.8)	4.4 (2.2 to 13.8)	102.1% (0.2 to 267.3%)	1.7 (0.9 to 6.7)	3.4 (1.7 to 10.3)	95.3% (-6.4 to 255.2%)	53.9 (27.8 to 219.9)	100.4 (50.5 to 316.6)	86.3% (-7.1 to 249.1%)
Jamaica	1.8 (1.6 to 2.9)	8.3 (4.8 to 10.6)	351.4% (59.5 to 513.9%)	1.3 (1.1 to 2.0)	5.5 (3.2 to 6.9)	322.5% (49.7 to 461.6%)	37.9 (32.9 to 59.8)	157.0 (91.5 to 203.1)	314.5% (47.4 to 464.8%)
Puerto Rico	2.4 (2.2 to 3.4)	6.9 (5.0 to 9.0)	185.8% (54.8 to 286.8%)	1.6 (1.5 to 2.2)	4.2 (3.0 to 5.5)	158.3% (39.5 to 243.9%)	45.4 (40.5 to 64.6)	115.6 (83.2 to 150.5)	154.9% (37.4 to 2.4%)
Saint Kitts and Nevis	3.4 (2.9 to 4.9)	10.1 (6.9 to 13.4)	197.2% (51.3 to 318.2%)	2.4 (2.0 to 3.4)	7.1 (4.8 to 8.9)	201.4% (40.5 to 303.8%)	68.6 (58.7 to 98.0)	188.2 (127.8 to 248.3)	174.2% (40.9 to 2.8%)
Saint Lucia	2.7 (2.4 to 4.1)	9.3 (6.4 to 11.7)	240.8% (51.1 to 359.4%)	2.0 (1.7 to 3.0)	6.1 (4.1 to 7.6)	209.3% (31.5 to 313.0%)	58.0 (50.9 to 86.5)	177.6 (120.5 to 223.9)	206.3% (35.7 to 314.8%)
Saint Vincent and the Grenadines	2.5 (2.1 to 4.5)	9.1 (6.7 to 10.8)	268.2% (48.3 to 368.1%)	1.8 (1.5 to 3.3)	6.2 (4.6 to 7.3)	250.8% (39.9 to 344.6%)	51.2 (43.6 to 91.8)	178.3 (132.8 to 211.3)	248.2% (40.9 to 347.7%)
Suriname	2.5 (1.9 to 4.4)	8.9 (4.6 to 11.5)	256.6% (25.0 to 460.9%)	1.8 (1.4 to 3.3)	6.1 (3.2 to 7.9)	231.2% (16.0 to 414.5%)	54.5 (40.8 to 95.9)	178.4 (92.4 to 232.1)	227.3% (15.2 to 410.2%)
Trinidad and Tobago	1.9 (1.7 to 3.2)	10.0 (4.3 to 13.9)	428.3% (31.2 to 672.8%)	1.4 (1.2 to 2.3)	6.6 (2.9 to 9.0)	385.9% (22.1 to 597.4%)	39.7 (35.1 to 67.0)	192.2 (83.3 to 268.3)	384.2% (20.9 to 605.7%)
United States Virgin Islands	7.5 (5.9 to 11.5)	14.1 (10.3 to 18.3)	88.8% (7.4 to 177.3%)	5.5 (4.3 to 8.1)	9.8 (7.0 to 12.3)	79.8% (2.6 to 157.5%)	151.8 (118.2 to 233.4)	262.3 (190.8 to 336.5)	72.8% (-1.8 to 156.9%)
**Tropical Latin America**	5.6 (5.3 to 5.8)	6.1 (5.6 to 6.7)	9.9% (0.3 to 20.9%)	4.1 (3.9 to 4.3)	4.1 (3.7 to 4.4)	-0.3% (-9.2 to 9.1%)	118.2 (112.6 to 123.2)	115.9 (106.2 to 126.1)	-2.0% (-10.7 to 7.0%)
Brazil	5.7 (5.4 to 5.9)	6.1 (5.6 to 6.7)	8.6% (-1.0 to 19.6%)	4.2 (3.9 to 4.3)	4.1 (3.7 to 4.5)	-1.6% (-10.0 to 7.9%)	119.8 (114.2 to 124.8)	116.1 (106.6 to 126.4)	-3.1% (-11.6 to 6.5%)
Paraguay	2.3 (1.9 to 3.0)	5.6 (3.1 to 7.8)	139.2% (12.3 to 261.0%)	1.7 (1.3 to 2.2)	3.8 (2.1 to 5.2)	124.3% (2.1 to 241.6%)	50.3 (39.8 to 63.9)	106.7 (59.3 to 148.9)	112.1% (0.5 to 221.8%)
**East Asia**	2.6 (2.0 to 3.6)	4.6 (3.4 to 5.7)	77.0% (8.7 to 147.0%)	1.8 (1.4 to 2.6)	2.8 (2.1 to 3.5)	56.6% (-7.0 to 124.6%)	56.1 (43.4 to 77.9)	81.2 (60.7 to 102.3)	44.8% (-10.9 to 106.5%)
China	2.6 (2.0 to 3.6)	4.5 (3.3 to 5.7)	77.3% (6.3 to 150.5%)	1.8 (1.4 to 2.6)	2.8 (2.0 to 3.5)	57.1% (-9.9 to 127.8%)	55.6 (42.9 to 77.8)	80.5 (59.4 to 102.6)	44.9% (-12.1 to 108.4%)
Democratic People's Republic of Korea	3.1 (1.5 to 6.5)	4.7 (3.1 to 7.6)	53.7% (-15。0 to 206.5%)	1.8 (0.9 to 3.7)	2.7 (1.8 to 4.2)	45.0% (-17.7 to 192.0%)	59.1 (29.1 to 125.8)	84.1 (55.0 to 137.3)	42.3% (-21.7 to 189.5%)
Taiwan (province of China)	4.7 (4.4 to 5.0)	8.7 (6.3 to 11.6)	84.1% (31.5 to 146.9%)	2.6 (2.5 to 2.8)	3.8 (2.8 to 5.1)	46.0% (4.1 to 94.1%)	79.9 (75.3 to 84.7)	116.5 (84.8 to 155.7)	45.8% (3.7 to 96.0%)
**Southeast Asia**	5.0 (4.0 to 7.2)	8.0 (6.2 to 11.0)	60.7% (0.3 to 101.4%)	3.4 (2.8 to 4.8)	4.8 (3.8 to 6.6)	37.9% (-10.3 to 71.5%)	105.2 (84.0 to 152.2)	144.2 (112.7 to 201.8)	37.1% (-15.2 to 72.7%)
Cambodia	3.7 (2.0 to 10.5)	8.4 (5.5 to 14.0)	126.2% (11.0 to 336.3%)	2.7 (1.6 to 7.2)	5.3 (3.6 to 8.8)	91.7% (-2.7 to 285.3%)	85.1 (45.7 to 243.7)	161.4 (105.1 to 271.4)	89.6% (-8.7 to 266.5%)
Indonesia	4.5 (3.3 to 8.5)	8.2 (5.1 to 15.2)	79.6% (-0.6 to 154.5%)	3.2 (2.4 to 5.7)	5.1 (3.2 to 9.7)	59.3% (-9.5 to 122.4%)	99.5 (73.5 to 186.3)	153.7 (97.0 to 289.0)	54.5% (-17.7 to 118.6%)
Lao People's Democratic Republic	4.3 (2.2 to 12.9)	7.9 (5.2 to 12.6)	83.5% (-20.1 to 250.7%)	3.3 (1.7 to 9.4)	5.2 (3.5 to 8.1)	57.9% (-27.4 to 196.3%)	102.3 (50.2 to 311.4)	159.7 (104.0 to 255.5)	56.1% (-32.3 to 204.3%)
Malaysia	4.9 (3.8 to 7.2)	8.1 (6.0 to 11.0)	64.7% (-3.1 to 141.2%)	3.5 (2.7 to 4.9)	4.8 (3.6 to 6.3)	38.2% (-16.5 to 101.7%)	99.9 (77.5 to 148.3)	136.8 (101.7 to 185.2)	37.0% (-21.6 to 103.6%)
Maldives	7.3 (4.0 to 23.1)	10.0 (7.8 to 13.0)	36.3% (-62.9 to 174.5%)	5.3 (2.9 to 15.6)	5.6 (4.4 to 7.1)	5.8% (-68.3 to 104.5%)	152.3 (82.4 to 482.0)	156.8 (121.8 to 206.3)	3.0% (-72.8 to 108.5%)
Mauritius	5.1 (4.6 to 5.7)	9.7 (7.6 to 12.3)	88.9% (46.5 to 1.4%)	3.2 (2.9 to 3.5)	5.2 (4.1 to 6.6)	64.9% (28.7 to 110.6%)	97.8 (87.6 to 108.1)	160.5 (124.6 to 204.4)	64.1% (27.1 to 113.0%)
Myanmar	5.4 (3.4 to 12.0)	8.1 (5.7 to 11.4)	49.7% (-27.3 to 157.1%)	4.0 (2.5 to 8.7)	5.1 (3.7 to 7.1)	28.2% (-36.6 to 114.3%)	122.9 (76.5 to 281.5)	155.7 (109.5 to 219.6)	26.7% (-42.5 to 111.7%)
Philippines	7.5 (5.8 to 9.7)	9.9 (7.3 to 13.3)	32.3% (-10.3 to 94.8%)	5.0 (3.8 to 6.6)	5.8 (4.3 to 8.1)	17.2% (-18.5 to 76.6%)	153.0 (118.3 to 197.6)	183.7 (135.7 to 247.3)	20.0% (-17.7 to 79.6%)
Sri Lanka	3.2 (2.7 to 4.1)	5.8 (3.9 to 8.0)	79.8% (19.0 to 153.8%)	2.1 (1.7 to 2.8)	3.2 (2.2 to 4.4)	52.5% (-1.2 to 115.1%)	64.1 (53.4 to 82.4)	92.5 (61.7 to 127.2)	44.5% (-4.3 to 105.8%)
Seychelles	8.7 (7.2 to 12.1)	15.7 (12.3 to 19.2)	80.1% (25.8 to 138.0%)	5.5 (4.5 to 7.9)	8.9 (7.0 to 10.8)	60.4% (11.4 to 109.8%)	173.6 (143.3 to 242.1)	263.4 (207.0 to 322.1)	51.8% (5.5 to 100.5%)
Thailand	5.5 (4.4 to 6.9)	7.1 (4.9 to 10.5)	29.7% (-18.3 to 96.2%)	3.6 (3.0 to 4.6)	3.8 (2.7 to 5.4)	3.5% (-32.4 to 53.1%)	109.5 (88.9 to 138.2)	113.1 (77.7 to 167.5)	3.2% (-34.6 to 56.4%)
Timor-Leste	3.0 (1.5 to 7.8)	5.6 (3.3 to 8.1)	83.3% (-15.4 to 248.5%)	2.3 (1.2 to 5.8)	3.6 (2.3 to 5.2)	55.3% (-20.8 to 188.8%)	70.0 (34.3 to 180.7)	110.3 (65.6 to 160.1)	57.6% (-27.1 to 203.1%)
Viet Nam	3.9 (2.9 to 5.1)	7.0 (5.1 to 9.1)	80.5% (19.7 to 158.0%)	2.9 (2.2 to 3.7)	4.3 (3.0 to 5.5)	48.7% (1.3 to 106.4%)	81.2 (60.9 to 107.9)	121.0 (88.0 to 158.8)	48.9% (-1.8 to 115.7%)
**Oceania**	2.7 (1.9 to 5.5)	4.3 (2.9 to 8.0)	58.1% (6.1 to 124.7%)	2.0 (1.4 to 3.9)	2.9 (2.1 to 5.3)	48.6% (-1.2 to 109.1%)	58.0 (41.2 to 120.0)	85.5 (57.6 to 163.8)	47.3% (-2.4 to 111.9%)
American Samoa	9.5 (7.2 to 13.6)	15.6 (11.7 to 21.8)	64.3% (3.8 to 152.4%)	6.6 (5.1 to 9.4)	10.0 (7.6 to 14.0)	50.4% (-5.2 to 126.4%)	186.1 (139.0 to 269.9)	278.7 (209.1 to 394.2)	49.8% (-5.3 to 132.3%)
Cook Islands	2.6 (1.8 to 4.1)	5.0 (3.4 to 6.6)	92.1% (4.6 to 211.6%)	1.6 (1.2 to 2.6)	2.8 (2.0 to 3.7)	73.6% (-2.9 to 174.3%)	47.7 (33.4 to 76.0)	80.4 (55.6 to 106.8)	68.7% (-8.0 to 175.1%)
Micronesia(Federated States of)	3.8 (2.3 to 9.5)	8.5 (4.7 to 16.0)	123.5% (7.3 to 349.9%)	2.8 (1.7 to 6.9)	5.7 (3.3 to 10.5)	103.1% (1.8 to 307.3%)	83.9 (49.9 to 211.6)	164.7 (90.6 to 313.5)	96.2% (-6.7 to 297.4%)
Fiji	2.2 (1.7 to 3.0)	3.0 (2.2 to 3.9)	33.9% (-11.1 to 97.3%)	1.6 (1.2 to 2.1)	1.9 (1.4 to 2.5)	23.8% (-16.6 to 79.6%)	46.4 (35.3 to 63.4)	57.0 (41.1 to 76.0)	22.9% (-19.4 to 82.5%)
Guam	4.0 (3.2 to 6.1)	6.7 (5.3 to 8.7)	66.7% (9.3 to 131.6%)	2.6 (2.0 to 3.9)	3.9 (3.1 to 4.9)	50.9% (-1.6 to 110.3%)	73.3 (57.7 to 112.1)	114.0 (89.9 to 146.4)	55.5% (0.7 to 118.2%)
Kiribati	2.2 (1.6 to 3.2)	3.7 (2.3 to 5.6)	66.6% (-7.7 to 149.0%)	1.6 (1.2 to 2.5)	2.5 (1.6 to 4.0)	57.9% (-10.3 to 134.9%)	51.4 (37.8 to 73.5)	78.7 (50.3 to 117.8)	53.1% (-15.1 to 131.4%)
Marshall Islands	3.6 (2.4 to 8.2)	7.2 (3.9 to 14.5)	102.0% (17.1 to 250.6%)	2.6 (1.8 to 6.0)	5.0 (2.7 to 9.6)	88.6% (9.4 to 228.0%)	78.7 (52.0 to 180.5)	146.4 (79.2 to 296.5)	86.1% (8.3 to 227.6%)
Nauru	4.3 (2.4 to 9.6)	7.7 (4.6 to 13.8)	78.8% (-1.2 to 252.1%)	2.9 (1.6 to 6.5)	4.8 (2.9 to 8.6)	64.5% (-9.2 to 227.4%)	87.1 (48.0 to 196.3)	140.8 (83.3 to 255.2)	61.6% (-11.3 to 231.1%)
Niue	4.7 (3.3 to 7.4)	8.6 (5.3 to 12.7)	80.2% (1.8 to 195.8%)	3.1 (2.2 to 4.9)	4.9 (3.1 to 7.3)	57.6% (-8.7 to 150.7%)	91.7 (62.0 to 145.3)	142.2 (88.6 to 210.7)	55.1% (-11.3 to 154.5%)
Northern Mariana Islands	4.4 (2.9 to 8.1)	7.8 (6.1 to 10.1)	75.5% (-2.1 to 176.5%)	2.8 (1.9 to 5.0)	4.6 (3.6 to 5.8)	63.3% (-12.0 to 155.2%)	80.5 (53.2 to 145.1)	126.3 (97.8 to 164.5)	56.9% (-13.1 to 147.9%)
Palau	3.1 (2.1 to 4.8)	4.7 (3.5 to 6.2)	50.4% (-9.1 to 126.9%)	2.1 (1.5 to 3.2)	2.9 (2.2 to 3.7)	34.6% (-17.2 to 100.2%)	60.4 (40.7 to 93.3)	81.0 (60.5 to 107.5)	34.2% (-18.8 to 101.8%)
Papua New Guinea	2.3 (1.4 to 5.4)	3.7 (2.3 to 7.8)	60.1% (1.6 to 146.4%)	1.7 (1.0 to 3.9)	2.6 (1.6 to 5.2)	50.6% (-5.6 to 131.1%)	51.8 (31.2 to 122.4)	77.0 (47.1 to 160.3)	48.7% (-7.2 to 132.2%)
Samoa	8.0 (5.6 to 11.1)	13.1 (6.9 to 23.5)	63.0% (-6.6 to 173.4%)	5.7 (4.0 to 8.0)	8.5 (4.4 to 15.1)	49.0% (-14.9 to 142.5%)	166.4 (116.5 to 230.6)	246.0 (126.9 to 440.2)	47.8% (-16.7 to 149.0%)
Solomon Islands	2.6 (1.2 to 11.9)	6.0 (3.2 to 19.3)	126.7% (18.7 to 366.3%)	1.8 (0.9 to 7.9)	3.8 (2.1 to 11.7)	108.3% (11.0 to 325.9%)	58.8 (27.1 to 273.7)	118.7 (62.7 to 389.3)	101.7% (6.8 to 319.2%)
Tokelau	3.4 (1.7 to 8.9)	6.4 (3.9 to 11.1)	87.2% (0.3 to 245.6%)	2.4 (1.2 to 6.1)	3.9 (2.4 to 6.7)	63.0% (-11.6 to 200.1%)	71.7 (36.5 to 190.7)	114.1 (68.7 to 201.6)	59.2% (-15.7 to 199.9%)
Tonga	3.8 (2.3 to 5.0)	6.3 (3.9 to 9.0)	62.9% (3.2 to 140.5%)	2.8 (1.6 to 3.6)	4.2 (2.6 to 5.9)	50.2% (-4.1 to 118.1%)	78.5 (48.4 to 103.4)	116.4 (71.9 to 168.2)	48.2% (-6.7 to 119.9%)
Tuvalu	3.9 (2.6 to 9.1)	6.9 (4.1 to 12.3)	76.4% (1.1 to 191.1%)	2.8 (1.9 to 6.4)	4.5 (2.7 to 7.8)	60.5% (-5.8 to 164.7%)	84.8 (56.1 to 197.2)	131.5 (78.0 to 233.2)	55.0% (-11.6 to 156.3%)
Vanuatu	2.0 (0.9 to 6.7)	4.3 (2.1 to 10.4)	108.7% (15.2 to 306.7%)	1.5 (0.7 to 5.0)	3.0 (1.5 to 7.2)	94.7% (7.6 to 269.4%)	44.7 (20.5 to 146.0)	87.8 (42.5 to 216.2)	96.2% (7.6 to 285.0%)
**North Africa and Middle East**	3.3 (2.4 to 6.0)	5.3 (4.3 to 6.1)	58.1% (-17.0 to 116.8%)	2.6 (1.9 to 4.5)	3.5 (2.9 to 4.2)	38.0% (-25.4 to 88.6%)	73.1 (52.7 to 134.4)	99.0 (80.8 to 115.5)	35.4% (-28.1 to 88.5%)
Afghanistan	2.6 (1.0 to 14.1)	3.7 (1.8 to 13.5)	39.1% (-10.7 to 141.5%)	2.1 (0.8 to 10.8)	2.8 (1.4 to 10.1)	33.0% (-13.6 to 127.5%)	65.3 (23.7 to 363.6)	85.3 (41.2 to 327.4)	30.6% (-15.8 to 131.5%)
Algeria	2.6 (1.9 to 3.5)	4.1 (2.7 to 5.4)	56.9% (-2.5 to 124.7%)	1.9 (1.4 to 2.6)	2.7 (1.8 to 3.5)	39.6% (-10.9 to 94.7%)	55.5 (39.6 to 75.7)	76.8 (50.1 to 101.5)	38.3% (-13.7 to 96.9%)
Bahrain	8.9 (6.6 to 12.9)	10.9 (7.7 to 16.3)	21.9% (-35.4 to 116.5%)	7.2 (5.0 to 10.5)	7.8 (5.7 to 10.9)	8.3% (-41.0 to 87.0%)	180.7 (134.4 to 266.1)	187.1 (131.0 to 284.0)	3.5% (-45.9 to 85.8%)
Egypt	1.7 (1.3 to 3.4)	3.3 (1.8 to 6.1)	89.4% (-0.8 to 195.8%)	1.3 (0.9 to 2.7)	2.2 (1.2 to 4.4)	70.1% (-3.6 to 164.6%)	39.0 (28.7 to 75.9)	64.8 (35.6 to 121.8)	66.2% (-12.4 to 158.7%)
Iran	2.3 (1.6 to 4.2)	4.8 (3.3 to 5.4)	106.5% (-2.8 to 206.4%)	1.6 (1.1 to 3.0)	3.0 (2.2 to 3.4)	87.0% (-10.0 to 177.2%)	48.1 (32.4 to 89.7)	85.4 (59.5 to 97.0)	77.5% (-16.2 to 161.5%)
Iraq	3.3 (1.8 to 5.9)	6.0 (4.3 to 8.3)	83.5% (-16.7 to 279.9%)	2.4 (1.3 to 4.6)	3.7 (2.7 to 5.3)	57.2% (-24.3 to 224.0%)	72.8 (40.5 to 129.2)	115.8 (82.3 to 160.1)	59.0% (-27.5 to 234.9%)
Jordan	3.6 (2.4 to 5.7)	5.4 (3.9 to 7.0)	51.2% (-25.3 to 136.5%)	2.7 (1.8 to 4.2)	3.6 (2.6 to 4.6)	31.5% (-33.8 to 108.5%)	76.1 (51.8 to 121.9)	98.3 (70.5 to 127.1)	29.1% (-35.4 to 108.3%)
Kuwait	6.7 (5.5 to 9.1)	5.7 (3.7 to 8.3)	-15.5% (-47.1 to 37.5%)	4.7 (3.8 to 6.5)	3.7 (2.5 to 5.3)	-20.9% (-50.2 to 27.3%)	127.2 (103.5 to 173.5)	95.1 (61.7 to 138.8)	-25.2% (-53.1 to 21.7%)
Lebanon	6.9 (4.9 to 10.7)	11.8 (8.0 to 15.7)	70.0% (-20.3 to 167.5%)	5.1 (3.6 to 7.7)	7.5 (5.1 to 10.0)	47.0% (-31.3 to 131.0%)	143.7 (101.1 to 219.9)	201.5 (136.5 to 269.5)	40.3% (-34.2 to 122.6%)
Libya	4.2 (2.6 to 7.0)	7.3 (5.2 to 10.5)	75.2% (-11.3 to 235.6%)	3.2 (2.0 to 5.3)	5.1 (3.6 to 7.2)	62.2% (-17.3 to 211.4%)	88.1 (55.2 to 148.3)	141.4 (99.1 to 201.6)	60.6% (-17.7 to 209.9%)
Morocco	3.9 (2.9 to 5.7)	7.7 (5.1 to 10.8)	98.2% (-1.5 to 212.2%)	3.1 (2.3 to 4.4)	5.4 (3.7 to 7.3)	75.4% (-9.6 to 170.0%)	88.8 (65.4 to 128.1)	156.7 (103.3 to 220.7)	76.5% (-12.9 to 179.0%)
Palestine	3.3 (1.8 to 6.7)	6.6 (3.8 to 8.4)	98.5% (-13.5 to 297.4%)	2.5 (1.3 to 4.8)	4.6 (2.7 to 5.8)	86.3% (-16.2 to 274.5%)	70.2 (37.2 to 140.5)	125.8 (72.9 to 158.5)	79.1% (-23.3 to 262.2%)
Oman	2.7 (1.3 to 4.9)	6.8 (4.5 to 9.1)	149.5% (-5.8 to 407.8%)	2.1 (1.0 to 3.7)	4.6 (3.1 to 6.0)	119.8% (-14.2 to 350.7%)	57.3 (27.2 to 102.8)	122.1 (79.8 to 162.9)	113.2% (-20.1 to 337.2%)
Qatar	6.7 (3.8 to 10.5)	11.4 (7.6 to 15.6)	70.0% (-16.1 to 188.7%)	5.3 (2.9 to 8.2)	8.2 (5.1 to 11.3)	53.6% (-21.1 to 157.8%)	137.8 (78.7 to 219.1)	195.6 (129.2 to 269.2)	42.0% (-32.5 to 140.6%)
Saudi Arabia	2.3 (1.5 to 4.2)	6.3 (4.4 to 9.1)	168.2% (27.4 to 355.9%)	2.1 (1.4 to 4.0)	3.9 (2.7 to 5.8)	82.0% (-14.7 to 224.4%)	61.8 (39.6 to 112.8)	113.3 (78.3 to 163.2)	83.3% (-14.9 to 223.5%)
Sudan	1.6 (0.6 to 6.0)	3.1 (1.9 to 5.4)	95.0% (-11.7 to 322.7%)	1.2 (0.5 to 4.6)	2.2 (1.4 to 3.7)	74.5% (-15.3 to 264.0%)	37.0 (14.6 to 144.9)	63.4 (39.8 to 110.6)	71.4% (-20.6 to 275.9%)
Syrian Arab Republic	1.9 (1.2 to 3.1)	3.6 (2.3 to 5.3)	87.4% (-17.1 to 2.5%)	1.4 (0.9 to 2.2)	2.3 (1.5 to 3.4)	67.5% (-22.3 to 221.7%)	41.3 (26.7 to 66.8)	66.5 (42.7 to 100.2)	61.1% (-29.7 to 209.1%)
Tunisia	3.4 (2.5 to 4.7)	5.4 (3.6 to 7.4)	87.4% (-17.1 to 254.1%)	2.5 (1.8 to 3.4)	3.5 (2.4 to 4.8)	38.6% (-22.4 to 115.3%)	69.6 (51.7 to 95.8)	96.9 (65.0 to 133.6)	39.3% (-23.9 to 119.6%)
Turkey	6.2 (3.5 to 10.9)	6.7 (4.1 to 8.8)	59.8% (-11.1 to 149.5%)	4.8 (2.8 to 8.4)	4.4 (2.7 to 5.7)	-7.8% (-52.8 to 42.6%)	134.5 (76.1 to 242.5)	119.6 (74.1 to 157.6)	-11.1% (-56.1 to 39.6%)
United Arab Emirates	5.3 (2.8 to 11.7)	8.5 (4.9 to 15.7)	8.6% (-45.3 to 69.4%)	3.9 (2.0 to 9.0)	5.7 (3.1 to 10.6)	43.9% (-36.4 to 174.8%)	114.3 (59.8 to 252.9)	163.1 (92.9 to 307.9)	42.7% (-34.3 to 169.5%)
Yemen	1.4 (0.4 to 5.9)	2.6 (1.5 to 6.0)	58.9% (-28.0 to 202.4%)	1.1 (0.3 to 4.5)	1.9 (1.1 to 4.3)	70.6% (-7.7 to 328.1%)	33.3 (9.8 to 140.5)	56.5 (33.4 to 132.9)	69.6% (-12.1 to 331.5%)
**South Asia**	3.2 (2.4 to 4.6)	5.9 (4.6 to 7.3)	86.3% (15.0 to 158.6%)	2.6 (2.0 to 3.8)	4.4 (3.4 to 5.5)	68.8% (4.8 to 133.2%)	73.0 (54.5 to 107.7)	125.3 (95.9 to 157.3)	71.7% (4.6 to 140.4%)
Bangladesh	2.5 (1.5 to 4.6)	4.6 (2.8 to 9.9)	80.9% (-20.5 to 195.5%)	2.1 (1.2 to 3.9)	3.4 (2.1 to 7.2)	60.6% (-25.9 to 159.0%)	58.8 (34.8 to 107.4)	94.5 (59.8 to 204.5)	60.7% (-31.5 to 169.3%)
Bhutan	3.0 (1.5 to 6.3)	6.8 (3.9 to 15.3)	129.2% (-10.3 to 322.5%)	2.5 (1.2 to 5.2)	5.1 (3.0 to 11.7)	103.0% (-16.9 to 267.0%)	69.6 (34.4 to 147.9)	139.0 (78.9 to 310.8)	99.8% (-23.0 to 280.5%)
India	2.9 (1.9 to 4.4)	5.0 (3.8 to 6.2)	74.7% (13.7 to 146.2%)	2.3 (1.6 to 3.6)	3.8 (2.9 to 4.7)	61.3% (7.0 to 128.9%)	65.6 (44.0 to 102.2)	104.8 (78.7 to 131.6)	59.7% (6.3 to 128.1%)
Nepal	2.4 (1.2 to 4.8)	6.0 (3.6 to 13.5)	144.4% (5.8 to 344.2%)	2.0 (1.0 to 4.0)	4.6 (2.8 to 10.3)	124.9% (2.4 to 313.9%)	57.7 (29.3 to 114.9)	126.0 (76.3 to 283.9)	118.3% (-6.4 to 293.4%)
Pakistan	6.4 (5.0 to 8.2)	15.8 (8.5 to 25.4)	146.8% (14.3 to 328.2%)	5.2 (4.0 to 6.6)	11.8 (6.2 to 19.3)	128.8% (7.5 to 309.2%)	152.0 (117.6 to 188.1)	348.4 (182.7 to 562.4)	129.1% (7.4 to 310.1%)
**Southern sub-Saharan Africa**	4.7 (4.0 to 5.6)	6.7 (5.5 to 8.0)	42.5% (10.7 to 67.9%)	3.6 (3.0 to 4.4)	5.1 (4.2 to 6.1)	41.2% (11.8 to 68.3%)	102.0 (86.7 to 122.2)	138.5 (113.3 to 166.7)	35.8% (4.9 to 63.7%)
Botswana	4.2 (2.8 to 6.1)	8.1 (5.3 to 12.3)	94.3% (11.5 to 211.1%)	3.3 (2.3 to 4.9)	5.9 (3.9 to 8.9)	77.3% (5.2 to 179.6%)	92.5 (61.7 to 136.8)	163.0 (104.8 to 250.5)	76.3% (-1.0 to 184.0%)
Lesotho	3.4 (2.2 to 5.1)	7.6 (4.2 to 14.7)	125.2% (19.7 to 340.9%)	2.8 (1.9 to 4.2)	6.3 (3.5 to 11.9)	120.9% (19.3 to 326.3%)	76.7 (49.3 to 115.8)	169.1 (91.0 to 331.9)	120.3% (15.0 to 335.2%)
Namibia	3.2 (2.2 to 4.4)	4.9 (3.4 to 6.9)	53.4% (-9.2 to 147.0%)	2.6 (1.9 to 3.6)	3.7 (2.6 to 5.2)	42.2% (-11.8 to 120.9%)	74.1 (51.0 to 102.7)	103.1 (69.9 to 148.6)	39.2% (-18.4 to 127.9%)
South Africa	4.6 (3.9 to 5.4)	6.1 (4.8 to 7.6)	32.6% (-0.4 to 56.5%)	3.6 (2.9 to 4.2)	4.7 (3.6 to 5.7)	32.6% (6.0 to 55.3%)	100.5 (85.2 to 117.3)	124.6 (94.4 to 155.2)	24.1% (-7.4 to 49.2%)
Eswatini	5.4 (2.9 to 10.9)	8.0 (4.4 to 13.8)	48.7% (-20.6 to 177.5%)	4.4 (2.4 to 8.8)	6.3 (3.5 to 10.9)	43.3% (-22.6 to 160.5%)	121.2 (63.9 to 242.9)	172.1 (92.9 to 301.4)	42.0% (-26.0 to 172.8%)
Zimbabwe	5.7 (4.4 to 7.5)	9.7 (6.7 to 13.2)	69.6% (5.9 to 154.4%)	4.5 (3.5 to 5.8)	7.5 (5.2 to 10.1)	67.6% (5.3 to 152.4%)	122.4 (93.9 to 158.7)	210.8 (140.3 to 290.2)	72.2% (6.6 to 158.5%)
**Western sub-Saharan Africa**	2.3 (1.7 to 3.3)	4.1 (2.9 to 5.5)	78.1% (4.4 to 169.9%)	1.9 (1.4 to 2.7)	3.2 (2.2 to 4.2)	70.9% (1.6 to 153.3%)	53.4 (40.6 to 76.9)	89.4 (63.8 to 120.3)	67.4% (-0.2 to 147.0%)
Benin	2.1 (1.6 to 2.7)	3.4 (2.5 to 4.7)	63.5% (9.4 to 127.7%)	1.7 (1.3 to 2.2)	2.6 (2.0 to 3.5)	57.5% (6.7 to 115.3%)	49.4 (37.1 to 63.7)	76.2 (55.0 to 106.0)	54.3% (3.5 to 115.0%)
Burkina Faso	2.0 (1.4 to 3.1)	3.3 (2.5 to 4.5)	62.5% (9.4 to 132.8%)	1.7 (1.1 to 2.6)	2.6 (1.9 to 3.5)	55.3% (5.9 to 122.1%)	48.6 (32.6 to 76.0)	75.0 (55.5 to 102.8)	54.4% (3.5 to 122.9%)
Cameroon	3.1 (2.3 to 4.5)	5.2 (3.1 to 8.2)	65.4% (2.9 to 149.9%)	2.5 (1.8 to 3.6)	4.0 (2.4 to 6.3)	57.4% (-3.5 to 137.8%)	74.5 (54.2 to 105.7)	114.7 (68.5 to 184.0)	54.0% (-5.4 to 136.3%)
Cape Verde	1.0 (0.7 to 1.5)	3.8 (2.0 to 5.4)	268.0% (43.1 to 532.3%)	0.7 (0.5 to 1.1)	2.5 (1.3 to 3.8)	249.6% (25.7 to 552.5%)	23.9 (16.4 to 35.0)	76.1 (38.6 to 106.5)	219.0% (25.1 to 443.0%)
Chad	1.6 (1.0 to 2.4)	2.2 (1.5 to 3.5)	44.1% (3.0 to 96.5%)	1.3 (0.9 to 2.0)	1.8 (1.2 to 2.8)	42.3% (2.5 to 93.1%)	37.9 (25.1 to 58.2)	52.5 (34.8 to 83.4)	38.6% (-1.0 to 92.0%)
Côte d’Ivoire	2.4 (1.8 to 3.1)	4.0 (2.7 to 5.7)	69.8% (9.8 to 153.7%)	1.9 (1.5 to 2.5)	3.2 (2.2 to 4.4)	65.2% (7.1 to 148.9%)	56.4 (43.3 to 73.6)	91.1 (61.4 to 130.2)	61.6% (3.1 to 144.9%)
Gambia	2.0 (1.5 to 2.7)	4.4 (2.5 to 7.0)	115.3% (13.5 to 281.2%)	1.7 (1.2 to 2.2)	3.4 (2.0 to 5.5)	105.0% (7.8 to 261.1%)	46.9 (33.3 to 63.1)	97.7 (55.4 to 160.1)	108.2% (6.1 to 273.8%)
Ghana	2.6 (1.8 to 3.7)	5.0 (2.8 to 8.4)	93.8% (5.9 to 205.2%)	2.0 (1.4 to 2.9)	3.7 (2.1 to 6.2)	84.4% (-2.7 to 188.1%)	61.1 (42.7 to 86.9)	109.4 (60.6 to 180.1)	79.0% (-3.1 to 181.8%)
Guinea	3.2 (2.5 to 4.0)	5.2 (3.0 to 7.8)	62.6% (-8.8 to 152.3%)	2.7 (2.1 to 3.3)	4.2 (2.4 to 6.2)	56.6% (-13.1 to 144.2%)	77.5 (60.4 to 97.2)	120.7 (69.5 to 182.6)	55.8% (-14.4 to 142.8%)
Guinea-Bissau	2.5 (1.6 to 4.3)	4.3 (2.7 to 7.0)	70.6% (-0.8 to 167.6%)	2.0 (1.3 to 3.3)	3.3 (2.0 to 5.4)	64.8% (-5.7 to 159.9%)	61.0 (39.5 to 106.2)	97.8 (60.3 to 160.2)	60.4% (-6.7 to 151.0%)
Liberia	2.4 (1.7 to 3.6)	4.4 (2.6 to 7.3)	88.1% (3.5 to 207.7%)	1.9 (1.4 to 2.9)	3.5 (2.0 to 5.8)	79.7% (-2.9 to 196.2%)	57.0 (42.3 to 85.0)	100.3 (59.0 to 165.2)	76.0% (-4.6 to 190.7%)
Mali	1.6 (1.2 to 1.9)	2.5 (1.4 to 3.5)	59.9% (-13.1 to 127.6%)	1.3 (1.0 to 1.6)	1.9 (1.1 to 2.7)	52.0% (-16.5 to 112.6%)	38.4 (30.1 to 47.4)	57.6 (32.3 to 80.5)	50.3% (-19.2 to 116.3%)
Mauritania	3.0 (2.3 to 4.0)	5.0 (2.4 to 7.6)	68.5% (-17.8 to 162.8%)	2.4 (1.9 to 3.3)	3.9 (1.9 to 5.8)	58.6% (-20.8 to 143.0%)	70.7 (54.7 to 95.4)	108.3 (53.6 to 166.8)	53.1% (-26.1 to 142.5%)
Niger	1.5 (1.0 to 2.5)	2.1 (1.3 to 3.4)	39.4% (-7.1 to 97.4%)	1.3 (0.8 to 2.0)	1.7 (1.0 to 2.6)	35.8% (-10.6 to 91.7%)	37.8 (25.4 to 61.7)	49.5 (29.4 to 78.3)	31.1% (-12.8 to 87.1%)
Nigeria	2.2 (1.4 to 3.8)	4.2 (2.6 to 6.2)	88.1% (-7.9 to 257.1%)	1.9 (1.2 to 3.2)	3.3 (2.1 to 4.9)	80.9% (-11.7 to 240.0%)	51.1 (32.0 to 91.8)	90.7 (59.0 to 135.4)	77.4% (-15.1 to 240.1%)
São Tomé and Príncipe	4.0 (3.0 to 5.1)	9.3 (4.7 to 16.1)	133.6% (21.4 to 288.8%)	3.2 (2.4 to 4.0)	6.8 (3.4 to 12.1)	111.3% (12.6 to 262.0%)	93.6 (70.0 to 119.8)	201.6 (102.2 to 351.2)	115.4% (12.0 to 263.4%)
Senegal	2.2 (1.7 to 2.9)	4.1 (2.6 to 6.1)	83.8% (6.8 to 186.4%)	1.8 (1.4 to 2.4)	3.2 (2.0 to 4.7)	79.0% (4.7 to 175.4%)	53.6 (39.2 to 70.9)	92.6 (57.4 to 135.8)	72.7% (-2.1 to 170.9%)
Sierra Leone	2.0 (1.4 to 2.9)	3.6 (2.3 to 5.6)	84.1% (11.6 to 177.7%)	1.6 (1.1 to 2.4)	2.8 (1.8 to 4.4)	76.9% (6.3 to 163.3%)	47.3 (32.1 to 69.9)	82.1 (52.0 to 127.8)	73.7% (4.2 to 165.5%)
Togo	2.3 (1.7 to 3.1)	3.9 (2.4 to 5.8)	65.1% (-2.9 to 155.8%)	1.9 (1.4 to 2.4)	3.0 (1.8 to 4.4)	60.4% (-5.1 to 148.7%)	55.2 (40.5 to 72.0)	86.3 (54.3 to 130.4)	56.4% (-7.9 to 144.6%)
**Eastern sub-Saharan Africa**	3.9 (2.4 to 8.3)	6.0 (4.9 to 7.2)	55.0% (-19.6 to 137.6%)	3.2 (2.0 to 6.7)	4.8 (4.0 to 5.8)	49.8% (-20.2 to 123.7%)	92.8 (57.7 to 203.8)	136.9 (110.4 to 166.5)	47.5% (-27.0 to 130.0%)
Burundi	4.1 (2.1 to 10.9)	4.6 (3.0 to 7.3)	11.5% (-41.6 to 89.7%)	3.4 (1.8 to 8.8)	3.7 (2.4 to 5.8)	8.2% (-42.8 to 80.8%)	100.3 (50.0 to 267.0)	105.8 (68.9 to 169.5)	5.5% (-45.6 to 83.5%)
Comoros	4.2 (2.1 to 6.6)	8.2 (4.8 to 12.5)	95.5% (-4.8 to 322.1%)	3.5 (1.9 to 5.5)	6.5 (3.9 to 9.8)	82.9% (-7.8 to 265.2%)	100.6 (46.1 to 162.3)	185.1 (106.3 to 282.2)	83.9% (-11.1 to 316.0%)
Djibouti	3.8 (2.2 to 5.7)	7.4 (4.2 to 11.2)	93.1% (-1.1 to 244.2%)	3.2 (1.9 to 4.7)	5.9 (3.5 to 8.8)	84.4% (-3.2 to 225.2%)	90.6 (52.5 to 136.3)	166.0 (94.2 to 256.8)	83.3% (-6.6 to 235.2%)
Eritrea	3.0 (1.7 to 5.8)	6.4 (4.0 to 9.7)	111.3% (11.0 to 291.1%)	2.6 (1.4 to 5.0)	5.2 (3.2 to 7.9)	102.4% (4.8 to 272.8%)	75.9 (41.4 to 150.2)	149.0 (92.1 to 230.1)	96.3% (0.1 to 265.1%)
Ethiopia	3.7 (1.5 to 15.2)	5.2 (2.9 to 8.4)	41.8% (-45.9 to 217.2%)	3.1 (1.2 to 12.0)	4.2 (2.3 to 6.8)	35.1% (-47.0 to 193.8%)	90.9 (35.7 to 380.9)	117.4 (62.8 to 192.9)	29.2% (-52.7 to 190.4%)
Kenya	2.7 (1.4 to 4.5)	5.4 (3.9 to 7.3)	101.5% (-0.4 to 270.5%)	2.2 (1.1 to 3.6)	4.7 (3.4 to 6.2)	113.8% (7.8 to 289.5%)	62.3 (32.0 to 102.4)	134.0 (94.6 to 181.1)	115.0% (5.4 to 297.2%)
Madagascar	3.7 (2.3 to 5.8)	4.9 (3.1 to 7.6)	34.3% (-17.1 to 109.3%)	3.0 (1.9 to 4.5)	3.9 (2.4 to 6.0)	32.3% (-16.7 to 105.8%)	88.9 (55.6 to 143.0)	114.6 (70.5 to 177.1)	29.0% (-20.7 to 102.3%)
Malawi	3.3 (2.5 to 4.3)	5.1 (2.9 to 7.8)	54.2% (-25.4 to 165.4%)	2.7 (2.0 to 3.5)	4.0 (2.3 to 6.2)	50.3% (-25.2 to 155.8%)	78.5 (58.3 to 102.6)	114.8 (64.5 to 178.0)	46.2% (-29.8 to 156.3%)
Mozambique	3.8 (2.4 to 5.6)	6.0 (4.1 to 8.5)	88.9% (46.5 to 141.9%)	3.2 (2.1 to 4.6)	4.8 (3.3 to 6.8)	50.5% (-1.9 to 127.2%)	91.4 (58.8 to 134.6)	137.4 (91.0 to 195.2)	50.4% (-5.7 to 131.2%)
Rwanda	4.7 (2.7 to 10.7)	7.9 (5.5 to 10.7)	58.9% (1.0 to 141.9%)	3.9 (2.3 to 8.7)	6.2 (4.4 to 8.3)	57.9% (-37.3 to 196.4%)	115.5 (64.2 to 266.7)	175.1 (121.4 to 241.1)	51.5% (-43.2 to 202.3%)
Somalia	3.2 (1.7 to 6.4)	3.9 (2.3 to 7.2)	20.7% (-23.3 to 83.1%)	2.7 (1.5 to 5.4)	3.3 (2.0 to 6.2)	22.0% (-18.7 to 79.2%)	79.9 (41.3 to 163.4)	95.9 (55.8 to 183.0)	20.0% (-23.3 to 0.8%)
South Sudan	4.0 (2.5 to 6.1)	4.6 (2.9 to 6.6)	16.8% (-29.8 to 73.2%)	3.4 (2.1 to 5.2)	3.9 (2.4 to 5.5)	15.5% (-30.0 to 0.7%)	95.2 (60.0 to 145.9)	108.9 (65.2 to 158.5)	14.3% (-32.7 to 0.8%)
United Republic of Tanzania	4.5 (2.8 to 6.5)	6.8 (5.1 to 8.6)	49.7% (2.4 to 126.9%)	3.7 (2.3 to 5.3)	5.3 (4.1 to 6.6)	43.2% (-1.1 to 111.1%)	106.2 (64.7 to 153.9)	151.7 (112.7 to 194.6)	42.8% (-2.8 to 118.4%)
Uganda	4.7 (3.3 to 6.1)	8.8 (6.0 to 13.1)	87.4% (14.4 to 202.5%)	3.9 (2.8 to 5.1)	6.9 (4.8 to 10.2)	75.8% (9.7 to 178.2%)	109.6 (75.8 to 144.9)	195.2 (130.0 to 291.4)	78.1% (7.4 to 193.8%)
Zambia	5.1 (3.0 to 8.0)	7.5 (5.4 to 10.2)	49.1% (-14.5 to 150.3%)	4.2 (2.5 to 6.5)	5.8 (4.2 to 7.7)	38.9% (-17.8 to 128.5%)	120.9 (72.0 to 195.2)	167.9 (119.0 to 228.7)	38.9% (-21.9 to 138.1%)
**Central sub-Saharan Africa**	2.2 (1.3 to 4.5)	3.2 (2.1 to 5.1)	43.9% (-9.2 to 131.1%)	1.9 (1.1 to 3.7)	2.6 (1.7 to 4.1)	38.5% (-11.4 to 117.6%)	53.1 (31.3 to 110.5)	72.6 (47.2 to 118.8)	36.8% (-13.8 to 121.9%)
Angola	1.9 (1.0 to 4.3)	3.3 (1.8 to 5.5)	73.5% (4.8 to 182.5%)	1.6 (0.8 to 3.5)	2.6 (1.4 to 4.4)	63.9% (0.7 to 162.8%)	46.2 (23.9 to 105.1)	73.4 (41.4 to 124.5)	59.0% (-6.1 to 161.9%)
Central African Republic	2.3 (1.4 to 5.8)	2.7 (1.5 to 5.9)	13.7% (-23.8 to 72.1%)	2.0 (1.2 to 4.7)	2.2 (1.3 to 4.7)	13.3% (-25.0 to 69.9%)	57.4 (33.7 to 146.5)	64.3 (36.4 to 145.1)	12.0% (-25.0 to 71.4%)
Congo	3.2 (2.1 to 6.3)	4.9 (3.1 to 7.7)	54.6% (-8.1 to 149.5%)	2.6 (1.8 to 5.0)	3.9 (2.5 to 5.9)	46.9% (-11.5 to 131.6%)	75.2 (49.9 to 153.0)	108.9 (69.2 to 174.1)	44.8% (-15.0 to 137.8%)
Democratic Republic of the Congo	2.2 (1.2 to 4.4)	3.0 (1.9 to 4.9)	35.8% (-16.0 to 129.0%)	1.8 (1.0 to 3.6)	2.4 (1.5 to 4.0)	32.2% (-17.9 to 120.8%)	52.1 (28.9 to 107.4)	68.0 (41.9 to 113.9)	30.6% (-20.6 to 121.0%)
Equatorial Guinea	1.8 (0.8 to 5.0)	5.1 (3.0 to 8.4)	179.6% (-19.3 to 556.4%)	1.6 (0.7 to 4.2)	3.9 (2.4 to 6.3)	153.1% (-22.6 to 473.2%)	44.9 (20.3 to 127.3)	108.0 (63.2 to 180.2)	140.6% (-32.2 to 478.9%)
Gabon	3.5 (2.3 to 6.4)	6.3 (4.0 to 9.9)	81.0% (-12.0 to 215.3%)	2.9 (1.9 to 5.2)	4.9 (3.2 to 7.5)	70.9% (-15.9 to 185.4%)	80.1 (53.2 to 149.2)	134.6 (85.7 to 213.0)	68.0% (-19.4 to 195.5%)

Data in parentheses are 95% uncertainty intervals. *DALYs* Disability-adjusted life-years, *SDI* Sociodemographic index, *ASIR* Age-standardized incidence rate, *ASDR* Age-standardized death rate, *ASDALY* Age-standardized disability-adjusted life-year

**Fig. 4:**
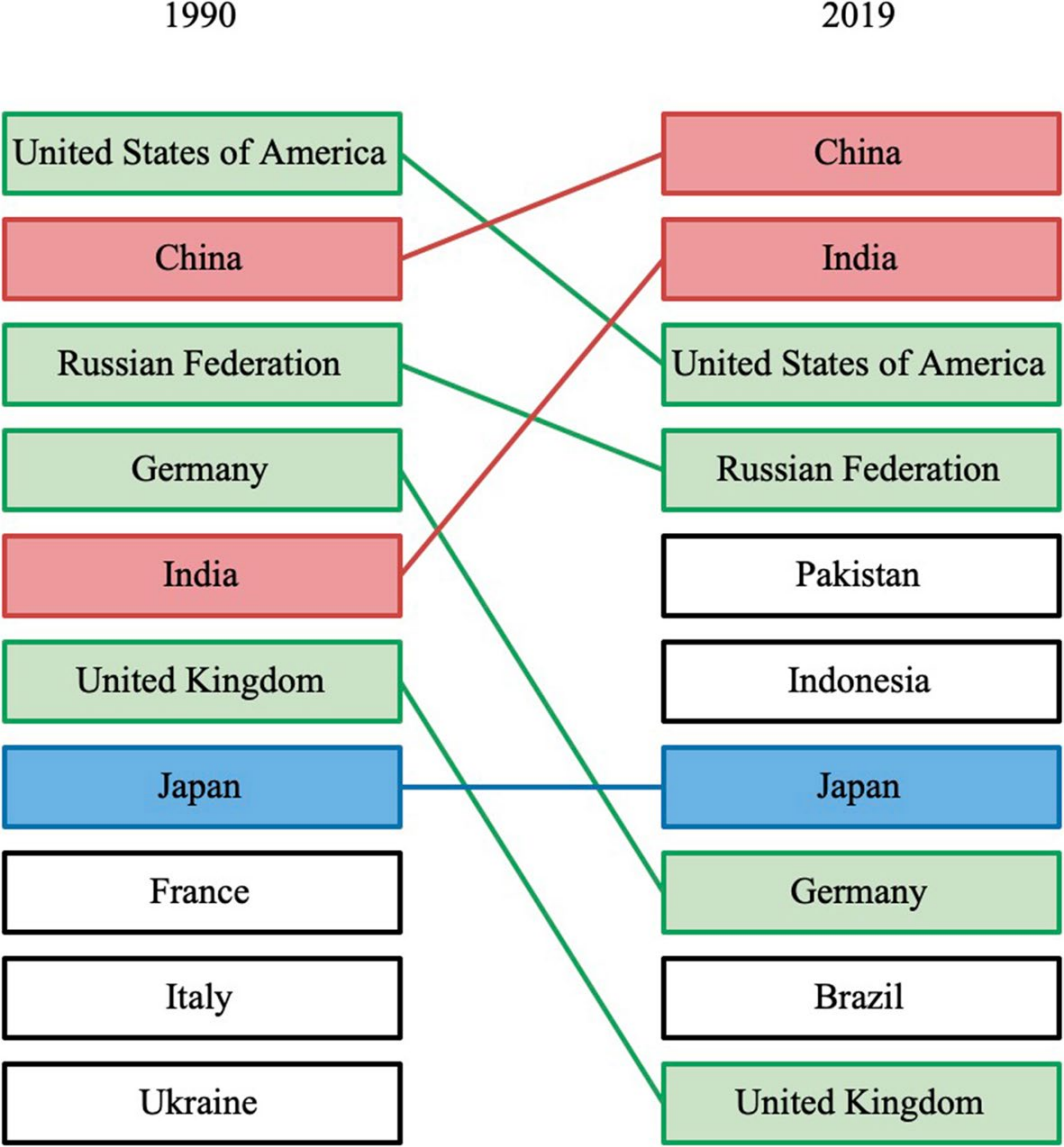
Ranking changes in incident cases by country, 1990–2019

The region with the highest number of incident cases was Western Europe in 1990, while East Asia and South Asia in 2019. And the age-standardized incidence rate of Central Europe was the highest among all world regions, reached 11.7 (10.0 to 13.7), while the rate of Central sub-Saharan Africa seemed lowest at the same time, showed 3.2 (2.1 to 5.1) (Table 2). In both regions, the highest number of incident cases was found in the 50–69 age group (Supplementary Table 1). However, from 1990 to 2019, Caribbean—the third lowest number of morbidity and mortality among all regions in 2019, showed the highest percentage increase in incident cases, with a number of 444.5% (152.5 to 582.7%). At the same time period, Western Europe turned out to be the region with no change (Table 1, Fig. 1, Supplementary Fig. 1a). From 1990 to 2019, United States of America and China were consistently the top countries with the highest number of cases (Table 1, Fig. 4), while Guatemala has seen an incredible increase in the number of cases in 30 years (1150.3% (469.1 to 1652.0%)) (Fig. 1). Focusing on age-standardized incidence rate per 100,000 people, Monaco ranked first both in the past 1990 and 2019, while Pakistan and Brunei Darussalam also climbed rapidly in recent years (Table 2, Supplementary Table 2, Fig. 3).

### Burden of deaths and DALYs

In 2019, the number of deaths for women due to ovarian cancer was 198412 (175357 to 217665), and the number of DALYs was 5.36 million (4.69 to 5.95), compared to 2.73 million (2.49 to 3.17) in 1990, and a percentage of DALYs increased by 96.1% (65.0 to 120.5%) (Table 1). Meanwhile, the percentage change of global age-standardized death rate and DALY rate were relatively flat. The age-standardized death rate due to ovarian cancer was 4.6 (4.0 to 5.0) per 100,000 in 2019 (Table 2). Of these, all three age groups were trending upward in the number of deaths and DALYs, with the highest number of deaths and DALYs in 2019 in the 50–69 age group(Table 1, Fig. 2b-c), but the largest percentage change was in the 70 + age group, with the number of 121.3% (95.7 to 140.3%) (Table 1). For DALYs, only the 15–49 age group in high SDI region showed a decrease in DALYs. And it should be noted that among all regions, the greatest increase in the percentage of DALYs was all observed in the 70 + group, with the highest number being in the low-middle SDI region (384.1% (209.8 to 546.5%)), followed by the middle SDI region (338.9% (215.1 to 431.3%)) (Supplementary Table 3).

From a global perspective, the burden of ovarian cancer reflected in DALYs and mortality was similar to that of morbidity. The high SDI region saw the highest deaths and DALYs in 2019, with the number of 56639 (50391 to 61318) and 1.23 million (1.13 to 1.32), and lowest in the low SDI area, while the high SDI region was the area that DALYs and death burden of which changed least, rising by 15.8% (6.5 to 35.4%) and 30.3% (19.1 to 47.3%). But the area with the largest deaths and DALYs number increase was low-middle SDI quintile with a percentage change of 308.6% (151.3 to 445.8%) and 282.1% (129.6 to 412.9%), respectively (Table 1). As for age-standardized rates of deaths and DALY, from 1990 to 2019, the high SDI region was in a decreasing trend, and with a significant decrease corresponding to 24.0% (-30.3 to -12.3%), and 27.5% (-33.1 to -14.2%), respectively. The same as the burden of incidence, the low-middle SDI quintile showed a striking increase (75.1% (10.1 to 134.4%) for ASDR and 75.1% (6.9 to 134.6%) for ASDALYR) (Table 2).

**Fig. 5:**
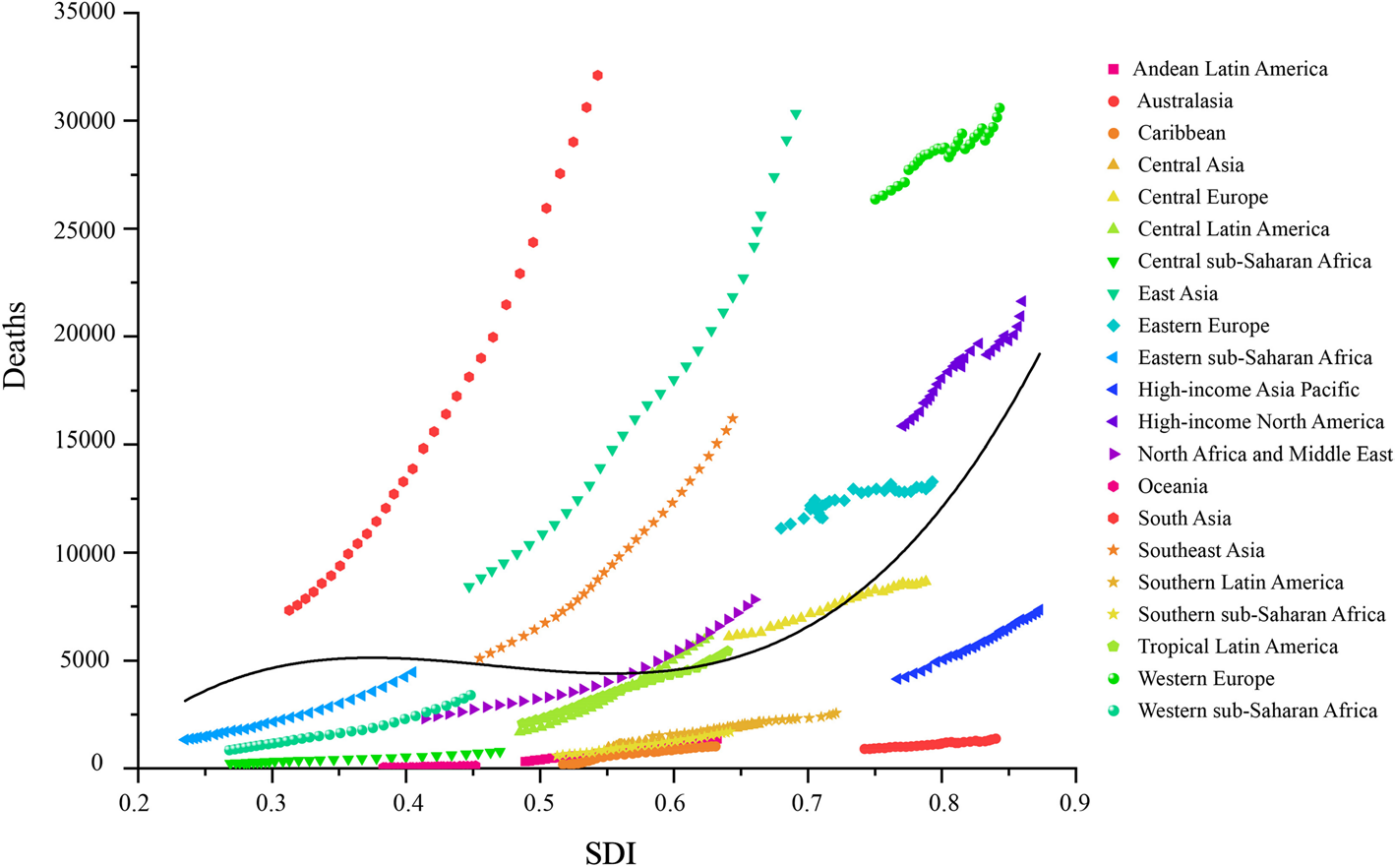
The correlation of ovarian cancer deaths and SDI, 1990–2019. The black line represents the average expected relation-ship between SDIs and deaths for ovarian cancer based on values from all countries from 1990 to 2019. SDI, social-demographic index

In 1990, Western Europe had the highest burden of both deaths and DALYs globally with 26356 (23607 to 27318) and 624896 (553975 to 645271), while in 2019, the region with the highest DALYs was South Asia (32105 (24894 to 39896)) for deaths and 982473 (748576 to 1238008) for DALYs). Central Europe had the highest ASDR (7.6, 6.6 to 8.9) while Central sub-Saharan Africa had the lowest ASDR (2.6, 1.7 to 4.1). Caribbean became the region with the highest percentage increase in both deaths and DALYs during this period, with changes of 432.7% (152.6 to 569.4%) and 388.3% (138.4 to 518.2%), respectively, while Western Europe had the flattest change in values, with little increase of 16.1% (4.9 to 30.2%) and the value of DALY showed no change. (Table 1, Table 2). High-income North America and Central sub-Saharan Africa had the highest and lowest age-standardized DALY rate, respectively. Similar to the results shown in Fig. 5 and Supplementary Fig. 2b, the values of deaths and DALYs in most of the regions showed numerically bigger with the increase of SDIs (Fig. 5, Supplementary Fig. 1b).

**Fig. 6:**
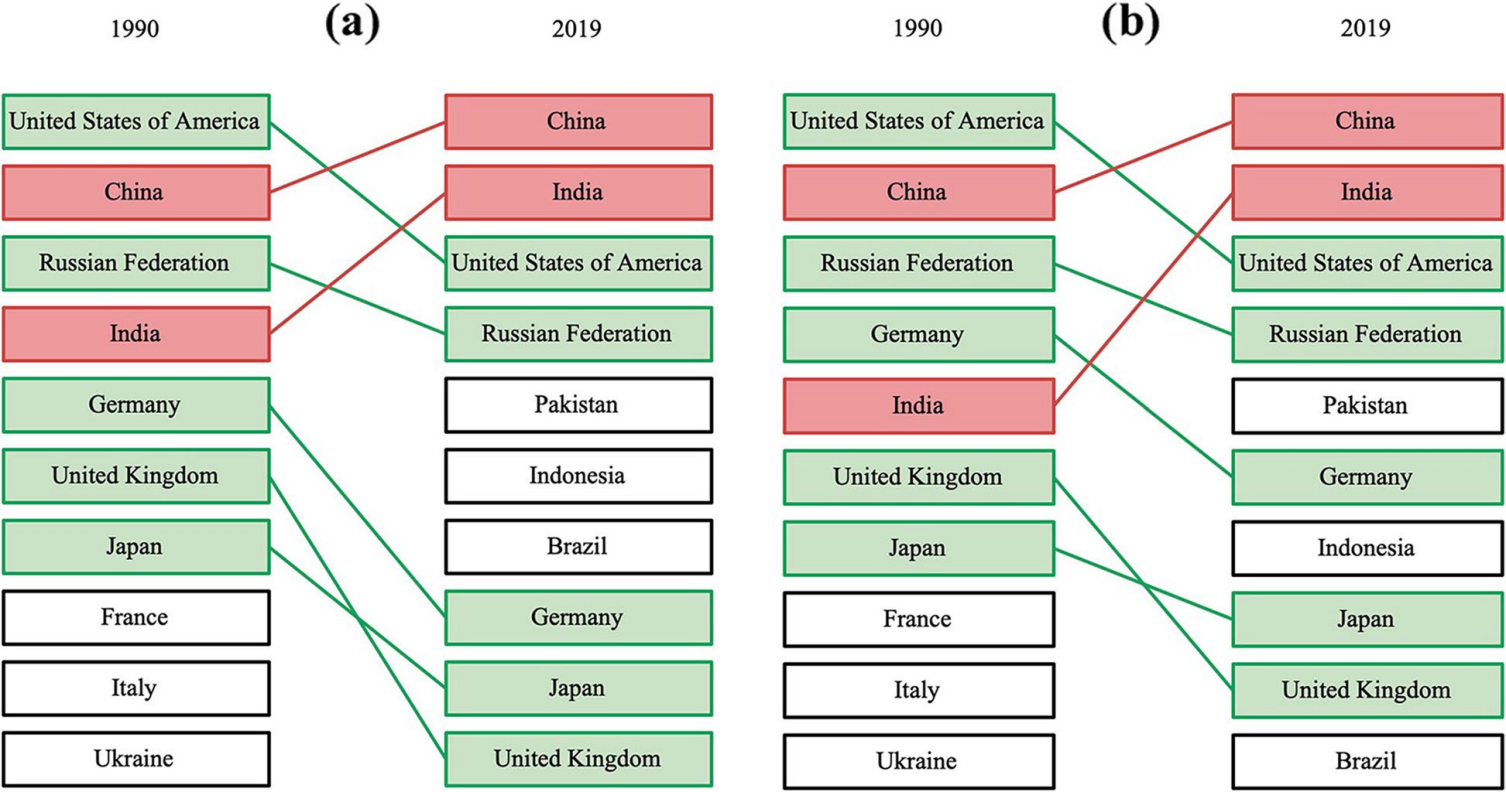
Ranking changes in number of deaths and DALYs by country, 1990–2019: (**a**) Number of DALYs; (**b**)Number of deaths

The same as the trend of cases, top three countries with the highest values of deaths and DALYs for ovarian cancer in 1990 were United States of America, China and Russian Federation, while in 2019, China, India, United States of America became the countries with the highest number, Guatemala had the highest change in the number of deaths among all over the world, United Arab Emirates had the biggest increase in DALYs as the same time (Supplementary Table 2, Fig. 6, Supplementary Fig. 2a-b). Based on an assessment conducted every five years, Monaco consistently ranked first in ASDR. As for ASDALYR, Greenland had the highest value in 1990, and then was overtaken by Monaco in 2005. During this period, the ranking changed a lot, and some countries with lower ASDALYR caught up, such as Pakistan and Brunei Darussalam, but as of 2019, the country with the highest ASDALYR was still Monaco (342.1 (248.9 to 436.0)) per 100,000 (Table 2, Supplementary Fig. 3a-b).

### Burden of ovarian cancer attributable to leading risk factors

In 2019, the ASDR due to all risk factors remained essentially constant from 1990 to 2019, with the ASDALYR increasing from 12.3 (5.8 to 20.7) per 100,000 to 13.9 (5.7 to 25.3) per 100,000. During the period 1990–2000, the global ASDALYR values were relatively stable, and from 2001 a small increase occurred, and then a small decrease followed by a yearly increase from 2010 to 2019 (Supplementary Table 4).

Among the most specific risk factors attributed to all deaths of ovarian cancer globally in 1990, the top three were high fasting plasma glucose, high body-mass index, and occupational exposure to asbestos, respectively. In 2019, the same pattern of risk factors for the number of ovarian cancer deaths worldwide did not change (Supplementary Table 5–6).

**Fig. 7:**
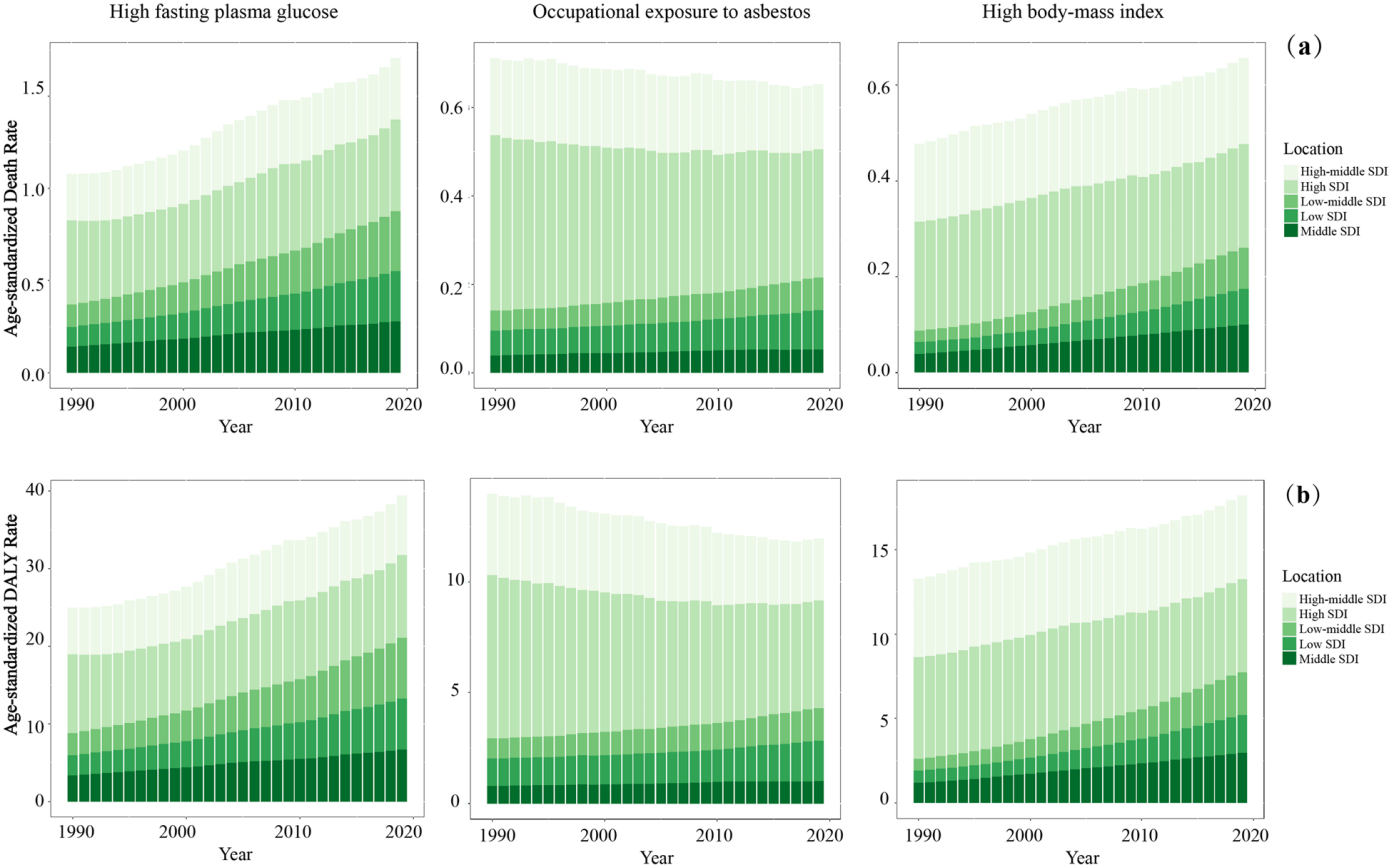
The ovarian cancer ASDR (**a**) and ASDALYR (**b**) attributable to risk factors between 1990 to 2019 by SDI regions. ASDR, age-standardized death rate; ASDALYR, age-standardized disability-adjusted life year rate

In 2019, of all the risk factors for ovarian cancer death, the risk factor that led to the highest number of deaths was high fasting plasma glucose, accounting for 15736 (3023 to 36227) or age-standardized death rate of 0.4 (0.1 to 0.8) per 100000, the corresponding ASDR has shown an increase over the last 30 years (34.7% (18.6 to 51.4%)) (Supplementary Table 6, 7). Among the SDI regions, the numbers of ASDR due to high fasting plasma glucose showed different dynamics. The high SDI region has the highest ASDR but the smallest overall change of 8.9% (0.6 to 27.8%), but low-middle SDI and low SDI regions had the largest ASDR growth of 169.4% (74.6 to 268.1%) and 151.4% (55.3 to 265.3%), respectively (Table 3). In total, the ASDR for all world regions showed an upward trend over the last 30 years (Fig. 7), but Tropical Latin America showed the smallest increase (10.8% (0.1 to 23.8%)), while Caribbean showed the largest increase with 282.3% (66.1 to 388.6%) (Supplementary Table 6).

**Table 3:** Percentage change in number of deaths and age-standardized death rate due to leading risk factors, 1990–2019

	High fasting plasma glucose		Occupational exposure to asbestos		High body-mass index	
	number of deaths	age-standardized rate of death	number of deaths	age-standardized rate of death	number of deaths	age-standardized rate of death
Global	180.9% (144.3 to 218.8%)	34.7% (18.6 to 51.4%)	62.7% (15.2 to 101.2%)	-24.9% (-46.7 to -7.4%)	139.9% (111.5 to 172.5%)	16.4% (2.7 to 32.0%)
High SDI	88.7% (72.7 to 118.5%)	8.9% (0.6 to 27.8%)	34.2% (-4.9 to 70.9%)	-26.8% (-47.9 to -7.1%)	61.0% (45.4 to 91.2%)	-5.0% (-14.1 to 14.5%)
High-middle SDI	148.2% (118.6 to 177.2%)	31.9% (16.2 to 47.1%)	62.4% (10.6 to 111.5%)	-15.8% (-42.7 to 8.9%)	101.8% (80.1 to 127.4%)	10.4% (-1.5 to 24.6%)
Middle SDI	409.7% (284.9 to 509.3%)	98.1% (50.7 to 135.9%)	252.2% (108.4 to 397.8%)	33.8% (-17.7 to 83.8%)	510.3% (357.6 to 695.4%)	152.9% (90.6 to 229.4%)
Low-middle SDI	574.1% (334.2 to 820.8%)	169.4% (74.6 to 268.1%)	328.8% (147.7 to 556.5%)	63.4% (-2.5 to 141.6%)	743.8% (427.0 to 1273.6%)	5.3% (-2.5 to 26.6%)
Low SDI	480.1% (253.7 to 753.6%)	151.4% (55.3 to 265.3%)	252.1% (70.0 to 575.4%)	58.3% (-22.4 to 188.0%)	602.8% (302.5 to 1171.8%)	208.5% (77.9 to 451.5%)

*SDI* Sociodemographic index

Occupational exposure to asbestos was the second leading cause of ovarian cancer deaths globally (Supplementary Table 5–6), with an ASDR of 0.1 (0.1 to 0.2) per 100000, while from 1990 to 2019, value of ASDR caused by this risk factor showed a decreasing trend year by year, and as of 2019, ASDR has decreased by 24.9% (-46.7 to -7.4%). Among all SDI quintiles, only high SDI quintile showed a decreasing trend in ASDR, decreasing by 26.8% (-47.9 to -7.1%), while the changes in other areas were not statistically significant in value (Table 3, Fig. 7, Supplementary Table 6, 8). High body-mass index was the third leading cause of ovarian cancer deaths globally, with an ASDR of 0.1 (0.0 to 0.3) per 100000 (Supplementary Table 6, 9), while from 1990 to 2019, the value showed a slow upward trend with a 16.4% (2.7 to 32.0%) increase (Table 3). However, the middle and low SDI regions showed significant increasing trends, with the number of 152.9% (90.6 to 229.4%) and 208.5% (77.9 to 451.5%), while other regions showed no change (Table 3, Fig. 7).

## Discussion

In this study, we estimated the distribution and trends of the global and regional burden of ovarian cancer from 1990 to 2019 using the latest GBD 2019 data, and explored the latest statistics of ovarian cancer by the major attributable risk factors.

Globally, the total number of incident cases, deaths and DALYs due to ovarian cancer increased significantly. Studies of GBD 2017 showed the same trends of incidence and mortality burden, while one of them didn’t analyze related DALY data, which is a crucial indicator of the dynamics of OC disease burden. Similar with the findings from previous study using 2017 GBD data, the number of cases, deaths and DALYs of ovarian cancer worldwide increased significantly, maintaining the trend shown in the 2017 study [6, 8], but the age-standardized rates did not change. Thus, we speculate the reasons caused this phenomenon were partly owing to the population increase as well as aging. At the same time, relatively stable ASIR could be explained as new risk factors not to introduce or the variation of exposure to risk factors in different parts of the world. Differences in access to health care in different regions of the world, and poor implementation of interventions to address ovarian cancer in some regions, could also contribute to a rise in global ovarian cancer deaths while ASDR remained unchanged. Moreover, the differentiation of OC burden varies greatly among regions. The incidence of OC in high SDI region was much more severe than in other regions, but fortunately, the value in these regions continued to decline. Worryingly, in contrast, we found a significant increase in OC morbidity and mortality in low SDI quintile, although age-standardized morbidity, mortality and DALY rates in these countries remained relatively low. High SDI regions are more severe than other regions, and East Asia has the highest burden of disease. This is associated with social and economic development, maternal number and the decrease of breastfeeding, infertility and the increase in obesity is the promoting factors of ovarian cancer [25–27], but fortunately, with the progress of treatment technology in recent years, and the active involvement of patients in treatment, The mortality rate of OC in these regions continued to decrease. In contrast, we found a significant increase in most developing countries. According to the data of the present study, disease burden of Caribbean showed an extraordinary growth in the last 30 years. Besides the influence of social and economic factors, genetic factors also played a big role. Studies have confirmed that breast and ovarian cancer patients who was born in the Caribbean, one in seven cases of ovarian/breast cancer was inherited [28, 29]. At the same time, young patients have a higher rate of genetic variation and the range of variation is very wide, which may be traced back to the period of European colonization and slave trade.

In this study, we found that the overall burden of ovarian cancer was heaviest in the age group 50–69, while the increase was the largest in the age group 70 + , which was similar to the conclusion of previous studies [29]. Such a result may be closely related to the emergence of aging, which is the same as the reason for the stabilization of age-standardized rates discussed above. For the two main types of epithelial ovarian cancer, patients before the age of 40 mostly belong to type I, which usually appears in the early stage and has a good prognosis. In older patients, type II epithelial neoplasms make up a large proportion, accounting for approximately 75% of it, and are usually present in advanced stages with poor prognosis [30]. At the same time, the elderly has more underlying diseases and more adverse biological factors [31], which may also be the reason for the difference in the incidence and death burden of ovarian cancer in different age groups.

At the same time, of all the risk factors, high fasting plasma glucose led to the most severe burden of ovarian cancer, and the burden changed a lot during the limited time period of this study. Therefore, this study confirms that carrying out effective policies based on local conditions and actively implementing prevention strategies for different risk factors are of great significance for the prevention and treatment of ovarian cancer.

Previous studies suggested that the high mortality and low survival rates of ovarian cancer are mainly due to the lack of screening [32], but a recent randomized controlled trial of the UK Collaborative Trial of Ovarian Cancer Screening (UKCTOCS) [33], which has a high compliance rate among women, showed that the number of deaths among patients who had undergone annual multimodal screening (MMS), and annual transvaginal ultrasound screening (USS) was not significantly higher than those who did not, and could not be considered to have a significant reduction in mortality from ovarian cancer. This shows that future research still has a great potential to select better methods for the detection and treatment of ovarian cancer. At the same time, some previous analysis failed to take full advantage of the different types of data on incidence, mortality, disability-adjusted life years, and risk factors reported in the GBD [7].

If people consume high GI foods for a long time in daily life, they will have a higher risk of high fasting glucose. Some studies have shown that the overall insulin response increases dramatically after eating high GI foods, and insulin has been shown to be a cancer cell growth factor and cancer promoter [34], and like supplying energy to normal cells, ingested glucose can also provide energy to tumor cells, thus promoting tumor growth. Currently, diabetes is now proven to be an independent risk factor for ovarian cancer mortality [35], and age-standardized death rate of ovarian cancer worldwide due to high fasting plasma glucose was on the rise among all SDI quintiles, which may be associated with death due to diabetes comorbidity. And among them, over the past 3 decades, the age-standardized death rate of OC had the smallest rise in the high SDI region, which may be associated with the declining diabetes mortality in women in this region, with data showing a decreasing trend in female diabetes mortality in 24 of 28 EU countries as of 2019 [36]. Similarly, for Eastern Europe and Australasia, high fasting plasma glucose was not the first risk factor for ovarian cancer mortality. In contrast, high fasting plasma glucose led to the largest increase in ASDR in low SDI and low-middle SDI regions. In a nationally representative cross-sectional survey in Iran, researchers found that the prevalence of diabetes and prediabetes increased at an alarming rate among adults, with an age-standardized prevalence rate of 25.4 (18.6 to 32.1) per 100000 [37]. In addition to regional differences in the prevalence of diabetes, unequal distribution of government health resources may also contribute to this situation. In highly developed countries, up to 75% of government health care spending on diabetes is spent on hospital treatment for its complications. In developing countries, however, the structure of diabetes-related spending varies considerably, with most of the costs shifting to patients who must pay for their own treatment [38].

Occupational exposure to asbestos was the second-leading specific risk factor for deaths from ovarian cancer in 2019, contributing to 3.3% (1.5 to 5.4%) of all deaths. Current studies uniformly suggest that human mesothelial cells are highly sensitive to asbestos toxicity [39], but further scientific investigation is needed urgently to clarify the causal relationship between asbestos and ovarian cancer. Ovarian cancer due to asbestos exposure was listed as a new occupational disease in gynecology in 2017. Although asbestos has been banned in 55 countries or regions (e.g. Denmark, the USA) [40], it is still widely used today. High SDI region showed extremely high ASDR values in 2019, but the large decline in the past 3 decades may reflect the industrialization and cumulative occupational exposures of decades ago. For other quintiles with rising ASDR, which may face continued national industrialization in the future, should also introduce appropriate policies, which are not protective against cancer mortality reduction for the time being only by controlling asbestos exposure limits [41], but should ban the use of asbestos or manage the corresponding structures that already contain asbestos.

Our study indicated that high body-mass index was the third-leading specific risk factor for deaths from ovarian cancer in 2019. Numerous studies have now shown a correlation between obesity and ovarian cancer risk, and obesity is also associated with reduced survival rates specific to ovarian cancer at the same time. And in a Women's Health Initiative cohort study, low-fat dietary patterns, and physical activity showed the possibility of a negative association with ovarian cancer risk [42]. This provides an effective solution for ovarian cancer risk avoidance at the individual level.

The accuracy of the results of this study depends on the quality and quantity of GBD data. In terms of quantity, the GBD study cannot cover all regions of the world. In terms of quality, the possibility of missing information in less developed countries cannot be excluded. In addition, information bias is inevitable. Due to the limitation of information, we could not investigate further history to capture the influence of genetic factors on ovarian cancer. Also, different risk factors may have different effects on different histological subtypes of ovarian cancer, and we are currently unable to specifically distinguish between these histological subtypes, and GBD still faces some challenges in estimating the cause-specific non-lethal and lethal burden of ovarian cancer [43]. In order to develop more effective preventive measures, there is a need to analyze the severity grading and subtypes of ovarian cancer according to the etiology in the future.

## Conclusion

GBD 2019 provides a more accurate source of data on ovarian cancer incidence, mortality, and DALYs, and the analysis of these data in this study revealed that the burden of ovarian cancer remains relatively heavy globally, especially in some less developed regions and older population. The findings suggest that ovarian cancer is strongly linked to women's lifestyles and occupational exposure, making it important to reduce related exposure to risk factors for both those women who already have the disease and those who want to prevent it. Currently, reducing the disease burden of ovarian cancer is still focused on primary prevention, and both governments and individuals should pay high attention to the early control of ovarian cancer, starting from both national health resource allocation and targeted prevention and treatment strategies.

## Supplementary Information

**Additional file 1: Supplementary Table 1.** Incident cases and deaths for ovarian cancer by age groups in 1990 and 2019.

**Additional file 2: Supplementary Table 2.** Top 10 countries in the number of cases, deaths, DALYs and age-standardized rates for ovarian cancer, 1990-2019.

**Additional file 3: Supplementary Table 3.** DALYs in 1990 and 2019 and percentage change for ovarian cancer during 1990–2019 by age groups.

**Additional file 4: Supplementary Table 4.** Age-standardized death rate, DALY rate per 100 000 population for all risk factors to ovarian cancer by SDI regions during 1990–2019.

**Additional file 5: Supplementary Table 5.**Leading 3 risk factors of ovarian cancer by deaths at the global and SDI level, 1990 and 2019 for females.

**Additional file 6: Supplementary Table 6.** Deaths in 1990 and 2019, percent of total deaths and percentage change in age-standardized rate per 100,000 population for ovarian cancer during 1990–2019 by top three attributed risk factors.

**Additional file 7: Supplementary Table 7.** Age-standardized death rate per 100 000 population for ovarian cancer due to high fasting plasma glucose by global and SDI regions during 1990–2019.

**Additional file 8: Supplementary Table 8.** Age-standardized death rate per 100 000 population for ovarian cancer due to occupational exposure to asbestos by global and SDI regions during 1990–2019.

**Additional file 9: Supplementary Table 9.** Age-standardized death rate per 100 000 population for ovarian cancer due to high body-mass index by global and SDI regions during 1990–2019.

**Additional file 10: Supplementary Table 10.** Incident cases for ovarian cancer in three age groups by global and SDI regions during 1990–2019.

**Additional file 11: Supplementary Table 11.** Age-standardized incidence rate, death rate and DALY rate per 100 000 population for ovarian cancer by global and SDI regions during 1990–2019.

**Additional file 12: Supplementary Figure 1.** The correlation of ovarian cancer incident cases and SDI (a), the correlation of ovarian cancer DALYs and SDI (b), 1990-2019.

**Additional file 13: Supplementary Figure 2.** Map of percentage change of deaths (a) and DALYs (b) due to ovarian cancer, 1990-2019.

**Additional file 14: Supplementary Figure 3.** Map of age-standardized mortality (a) and DALY (b) rate due to ovarian cancer in 2019.

## Data Availability

The Global Burden of Disease (GBD) Study estimates supporting the conclusions of this article is available in the Institute for Health Metrics and Evaluation (IHME) GBD Results Tool | Global Health Data Exchange, http://ghdx.healthdata.org/gbd-results-tool

## Abbreviations

OC: Ovarian cancer
GBD: Global Burden of Disease
DALYs: Disability-adjusted life years
ASR: Age-standardized rate
UI: Uncertainty interval
CI: Confidence interval
EAPC: Estimated annual percentage change
GHDx: Global Health Data Exchange
SDI: Socio-demographic index
GATHER: Guidelines for Accurate and Transparent Health Estimates Reporting
MIRs: Mortality-to-incidence Ratios
CODEm: Cause of death ensemble model
YLL: Years of life lost
YLD: Years of life disabled
UKCTOCS: UK Collaborative Trial of Ovarian Cancer Screening
MMS: Multimodal screening
USS: Ultrasound screening
