# Supplementary material for: The global burden and associated factors of ovarian cancer in 1990–2019: findings from the Global Burden of Disease Study 2019
Source: BMC Public Health. 2022 Jul 30;22:1455. doi: 10.1186/s12889-022-13861-y (PMC9339194; doi:10.1186/s12889-022-13861-y)
Supplement: Supplementary file 1 — Additional file 1: Supplementary Table 1. Incident cases and deaths for ovarian cancer by age groups in 1990 and 2019. [file 12889_2022_13861_MOESM1_ESM.docx]

Supplementary Table 1. Incident cases and deaths for ovarian cancer by age groups in 1990 and 2019.

|  | 1990 | | | | | |  | 2019 | | | | | |
| --- | --- | --- | --- | --- | --- | --- | --- | --- | --- | --- | --- | --- | --- |
|  | 15–49 years | | 50–69 years | | 70+ years | |  | 15–49 years | 50–69 years | | | 70+ years | |
|  | Incident case | Deaths | Incident case | Deaths | Incident case | Deaths |  | Incident case | Deaths | Incident case | Deaths | Incident case | Deaths |
| **Global** | 39872 (34418 to 48766) | 15139 (12927 to 18976) | 65290 (60965 to 73424) | 47082 (43811 to 53462) | 35083 (32165 to 37477) | 34775 (31793 to 37331) |  | 79672 (68573 to 90845) | 27759 (23923 to 31722) | 135107 (118168 to 151221) | 93173 (81327 to 103382) | 77131 (66946 to 85958) | 76943 (66525 to 84280) |
| **SDI** |  |  |  |  |  |  |  |  |  |  |  |  |  |
| High SDI | 12955 (11660 to 13353) | 4189 (3753 to 4320) | 28008 (25054 to 28813) | 18799 (16831 to 19335) | 21290 (18851 to 22379) | 20436 (18006 to 21516) |  | 12699 (11168 to 14532) | 3473 (3219 to 3783) | 33813 (29810 to 38581) | 20886 (19409 to 22523) | 33741 (28096 to 38375) | 32255 (27017 to 35590) |
| High-middle SDI | 12535 (10591 to 13779) | 4666 (4005 to 5131) | 21957 (20544 to 23428) | 16070 (14989 to 17180) | 8723 (8127 to 9465) | 8969 (8342 to 9742) |  | 19717 (16578 to 22330) | 6353 (5384 to 7107) | 37733 (32039 to 42297) | 25662 (22154 to 28533) | 19512 (16591 to 21838) | 19898 (17184 to 21857) |
| Middle SDI | 9162 (7157 to 12037) | 3729 (2965 to 4841) | 8692 (7605 to 11125) | 6767 (5934 to 8631) | 2922 (2588 to 3740) | 3075 (2727 to 3947) |  | 25734 (21011 to 30453) | 8881 (7332 to 10448) | 36400 (29825 to 42800) | 25545 (20901 to 29977) | 13611 (11163 to 15822) | 13906 (11417 to 16179) |
| Low-middle SDI | 3762 (2782 to 6583) | 1791 (1332 to 3127) | 4649 (3635 to 7341) | 3788 (2964 to 5980) | 1545 (1176 to 2280) | 1646 (1253 to 2408) |  | 15179 (11950 to 19185) | 6201 (4870 to 7968) | 19825 (15987 to 25130) | 15224 (12163 to 19334) | 7820 (6471 to 10044) | 8261 (6799 to 10798) |
| Low SDI | 1442 (936 to 3283) | 757 (490 to 1721) | 1959 (1320 to 3945) | 1639 (1118 to 3294) | 589 (411 to 1002) | 634 (443 to 1068) |  | 6296 (4998 to 7975) | 2835 (2282 to 3608) | 7269 (5962 to 8989) | 5812 (4789 to 7166) | 2408 (2005 to 3078) | 2584 (2150 to 3272) |
| **World region** |  |  |  |  |  |  |  |  |  |  |  |  |  |
| High-income North America | 4778 (4556 to 4958) | 1436 (1356 to 1490) | 9959 (8976 to 10291) | 6570 (5890 to 6779) | 8298 (7322 to 8763) | 7833 (6877 to 8287) |  | 4110 (3236 to 5237) | 1149 (1056 to 1286) | 13325 (10786 to 16395) | 8524 (7947 to 9345) | 12272 (10103 to 14630) | 11948 (10332 to 13206) |
| Southern Latin America | 506 (431 to 612) | 212 (179 to 256) | 909 (787 to 1067) | 698 (608 to 824) | 537 (455 to 655) | 556 (472 to 678) |  | 920 (669 to 1215) | 310 (273 to 358) | 1607 (1228 to 2085) | 1129 (1014 to 1283) | 1082 (860 to 1357) | 1110 (969 to 1281) |
| Western Europe | 6918 (5886 to 7198) | 2083 (1743 to 2160) | 17301 (15050 to 17885) | 11449 (10012 to 11828) | 13062 (11680 to 13702) | 12809 (11455 to 13468) |  | 5922 (5003 to 7071) | 1460 (1347 to 1629) | 16913 (14576 to 19949) | 10150 (9339 to 11072) | 19307 (15837 to 22287) | 18992 (15954 to 21000) |
| Australasia | 281 (251 to 306) | 91 (80 to 98) | 603 (519 to 640) | 411 (351 to 434) | 426 (374 to 459) | 403 (353 to 435) |  | 289 (216 to 386) | 81 (68 to 97) | 781 (606 to 1020) | 494 (424 to 590) | 855 (667 to 1086) | 808 (666 to 958) |
| Central Europe | 2350 (1989 to 2462) | 929 (793 to 969) | 4575 (4365 to 4745) | 3356 (3211 to 3488) | 1767 (1649 to 1884) | 1816 (1688 to 1945) |  | 2170 (1817 to 2583) | 749 (629 to 893) | 5736 (4904 to 6657) | 4022 (3432 to 4709) | 3788 (3198 to 4388) | 3876 (3287 to 4489) |
| High-income Asia Pacific | 2138 (1925 to 2249) | 822 (756 to 851) | 2935 (2801 to 3172) | 1998 (1918 to 2164) | 1504 (1352 to 1645) | 1305 (1175 to 1425) |  | 2421 (1950 to 2924) | 709 (630 to 759) | 4709 (3850 to 5607) | 2679 (2375 to 2864) | 4724 (3493 to 5648) | 3950 (3011 to 4521) |
| Eastern Europe | 3962 (2962 to 4329) | 1497 (1152 to 1640) | 8809 (7616 to 9361) | 6652 (5746 to 7047) | 2783 (2575 to 2992) | 2963 (2742 to 3190) |  | 4694 (3729 to 5755) | 1607 (1277 to 1956) | 10052 (8471 to 12157) | 7245 (5968 to 8709) | 4153 (3533 to 4907) | 4424 (3745 to 5242) |
| Caribbean | 116 (99 to 186) | 48 (40 to 82) | 112 (96 to 175) | 85 (73 to 136) | 54 (46 to 80) | 57 (48 to 83) |  | 470 (354 to 697) | 175 (130 to 273) | 685 (474 to 897) | 487 (340 to 655) | 361 (228 to 444) | 375 (238 to 460) |
| South Asia | 3456 (2356 to 5479) | 1724 (1178 to 2716) | 4737 (3495 to 6909) | 3898 (2848 to 5688) | 1514 (1132 to 2186) | 1615 (1207 to 2335) |  | 15263 (11146 to 19839) | 6460 (4739 to 8450) | 21083 (15785 to 26348) | 16355 (12191 to 20950) | 8588 (6777 to 11282) | 9085 (6998 to 12296) |
| Central Latin America | 1136 (1094 to 1180) | 449 (433 to 468) | 1005 (966 to 1097) | 780 (750 to 853) | 417 (385 to 449) | 440 (406 to 472) |  | 3671 (2952 to 4510) | 1251 (1000 to 1513) | 4154 (3458 to 5059) | 2953 (2460 to 3528) | 1845 (1538 to 2199) | 1913 (1593 to 2255) |
| Andean Latin America | 197 (153 to 301) | 88 (68 to 134) | 180 (141 to 258) | 144 (114 to 209) | 75 (59 to 108) | 82 (64 to 117) |  | 824 (574 to 1103) | 287 (202 to 387) | 858 (604 to 1090) | 625 (440 to 793) | 422 (284 to 532) | 448 (301 to 566) |
| Southern Sub-Saharan Africa | 305 (239 to 414) | 132 (106 to 177) | 336 (270 to 420) | 268 (214 to 332) | 164 (127 to 198) | 177 (137 to 215) |  | 717 (563 to 923) | 296 (237 to 380) | 1062 (844 to 1279) | 825 (659 to 993) | 521 (414 to 603) | 560 (440 to 652) |
| Central Asia | 501 (413 to 569) | 191 (158 to 219) | 712 (604 to 801) | 539 (456 to 610) | 211 (169 to 244) | 219 (175 to 253) |  | 1145 (975 to 1311) | 428 (366 to 490) | 1611 (1390 to 1825) | 1185 (1024 to 1338) | 409 (337 to 464) | 425 (351 to 482) |
| Tropical Latin America | 1226 (1146 to 1285) | 501 (469 to 527) | 1303 (1232 to 1364) | 1016 (962 to 1062) | 533 (485 to 566) | 557 (506 to 593) |  | 2578 (2329 to 2844) | 912 (827 to 998) | 3563 (3267 to 3902) | 2583 (2370 to 2816) | 1842 (1593 to 2041) | 1927 (1662 to 2133) |
| Central Sub-Saharan Africa | 106 (59 to 254) | 55 (31 to 131) | 167 (94 to 353) | 140 (80 to 293) | 41 (23 to 76) | 44 (24 to 80) |  | 405 (254 to 678) | 190 (120 to 324) | 532 (340 to 898) | 432 (277 to 727) | 157 (90 to 239) | 171 (97 to 261) |
| Southeast Asia | 3578 (2590 to 5831) | 1429 (1044 to 2286) | 3403 (2820 to 4846) | 2579 (2138 to 3668) | 1016 (844 to 1295) | 1040 (854 to 1320) |  | 11271 (8347 to 15443) | 3748 (2747 to 5298) | 12514 (9801 to 17368) | 8434 (6560 to 12022) | 4021 (3215 to 5601) | 3941 (3160 to 5516) |
| North Africa and Middle East | 1150 (729 to 2289) | 510 (326 to 1038) | 1550 (1116 to 2821) | 1225 (885 to 2245) | 504 (354 to 793) | 532 (373 to 841) |  | 4933 (3726 to 5886) | 1708 (1305 to 2054) | 5693 (4654 to 6743) | 4008 (3310 to 4757) | 2051 (1685 to 2452) | 2071 (1717 to 2471) |
| East Asia | 6153 (4523 to 8375) | 2428 (1834 to 3310) | 5318 (4058 to 8150) | 4122 (3109 to 6209) | 1704 (1350 to 2624) | 1820 (1447 to 2809) |  | 13510 (10248 to 17809) | 4300 (3222 to 5604) | 25041 (17922 to 31517) | 16863 (11892 to 21531) | 9104 (6324 to 11132) | 9156 (6524 to 11288) |
| Western Sub-Saharan Africa | 363 (268 to 520) | 181 (135 to 256) | 524 (385 to 773) | 436 (327 to 630) | 207 (149 to 299) | 221 (161 to 319) |  | 1780 (1273 to 2560) | 777 (555 to 1100) | 2324 (1615 to 3219) | 1842 (1296 to 2518) | 693 (454 to 957) | 743 (480 to 1016) |
| Oceania | 21 (14 to 49) | 9 (6 to 22) | 21 (14 to 41) | 16 (11 to 32) | 6 (4 to 10) | 6 (4 to 10) |  | 84 (51 to 181) | 33 (20 to 72) | 77 (51 to 136) | 55 (37 to 100) | 21 (15 to 32) | 21 (15 to 32) |
| Eastern Sub-Saharan Africa | 632 (371 to 1597) | 325 (188 to 837) | 837 (519 to 1850) | 703 (435 to 1522) | 261 (170 to 447) | 280 (183 to 484) |  | 2496 (1881 to 3164) | 1131 (868 to 1438) | 2790 (2225 to 3371) | 2284 (1825 to 2761) | 916 (751 to 1080) | 999 (825 to 1166) |

Data in parentheses are 95% uncertainty intervals. SDI=Sociodemographic index.
