# Supplementary material for: The global burden and associated factors of ovarian cancer in 1990–2019: findings from the Global Burden of Disease Study 2019
Source: BMC Public Health. 2022 Jul 30;22:1455. doi: 10.1186/s12889-022-13861-y (PMC9339194; doi:10.1186/s12889-022-13861-y)
Supplement: Supplementary file 2 — Additional file 2: Supplementary Table 2. Top 10 countries in the number of cases, deaths, DALYs and age-standardized rates for ovarian cancer, 1990-2019. [file 12889_2022_13861_MOESM2_ESM.docx]

Supplementary Table 2. Top 10 countries in the number of cases, deaths, DALYs and age-standardized rates for ovarian cancer, 1990-2019.

| Ranking by cases | 1990 | | | 1995 | | | 2000 | | | 2005 | | | 2010 | | | 2015 | | | 2019 | | |
| --- | --- | --- | --- | --- | --- | --- | --- | --- | --- | --- | --- | --- | --- | --- | --- | --- | --- | --- | --- | --- | --- |
|  | Cases | Deaths | DALYs | Cases | Deaths | DALYs | Cases | Deaths | DALYs | Cases | Deaths | DALYs | Cases | Deaths | DALYs | Cases | Deaths | DALYs | Cases | Deaths | DALYs |
| 1 | United States of America | United States of America | United States of America | United States of America | United States of America | United States of America | United States of America | United States of America | China | China | United States of America | China | China | China | China | China | China | China | China | China | China |
| 2 | China | China | China | China | China | China | China | China | United States of America | United States of America | China | United States of America | United States of America | United States of America | India | India | India | India | India | India | India |
| 3 | Russian Federation | Russian Federation | Russian Federation | Russian Federation | Russian Federation | Russian Federation | India | India | India | India | India | India | India | India | United States of America | United States of America | United States of America | United States of America | United States of America | United States of America | United States of America |
| 4 | Germany | Germany | India | Germany | Germany | India | Russian Federation | Russian Federation | Russian Federation | Russian Federation | Russian Federation | Russian Federation | Russian Federation | Russian Federation | Russian Federation | Russian Federation | Russian Federation | Russian Federation | Russian Federation | Russian Federation | Russian Federation |
| 5 | India | India | Germany | India | India | Germany | Germany | Germany | Germany | Germany | Germany | Germany | Germany | Germany | Indonesia | Japan | Germany | Pakistan | Pakistan | Pakistan | Pakistan |
| 6 | United Kingdom | United Kingdom | United Kingdom | United Kingdom | United Kingdom | Japan | Japan | United Kingdom | Japan | Japan | United Kingdom | Japan | Japan | Japan | Pakistan | Indonesia | Pakistan | Indonesia | Indonesia | Germany | Indonesia |
| 7 | Japan | Japan | Japan | Japan | Japan | United Kingdom | United Kingdom | Japan | United Kingdom | United Kingdom | Japan | Indonesia | Indonesia | United Kingdom | Germany | Germany | Japan | Brazil | Japan | Indonesia | Brazil |
| 8 | France | France | France | France | France | France | France | France | Indonesia | Indonesia | France | United Kingdom | United Kingdom | Indonesia | Japan | Pakistan | Indonesia | Germany | Germany | Japan | Germany |
| 9 | Italy | Italy | Italy | Italy | Italy | Italy | Italy | Italy | Brazil | Italy | Italy | Pakistan | Brazil | France | Brazil | United Kingdom | United Kingdom | Japan | Brazil | United Kingdom | Japan |
| 10 | Ukraine | Ukraine | Ukraine | Indonesia | Ukraine | Indonesia | Indonesia | Brazil | France | France | Brazil | Brazil | Pakistan | Pakistan | United Kingdom | Brazil | Brazil | United Kingdom | United Kingdom | Brazil | United Kingdom |
| Ranking rate per 100 000 population |  |  |  |  |  |  |  |  |  |  |  |  |  |  |  |  |  |  |  |  |  |
| 1 | Monaco | Monaco | Greenland | Monaco | Monaco | Greenland | Monaco | Monaco | Monaco | Monaco | Monaco | Monaco | Monaco | Monaco | Monaco | Monaco | Monaco | Monaco | Monaco | Monaco | Pakistan |
| 2 | Greenland | Greenland | Monaco | Greenland | Greenland | Monaco | Greenland | Greenland | Greenland | Greenland | Greenland | Greenland | Brunei Darussalam | Greenland | Lithuania | Brunei Darussalam | Pakistan | Pakistan | Brunei Darussalam | Pakistan | Monaco |
| 3 | United Kingdom | Luxembourg | Lithuania | Denmark | Denmark | Lithuania | Denmark | Denmark | Denmark | American Samoa | American Samoa | Lithuania | American Samoa | Lithuania | Greenland | American Samoa | Brunei Darussalam | Brunei Darussalam | Pakistan | Brunei Darussalam | Brunei Darussalam |
| 4 | Luxembourg | United Kingdom | United Kingdom | United Kingdom | Lithuania | Denmark | United Kingdom | Lithuania | Lithuania | Czechia | Denmark | American Samoa | Greenland | Latvia | Latvia | Seychelles | American Samoa | American Samoa | Seychelles | American Samoa | American Samoa |
| 5 | Ireland | Ireland | Ireland | Ireland | Latvia | Latvia | Ireland | United Kingdom | Poland | Ireland | Lithuania | Latvia | Croatia | Brunei Darussalam | Pakistan | Pakistan | Greenland | Greenland | American Samoa | United States Virgin Islands | Seychelles |
| 6 | Austria | Lithuania | Luxembourg | Czechia | Ireland | Estonia | Czechia | Ireland | Latvia | United Kingdom | Poland | Poland | Seychelles | American Samoa | Brunei Darussalam | United States Virgin Islands | United States Virgin Islands | Lithuania | United States Virgin Islands | Greenland | United States Virgin Islands |
| 7 | Netherlands | Belgium | Latvia | Luxembourg | United Kingdom | Poland | Luxembourg | Poland | Estonia | Denmark | Latvia | Denmark | Lithuania | United States Virgin Islands | American Samoa | Greenland | Lithuania | United States Virgin Islands | Greenland | Latvia | Greenland |
| 8 | Belgium | Netherlands | Poland | Lithuania | Luxembourg | Ireland | Norway | Latvia | United Kingdom | Norway | Ireland | Brunei Darussalam | Ireland | Poland | Poland | Croatia | Poland | Seychelles | United Kingdom | Lithuania | Latvia |
| 9 | Croatia | Estonia | Estonia | Norway | Estonia | Czechia | Lithuania | Estonia | Ireland | Poland | United Kingdom | United States Virgin Islands | United States Virgin Islands | Pakistan | United States Virgin Islands | Ireland | Latvia | Latvia | Samoa | Poland | Lithuania |
| 10 | Norway | Austria | Czechia | Iceland | Czechia | United Kingdom | Iceland | Czechia | Czechia | Croatia | United States Virgin Islands | Czechia | Norway | Denmark | Guyana | United Kingdom | Seychelles | Poland | Ireland | Seychelles | Guyana |

Percentages indicate proportions of the global count that are accounted for by the top ten countries. Cases = number of incident cases. DALYs= disability-adjusted life-years.
