# Supplementary material for: The global burden and associated factors of ovarian cancer in 1990–2019: findings from the Global Burden of Disease Study 2019
Source: BMC Public Health. 2022 Jul 30;22:1455. doi: 10.1186/s12889-022-13861-y (PMC9339194; doi:10.1186/s12889-022-13861-y)
Supplement: Supplementary file 3 — Additional file 3: Supplementary Table 3. DALYs in 1990 and 2019 and percentage change for ovarian cancer during 1990–2019 by age groups. [file 12889_2022_13861_MOESM3_ESM.docx]

Supplementary Table 3. DALYs in 1990 and 2019 and percentage change for ovarian cancer during 1990–2019 by age groups.

|  | 15-49 years | | |  | 50-69 years | | |  | 70+ years | | |
| --- | --- | --- | --- | --- | --- | --- | --- | --- | --- | --- | --- |
|  | 1990 | 2019 | Percentage change 1990 and 2019 |  | 1990 | 2019 | Percentage change 1990 and 2019 |  | 1990 | 2019 | Percentage change 1990 and 2019 |
| Global | 760870 (645441 to 960213) | 1379355 (1183284 to 1574162) | 81.3% (37.8% to 115.7%) |  | 1420153 (1321775 to 1614600) | 2823174 (2459433 to 3132271) | 98.8% (67.9% to 124.4%) |  | 522102 (482096 to 560730) | 1113871 (975511 to 1221853) | 113.3% (87.7% to 132.6%) |
| SDI regions |  |  |  |  |  |  |  |  |  |  |  |
| High SDI | 204846 (184898 to 211647) | 169954 (157411 to 185595) | -17.0% (-23.7% to 1.6%) |  | 555887 (496380 to 572109) | 618330 (574506 to 668025) | 11.2% (2.4% to 34.0%) |  | 297613 (263956 to 312454) | 438771 (377894 to 480325) | 47.4% (34.3% to 60.9%) |
| High-middle SDI | 233422 (199887 to 256571) | 311015 (263965 to 348154) | 33.2% (12.6% to 55.6%) |  | 486575 (452729 to 521218) | 772167 (666130 to 856803) | 58.7% (37.2% to 76.7%) |  | 138479 (129375 to 150083) | 290673 (249478 to 319640) | 109.9% (82.4% to 131.4%) |
| Middle SDI | 193115 (152327 to 251638) | 442640 (365051 to 516315) | 129.2% (51.8% to 186.0%) |  | 209442 (183533 to 267212) | 784886 (643613 to 922999) | 274.8% (167.5% to 357.2%) |  | 48688 (42882 to 63100) | 213674 (175831 to 248035) | 338.9% (215.1% to 431.3%) |
| Low-middle SDI | 90996 (67214 to 160326) | 311602 (245964 to 397105) | 242.4% (86.4% to 383.5%) |  | 116912 (91288 to 185628) | 466845 (370804 to 594484) | 299.3% (154.5% to 447.6%) |  | 26655 (20335 to 39523) | 129036 (106298 to 167305) | 384.1% (209.8% to 546.5%) |
| Low SDI | 38171 (24697 to 87111) | 143327 (115635 to 181753) | 275.5% (98.6% to 481.4%) |  | 50787 (34514 to 102922) | 179563 (148054 to 221677) | 253.6% (105.9% to 415.9%) |  | 10446 (7296 to 17801) | 41169 (34289 to 52399) | 294.1% (154.2% to 448.9%) |
| High-income North America | 70550 (66844 to 73284) | 56178 (51532 to 62613) | -20.4% (-28.2% to -6.9%) |  | 192413 (173839 to 198940) | 249762 (232646 to 274152) | 29.8% (19.6% to 59.8%) |  | 115410 (102216 to 121522) | 166213 (146020 to 182712) | 44.0% (31.9% to 66.3%) |
| Canada | 7277 (6630 to 7819) | 5354 (4300 to 6667) | -26.4% (-41.9% to -5.2%) |  | 18659 (17161 to 19888) | 24603 (20438 to 29898) | 31.9% (7.8% to 65.2%) |  | 9519 (8603 to 10300) | 16345 (12939 to 19895) | 71.7% (35.9% to 113.3%) |
| Greenland | 20 (12 to 27) | 16 (9 to 23) | -18.1% (-48.2% to 22.7%) |  | 39 (30 to 50) | 58 (40 to 77) | 48.2% (-3.8% to 112.1%) |  | 9 (6 to 15) | 14 (9 to 22) | 58.7% (2.1% to 137.3%) |
| United States of America | 63251 (59990 to 65747) | 50807 (46727 to 56950) | -19.7% (-27.3% to -5.4%) |  | 173710 (156323 to 179482) | 225097 (210839 to 248554) | 29.6% (19.7% to 63.1%) |  | 105880 (93628 to 111725) | 149852 (132324 to 164217) | 41.5% (30.0% to 66.5%) |
| Australasia | 4494.5 (3967.9 to 4846.9) | 3980.3 (3374.9 to 4759.5) | -11.4% (-28.1% to 21.4%) |  | 12081 (10319 to 12754) | 14568 (12529 to 17447) | 20.6% (0.5% to 75.4%) |  | 6089 (5344 to 6569) | 11013 (9160 to 12930) | 80.9% (51.7% to 124.0%) |
| Australia | 3660 (3192 to 3982) | 3217 (2657 to 3948) | -12.1% (-29.7% to 23.3%) |  | 9829 (8408 to 10472) | 11752 (9849 to 14208) | 19.6% (-2.5% to 76.8%) |  | 4968 (4353 to 5387) | 9234 (7611 to 11006) | 85.9% (53.9% to 134.2%) |
| New Zealand | 834 (715 to 944) | 763 (637 to 898) | -8.5% (-27.1% to 15.4%) |  | 2251 (1944 to 2472) | 2817 (2416 to 3305) | 25.1% (3.3% to 67.9%) |  | 1121 (969 to 1262) | 1779 (1455 to 2074) | 58.6% (30.1% to 92.1%) |
| High-income Asia Pacific | 40151 (36901 to 41682) | 34142 (30295 to 36630) | -15.0% (-24.2% to -7.4%) |  | 62054 (59552 to 66983) | 81855 (72455 to 87451) | 31.9% (11.3% to 42.0%) |  | 19565 (17879 to 21373) | 51433 (40427 to 57969) | 162.9% (120.0% to 192.8%) |
| Brunei Darussalam | 75 (51 to 120) | 212 (159 to 282) | 182.8% (64.3% to 337.2%) |  | 63 (45 to 96) | 294 (228 to 365) | 368.2% (191.2% to 590.4%) |  | 14 (10 to 20) | 53 (39 to 67) | 281.7% (143.5% to 492.8%) |
| Japan | 34208 (29029 to 35509) | 25203 (22677 to 27091) | -26.3% (-34.3% to -9.0%) |  | 55693 (53560 to 57786) | 59175 (53951 to 63334) | 6.3% (-3.7% to 13.1%) |  | 18157 (16437 to 19228) | 42518 (33307 to 48438) | 134.2% (97.9% to 158.9%) |
| Singapore | 780 (682 to 884) | 1010 (788 to 1275) | 29.5% (-1.1% to 65.5%) |  | 1034 (913 to 1168) | 2651 (2257 to 3088) | 156.4% (110.4% to 211.0%) |  | 259 (221 to 299) | 808 (635 to 965) | 211.4% (146.7% to 292.9%) |
| Republic of Korea | 5088 (4398 to 7290) | 7717 (5495 to 9322) | 51.7% (-36.1% to 95.2%) |  | 5265 (4305 to 9747) | 19735 (10454 to 23871) | 274.8% (-7.8% to 418.6%) |  | 1135 (852 to 2233) | 8054 (4887 to 10109) | 609.5% (90.3% to 973.9%) |
| Western Europe | 101603 (85817 to 105716) | 71381 (65870 to 79943) | -29.7% (-36.4% to -6.6%) |  | 337535 (291773 to 348742) | 300617 (276139 to 328396) | -10.9% (-19.2% to 13.4%) |  | 184521 (166506 to 193458) | 253500 (216901 to 278676) | 37.4% (24.2% to 49.9%) |
| Andorra | 8 (6 to 13) | 14 (10 to 20) | 68.7% (3.7% to 169.2%) |  | 18 (13 to 29) | 43 (29 to 59) | 132.0% (26.2% to 271.3%) |  | 7 (5 to 12) | 20 (13 to 28) | 169.3% (48.5% to 355.2%) |
| Austria | 2389 (1856 to 2660) | 1323 (1059 to 2130) | -44.6% (-56.9% to 19.9%) |  | 8023 (6279 to 8664) | 6022 (5091 to 9164) | -24.9% (-37.9% to 50.1%) |  | 4860 (3761 to 5262) | 4671 (3909 to 5513) | -3.9% (-19.6% to 46.0%) |
| Belgium | 2806 (2395 to 3126) | 1622 (1310 to 2164) | -42.2% (-55.5% to -10.3%) |  | 10514 (8883 to 11387) | 7387 (6210 to 9871) | -29.7% (-43.3% to 15.2%) |  | 6207 (5117 to 6849) | 6668 (5518 to 7753) | 7.4% (-11.7% to 30.6%) |
| Cyprus | 129 (97 to 182) | 253 (168 to 336) | 96.9% (10.3% to 203.7%) |  | 385 (287 to 567) | 769 (571 to 969) | 99.6% (11.5% to 199.7%) |  | 190 (134 to 305) | 484 (320 to 640) | 154.4% (17.5% to 323.4%) |
| Denmark | 1531 (1312 to 1715) | 978 (814 to 1201) | -36.1% (-49.2% to -16.9%) |  | 5145 (4730 to 5568) | 4963 (4265 to 5885) | -3.6% (-18.6% to 17.0%) |  | 2737 (2492 to 2988) | 4248 (3548 to 5058) | 55.2% (28.7% to 87.0%) |
| Finland | 1302 (1159 to 1453) | 783 (634 to 977) | -39.9% (-53.1% to -19.9%) |  | 4055 (3692 to 4347) | 4031 (3414 to 4814) | -0.6% (-17.7% to 34.2%) |  | 2453 (2205 to 2666) | 4096 (3372 to 4838) | 67.0% (40.6% to 97.4%) |
| France | 14230 (11675 to 15442) | 9355 (7716 to 12037) | -34.3% (-47.6% to 9.2%) |  | 44361 (38374 to 47443) | 43214 (36520 to 51487) | -2.6% (-18.7% to 28.9%) |  | 25645 (22295 to 27739) | 37345 (29641 to 44123) | 45.6% (19.4% to 73.0%) |
| Germany | 21521 (17704 to 23280) | 12276 (10498 to 14525) | -43.0% (-52.6% to -20.5%) |  | 83608 (68449 to 89123) | 63664 (55279 to 73332) | -23.9% (-35.2% to 1.9%) |  | 48316 (39380 to 52315) | 56579 (46097 to 65851) | 17.1% (-0.5% to 36.9%) |
| Greece | 2238 (2019 to 2476) | 2222 (1880 to 2644) | -0.7% (-17.6% to 24.6%) |  | 6930 (6374 to 7811) | 7350 (6421 to 8600) | 6.1% (-8.2% to 21.7%) |  | 3227 (2911 to 3533) | 6046 (4994 to 6900) | 87.3% (56.2% to 119.4%) |
| Iceland | 61 (53 to 71) | 55 (44 to 71) | -9.9% (-31.3% to 22.3%) |  | 177 (151 to 203) | 178 (146 to 241) | 0.8% (-21.2% to 44.8%) |  | 94 (79 to 109) | 112 (87 to 142) | 19.5% (-7.4% to 67.1%) |
| Ireland | 989 (853 to 1131) | 982 (742 to 1287) | -0.7% (-29.1% to 37.6%) |  | 3106 (2769 to 3424) | 3790 (2950 to 4631) | 22.0% (-6.3% to 58.3%) |  | 1487 (1322 to 1706) | 2327 (1769 to 2879) | 56.5% (6.5% to 107.0%) |
| Israel | 1029 (876 to 1162) | 1390 (1159 to 1652) | 35.1% (8.1% to 73.6%) |  | 2928 (2346 to 3213) | 4509 (3860 to 5187) | 54.0% (29.6% to 100.3%) |  | 1335 (1150 to 1486) | 2721 (2285 to 3204) | 103.8% (69.7% to 146.8%) |
| Italy | 14238 (11485 to 15031) | 11639 (10531 to 12755) | -18.3% (-26.6% to -5.9%) |  | 44248 (42206 to 45872) | 41968 (38561 to 45558) | -5.2% (-13.1% to 5.0%) |  | 20892 (19643 to 23483) | 34523 (29001 to 38798) | 65.2% (45.1% to 82.0%) |
| Luxembourg | 122 (106 to 139) | 135 (109 to 175) | 10.6% (-14.0% to 45.1%) |  | 427 (377 to 483) | 424 (341 to 574) | -0.9% (-23.5% to 36.3%) |  | 242 (211 to 275) | 306 (234 to 382) | 26.5% (-5.2% to 63.2%) |
| Malta | 101 (86 to 116) | 85 (68 to 107) | -15.3% (-35.6% to 9.8%) |  | 291 (246 to 336) | 350 (284 to 435) | 20.2% (-7.1% to 59.2%) |  | 137 (108 to 160) | 279 (216 to 350) | 104.3% (55.7% to 168.0%) |
| Monaco | 14 (10 to 19) | 14 (10 to 20) | 1.6% (-31.3% to 55.4%) |  | 50 (34 to 71) | 63 (46 to 83) | 26.3% (-19.3% to 95.4%) |  | 41 (29 to 56) | 59 (41 to 77) | 42.7% (-7.0% to 113.7%) |
| Netherlands | 4468 (3927 to 4853) | 2667 (2257 to 3187) | -40.3% (-50.5% to -25.4%) |  | 13433 (11565 to 14357) | 13043 (11363 to 14866) | -2.9% (-16.1% to 17.9%) |  | 7564 (6660 to 8182) | 11781 (9481 to 13602) | 55.8% (30.7% to 80.9%) |
| Norway | 1313 (1232 to 1384) | 1060 (944 to 1199) | -19.3% (-29.4% to -5.2%) |  | 3869 (3622 to 4039) | 4241 (3847 to 4773) | 9.6% (-1.8% to 35.0%) |  | 2561 (2299 to 2708) | 3029 (2614 to 3383) | 18.3% (5.9% to 36.0%) |
| Portugal | 2127 (1541 to 2404) | 1567 (1281 to 1892) | -26.3% (-42.2% to 18.7%) |  | 4779 (4392 to 5288) | 5150 (4350 to 6421) | 7.7% (-10.3% to 31.5%) |  | 2143 (1923 to 2633) | 3801 (3143 to 4759) | 77.3% (46.9% to 111.2%) |
| San Marino | 2 (2 to 3) | 6 (3 to 9) | 128.3% (17.0% to 334.4%) |  | 9 (6 to 12) | 15 (8 to 24) | 70.0% (-7.6% to 199.0%) |  | 6 (4 to 8) | 11 (7 to 17) | 90.2% (14.4% to 197.6%) |
| Spain | 8269 (6509 to 8986) | 8106 (6732 to 9628) | -2.0% (-18.6% to 19.6%) |  | 23052 (19141 to 24699) | 25976 (22071 to 29717) | 12.7% (-4.5% to 31.3%) |  | 10008 (8887 to 11016) | 18053 (14301 to 21102) | 80.4% (49.9% to 111.8%) |
| Sweden | 2576 (2082 to 2862) | 1466 (1278 to 1781) | -43.1% (-52.2% to -13.0%) |  | 8493 (6489 to 9123) | 6569 (5863 to 8323) | -22.6% (-32.8% to 33.9%) |  | 5492 (4736 to 6011) | 5869 (5044 to 6995) | 6.9% (-7.5% to 47.6%) |
| Switzerland | 984 (868 to 1152) | 1093 (902 to 1336) | 11.1% (-12.9% to 39.9%) |  | 3293 (3021 to 3619) | 4701 (4040 to 5343) | 42.8% (20.1% to 67.8%) |  | 2249 (1985 to 2478) | 4388 (3187 to 5171) | 95.1% (51.0% to 132.7%) |
| United Kingdom | 19070 (16491 to 19910) | 12225 (11356 to 13808) | -35.9% (-41.6% to -16.5%) |  | 66060 (55923 to 68478) | 51935 (48469 to 58105) | -21.4% (-28.1% to 4.0%) |  | 36476 (33579 to 38108) | 45865 (40805 to 50070) | 25.7% (15.6% to 35.8%) |
| Southern Latin America | 10479 (8896 to 12742) | 15343 (13551 to 17596) | 46.4% (14.8% to 83.6%) |  | 20817 (18120 to 24518) | 33734 (30302 to 38209) | 62.1% (32.0% to 102.4%) |  | 8541 (7224 to 10450) | 16127 (14188 to 18527) | 88.8% (48.4% to 144.3%) |
| Argentina | 7236 (5961 to 9099) | 11055 (9545 to 12861) | 52.8% (17.7% to 96.9%) |  | 15207 (12709 to 18325) | 22788 (19768 to 26658) | 49.8% (17.5% to 93.2%) |  | 6309 (5114 to 7938) | 11240 (9625 to 13438) | 78.2% (34.5% to 140.6%) |
| Chile | 2544 (2159 to 3026) | 3429 (2876 to 4135) | 34.8% (-1.0% to 80.7%) |  | 3941 (3461 to 4463) | 8707 (7456 to 10073) | 120.9% (76.7% to 176.7%) |  | 1443 (1239 to 1663) | 3559 (3009 to 4278) | 146.6% (96.3% to 225.0%) |
| Uruguay | 698 (581 to 837) | 859 (721 to 1019) | 23.1% (-6.2% to 57.8%) |  | 1667 (1421 to 1957) | 2238 (1915 to 2578) | 34.2% (7.5% to 68.1%) |  | 789 (661 to 941) | 1328 (1110 to 1563) | 68.3% (30.6% to 110.5%) |
| Eastern Europe | 73669 (56199 to 80670) | 77788 (61870 to 94615) | 5.6% (-13.1% to 46.8%) |  | 201584 (172724 to 213492) | 215469 (178970 to 258732) | 6.9% (-9.9% to 35.2%) |  | 47170 (43827 to 50471) | 66681 (56659 to 78861) | 41.4% (22.5% to 64.5%) |
| Belarus | 2529 (2172 to 2918) | 2862 (1827 to 3934) | 13.2% (-24.2% to 58.9%) |  | 10247 (8071 to 11451) | 8692 (5981 to 11968) | -15.2% (-41.7% to 24.4%) |  | 2342 (2031 to 2689) | 2458 (1731 to 3284) | 5.0% (-26.3% to 45.2%) |
| Estonia | 532 (456 to 619) | 304 (200 to 539) | -42.9% (-62.8% to -7.8%) |  | 1978 (1672 to 2195) | 1222 (862 to 1806) | -38.2% (-56.5% to 6.4%) |  | 677 (467 to 762) | 922 (696 to 1186) | 36.2% (-0.1% to 88.7%) |
| Latvia | 1158 (1009 to 1313) | 714 (455 to 1041) | -38.4% (-59.9% to -9.7%) |  | 3473 (3076 to 3830) | 2601 (1838 to 3591) | -25.1% (-47.9% to 5.7%) |  | 947 (830 to 1060) | 1469 (1105 to 1925) | 55.1% (14.4% to 104.7%) |
| Lithuania | 1537 (1345 to 1729) | 1056 (751 to 1420) | -31.3% (-51.2% to -5.3%) |  | 4607 (4139 to 5010) | 3793 (2907 to 4836) | -17.7% (-37.8% to 9.6%) |  | 1305 (1102 to 1442) | 2019 (1601 to 2470) | 54.8% (21.4% to 96.5%) |
| Republic of Moldova | 1322 (719 to 1536) | 976 (702 to 1243) | -26.2% (-46.2% to 19.8%) |  | 2756 (1873 to 3108) | 2602 (1978 to 3210) | -5.6% (-28.6% to 38.6%) |  | 376 (311 to 445) | 539 (413 to 702) | 43.5% (9.0% to 87.9%) |
| Russian Federation | 50279 (37061 to 54078) | 52445 (38890 to 67810) | 4.3% (-19.9% to 59.1%) |  | 136301 (113704 to 142612) | 151109 (118299 to 190587) | 10.9% (-11.2% to 49.5%) |  | 32314 (30330 to 33982) | 47915 (39210 to 58303) | 48.3% (23.2% to 80.1%) |
| Ukraine | 16311 (11762 to 20052) | 19430 (13836 to 25817) | 19.1% (-16.9% to 78.8%) |  | 42222 (34343 to 49994) | 45449 (34436 to 58502) | 7.6% (-22.0% to 46.2%) |  | 9210 (7349 to 12194) | 11358 (8804 to 14860) | 23.3% (-9.5% to 67.9%) |
| Central Europe | 45352 (38449 to 47365) | 35865 (30146 to 42781) | -20.9% (-33.5% to -3.9%) |  | 100898 (95939 to 104894) | 117871 (100497 to 137659) | 16.8% (0.6% to 36.6%) |  | 27656 (25894 to 29643) | 56947 (48688 to 65917) | 105.9% (77.7% to 136.6%) |
| Albania | 350 (217 to 427) | 413 (250 to 625) | 18.1% (-24.6% to 73.7%) |  | 328 (269 to 453) | 895 (593 to 1295) | 172.6% (80.4% to 299.2%) |  | 96 (74 to 148) | 340 (237 to 508) | 253.8% (142.9% to 417.2%) |
| Bosnia and Herzegovina | 984 (824 to 1140) | 1046 (717 to 1486) | 6.3% (-26.8% to 48.0%) |  | 2196 (1884 to 2850) | 3597 (2582 to 4890) | 63.8% (11.5% to 123.2%) |  | 390 (311 to 591) | 1358 (1037 to 1800) | 248.2% (136.0% to 394.3%) |
| Bulgaria | 2997 (2660 to 3368) | 2999 (1988 to 4105) | 0.1% (-32.7% to 41.6%) |  | 6529 (5875 to 7363) | 7550 (5358 to 9959) | 15.6% (-22.6% to 55.0%) |  | 1442 (1287 to 1762) | 3436 (2606 to 4437) | 138.3% (63.5% to 217.7%) |
| Croatia | 1725 (1463 to 1978) | 999 (684 to 1425) | -42.1% (-61.6% to -13.1%) |  | 4852 (4244 to 5455) | 3968 (2888 to 5291) | -18.2% (-40.5% to 13.0%) |  | 1625 (1335 to 1847) | 2330 (1767 to 3008) | 43.4% (7.5% to 89.0%) |
| Czechia | 4062 (3623 to 4425) | 2685 (1946 to 3710) | -33.9% (-53.4% to -4.1%) |  | 10603 (8801 to 11317) | 9859 (7599 to 12797) | -7.0% (-29.0% to 25.2%) |  | 3870 (3370 to 4202) | 6321 (5008 to 7837) | 63.4% (28.9% to 104.6%) |
| Hungary | 3854 (3495 to 4241) | 2862 (2128 to 3869) | -25.7% (-45.8% to 2.0%) |  | 9841 (9094 to 10739) | 9607 (7618 to 12374) | -2.4% (-23.3% to 24.5%) |  | 3913 (3463 to 4260) | 5828 (4691 to 7182) | 48.9% (20.5% to 83.9%) |
| Montenegro | 145 (106 to 187) | 165 (117 to 224) | 13.8% (-22.5% to 53.9%) |  | 288 (219 to 407) | 454 (326 to 639) | 57.8% (10.9% to 113.0%) |  | 77 (56 to 110) | 169 (120 to 235) | 119.4% (56.2% to 218.8%) |
| North Macedonia | 531 (396 to 635) | 711 (471 to 1010) | 33.8% (-10.5% to 89.8%) |  | 1004 (835 to 1191) | 1803 (1265 to 2414) | 79.6% (24.8% to 144.8%) |  | 185 (146 to 270) | 621 (464 to 811) | 236.1% (123.1% to 386.7%) |
| Poland | 17753 (14936 to 18550) | 13145 (9817 to 17227) | -26.0% (-45.3% to 1.1%) |  | 35733 (33792 to 37107) | 46895 (35947 to 60241) | 31.2% (-0.4% to 68.7%) |  | 10113 (9423 to 10861) | 21897 (17137 to 27315) | 116.5% (71.8% to 165.7%) |
| Romania | 8056 (5474 to 8828) | 6062 (4569 to 7900) | -24.7% (-44.0% to 1.9%) |  | 16531 (15039 to 17783) | 17393 (13543 to 21965) | 5.2% (-18.6% to 34.8%) |  | 2738 (2403 to 4333) | 7521 (6033 to 9123) | 174.7% (77.3% to 244.9%) |
| Serbia | 2732 (2127 to 3400) | 2810 (1911 to 3871) | 2.9% (-34.6% to 55.0%) |  | 7058 (5682 to 8957) | 9351 (6809 to 12326) | 32.5% (-13.3% to 87.6%) |  | 1434 (1017 to 1964) | 3948 (2943 to 5075) | 175.4% (78.4% to 315.6%) |
| Slovakia | 1501 (1202 to 1763) | 1497 (1010 to 2102) | -0.3% (-37.5% to 43.1%) |  | 3829 (2671 to 4400) | 4685 (3156 to 6551) | 22.4% (-18.9% to 75.1%) |  | 1061 (837 to 1230) | 2099 (1352 to 2925) | 97.8% (28.5% to 175.1%) |
| Slovenia | 663 (474 to 929) | 472 (319 to 690) | -28.7% (-57.6% to 17.1%) |  | 2106 (1524 to 2817) | 1815 (1287 to 2493) | -13.8% (-44.9% to 34.7%) |  | 712 (529 to 898) | 1080 (799 to 1417) | 51.6% (1.6% to 118.8%) |
| Central Asia | 9905 (8208 to 11242) | 21325 (18186 to 24425) | 115.3% (76.7% to 162.3%) |  | 16567 (14033 to 18759) | 36397 (31333 to 41138) | 119.7% (82.3% to 166.7%) |  | 3442 (2762 to 3999) | 6625 (5445 to 7524) | 92.5% (57.0% to 137.4%) |
| Armenia | 587 (440 to 895) | 681 (518 to 890) | 16.0% (-33.2% to 74.7%) |  | 1263 (966 to 1814) | 1922 (1485 to 2414) | 52.1% (-1.9% to 120.5%) |  | 276 (203 to 362) | 616 (484 to 755) | 122.9% (55.4% to 213.5%) |
| Azerbaijan | 895 (614 to 1239) | 2438 (1422 to 3514) | 172.3% (51.7% to 313.8%) |  | 1216 (826 to 1686) | 3818 (2370 to 5356) | 214.0% (94.4% to 381.3%) |  | 198 (111 to 305) | 475 (305 to 702) | 140.3% (58.6% to 335.4%) |
| Georgia | 829 (668 to 1087) | 1078 (787 to 1376) | 30.1% (-20.1% to 83.4%) |  | 1930 (1566 to 2498) | 3525 (2229 to 4463) | 82.6% (0.9% to 150.5%) |  | 420 (339 to 518) | 1055 (643 to 1311) | 151.4% (29.3% to 240.8%) |
| Kazakhstan | 4170 (2998 to 5005) | 6271 (4879 to 7828) | 50.4% (13.8% to 99.4%) |  | 7325 (5891 to 8754) | 11983 (9763 to 14485) | 63.6% (27.5% to 115.6%) |  | 1430 (1112 to 1760) | 2312 (1864 to 2796) | 61.7% (25.7% to 112.5%) |
| Kyrgyzstan | 593 (504 to 702) | 1393 (1067 to 1728) | 134.7% (67.8% to 213.0%) |  | 1234 (1032 to 1453) | 2438 (1846 to 2980) | 97.7% (49.7% to 158.1%) |  | 274 (219 to 333) | 360 (269 to 451) | 31.3% (-1.8% to 74.1%) |
| Mongolia | 235 (148 to 433) | 860 (556 to 1348) | 266.7% (112.4% to 495.4%) |  | 257 (160 to 482) | 1111 (774 to 1572) | 331.9% (146.2% to 627.6%) |  | 56 (34 to 105) | 153 (106 to 212) | 170.4% (55.9% to 348.9%) |
| Tajikistan | 397 (258 to 691) | 1356 (908 to 2177) | 241.2% (87.7% to 457.8%) |  | 762 (511 to 1074) | 2106 (1585 to 2732) | 176.3% (81.0% to 340.5%) |  | 231 (101 to 371) | 327 (166 to 470) | 41.3% (-9.9% to 207.8%) |
| Turkmenistan | 638 (391 to 742) | 1243 (834 to 1683) | 94.8% (32.2% to 181.4%) |  | 574 (475 to 662) | 1254 (872 to 1663) | 118.4% (52.0% to 211.5%) |  | 91 (73 to 109) | 155 (111 to 208) | 70.4% (19.9% to 140.1%) |
| Uzbekistan | 1561 (904 to 1992) | 6007 (4500 to 7546) | 284.7% (167.5% to 513.7%) |  | 2005 (1054 to 2619) | 8239 (6297 to 10146) | 310.9% (181.5% to 600.0%) |  | 466 (180 to 636) | 1173 (843 to 1459) | 151.8% (54.2% to 453.3%) |
| Central Latin America | 23489 (22639 to 24464) | 62952 (50691 to 76289) | 168.0% (115.7% to 223.6%) |  | 23850 (22941 to 26221) | 90569 (75194 to 108531) | 279.7% (209.5% to 352.2%) |  | 6720 (6209 to 7292) | 28364 (23891 to 33672) | 322.1% (254.5% to 393.0%) |
| Colombia | 6323 (5770 to 6868) | 11899 (8482 to 17130) | 88.2% (32.7% to 168.1%) |  | 7339 (6792 to 7926) | 19333 (14327 to 26523) | 163.4% (94.2% to 267.6%) |  | 1920 (1712 to 2129) | 6613 (4924 to 9133) | 244.4% (158.0% to 366.2%) |
| Costa Rica | 252 (218 to 318) | 837 (592 to 1151) | 231.7% (116.1% to 368.7%) |  | 319 (279 to 365) | 1514 (1096 to 2029) | 375.1% (235.3% to 546.8%) |  | 104 (88 to 120) | 613 (408 to 806) | 491.0% (285.6% to 692.9%) |
| El Salvador | 383 (317 to 525) | 1396 (809 to 2021) | 264.1% (80.3% to 466.2%) |  | 402 (324 to 626) | 1836 (1190 to 2553) | 356.9% (114.6% to 601.6%) |  | 112 (84 to 190) | 666 (449 to 889) | 493.0% (171.6% to 815.1%) |
| Guatemala | 331 (261 to 407) | 2644 (1595 to 3660) | 697.6% (366.7% to 1084.3%) |  | 275 (221 to 360) | 3025 (1747 to 3983) | 1000.8% (373.1% to 1471.7%) |  | 39 (30 to 81) | 854 (560 to 1088) | 2093.9% (540.2% to 3072.4%) |
| Honduras | 379 (271 to 637) | 1585 (606 to 3666) | 318.7% (71.7% to 647.2%) |  | 396 (286 to 566) | 1988 (960 to 4674) | 402.1% (166.3% to 952.5%) |  | 102 (69 to 146) | 622 (303 to 1347) | 507.0% (228.8% to 1297.0%) |
| Mexico | 14264 (13751 to 14782) | 35678 (27710 to 44716) | 150.1% (95.1% to 215.0%) |  | 13808 (13300 to 15107) | 50132 (40266 to 62107) | 263.1% (181.8% to 351.2%) |  | 4056 (3733 to 4306) | 14768 (12243 to 18055) | 264.1% (199.9% to 335.8%) |
| Nicaragua | 255 (185 to 390) | 1007 (680 to 1336) | 294.8% (109.0% to 504.9%) |  | 201 (130 to 278) | 1307 (873 to 1754) | 551.7% (253.0% to 1008.8%) |  | 58 (27 to 82) | 444 (251 to 585) | 669.6% (295.0% to 1743.3%) |
| Panama | 221 (188 to 268) | 721 (500 to 1001) | 226.6% (117.9% to 379.0%) |  | 250 (217 to 291) | 1119 (805 to 1490) | 346.7% (213.8% to 520.3%) |  | 90 (75 to 107) | 464 (323 to 612) | 416.0% (249.6% to 611.0%) |
| Venezuela | 1081 (922 to 1610) | 7185 (4059 to 10131) | 564.6% (132.0% to 888.4%) |  | 861 (721 to 1693) | 10316 (6084 to 14265) | 1098.3% (251.3% to 1712.5%) |  | 238 (192 to 498) | 3320 (1947 to 4404) | 1292.4% (272.3% to 1945.1%) |
| Andean Latin America | 4525 (3502 to 6959) | 14602 (10241 to 19597) | 222.7% (89.3% to 382.6%) |  | 4442 (3485 to 6375) | 19101 (13596 to 24415) | 330.0% (168.6% to 515.4%) |  | 1221 (945 to 1756) | 6435 (4331 to 8171) | 427.1% (199.5% to 668.2%) |
| Bolivia | 601 (360 to 1388) | 2265 (1418 to 3517) | 277.1% (99.9% to 583.7%) |  | 701 (420 to 1700) | 3157 (2039 to 5316) | 350.5% (149.9% to 640.0%) |  | 191 (107 to 463) | 1021 (660 to 1833) | 433.5% (204.4% to 818.7%) |
| Ecuador | 745 (582 to 1228) | 4015 (2450 to 5596) | 438.9% (128.1% to 765.9%) |  | 653 (495 to 1157) | 5297 (3140 to 7245) | 711.2% (209.5% to 1208.9%) |  | 167 (115 to 319) | 1826 (996 to 2458) | 994.0% (259.5% to 1767.0%) |
| Peru | 3179 (2192 to 4881) | 8322 (5426 to 12288) | 161.8% (37.7% to 340.1%) |  | 3088 (2343 to 4080) | 10647 (7277 to 14839) | 244.7% (109.3% to 434.7%) |  | 862 (646 to 1139) | 3588 (2427 to 5026) | 316.0% (147.8% to 553.3%) |
| Caribbean | 2423 (2028 to 4216) | 8718 (6504 to 13760) | 259.8% (95.9% to 367.2%) |  | 2618 (2231 to 4182) | 14915 (10390 to 20087) | 469.7% (165.5% to 639.2%) |  | 859 (734 to 1244) | 5418 (3454 to 6675) | 530.7% (168.3% to 697.5%) |
| Antigua and  Barbuda | 4 (3 to 8) | 24 (17 to 31) | 498.4% (107.0% to 747.0%) |  | 5 (4 to 9) | 55 (32 to 70) | 1071.4% (257.4% to 1559.8%) |  | 3 (2 to 5) | 19 (10 to 24) | 667.7% (107.5% to 968.7%) |
| Bahamas | 34 (29 to 51) | 179 (111 to 240) | 421.4% (117.9% to 642.5%) |  | 31 (26 to 50) | 248 (173 to 323) | 707.4% (253.6% to 1017.9%) |  | 9 (7 to 17) | 64 (47 to 81) | 594.9% (177.0% to 863.7%) |
| Barbados | 18 (15 to 31) | 92 (45 to 121) | 410.8% (40.3% to 620.9%) |  | 24 (20 to 43) | 230 (118 to 294) | 869.8% (160.7% to 1244.0%) |  | 15 (13 to 27) | 106 (50 to 132) | 586.7% (83.6% to 833.7%) |
| Belize | 7 (6 to 8) | 59 (41 to 74) | 760.9% (448.8% to 1033.4%) |  | 5 (4 to 8) | 65 (44 to 81) | 1115.4% (479.1% to 1543.3%) |  | 1 (1 to 3) | 14 (10 to 17) | 850.2% (260.0% to 1234.2%) |
| Bermuda | 19 (14 to 26) | 17 (12 to 23) | -8.8% (-41.9% to 36.9%) |  | 32 (25 to 44) | 48 (36 to 63) | 49.0% (-7.0% to 116.8%) |  | 16 (13 to 21) | 33 (25 to 43) | 103.6% (35.6% to 192.6%) |
| Cuba | 762 (655 to 1089) | 2306 (1400 to 3085) | 202.7% (33.7% to 323.6%) |  | 854 (734 to 1405) | 5444 (3040 to 6985) | 537.9% (124.1% to 777.4%) |  | 263 (214 to 442) | 1927 (1017 to 2441) | 632.1% (123.7% to 904.9%) |
| Dominica | 5 (4 to 6) | 12 (6 to 17) | 155.0% (16.3% to 300.7%) |  | 5 (4 to 8) | 18 (10 to 25) | 262.0% (43.7% to 504.0%) |  | 3 (2 to 5) | 9 (5 to 12) | 244.8% (9.1% to 494.5%) |
| Dominican  Republic | 354 (275 to 476) | 1043 (637 to 1575) | 194.2% (81.3% to 351.5%) |  | 278 (208 to 423) | 1139 (729 to 1809) | 309.5% (154.2% to 544.6%) |  | 75 (54 to 121) | 367 (234 to 562) | 390.7% (213.4% to 688.5%) |
| Grenada | 6 (5 to 11) | 36 (24 to 45) | 485.2% (120.9% to 708.8%) |  | 9 (7 to 14) | 66 (41 to 82) | 659.2% (181.4% to 942.2%) |  | 4 (3 to 8) | 23 (15 to 29) | 461.4% (83.4% to 673.6%) |
| Guyana | 71 (55 to 132) | 371 (232 to 522) | 419.3% (71.7% to 710.6%) |  | 69 (54 to 121) | 456 (306 to 629) | 558.6% (153.4% to 916.7%) |  | 17 (13 to 35) | 91 (66 to 119) | 424.6% (110.9% to 684.9%) |
| Haiti | 515 (236 to 2290) | 2088 (1019 to 6916) | 305.5% (116.5% to 681.9%) |  | 482 (241 to 1889) | 2090 (1035 to 6464) | 333.7% (104.2% to 774.7%) |  | 102 (50 to 344) | 439 (222 to 1253) | 331.5% (94.7% to 735.8%) |
| Jamaica | 116 (96 to 195) | 746 (450 to 1017) | 544.8% (131.2% to 848.2%) |  | 154 (129 to 235) | 1199 (680 to 1613) | 680.7% (177.7% to 1018.7%) |  | 67 (55 to 108) | 424 (245 to 543) | 533.2% (116.2% to 784.0%) |
| Puerto Rico | 277 (235 to 436) | 633 (437 to 881) | 128.6% (28.5% to 239.6%) |  | 399 (342 to 564) | 1783 (1241 to 2369) | 347.1% (136.3% to 519.2%) |  | 190 (161 to 233) | 1166 (757 to 1517) | 512.5% (219.1% to 743.9%) |
| Saint Kitts and Nevis | 4 (3 to 5) | 16 (5 to 26) | 319.7% (47.2% to 655.9%) |  | 6 (5 to 8) | 42 (28 to 55) | 671.7% (268.5% to 976.1%) |  | 3 (3 to 5) | 10 (6 to 13) | 220.4% (25.9% to 337.4%) |
| Saint Lucia | 11 (9 to 16) | 66 (46 to 86) | 527.1% (186.3% to 789.1%) |  | 13 (11 to 19) | 97 (65 to 126) | 654.2% (232.3% to 950%) |  | 5 (4 to 8) | 34 (22 to 43) | 586.7% (172.1% to 829.8%) |
| Saint Vincent  and the Grenadines | 7 (6 to 12) | 38 (26 to 48) | 414.6% (120.6% to 600.7%) |  | 9 (7 to 16) | 57 (43 to 70) | 571.9% (176.7% to 823.4%) |  | 4 (3 to 8) | 20 (14 to 24) | 408.9% (85.1% to 593.6%) |
| Suriname | 31 (21 to 53) | 190 (96 to 263) | 512.2% (113.4% to 955.8%) |  | 39 (29 to 69) | 292 (156 to 392) | 646.2% (169.0% to 1111.1%) |  | 10 (7 to 19) | 94 (49 to 130) | 815.3% (198.0% to 1454.0%) |
| Trinidad and  Tobago | 74 (62 to 132) | 468 (214 to 692) | 535.1% (61.2% to 910.4%) |  | 82 (70 to 134) | 953 (397 to 1349) | 1067.2% (193.7% to 1661.3%) |  | 30 (25 to 51) | 322 (139 to 435) | 969.3% (165.1% to 1432.5%) |
| United States Virgin Islands | 28 (20 to 47) | 40 (27 to 58) | 42.0% (-23.2% to 140.7%) |  | 38 (28 to 56) | 125 (89 to 163) | 233.6% (93.6% to 405.3%) |  | 12 (9 to 17) | 70 (46 to 94) | 494.1% (220.9% to 832.9%) |
| Tropical Latin America | 25792 (24148 to 27116) | 45741 (41450 to 50209) | 77.4% (58.7% to 98.4%) |  | 30971 (29372 to 32489) | 78370 (72141 to 85250) | 153.0% (130.0% to 179.2%) |  | 8764 (8030 to 9317) | 28425 (24865 to 31325) | 224.3% (190.6% to 260.3%) |
| Brazil | 25513 (23859 to 26836) | 44632 (40553 to 48951) | 74.9% (56.4% to 96.6%) |  | 30692 (29106 to 32183) | 76811 (70804 to 83701) | 150.3% (127.4% to 176.9%) |  | 8677 (7947 to 9221) | 27869 (24421 to 30733) | 221.2% (188.8% to 258.0%) |
| Paraguay | 279 (212 to 355) | 1110 (611 to 1585) | 297.6% (106.8% to 503.8%) |  | 279 (213 to 371) | 1559 (870 to 2206) | 459.6% (164.8% to 805.5%) |  | 87 (64 to 122) | 556 (289 to 778) | 540.3% (158.5% to 954.5%) |
| East Asia | 126298 (95178 to 171835) | 210617 (158692 to 271764) | 66.8% (4.5% to 147.8%) |  | 127954 (97085 to 190746) | 515530 (369448 to 654369) | 302.9% (123.8% to 498.4%) |  | 29108 (23056 to 45786) | 142891 (100175 to 176838) | 390.9% (164.8% to 603.6%) |
| China | 121082 (90513 to 164525) | 200304 (146751 to 262304) | 65.4% (2.5% to 150.0%) |  | 121338 (91110 to 182532) | 495322 (351305 to 631507) | 308.2% (123.2% to 509.7%) |  | 27771 (21913 to 44251) | 136926 (94190 to 170874) | 393.1% (159.7% to 614.6%) |
| Democratic People's Republic of Korea | 2420 (1121 to 5530) | 4477 (2645 to 7745) | 85.0% (-7.8% to 311.8%) |  | 3366 (1658 to 7011) | 7892 (5029 to 13445) | 134.4% (28.0% to 407.8%) |  | 552 (262 to 1065) | 2212 (1477 to 3133) | 301.0% (120.0% to 680.4%) |
| Taiwan (province of China) | 2796 (2538 to 3040) | 5836 (4056 to 7985) | 108.7% (43.9% to 186.2%) |  | 3250 (2978 to 3546) | 12316 (8765 to 16996) | 278.9% (162.5% to 428.3%) |  | 785 (701 to 871) | 3753 (2699 to 4858) | 377.8% (238.4% to 528.1%) |
| Southeast Asia | 73669 (53346 to 118335) | 188507 (138918 to 263802) | 155.9% (35.2% to 238.8%) |  | 80646 (66773 to 114761) | 263180 (205864 to 374773) | 226.3% (112.0% to 318.8%) |  | 16472 (13555 to 21205) | 60517 (48715 to 85474) | 267.4% (163.1% to 371.7%) |
| Cambodia | 1193 (524 to 3917) | 4598 (2678 to 7917) | 285.3% (69.7% to 673.3%) |  | 1300 (698 to 3526) | 6432 (4214 to 11273) | 394.6% (148.7% to 925.2%) |  | 213 (118 to 468) | 1335 (907 to 2227) | 527.6% (225.4% to 1448.1%) |
| Indonesia | 29259 (18326 to 59227) | 81026 (48262 to 142868) | 176.9% (25.2% to 326.2%) |  | 30175 (22678 to 54930) | 98899 (60562 to 189925) | 227.7% (85.6% to 369.8%) |  | 4776 (3605 to 8016) | 20818 (13749 to 40929) | 335.9% (185.5% to 483.6%) |
| Lao People's Democratic Republic | 573 (240 to 2044) | 2033 (1153 to 3430) | 254.9% (25.8% to 668.5%) |  | 646 (318 to 1842) | 2075 (1343 to 3395) | 221.3% (41.4% to 561.5%) |  | 115 (60 to 274) | 369 (256 to 593) | 220.4% (61.5% to 551.4%) |
| Malaysia | 2085 (1462 to 3339) | 6117 (4233 to 9216) | 193.3% (57.2% to 361.0%) |  | 2853 (2095 to 4345) | 11167 (8191 to 14937) | 291.4% (119.5% to 513.6%) |  | 629 (443 to 928) | 2694 (1985 to 3680) | 328.6% (161.4% to 580.3%) |
| Maldives | 32 (15 to 120) | 105 (78 to 149) | 227.4% (-28.3% to 657.7%) |  | 40 (21 to 128) | 123 (90 to 169) | 202.9% (-18.9% to 539.1%) |  | 5 (3 to 13) | 32 (24 to 43) | 513.5% (130.9% to 1073.2%) |
| Mauritius | 180 (156 to 205) | 390 (288 to 512) | 116.0% (57.8% to 191.9%) |  | 208 (181 to 238) | 843 (648 to 1095) | 305.2% (208.9% to 443.8%) |  | 49 (42 to 58) | 213 (162 to 269) | 332.1% (219.0% to 476.8%) |
| Myanmar | 7804 (3939 to 22299) | 16898 (10749 to 25974) | 116.5% (-18.7% to 305.7%) |  | 8107 (5090 to 17768) | 22302 (15557 to 32319) | 175.1% (34.3% to 375.0%) |  | 1782 (952 to 3149) | 5150 (3368 to 7279) | 189.0% (68.0% to 504.1%) |
| Philippines | 14613 (11019 to 18998) | 37970 (25567 to 51300) | 159.8% (70.4% to 280.9%) |  | 12674 (9277 to 17742) | 41125 (30074 to 57662) | 224.5% (122.8% to 405.4%) |  | 2653 (1980 to 3867) | 8299 (6204 to 12846) | 212.8% (123.3% to 360.3%) |
| Sri Lanka | 1870 (1515 to 2322) | 3450 (2205 to 4946) | 84.5% (15.9% to 173.2%) |  | 1871 (1481 to 2437) | 6996 (4532 to 10145) | 274.0% (137.4% to 464.8%) |  | 420 (313 to 606) | 2345 (1570 to 3261) | 458.2% (229.2% to 775.8%) |
| Seychelles | 20 (16 to 25) | 52 (38 to 67) | 165.5% (84.3% to 274.3%) |  | 24 (19 to 35) | 80 (62 to 102) | 233.1% (131.9% to 368.2%) |  | 8 (6 to 12) | 22 (17 to 28) | 178.5% (77.9% to 308.0%) |
| Thailand | 10113 (7871 to 13006) | 17283 (11110 to 26367) | 70.9% (-0.2% to 170.1%) |  | 12113 (9640 to 15568) | 32578 (21828 to 48774) | 169.0% (71.6% to 315.7%) |  | 2562 (1949 to 3334) | 9694 (6714 to 13529) | 278.4% (146.1% to 446.7%) |
| Timor-Leste | 65 (27 to 189) | 201 (55 to 319) | 208.8% (-28.1% to 616.2%) |  | 66 (32 to 164) | 239 (152 to 357) | 260.6% (78.5% to 617.2%) |  | 8 (4 to 17) | 58 (39 to 88) | 659.7% (329.0% to 1440.7%) |
| Viet Nam | 5764 (3943 to 8412) | 18138 (12787 to 24805) | 214.7% (90.0% to 386.7%) |  | 10461 (7608 to 14079) | 39976 (26484 to 55223) | 282.1% (136.5% to 488.8%) |  | 3231 (2227 to 4188) | 9407 (6699 to 12472) | 191.1% (97.1% to 323.9%) |
| Oceania | 461 (308 to 1091) | 1653 (1007 to 3622) | 258.3% (133.2% to 442.1%) |  | 495 (339 to 988) | 1749 (1167 to 3163) | 253.3% (128.8% to 417.8%) |  | 92 (64 to 170) | 325 (236 to 516) | 253.9% (134.6% to 408.5%) |
| American  Samoa | 11 (8 to 16) | 24 (17 to 35) | 113.3% (29.9% to 255.2%) |  | 11 (8 to 17) | 38 (27 to 55) | 239.3% (106.4% to 446.6%) |  | 3 (2 to 4) | 11 (8 to 16) | 284.1% (133.0% to 500.9%) |
| Cook Islands | 1 (1 to 2) | 2 (1 to 3) | 78.9% (-16.2% to 236.0%) |  | 2 (1 to 2) | 6 (4 to 8) | 271.2% (93.7% to 530.6%) |  | 0 (0 to 1) | 2 (1 to 2) | 322.8% (132.7% to 591.3%) |
| Micronesia（Federated States of） | 10 (6 to 27) | 26 (7 to 51) | 152.0% (-34.2% to 484.4%) |  | 10 (6 to 25) | 38 (21 to 77) | 284.7% (87.7% to 728.7%) |  | 2 (1 to 6) | 6 (4 to 12) | 160.4% (23.9% to 460.5%) |
| Fiji | 48 (35 to 70) | 79 (55 to 110) | 64.7% (-0.2% to 159.8%) |  | 52 (38 to 76) | 139 (95 to 193) | 166.2% (70.1% to 312.8%) |  | 9 (6 to 13) | 28 (20 to 38) | 219.4% (107.5% to 379.8%) |
| Guam | 14 (10 to 21) | 32 (24 to 42) | 126.0% (42.7% to 244.2%) |  | 16 (11 to 24) | 56 (43 to 73) | 263.4% (128.1% to 447.6%) |  | 4 (3 to 6) | 18 (13 to 23) | 379.5% (206.5% to 597.0%) |
| Kiribati | 1 (1 to 2) | 2 (1 to 3) | 166.9% (39.0% to 324.1%) |  | 2 (1 to 3) | 6 (4 to 9) | 223.4% (81.8% to 418.7%) |  | 0 (0 to 1) | 1 (1 to 2) | 191.7% (66.4% to 371.2%) |
| Marshall  Islands | 6 (4 to 9) | 16 (10 to 24) | 270.0% (108.0% to 548.9%) |  | 6 (4 to 8) | 18 (11 to 28) | 391.7% (176.3% to 827.6%) |  | 1 (1 to 2) | 3 (2 to 6) | 142.4% (31.8% to 361.1%) |
| Nauru | 4 (2 to 9) | 14 (7 to 31) | 86.3% (2.5% to 282.3%) |  | 3 (2 to 7) | 16 (8 to 33) | 145.1% (31.2% to 410.7%) |  | 1 (1 to 2) | 2 (1 to 4) | 46.0% (-22.9% to 205.7%) |
| Niue | 1 (1 to 3) | 2 (1 to 4) | 29.8% (-32.6% to 137.3%) |  | 1 (0 to 2) | 2 (1 to 4) | 69.9% (-7.3% to 190.2%) |  | 0 (0 to 0) | 0 (0 to 0) | 38.4% (-24.0% to 125.3%) |
| Northern  Mariana Islands | 0 (0 to 0) | 0 (0 to 1) | 37.1% (-23.5% to 148.2%) |  | 0 (0 to 1) | 1 (0 to 1) | 719.7% (354.5% to 1258.1%) |  | 0 (0 to 0) | 0 (0 to 0) | 517.8% (212.0% to 962.0%) |
| Palau | 5 (3 to 10) | 7 (5 to 11) | 103.5% (13.0% to 247.2%) |  | 3 (2 to 5) | 25 (19 to 34) | 258.6% (109.2% to 458.7%) |  | 1 (0 to 1) | 4 (3 to 5) | 143.6% (43.9% to 293.4%) |
| Papua New  Guinea | 261 (147 to 685) | 1112 (615 to 2557) | 326.4% (155.4% to 615.5%) |  | 276 (155 to 636) | 1046 (618 to 2080) | 278.9% (124.5% to 526.6%) |  | 47 (26 to 103) | 173 (105 to 321) | 268.1% (128.6% to 509.5%) |
| Samoa | 27 (18 to 39) | 64 (31 to 117) | 135.3% (24.0% to 326.7%) |  | 43 (28 to 63) | 107 (51 to 192) | 150.5% (35.9% to 323.4%) |  | 10 (7 to 15) | 26 (13 to 47) | 146.5% (36.7% to 304.9%) |
| Solomon  Islands | 27 (11 to 134) | 138 (64 to 505) | 411.9% (168.9% to 1047.4%) |  | 21 (9 to 96) | 100 (52 to 316) | 370.8% (132.4% to 904.2%) |  | 2 (1 to 8) | 15 (9 to 40) | 572.3% (247.9% to 1418.3%) |
| Tokelau | 0 (0 to 0) | 0 (0 to 0) | 60.8% (-13.8% to 218.1%) |  | 0 (0 to 1) | 0 (0 to 1) | 63.1% (-17.5% to 228.7%) |  | 0 (0 to 0) | 0 (0 to 0) | 22.7% (-32.8% to 136.0%) |
| Tonga | 9 (5 to 12) | 16 (10 to 25) | 89.9% (12.3% to 205.4%) |  | 13 (8 to 18) | 24 (15 to 35) | 86.9% (15.1% to 187.8%) |  | 3 (2 to 5) | 9 (5 to 13) | 184.1% (76.7% to 344.0%) |
| Tuvalu | 1 (1 to 3) | 2 (1 to 4) | 63.7% (-8.5% to 191.4%) |  | 2 (1 to 4) | 4 (2 to 7) | 117.4% (19.4% to 278.6%) |  | 0 (0 to 1) | 1 (1 to 2) | 165.6% (48.5% to 377.3%) |
| Vanuatu | 8 (3 to 27) | 37 (17 to 88) | 362.0% (141.6% to 914.7%) |  | 7 (3 to 24) | 39 (19 to 97) | 443.5% (189.9% to 998.1%) |  | 2 (1 to 5) | 10 (5 to 23) | 497.5% (225.6% to 1044.7%) |
| North Africa and Middle East | 25668 (16352 to 52534) | 85302 (64472 to 102699) | 232.3% (71.4% to 382.8%) |  | 37603 (27247 to 69638) | 123427 (101705 to 146506) | 228.2% (69.4% to 363.2%) |  | 8390 (5884 to 13445) | 31988 (26626 to 38523) | 281.3% (117.8% to 459.8%) |
| Afghanistan | 933 (282 to 5921) | 3941 (1790 to 16800) | 322.4% (172.0% to 755.9%) |  | 1396 (490 to 7382) | 3255 (1455 to 13378) | 133.1% (47.9% to 333.8%) |  | 240 (79 to 817) | 583 (258 to 1543) | 142.6% (52.2% to 346.0%) |
| Algeria | 1586 (985 to 2375) | 5415 (3575 to 7492) | 241.5% (97.5% to 448.4%) |  | 1884 (1317 to 2654) | 7322 (4505 to 10010) | 288.7% (134.5% to 475.4%) |  | 517 (335 to 764) | 1801 (1120 to 2567) | 248.3% (119.7% to 414.3%) |
| Bahrain | 64 (48 to 97) | 321 (216 to 531) | 398.3% (179.2% to 732.6%) |  | 93 (65 to 151) | 495 (318 to 803) | 431.6% (150.1% to 941.1%) |  | 22 (15 to 32) | 98 (71 to 134) | 348.9% (145.7% to 643.4%) |
| Egypt | 2864 (2230 to 4279) | 8873 (4717 to 13919) | 209.9% (34.0% to 398.7%) |  | 3310 (2301 to 6738) | 12607 (6691 to 23809) | 280.9% (110.3% to 545.0%) |  | 649 (353 to 1773) | 1986 (957 to 4991) | 206.0% (70.6% to 471.8%) |
| Iran | 3094 (1680 to 6147) | 11936 (7362 to 14047) | 285.8% (68.8% to 477.2%) |  | 3684 (2521 to 6717) | 18065 (12220 to 20639) | 390.4% (132.8% to 632.9%) |  | 658 (437 to 1170) | 4835 (3748 to 5570) | 635.1% (252.7% to 1030.5%) |
| Iraq | 1408 (749 to 2548) | 8387 (4599 to 12915) | 495.8% (137.7% to 1211.8%) |  | 1569 (828 to 2866) | 7443 (5164 to 10553) | 374.3% (120.5% to 910.8%) |  | 351 (168 to 834) | 1474 (995 to 2559) | 320.0% (105.7% to 772.0%) |
| Jordan | 243 (158 to 383) | 1375 (942 to 1883) | 465.2% (168.8% to 893.0%) |  | 319 (209 to 520) | 1872 (1299 to 2512) | 486.6% (182.5% to 886.7%) |  | 66 (43 to 106) | 488 (343 to 658) | 642.8% (281.4% to 1154.1%) |
| Kuwait | 218 (173 to 285) | 634 (377 to 1018) | 190.7% (85.6% to 410.4%) |  | 163 (128 to 225) | 646 (404 to 961) | 297.3% (136.2% to 527.9%) |  | 40 (30 to 57) | 138 (95 to 203) | 241.4% (112.6% to 448.8%) |
| Lebanon | 561 (359 to 951) | 1419 (990 to 1951) | 153.1% (13.2% to 349.9%) |  | 1006 (673 to 1530) | 2929 (1918 to 4104) | 191.1% (37.8% to 380.7%) |  | 244 (162 to 372) | 1332 (858 to 1900) | 445.5% (137.3% to 832.9%) |
| Libya | 283 (167 to 534) | 1499 (992 to 2293) | 430.0% (147.6% to 965.0%) |  | 457 (277 to 772) | 2143 (1459 to 3111) | 369.3% (129.6% to 862.7%) |  | 118 (70 to 194) | 521 (339 to 740) | 342.4% (115.5% to 771.5%) |
| Morocco | 2394 (1605 to 3883) | 8662 (5230 to 13670) | 261.8% (59.1% to 558.1%) |  | 3793 (2727 to 5388) | 15669 (10157 to 23084) | 313.1% (111.8% to 590.4%) |  | 820 (558 to 1192) | 3258 (2067 to 4502) | 297.1% (102.9% to 558.5%) |
| Palestine | 129 (64 to 280) | 645 (373 to 837) | 399.9% (102.7% to 954.9%) |  | 198 (102 to 388) | 940 (538 to 1220) | 374.3% (103.7% to 901.3%) |  | 50 (26 to 93) | 249 (143 to 342) | 402.2% (126.0% to 1029.0%) |
| Oman | 64 (30 to 126) | 420 (238 to 604) | 559.2% (111.9% to 1315.0%) |  | 108 (50 to 196) | 585 (360 to 816) | 441.7% (94.7% to 1050.1%) |  | 26 (12 to 48) | 134 (87 to 185) | 408.1% (104.4% to 1121.3%) |
| Qatar | 29 (17 to 56) | 271 (169 to 444) | 848.5% (238.9% to 1748.9%) |  | 33 (18 to 54) | 346 (216 to 503) | 954.4% (347.1% to 1807.3%) |  | 7 (3 to 11) | 50 (25 to 76) | 630.9% (278.5% to 1319.5%) |
| Saudi Arabia | 856 (524 to 1584) | 6676 (4415 to 9437) | 679.9% (237.6% to 1408.6%) |  | 838 (527 to 1520) | 5620 (3820 to 7999) | 570.3% (208.1% to 1126.1%) |  | 209 (125 to 412) | 656 (414 to 1088) | 213.8% (43.9% to 478.7%) |
| Sudan | 828 (329 to 3691) | 3227 (1670 to 6250) | 289.8% (57.4% to 848.6%) |  | 895 (344 to 3351) | 3193 (1963 to 5679) | 256.6% (64.1% to 722.6%) |  | 230 (70 to 679) | 674 (390 to 1078) | 193.5% (47.8% to 554.0%) |
| Syrian Arab Republic | 590 (321 to 1080) | 1628 (961 to 2553) | 175.9% (7.7% to 487.4%) |  | 589 (364 to 953) | 2541 (1582 to 3903) | 331.7% (89.9% to 742.8%) |  | 112 (61 to 207) | 516 (317 to 823) | 362.7% (122.2% to 867.7%) |
| Tunisia | 593 (426 to 847) | 1730 (1090 to 2487) | 191.6% (52.0% to 388.7%) |  | 1024 (729 to 1418) | 3662 (2357 to 5210) | 257.7% (95.0% to 493.3%) |  | 277 (190 to 394) | 1087 (704 to 1529) | 292.0% (112.5% to 548.4%) |
| Turkey | 8370 (4361 to 16456) | 14191 (8300 to 19359) | 69.5% (-22.2% to 192.9%) |  | 15619 (8721 to 27964) | 30462 (18634 to 41376) | 95.0% (-6.6% to 222.6%) |  | 3625 (2062 to 5630) | 11534 (7187 to 15443) | 218.1% (79.7% to 431.9%) |
| United Arab  Emirates | 171 (95 to 304) | 1995 (1173 to 3476) | 1065.3% (452.9% to 2162.3%) |  | 100 (49 to 247) | 1204 (655 to 2429) | 1104.0% (447.1% to 2329.8%) |  | 15 (6 to 35) | 77 (35 to 148) | 421.5% (111.1% to 976.5%) |
| Yemen | 373 (107 to 1955) | 1970 (1036 to 5199) | 427.9% (144.6% to 1472.4%) |  | 499 (141 to 1996) | 2303 (1331 to 5271) | 361.8% (135.1% to 1151.0%) |  | 108 (24 to 334) | 466 (242 to 891) | 330.0% (130.3% to 1069.9%) |
| South Asia | 86836 (58700 to 137398) | 323472 (237328 to 421778) | 272.5% (104.8% to 454.0%) |  | 120211 (87859 to 175661) | 500374 (372853 to 641490) | 316.2% (165.6% to 482.8%) |  | 26431 (19752 to 37952) | 142109 (110533 to 191109) | 437.7% (230.4% to 638.4%) |
| Bangladesh | 5776 (3287 to 11554) | 21743 (12075 to 49887) | 276.4% (25.3% to 623.0%) |  | 6890 (3921 to 13021) | 32847 (20903 to 69035) | 376.7% (118.7% to 725.2%) |  | 1941 (979 to 3568) | 10340 (5631 to 21923) | 432.6% (163.0% to 804.2%) |
| Bhutan | 39 (17 to 93) | 136 (67 to 322) | 251.9% (11.7% to 696.1%) |  | 51 (24 to 107) | 203 (113 to 447) | 302.2% (58.6% to 686.6%) |  | 13 (6 to 26) | 73 (41 to 166) | 454.0% (148.5% to 953.0%) |
| India | 61722 (38720 to 102817) | 191664 (141604 to 242120) | 210.5% (95.2% to 378.9%) |  | 89536 (60711 to 137747) | 350850 (252298 to 448138) | 291.9% (165.3% to 451.8%) |  | 18883 (13343 to 27987) | 107136 (82564 to 134346) | 467.4% (273.8% to 710.1%) |
| Nepal | 1207 (594 to 2627) | 5371 (2992 to 12356) | 344.9% (52.4% to 806.5%) |  | 1597 (770 to 3173) | 8219 (4828 to 18441) | 414.7% (122.2% to 891.8%) |  | 345 (137 to 687) | 2581 (1494 to 5778) | 648.1% (262.3% to 1481.9%) |
| Pakistan | 18092 (11787 to 23134) | 104558 (48108 to 177745) | 477.9% (155.1% to 940.3%) |  | 22138 (16524 to 28118) | 108255 (54909 to 180586) | 389.0% (130.1% to 792.9%) |  | 5249 (3650 to 8517) | 21979 (11044 to 38460) | 318.8% (94.7% to 634.8%) |
| Southern sub-Saharan Africa | 6859 (5448 to 9185) | 14993 (11830 to 19115) | 118.6% (38.4% to 201.6%) |  | 8122 (6531 to 10097) | 24861 (19878 to 30177) | 206.1% (139.5% to 280.1%) |  | 2644 (2048 to 3229) | 8370 (6597 to 9752) | 216.6% (144.9% to 295.4%) |
| Botswana | 135 (78 to 200) | 609 (328 to 986) | 351.8% (128.4% to 705.6%) |  | 162 (105 to 251) | 710 (429 to 1097) | 338.8% (130.9% to 643.1%) |  | 46 (32 to 68) | 186 (126 to 275) | 302.6% (136.2% to 542.1%) |
| Lesotho | 134 (82 to 203) | 424 (224 to 827) | 215.8% (52.4% to 571.1%) |  | 230 (141 to 366) | 717 (362 to 1452) | 211.5% (54.5% to 535.4%) |  | 84 (54 to 124) | 226 (125 to 417) | 170.2% (46.0% to 409.4%) |
| Namibia | 96 (55 to 148) | 288 (164 to 463) | 198.8% (47.5% to 506.0%) |  | 164 (112 to 231) | 469 (305 to 705) | 185.7% (60.2% to 389.8%) |  | 51 (36 to 71) | 150 (106 to 209) | 192.4% (78.2% to 372.8%) |
| South Africa | 5237 (4048 to 7274) | 9423 (6825 to 13217) | 79.9% (1.7% to 174.2%) |  | 6033 (4634 to 7535) | 17814 (13234 to 22400) | 195.3% (128.4% to 258.7%) |  | 1996 (1469 to 2416) | 6465 (4992 to 7570) | 223.8% (157.1% to 301.9%) |
| Eswatini | 102 (54 to 214) | 253 (118 to 462) | 148.5% (19.7% to 402.0%) |  | 105 (53 to 207) | 313 (160 to 549) | 198.3% (47.0% to 518.0%) |  | 34 (18 to 69) | 108 (63 to 185) | 220.2% (83.6% to 477.1%) |
| Zimbabwe | 1155 (821 to 1586) | 3997 (2574 to 5707) | 245.9% (111.6% to 467.4%) |  | 1428 (1071 to 1889) | 4838 (3087 to 6851) | 238.9% (100.5% to 434.0%) |  | 433 (325 to 586) | 1236 (870 to 1704) | 185.7% (78.0% to 353.2%) |
| Western sub-Saharan Africa | 9199 (6868 to 13008) | 39495 (28148 to 56323) | 329.3% (158.2% to 547.5%) |  | 13278 (9951 to 19350) | 56888 (40353 to 77233) | 328.4% (153.9% to 547.6%) |  | 3624 (2625 to 5257) | 11869 (7724 to 16197) | 227.6% (89.3% to 416.7%) |
| Benin | 221 (155 to 291) | 986 (635 to 1466) | 345.4% (176.8% to 604.0%) |  | 300 (222 to 404) | 1147 (814 to 1632) | 281.8% (150.0% to 471.1%) |  | 68 (50 to 93) | 275 (201 to 370) | 303.8% (159.6% to 507.6%) |
| Burkina Faso | 484 (307 to 779) | 1878 (1235 to 2747) | 287.9% (132.4% to 511.9%) |  | 671 (444 to 1072) | 2258 (1603 to 3234) | 236.5% (118.4% to 408.4%) |  | 157 (99 to 250) | 492 (355 to 666) | 212.8% (95.5% to 387.5%) |
| Cameroon | 785 (562 to 1081) | 3718 (2126 to 6174) | 373.9% (173.3% to 676.3%) |  | 1095 (765 to 1560) | 4209 (2485 to 6817) | 284.2% (129.4% to 515.2%) |  | 200 (141 to 294) | 929 (538 to 1426) | 363.9% (164.3% to 631.1%) |
| Cabo Verde | 12 (8 to 15) | 65 (32 to 95) | 448.9% (175.7% to 712.3%) |  | 14 (9 to 23) | 99 (50 to 145) | 621.4% (146.6% to 1277.9%) |  | 4 (2 to 7) | 24 (11 to 47) | 520.8% (82.7% to 1371.3%) |
| Chad | 216 (134 to 340) | 690 (397 to 1161) | 220.1% (102.0% to 377.6%) |  | 323 (209 to 512) | 850 (553 to 1424) | 162.8% (81.0% to 284.9%) |  | 78 (50 to 122) | 173 (111 to 253) | 122.2% (48.9% to 232.1%) |
| Côte d’Ivoire | 656 (468 to 914) | 2614 (1612 to 3996) | 298.5% (131.2% to 571.4%) |  | 694 (505 to 946) | 2838 (1827 to 4207) | 308.9% (153.8% to 564.8%) |  | 105 (76 to 140) | 565 (368 to 794) | 436.6% (223.6% to 731.3%) |
| Gambia | 35 (23 to 51) | 212 (107 to 359) | 501.1% (187.4% to 988.2%) |  | 45 (29 to 64) | 270 (146 to 453) | 503.6% (190.6% to 1084.1%) |  | 13 (9 to 17) | 74 (41 to 122) | 485.8% (214.6% to 926.4%) |
| Ghana | 1112 (730 to 1546) | 5003 (2754 to 8535) | 349.8% (135.2% to 652.6%) |  | 1177 (787 to 1716) | 5686 (3198 to 9598) | 383.1% (152.4% to 692.7%) |  | 226 (154 to 335) | 1286 (676 to 2234) | 468.1% (185.0% to 868.4%) |
| Guinea | 472 (350 to 611) | 1576 (872 to 2483) | 233.5% (70.4% to 452.8%) |  | 769 (581 to 1003) | 1932 (1102 to 2987) | 151.4% (37.5% to 307.7%) |  | 187 (135 to 249) | 446 (241 to 675) | 138.2% (29.6% to 300.9%) |
| Guinea-Bissau | 70 (44 to 123) | 228 (145 to 376) | 226.8% (91.3% to 431.7%) |  | 76 (48 to 137) | 227 (135 to 395) | 197% (65.0% to 393.3%) |  | 14 (9 to 22) | 45 (26 to 71) | 223.5% (71.8% to 484.5%) |
| Liberia | 106 (73 to 160) | 556 (323 to 929) | 427.1% (173.6% to 813.3%) |  | 174 (123 to 262) | 591 (341 to 985) | 239.2% (76.9% to 477.8%) |  | 44 (31 to 64) | 128 (73 to 215) | 193.1% (43.7% to 429.0%) |
| Mali | 348 (269 to 446) | 1183 (627 to 1750) | 239.7% (69.2% to 431.4%) |  | 504 (380 to 661) | 1505 (846 to 2177) | 198.8% (57.0% to 350.0%) |  | 91 (66 to 120) | 278 (162 to 385) | 206.5% (73.3% to 366.9%) |
| Mauritania | 121 (91 to 162) | 416 (201 to 681) | 244.0% (57.3% to 502.7%) |  | 224 (168 to 310) | 706 (347 to 1122) | 215.8% (49.7% to 416.7%) |  | 59 (43 to 82) | 170 (83 to 259) | 186.1% (42.4% to 380.7%) |
| Niger | 291 (189 to 482) | 895 (461 to 1503) | 207.7% (90.5% to 367.3%) |  | 315 (204 to 526) | 1338 (791 to 2178) | 325.3% (175.9% to 547.5%) |  | 53 (34 to 86) | 245 (153 to 381) | 364.3% (199.5% to 606.2%) |
| Nigeria | 3472 (1963 to 6510) | 16423 (9909 to 26684) | 373.1% (107.2% to 865.0%) |  | 5897 (3562 to 10541) | 29010 (18444 to 43827) | 391.9% (133.2% to 857.8%) |  | 2099 (1233 to 3484) | 5750 (3362 to 8516) | 174.0% (45.9% to 427.1%) |
| São Tomé and  Príncipe | 8 (5 to 13) | 50 (24 to 88) | 489.7% (164.1% to 1045.3%) |  | 20 (14 to 26) | 70 (34 to 130) | 250.2% (77.9% to 519.5%) |  | 5 (4 to 6) | 13 (6 to 24) | 164.3% (35.5% to 365.7%) |
| Senegal | 399 (282 to 529) | 1419 (803 to 2222) | 256.0% (92.8% to 492.3%) |  | 512 (364 to 700) | 2229 (1345 to 3396) | 335.4% (133.7% to 630.3%) |  | 113 (82 to 152) | 520 (304 to 772) | 360.6% (150.3% to 641.9%) |
| Sierra Leone | 182 (116 to 265) | 718 (445 to 1141) | 294.0% (133.3% to 564.9%) |  | 247 (161 to 374) | 838 (514 to 1367) | 239.0% (95.6% to 446.7%) |  | 65 (45 to 99) | 214 (126 to 332) | 227.5% (81.6% to 417.4%) |
| Togo | 209 (145 to 288) | 864 (507 to 1387) | 313.4% (129.1% to 621.3%) |  | 221 (160 to 303) | 1084 (666 to 1675) | 391.3% (182.5% to 705.6%) |  | 42 (30 to 57) | 242 (141 to 378) | 477.5% (213.2% to 891.8%) |
| Eastern sub-Saharan Africa | 16637 (9644 to 42744) | 57704 (44216 to 73375) | 246.8% (45.8% to 505.1%) |  | 21696 (13381 to 47495) | 70569 (56376 to 85783) | 225.3% (60.9% to 408.8%) |  | 4645 (3029 to 8112) | 15944 (13246 to 18744) | 243.3% (118.5% to 383.5%) |
| Burundi | 571 (255 to 1625) | 1211 (754 to 2002) | 112.1% (6.3% to 314.1%) |  | 731 (357 to 1981) | 1483 (936 to 2484) | 102.8% (5.0% to 264.8%) |  | 187 (102 to 415) | 269 (172 to 415) | 43.7% (-20.2% to 133.0%) |
| Comoros | 45 (11 to 79) | 176 (89 to 283) | 293.7% (62.2% to 1497.9%) |  | 64 (29 to 102) | 269 (153 to 412) | 322.1% (99.2% to 859.4%) |  | 20 (13 to 28) | 82 (48 to 123) | 315.0% (125.8% to 627.5%) |
| Djibouti | 36 (19 to 59) | 249 (119 to 429) | 585.2% (187.7% to 1331.1%) |  | 41 (23 to 66) | 292 (163 to 465) | 609.1% (242.5% to 1255.4%) |  | 7 (4 to 10) | 54 (34 to 77) | 698.4% (335.4% to 1369.9%) |
| Eritrea | 229 (119 to 472) | 1076 (639 to 1706) | 370.1% (124.5% to 812.2%) |  | 288 (152 to 607) | 1463 (883 to 2303) | 408.6% (152.2% to 873.2%) |  | 45 (23 to 82) | 269 (158 to 416) | 503.8% (219.8% to 1092.0%) |
| Ethiopia | 4252 (1524 to 22596) | 11291 (6030 to 19924) | 165.5% (-20.3% to 588.2%) |  | 5789 (2260 to 23070) | 14679 (7587 to 23998) | 153.6% (-5.5% to 483.8%) |  | 938 (379 to 2928) | 3265 (1792 to 5304) | 247.9% (70.2% to 608.6%) |
| Kenya | 1143 (606 to 1933) | 7447 (5076 to 10329) | 551.7% (196.5% to 1156.0%) |  | 1646 (835 to 2677) | 9773 (6655 to 13555) | 493.8% (182.5% to 1022.1%) |  | 361 (189 to 620) | 1997 (1558 to 2476) | 452.5% (195.7% to 882.7%) |
| Madagascar | 1209 (689 to 2287) | 3744 (2305 to 5967) | 209.6% (73.7% to 430.4%) |  | 1322 (852 to 2062) | 4198 (2508 to 6853) | 217.6% (88.9% to 422.1%) |  | 253 (159 to 363) | 664 (415 to 1000) | 162.5% (65.7% to 324.8%) |
| Malawi | 767 (508 to 1077) | 2205 (1111 to 3705) | 187.5% (17.8% to 474.2%) |  | 968 (712 to 1283) | 2560 (1440 to 3982) | 164.5% (24.5% to 370.0%) |  | 216 (159 to 291) | 718 (404 to 1135) | 231.8% (65.3% to 465.1%) |
| Mozambique | 1343 (807 to 2079) | 4307 (2531 to 6391) | 220.6% (80.7% to 440.2%) |  | 1729 (1122 to 2627) | 4811 (3114 to 7114) | 178.2% (67.2% to 340.7%) |  | 404 (260 to 590) | 1185 (837 to 1669) | 193.6% (86.5% to 354.5%) |
| Rwanda | 854 (424 to 2202) | 2595 (1671 to 3823) | 203.9% (-1.1% to 607.2%) |  | 1124 (620 to 2658) | 3767 (2522 to 5310) | 235% (24.5% to 582.9%) |  | 237 (147 to 447) | 841 (578 to 1134) | 255.3% (64.1% to 495.7%) |
| Somalia | 604 (288 to 1355) | 1897 (1043 to 3684) | 214.3% (91.5% to 416.5%) |  | 698 (363 to 1504) | 2158 (1216 to 4172) | 208.9% (92.8% to 390.0%) |  | 105 (58 to 195) | 432 (254 to 762) | 311.4% (165.5% to 524.4%) |
| South Sudan | 483 (277 to 768) | 1055 (570 to 1687) | 118.6% (13.6% to 299.6%) |  | 530 (334 to 841) | 1276 (750 to 1877) | 140.5% (34.1% to 285.1%) |  | 167 (109 to 261) | 255 (162 to 372) | 52.2% (-9.1% to 122.5%) |
| United Republic of Tanzania | 2569 (1482 to 3940) | 9110 (5995 to 12875) | 254.6% (105.7% to 523.2%) |  | 3605 (2236 to 5320) | 11571 (8213 to 15157) | 221.0% (99.8% to 410.4%) |  | 887 (544 to 1295) | 2784 (2095 to 3606) | 214.0% (108.2% to 351.9%) |
| Uganda | 1559 (966 to 2272) | 7773 (4617 to 12803) | 398.6% (168.7% to 848.2%) |  | 2112 (1417 to 2916) | 8869 (5720 to 13155) | 319.9% (144.0% to 623.5%) |  | 624 (465 to 819) | 2353 (1589 to 3484) | 276.9% (136.9% to 472.9%) |
| Zambia | 962 (532 to 1689) | 3524 (2323 to 4988) | 266.4% (82.9% to 594.2%) |  | 1033 (620 to 1728) | 3344 (2280 to 4757) | 223.9% (78.8% to 472.2%) |  | 190 (117 to 289) | 762 (542 to 1053) | 301.2% (134.3% to 567.1%) |
| Central sub-Saharan Africa | 2810 (1592 to 6698) | 9597 (6071 to 16256) | 241.5% (101.6% to 498.4%) |  | 4319 (2447 to 9130) | 13369 (8517 to 22672) | 209.5% (88.3% to 419.9%) |  | 739 (413 to 1379) | 2677 (1558 to 4095) | 262.3% (135.1% to 462.8%) |
| Angola | 482 (245 to 1309) | 2318 (1210 to 4116) | 381.2% (143.9% to 790.0%) |  | 579 (284 to 1353) | 2934 (1622 to 5030) | 406.9% (197.2% to 788.0%) |  | 97 (45 to 203) | 512 (267 to 864) | 429.1% (211.8% to 764.9%) |
| Central African  Republic | 163 (92 to 476) | 383 (211 to 941) | 135.5% (53.3% to 283.8%) |  | 241 (132 to 644) | 490 (259 to 1136) | 103.1% (24.9% to 227.5%) |  | 39 (23 to 81) | 82 (48 to 153) | 110.7% (29.5% to 230.7%) |
| Congo | 177 (110 to 393) | 756 (437 to 1254) | 326.7% (129.0% to 664.4%) |  | 282 (179 to 599) | 895 (532 to 1507) | 217% (78.8% to 441.7%) |  | 59 (35 to 98) | 185 (120 to 278) | 216.0% (91.0% to 419.5%) |
| Democratic  Republic of the  Congo | 1897 (983 to 4216) | 5668 (3452 to 9603) | 198.8% (68.6% to 452.6%) |  | 3035 (1645 to 6337) | 8435 (5116 to 14618) | 177.9% (61.5% to 401.1%) |  | 499 (253 to 960) | 1743 (943 to 2803) | 248.9% (106.1% to 498.0%) |
| Equatorial  Guinea | 20 (9 to 64) | 156 (77 to 280) | 668.8% (95.5% to 1987.0%) |  | 32 (14 to 93) | 178 (99 to 306) | 465.6% (57.4% to 1337.6%) |  | 6 (3 to 14) | 42 (28 to 63) | 583.9% (158.9% to 1415.0%) |
| Gabon | 71 (45 to 138) | 315 (177 to 520) | 342.3% (79.9% to 774.0%) |  | 151 (94 to 291) | 438 (268 to 707) | 189.8% (32.0% to 429.1%) |  | 39 (23 to 64) | 113 (75 to 168) | 189.5% (47.9% to 383.5%) |

Data in parentheses are 95% uncertainty intervals. DALYs= disability-adjusted life-years. SDI=Sociodemographic index.
