# Supplementary material for: The global burden and associated factors of ovarian cancer in 1990–2019: findings from the Global Burden of Disease Study 2019
Source: BMC Public Health. 2022 Jul 30;22:1455. doi: 10.1186/s12889-022-13861-y (PMC9339194; doi:10.1186/s12889-022-13861-y)
Supplement: Supplementary file 4 — Additional file 4: Supplementary Table 4. Age-standardized death rate, DALY rate per 100 000 population for all risk factors to ovarian cancer by SDI regions during 1990–2019. [file 12889_2022_13861_MOESM4_ESM.docx]

Supplementary Table 4. Age-standardized death rate, DALY rate per 100 000 population for all risk factors to ovarian cancer by SDI regions during 1990–2019.

|  | 1990 | 1991 | 1992 | 1993 | 1994 | 1995 | 1996 | 1997 | 1998 | 1999 |
| --- | --- | --- | --- | --- | --- | --- | --- | --- | --- | --- |
| **Age-standardized death rate (per 100,000)** |  |  |  |  |  |  |  |  |  |  |
| Global | 0.6 (0.3 to 0.9) | 0.6 (0.3 to 0.9) | 0.6 (0.3 to 0.9) | 0.6 (0.3 to 0.9) | 0.6 (0.3 to 0.9) | 0.6 (0.3 to 0.9) | 0.6 (0.3 to 0.9) | 0.6 (0.3 to 0.9) | 0.6 (0.3 to 0.9) | 0.6 (0.3 to 1.0) |
| High SDI | 1.0 (0.5 to 1.7) | 1.0 (0.5 to 1.6) | 1.0 (0.5 to 1.6) | 1.0 (0.5 to 1.6) | 1.0 (0.5 to 1.6) | 1.0 (0.5 to 1.6) | 1.0 (0.5 to 1.6) | 1.0 (0.5 to 1.6) | 1.0 (0.5 to 1.6) | 1.0 (0.5 to 1.6) |
| High-middle SDI | 0.6 (0.3 to 0.9) | 0.6 (0.3 to 1.0) | 0.6 (0.3 to 1.0) | 0.6 (0.3 to 1.0) | 0.6 (0.3 to 1.0) | 0.6 (0.3 to 1.0) | 0.6 (0.3 to 1.0) | 0.6 (0.3 to 1.0) | 0.6 (0.3 to 1.0) | 0.6 (0.3 to 1.0) |
| Middle SDI | 0.2 (0.1 to 0.4) | 0.2 (0.1 to 0.4) | 0.2 (0.1 to 0.4) | 0.2 (0.1 to 0.4) | 0.2 (0.1 to 0.5) | 0.2 (0.1 to 0.5) | 0.3 (0.1 to 0.5) | 0.3 (0.1 to 0.5) | 0.3 (0.1 to 0.5) | 0.3 (0.1 to 0.5) |
| Low-middle SDI | 0.2 (0.1 to 0.4) | 0.2 (0.1 to 0.4) | 0.2 (0.1 to 0.4) | 0.2 (0.1 to 0.4) | 0.2 (0.1 to 0.4) | 0.2 (0.1 to 0.4) | 0.2 (0.1 to 0.4) | 0.2 (0.1 to 0.5) | 0.2 (0.1 to 0.5) | 0.2 (0.1 to 0.5) |
| Low SDI | 0.2 (0.1 to 0.4) | 0.2 (0.1 to 0.4) | 0.2 (0.1 to 0.4) | 0.2 (0.1 to 0.4) | 0.2 (0.1 to 0.4) | 0.2 (0.1 to 0.4) | 0.2 (0.1 to 0.4) | 0.2 (0.1 to 0.4) | 0.2 (0.1 to 0.4) | 0.2 (0.1 to 0.5) |
| **Age-standardized DALY rate (per 100,000)** |  |  |  |  |  |  |  |  |  |  |
| Global | 12.3 (5.8 to 20.7) | 12.2 (5.7 to 20.6) | 12.2 (5.6 to 20.6) | 12.3 (5.6 to 20.8) | 12.3 (5.6 to 20.8) | 12.4 (5.6 to 21.1) | 12.3 (5.6 to 21.0) | 12.3 (5.6 to 21.0) | 12.3 (5.6 to 21.0) | 12.3 (5.6 to 21.1) |
| High SDI | 22.5 (10.8 to 37.5) | 22.1 (10.6 to 36.7) | 21.7 (10.5 to 36.1) | 21.5 (10.4 to 35.6) | 21.3 (10.3 to 35.3) | 21.4 (10.3 to 35.4) | 21.2 (10.1 to 35.2) | 21.0 (9.9 to 35.0) | 20.9 (9.9 to 34.7) | 20.8 (9.8 to 34.7) |
| High-middle SDI | 13.8 (6.0 to 23.6) | 13.9 (6.1 to 23.7) | 14.1 (6.1 to 24.0) | 14.4 (6.1 to 24.4) | 14.5 (6.2 to 24.7) | 14.7 (6.3 to 25.1) | 14.5 (6.1 to 24.8) | 14.4 (6.1 to 24.7) | 14.3 (6.1 to 24.6) | 14.3 (6.1 to 24.5) |
| Middle SDI | 5.2 (2.0 to 10.3) | 5.3 (2.0 to 10.4) | 5.5 (2.1 to 10.9) | 5.6 (2.1 to 11.0) | 5.8 (2.2 to 11.1) | 5.9 (2.2 to 11.5) | 6.1 (2.3 to 11.8) | 6.3 (2.4 to 12.1) | 6.5 (2.4 to 12.4) | 6.6 (2.5 to 12.8) |
| Low-middle SDI | 4.4 (1.6 to 9.1) | 4.5 (1.7 to 9.3) | 4.6 (1.7 to 9.6) | 4.7 (1.8 to 9.8) | 4.8 (1.8 to 10.1) | 5.0 (1.9 to 10.3) | 5.2 (2.0 to 10.6) | 5.4 (2.1 to 11.1) | 5.5 (2.1 to 11.4) | 5.7 (2.2 to 12.0) |
| Low SDI | 4.5 (1.5 to 10.4) | 4.5 (1.6 to 10.4) | 4.6 (1.6 to 10.8) | 4.7 (1.6 to 10.7) | 4.8 (1.7 to 10.6) | 4.9 (1.7 to 10.8) | 5.0 (1.7 to 10.9) | 5.1 (1.8 to 11.1) | 5.2 (1.9 to 11.2) | 5.4 (1.9 to 11.5) |

|  | 2000 | 2001 | 2002 | 2003 | 2004 | 2005 | 2006 | 2007 | 2008 | 2009 |
| --- | --- | --- | --- | --- | --- | --- | --- | --- | --- | --- |
| **Age-standardized death rate (per 100,000)** |  |  |  |  |  |  |  |  |  |  |
| Global | 0.6 (0.3 to 1.0) | 0.6 (0.3 to 1.0) | 0.6 (0.3 to 1.0) | 0.6 (0.3 to 1.0) | 0.6 (0.3 to 1.0) | 0.6 (0.3 to 1.0) | 0.6 (0.3 to 1.0) | 0.6 (0.3 to 1.0) | 0.6 (0.3 to 1.0) | 0.6 (0.3 to 1.0) |
| High SDI | 1.0 (0.5 to 1.6) | 1.0 (0.5 to 1.6) | 1.0 (0.5 to 1.6) | 1.0 (0.5 to 1.6) | 1.0 (0.5 to 1.6) | 0.9 (0.5 to 1.6) | 1.0 (0.5 to 1.6) | 1.0 (0.5 to 1.6) | 1.0 (0.5 to 1.6) | 1.0 (0.5 to 1.6) |
| High-middle SDI | 0.6 (0.3 to 1.0) | 0.6 (0.3 to 1.1) | 0.6 (0.3 to 1.1) | 0.6 (0.3 to 1.1) | 0.7 (0.3 to 1.1) | 0.7 (0.3 to 1.1) | 0.7 (0.3 to 1.1) | 0.7 (0.3 to 1.1) | 0.7 (0.3 to 1.1) | 0.7 (0.3 to 1.1) |
| Middle SDI | 0.3 (0.1 to 0.5) | 0.3 (0.1 to 0.5) | 0.3 (0.1 to 0.6) | 0.3 (0.1 to 0.6) | 0.3 (0.1 to 0.6) | 0.3 (0.1 to 0.6) | 0.3 (0.1 to 0.6) | 0.3 (0.1 to 0.6) | 0.3 (0.1 to 0.6) | 0.3 (0.1 to 0.6) |
| Low-middle SDI | 0.2 (0.1 to 0.5) | 0.3 (0.1 to 0.5) | 0.3 (0.1 to 0.5) | 0.3 (0.1 to 0.6) | 0.3 (0.1 to 0.6) | 0.3 (0.1 to 0.6) | 0.3 (0.1 to 0.6) | 0.3 (0.1 to 0.6) | 0.3 (0.1 to 0.6) | 0.3 (0.1 to 0.6) |
| Low SDI | 0.2 (0.1 to 0.5) | 0.2 (0.1 to 0.5) | 0.2 (0.1 to 0.5) | 0.3 (0.1 to 0.5) | 0.3 (0.1 to 0.5) | 0.3 (0.1 to 0.5) | 0.3 (0.1 to 0.5) | 0.3 (0.1 to 0.6) | 0.3 (0.1 to 0.6) | 0.3 (0.1 to 0.6) |
| **Age-standardized DALY rate (per 100,000)** |  |  |  |  |  |  |  |  |  |  |
| Global | 12.4 (5.7 to 21.3) | 12.5 (5.7 to 21.5) | 12.7 (5.7 to 22.0) | 12.8 (5.7 to 22.2) | 12.9 (5.8 to 22.5) | 12.9 (5.8 to 22.7) | 13.0 (5.8 to 22.8) | 13.0 (5.8 to 23.0) | 13.2 (5.8 to 23.2) | 13.3 (5.9 to 23.2) |
| High SDI | 20.8 (9.7 to 34.5) | 20.7 (9.6 to 34.6) | 20.8 (9.6 to 34.8) | 20.8 (9.6 to 34.8) | 20.7 (9.6 to 34.7) | 20.3 (9.4 to 34.4) | 20.3 (9.3 to 34.4) | 20.4 (9.4 to 34.6) | 20.6 (9.4 to 34.9) | 20.8 (9.5 to 35.0) |
| High-middle SDI | 14.6 (6.3 to 24.9) | 14.7 (6.3 to 25.2) | 15.0 (6.5 to 25.7) | 15.2 (6.6 to 26.0) | 15.3 (6.6 to 26.5) | 15.5 (6.6 to 26.9) | 15.4 (6.5 to 26.7) | 15.5 (6.5 to 26.8) | 15.5 (6.6 to 26.8) | 15.5 (6.5 to 27.0) |
| Middle SDI | 6.8 (2.6 to 12.8) | 6.9 (2.6 to 13.3) | 7.2 (2.8 to 13.6) | 7.4 (2.8 to 14.2) | 7.6 (2.9 to 14.4) | 7.8 (2.9 to 14.7) | 7.9 (3.0 to 15.0) | 8.0 (3.1 to 15.1) | 8.2 (3.1 to 15.5) | 8.3 (3.2 to 15.8) |
| Low-middle SDI | 5.9 (2.3 to 12.3) | 6.2 (2.3 to 12.9) | 6.5 (2.4 to 13.1) | 6.7 (2.5 to 13.6) | 6.9 (2.6 to 14.0) | 7.2 (2.7 to 14.4) | 7.5 (2.8 to 14.9) | 7.7 (2.9 to 15.3) | 7.9 (3.0 to 15.6) | 8.0 (3.0 to 16.0) |
| Low SDI | 5.5 (2.0 to 11.5) | 5.7 (2.1 to 12.0) | 5.9 (2.2 to 12.2) | 6.1 (2.2 to 12.7) | 6.3 (2.3 to 13.0) | 6.6 (2.4 to 13.4) | 6.7 (2.5 to 13.6) | 6.9 (2.5 to 13.8) | 7.1 (2.7 to 14.1) | 7.3 (2.7 to 14.4) |

|  | 2010 | 2011 | 2012 | 2013 | 2014 | 2015 | 2016 | 2017 | 2018 | 2019 |
| --- | --- | --- | --- | --- | --- | --- | --- | --- | --- | --- |
| **Age-standardized death rate (per 100,000)** |  |  |  |  |  |  |  |  |  |  |
| Global | 0.6 (0.3 to 1.0) | 0.6 (0.3 to 1.0) | 0.6 (0.3 to 1.0) | 0.6 (0.3 to 1.0) | 0.6 (0.3 to 1.1) | 0.6 (0.3 to 1.1) | 0.6 (0.3 to 1.1) | 0.6 (0.3 to 1.1) | 0.6 (0.3 to 1.1) | 0.6 (0.3 to 1.1) |
| High SDI | 0.9 (0.5 to 1.6) | 0.9 (0.5 to 1.6) | 0.9 (0.5 to 1.6) | 0.9 (0.5 to 1.6) | 0.9 (0.5 to 1.6) | 0.9 (0.4 to 1.5) | 0.9 (0.4 to 1.5) | 0.9 (0.4 to 1.5) | 0.9 (0.4 to 1.6) | 0.9 (0.4 to 1.6) |
| High-middle SDI | 0.7 (0.3 to 1.1) | 0.7 (0.3 to 1.1) | 0.6 (0.3 to 1.1) | 0.6 (0.3 to 1.1) | 0.6 (0.3 to 1.1) | 0.6 (0.3 to 1.1) | 0.6 (0.3 to 1.1) | 0.6 (0.3 to 1.1) | 0.6 (0.3 to 1.1) | 0.6 (0.3 to 1.1) |
| Middle SDI | 0.3 (0.1 to 0.7) | 0.4 (0.1 to 0.7) | 0.4 (0.1 to 0.7) | 0.4 (0.1 to 0.7) | 0.4 (0.1 to 0.7) | 0.4 (0.1 to 0.7) | 0.4 (0.1 to 0.7) | 0.4 (0.1 to 0.8) | 0.4 (0.2 to 0.8) | 0.4 (0.2 to 0.8) |
| Low-middle SDI | 0.3 (0.1 to 0.7) | 0.4 (0.1 to 0.7) | 0.4 (0.1 to 0.7) | 0.4 (0.1 to 0.7) | 0.4 (0.1 to 0.8) | 0.4 (0.2 to 0.8) | 0.4 (0.2 to 0.8) | 0.4 (0.2 to 0.8) | 0.5 (0.2 to 0.9) | 0.5 (0.2 to 0.9) |
| Low SDI | 0.3 (0.1 to 0.6) | 0.3 (0.1 to 0.6) | 0.3 (0.1 to 0.6) | 0.3 (0.1 to 0.7) | 0.4 (0.1 to 0.7) | 0.4 (0.1 to 0.7) | 0.4 (0.1 to 0.7) | 0.4 (0.2 to 0.8) | 0.4 (0.2 to 0.8) | 0.4 (0.2 to 0.8) |
| **Age-standardized DALY rate (per 100,000)** |  |  |  |  |  |  |  |  |  |  |
| Global | 13.1 (5.7 to 23.1) | 13.1 (5.6 to 23.2) | 13.2 (5.7 to 23.4) | 13.3 (5.7 to 23.7) | 13.4 (5.7 to 23.9) | 13.4 (5.6 to 24.0) | 13.4 (5.6 to 23.9) | 13.5 (5.6 to 24.2) | 13.7 (5.6 to 25.0) | 13.9 (5.7 to 25.3) |
| High SDI | 20.1 (9.1 to 33.9) | 20.0 (9.0 to 33.8) | 20.0 (9.0 to 33.8) | 19.9 (9.0 to 33.8) | 19.9 (9.0 to 33.6) | 19.3 (8.6 to 32.6) | 19.4 (8.6 to 32.6) | 19.4 (8.6 to 33.1) | 19.6 (8.9 to 33.7) | 19.9 (8.8 to 34.5) |
| High-middle SDI | 15.4 (6.3 to 26.8) | 15.2 (6.2 to 26.5) | 15.1 (6.1 to 26.2) | 14.9 (6.1 to 26.0) | 14.9 (5.9 to 26.5) | 14.8 (5.8 to 26.0) | 14.7 (5.9 to 25.8) | 14.6 (5.9 to 25.8) | 14.7 (5.8 to 26.1) | 14.8 (5.8 to 26.4) |
| Middle SDI | 8.5 (3.2 to 16.0) | 8.7 (3.2 to 16.4) | 8.9 (3.3 to 16.7) | 9.1 (3.3 to 17.4) | 9.4 (3.4 to 17.9) | 9.5 (3.5 to 18.2) | 9.7 (3.5 to 18.4) | 9.9 (3.6 to 18.9) | 10.1 (3.6 to 19.2) | 10.3 (3.6 to 19.9) |
| Low-middle SDI | 8.2 (3.1 to 16.1) | 8.5 (3.1 to 16.9) | 8.9 (3.3 to 17.5) | 9.3 (3.4 to 18.4) | 9.7 (3.6 to 19.3) | 10.1 (3.7 to 19.7) | 10.4 (3.7 to 20.4) | 10.7 (3.8 to 21.1) | 11.1 (4.0 to 22.3) | 11.5 (4.1 to 23.4) |
| Low SDI | 7.5 (2.8 to 14.7) | 7.7 (2.8 to 15.3) | 8.1 (3.1 to 15.9) | 8.4 (3.2 to 16.7) | 8.8 (3.4 to 17.2) | 9.0 (3.4 to 17.5) | 9.4 (3.5 to 18.1) | 9.6 (3.6 to 18.8) | 10.0 (3.6 to 19.3) | 10.4 (3.7 to 20.4) |

DALYs= disability-adjusted life-years.
