# Supplementary material for: The global burden and associated factors of ovarian cancer in 1990–2019: findings from the Global Burden of Disease Study 2019
Source: BMC Public Health. 2022 Jul 30;22:1455. doi: 10.1186/s12889-022-13861-y (PMC9339194; doi:10.1186/s12889-022-13861-y)
Supplement: Supplementary file 5 — Additional file 5: Supplementary Table 5.Leading 3 risk factors of ovarian cancer by deaths at the global and SDI level, 1990 and 2019 for females. [file 12889_2022_13861_MOESM5_ESM.docx]

Supplementary Table 5．Leading 3 risk factors of ovarian cancer by deaths at the global and SDI level, 1990 and 2019 for females.

|  | Leading risks in 1990 | Leading risks in 2019 |
| --- | --- | --- |
| All |  |  |
| 1 | High fasting plasma glucose | High fasting plasma glucose |
| 2 | Occupational exposure to asbestos | Occupational exposure to asbestos |
| 3 | High body-mass index | High body-mass index |
| High SDI |  |  |
| 1 | High fasting plasma glucose | High fasting plasma glucose |
| 2 | Occupational exposure to asbestos | Occupational exposure to asbestos |
| 3 | High body-mass index | High body-mass index |
| High-middle SDI |  |  |
| 1 | High fasting plasma glucose | High fasting plasma glucose |
| 2 | Occupational exposure to asbestos | High body-mass index |
| 3 | High body-mass index | Occupational exposure to asbestos |
| Middle SDI |  |  |
| 1 | High fasting plasma glucose | High fasting plasma glucose |
| 2 | High body-mass index | High body-mass index |
| 3 | Occupational exposure to asbestos | Occupational exposure to asbestos |
| Low-middle SDI |  |  |
| 1 | High fasting plasma glucose | High fasting plasma glucose |
| 2 | Occupational exposure to asbestos | High body-mass index |
| 3 | High body-mass index | Occupational exposure to asbestos |
| Low SDI |  |  |
| 1 | High fasting plasma glucose | High fasting plasma glucose |
| 2 | Occupational exposure to asbestos | High body-mass index |
| 3 | High body-mass index | Occupational exposure to asbestos |
| 4 | High body-mass index | Occupational exposure to asbestos |

SDI=Sociodemographic index.
