# Supplementary material for: The global burden and associated factors of ovarian cancer in 1990–2019: findings from the Global Burden of Disease Study 2019
Source: BMC Public Health. 2022 Jul 30;22:1455. doi: 10.1186/s12889-022-13861-y (PMC9339194; doi:10.1186/s12889-022-13861-y)
Supplement: Supplementary file 6 — Additional file 6: Supplementary Table 6. Deaths in 1990 and 2019, percent of total deaths and percentage change in age-standardized rate per 100,000 population for ovarian cancer during 1990–2019 by top three attributed risk factors. [file 12889_2022_13861_MOESM6_ESM.docx]

Supplementary Table 6. Deaths in 1990 and 2019, percent of total deaths and percentage change in age-standardized rate per 100,000 population for ovarian cancer during 1990–2019 by top three attributed risk factors.

|  | High fasting plasma glucose | | | |  | Occupational exposure to asbestos | | | |  | High body-mass index | | | |
| --- | --- | --- | --- | --- | --- | --- | --- | --- | --- | --- | --- | --- | --- | --- |
|  | 1990 | 2019 | Percent of total deaths of ovarian cancer in 2019 | Percentage change in age-standardized rate,1990-2019 |  | 1990 | 2019 | Percent of total deaths of ovarian cancer in 2019 | Percentage change in age-standardized rate,1990-2019 |  | 1990 | 2019 | Percent of total deaths of ovarian cancer in 2019 | Percentage change in age-standardized rate,1990-2019 |
| **Global** | 5537 (1096 to 13331) | 15736 (3023 to 36227) | 7.9% (1.6% to 18.3%) | 34.7% (18.6% to 51.4%) |  | 4030 (1857 to 6541) | 6557 (2951 to 10664) | 3.3% (1.5% to 5.4%) | -24.9% (-46.7% to -7.4%) |  | 2630 (-86 to 6141) | 6309 (-177 to 14309) | 3.2% (-0.1% to 7.1%) | 16.4% (2.7% to 32.0%) |
| **SDI regions** |  |  |  |  |  |  |  |  |  |  |  |  |  |  |
| High SDI | 2842 (557 to 6686) | 5362 (1098 to 12355) | 9.5% (2.0% to 21.4%) | 8.9% (0.6% to 27.8%) |  | 2619 (1183 to 4224) | 3514 (1595 to 5854) | 6.2% (2.8% to 10.1%) | -26.8% (-47.9% to -7.1%) |  | 1319 (-41 to 3027) | 2124 (-61 to 4898) | 3.7% (-0.1% to 8.4%) | -5.0% (-14.1% to 14.5%) |
| High-middle SDI | 1529 (304 to 3653) | 3795 (710 to 8859) | 7.3% (1.5% to 17.1%) | 31.9% (16.2% to 47.1%) |  | 1059 (481 to 1700) | 1721 (732 to 2822) | 3.3% (1.5% to 5.4%) | -15.8% (-42.7% to 8.9%) |  | 983 (-32 to 2252) | 1983 (-56 to 4526) | 3.8% (-0.1% to 8.6%) | 10.4% (-1.5% to 24.6%) |
| Middle SDI | 710 (133 to 1763) | 3618 (691 to 8516) | 7.5% (1.5% to 17.4%) | 98.1% (50.7% to 135.9%) |  | 180 (82 to 336) | 634 (276 to 1087) | 1.3% (0.6% to 2.2%) | 33.8% (-17.7% to 83.8%) |  | 222 (-8 to 563) | 1352 (-36 to 3131) | 2.8% (-0.1% to 6.4%) | 152.9% (90.6% to 229.4%) |
| Low-middle SDI | 336 (59 to 865) | 2266 (425 to 5349) | 7.6% (1.5% to 17.6%) | 169.4% (74.6% to 268.1%) |  | 112 (43 to 241) | 481 (197 to 892) | 1.6% (0.7% to 2.8%) | 63.4% (-2.5% to 141.6%) |  | 75 (-2 to 205) | 631 (-17 to 1486) | 2.1% (-0.1% to 5.0%) | 5.3% (-2.5% to 26.6%) |
| Low SDI | 118 (19 to 328) | 685 (132 to 1654) | 6.0% (1.2% to 14.1%) | 151.4% (55.3% to 265.3%) |  | 58 (15 to 146) | 203 (73 to 371) | 1.8% (0.7% to 3.2%) | 58.3% (-22.4% to 188.0%) |  | 31 (-1 to 93) | 215 (-7 to 525) | 1.9% (-0.1% to 4.6%) | 208.5% (77.9% to 451.5%) |
| **World regions** |  |  |  |  |  |  |  |  |  |  |  |  |  |  |
| Central Asia | 42 (8 to 103) | 157 (31 to 369) | 7.7% (1.6% to 17.9%) | 146.5% (107.6% to 208.0%) |  | 24 (7 to 60) | 43 (14 to 85) | 2.1% (0.7% to 4.0%) | 26.1% (-40.2% to 178.4%) |  | 34 (-1 to 77) | 91 (-3 to 204) | 4.4% (-0.1% to 10.0%) | 69.2% (41.7% to 107.3%) |
| Central Europe | 361 (71 to 852) | 773 (152 to 1801) | 9.0% (1.8% to 20.5%) | 45.9% (26.8% to 72.9%) |  | 121 (56 to 202) | 214 (87 to 377) | 2.5% (1.0% to 4.3%) | 16.5% (-41.1% to 66.2%) |  | 231 (-7 to 529) | 394 (-11 to 893) | 4.6% (-0.1% to 10.4%) | 23.2% (5.9% to 47.7%) |
| Eastern Europe | 383 (71 to 951) | 636 (110 to 1564) | 4.8% (0.9% to 11.6%) | 40.3% (20.8% to 70.0%) |  | 325 (134 to 559) | 406 (172 to 710) | 3.1% (1.3% to 5.1%) | 5.0% (-33.0% to 56.7%) |  | 468 (-14 to 1061) | 683 (-20 to 1492) | 5.1% (-0.1% to 11.5%) | 25.8% (5.7% to 58.9%) |
| Australasia | 36 (6 to 86) | 96 (18 to 229) | 6.9% (1.3% to 16.1%) | 26.0% (6.4% to 61.8%) |  | 83 (42 to 142) | 160 (78 to 265) | 11.5% (5.8% to 18.4%) | -9.9% (-49.9% to 36.8%) |  | 29 (-1 to 67) | 59 (-2 to 135) | 4.3% (-0.1% to 9.7%) | -0.6% (-18.0% to 33.7%) |
| High-income Asia Pacific | 166 (31 to 406) | 408 (76 to 990) | 5.6% (1.0% to 13.2%) | 12.8% (-8.0% to 25.5%) |  | 96 (44 to 166) | 224 (91 to 396) | 3.0% (1.3% to 5.1%) | -22.2% (-56.1% to 13.7%) |  | 57 (-2 to 162) | 111 (-3 to 305) | 1.5% (0.0% to 4.2%) | 3.7% (-14.3% to 30.6%) |
| High-income North America | 1206 (237 to 2846) | 2359 (490 to 5416) | 10.9% (2.3% to 24.4%) | 13.4% (3.3% to 37.1%) |  | 721 (316 to 1150) | 1054 (468 to 1746) | 4.9% (2.2% to 8.0%) | -12.7% (-30.9% to 9.0%) |  | 568 (-17 to 1280) | 1009 (-28 to 2254) | 4.7% (-0.1% to 10.3%) | 2.1% (-8.4% to 22.9%) |
| Southern Latin America | 80 (15 to 196) | 215 (42 to 504) | 8.4% (1.7% to 19.3%) | 46.7% (19.6% to 88.9%) |  | 33 (15 to 60) | 91 (38 to 161) | 3.6% (1.5% to 6.3%) | 46.1% (-30.5% to 125.9%) |  | 38 (-1 to 92) | 97 (-3 to 224) | 3.8% (-0.1% to 8.8%) | 44.3% (13.0% to 92.9%) |
| Western Europe | 1741 (342 to 4126) | 2994 (618 to 6914) | 9.8% (2.0% to 22.1%) | 13.8% (3.4% to 30.0%) |  | 2122 (955 to 3437) | 2628 (1161 to 4295) | 8.6% (3.8% to 13.7%) | -22.0% (-47.9% to 2.8%) |  | 765 (-24 to 1781) | 1064 (-31 to 2447) | 3.5% (-0.1% to 8.0%) | -7.4% (-17.0% to 10.7%) |
| Andean Latin America | 12 (2 to 31) | 91 (16 to 222) | 6.7% (1.3% to 15.5%) | 169.1% (55.2% to 281.3%) |  | 8 (3 to 15) | 30 (11 to 60) | 2.1% (0.9% to 3.9%) | 33.2% (-29.8% to 149.8%) |  | 8 (0 to 20) | 58 (-1 to 132) | 4.2% (-0.1% to 9.6%) | 161.5% (56.1% to 298.2%) |
| Caribbean | 14 (3 to 33) | 111 (21 to 254) | 10.6% (2.2% to 23.9%) | 282.3% (66.1% to 388.6%) |  | 2 (1 to 6) | 12 (5 to 25) | 1.1% (0.5% to 2.1%) | 127.8% (7.7% to 264.1%) |  | 5 (0 to 13) | 42 (-1 to 94) | 4.0% (-0.1% to 9.2%) | 287.3% (71.8% to 393.2%) |
| Central Latin America | 153 (32 to 345) | 658 (134 to 1488) | 10.7% (2.3% to 23.9%) | 39.5% (15.4% to 63.5%) |  | 27 (11 to 45) | 102 (44 to 182) | 1.7% (0.7% to 2.8%) | 19.5% (-20.7% to 65.0%) |  | 53 (-2 to 124) | 252 (-7 to 577) | 4.1% (-0.1% to 9.4%) | 70.1% (38.7% to 107.8%) |
| Tropical Latin America | 132 (26 to 313) | 415 (81 to 966) | 7.6% (1.5% to 18.0%) | 10.8% (0.1% to 23.8%) |  | 75 (32 to 121) | 227 (103 to 374) | 4.2% (1.9% to 6.8%) | -6.3% (-33.5% to 16.6%) |  | 55 (-2 to 130) | 213 (-6 to 474) | 3.9% (-0.1% to 8.8%) | 48.2% (25.3% to 86.3%) |
| North Africa and Middle East | 253 (44 to 643) | 1455 (294 to 3316) | 9.3% (1.9% to 21.3%) | 128.9% (30.4% to 226.7%) |  | 142 (45 to 323) | 313 (119 to 581) | 2.0% (0.8% to 3.7%) | -6.5% (-59.6% to 97.7%) |  | 165 (-6 to 392) | 772 (-19 to 1693) | 4.9% (-0.1% to 11.0%) | 87.7% (0.0% to 170.2%) |
| South Asia | 714 (125 to 1843) | 5221 (947 to 12666) | 8.1% (1.7% to 18.8%) | 159.2% (64.8% to 260.5%) |  | 212 (68 to 500) | 976 (372 to 1944) | 1.5% (0.6% to 2.8%) | 54.5% (-18.6% to 168.5%) |  | 119 (-3 to 355) | 1214 (-31 to 2908) | 1.9% (-0.1% to 4.4%) | 296.3% (127.7% to 647.0%) |
| East Asia | 403 (73 to 1073) | 1846 (319 to 4633) | 6.1% (1.2% to 14.4%) | 82.9% (2.5% to 161.2%) |  | 114 (48 to 249) | 411 (151 to 747) | 1.3% (0.6% to 2.3%) | 37.8% (-50.6% to 165.3%) |  | 76 (-2 to 238) | 593 (-14 to 1541) | 1.9% (-0.1% to 5.1%) | 231.4% (93.0% to 563.6%) |
| Oceania | 2 (0 to 5) | 12 (2 to 29) | 10.9% (2.3% to 24.3%) | 134.1% (57.6% to 232.3%) |  | 0 (0 to 1) | 1 (0 to 2) | 0.8% (0.3% to 1.6%) | 38.8% (-26.0% to 150.5%) |  | 1 (0 to 2) | 3 (0 to 8) | 2.9% (-0.1% to 6.8%) | 63.1% (9.7% to 128.4%) |
| Southeast Asia | 207 (37 to 512) | 1083 (198 to 2684) | 6.7% (1.3% to 15.6%) | 110.5% (45.2% to 174.4%) |  | 27 (11 to 61) | 85 (32 to 163) | 0.5% (0.2% to 0.9%) | 29.8% (-27.3% to 113.7%) |  | 46 (-1 to 132) | 350 (-11 to 897) | 2.2% (-0.1% to 5.1%) | 227.1% (110.7% to 474.0%) |
| Central sub-Saharan Africa | 10 (1 to 29) | 45 (7 to 121) | 5.6% (1.1% to 13.4%) | 89.0% (23.9% to 190.3%) |  | 6 (1 to 15) | 20 (5 to 43) | 2.5% (0.6% to 4.7%) | 35.5% (-32.6% to 181.3%) |  | 3 (0 to 9) | 14 (-1 to 36) | 1.8% (-0.1% to 4.3%) | 83.0% (12.1% to 219.2%) |
| Eastern sub-Saharan Africa | 42 (6 to 118) | 177 (33 to 440) | 4.0% (0.7% to 9.7%) | 88.2% (3.4% to 175.4%) |  | 36 (6 to 91) | 111 (27 to 218) | 2.5% (0.6% to 4.7%) | 42.7% (-42.3% to 184.7%) |  | 14 (0 to 42) | 96 (-3 to 230) | 2.2% (-0.1% to 5.1%) | 206.3% (67.2% to 465.3%) |
| Southern sub-Saharan Africa | 34 (6 to 82) | 156 (31 to 366) | 9.3% (1.9% to 21.0%) | 112.1% (71.6% to 160.6%) |  | 25 (10 to 42) | 72 (29 to 122) | 4.3% (1.8% to 7.0%) | 36.6% (9.2% to 78.1%) |  | 24 (-1 to 53) | 87 (-2 to 194) | 5.1% (-0.1% to 11.2%) | 75.0% (38.7% to 113.1%) |
| Western sub-Saharan Africa | 30 (5 to 77) | 167 (30 to 419) | 4.9% (0.9% to 11.4%) | 150.6% (47.7% to 279.6%) |  | 7 (2 to 16) | 23 (7 to 46) | 0.7% (0.2% to 1.3%) | 43.8% (-21.0% to 175.3%) |  | 14 (0 to 35) | 100 (-2 to 246) | 2.9% (-0.1% to 6.7%) | 202.7% (74.9% to 410.4%) |

Data in parentheses are 95% uncertainty intervals. SDI=Sociodemographic index.
