# Supplementary material for: The global burden and associated factors of ovarian cancer in 1990–2019: findings from the Global Burden of Disease Study 2019
Source: BMC Public Health. 2022 Jul 30;22:1455. doi: 10.1186/s12889-022-13861-y (PMC9339194; doi:10.1186/s12889-022-13861-y)
Supplement: Supplementary file 7 — Additional file 7: Supplementary Table 7. Age-standardized death rate per 100 000 population for ovarian cancer due to high fasting plasma glucose by global and SDI regions during 1990–2019. [file 12889_2022_13861_MOESM7_ESM.docx]

Supplementary Table **7.** Age-standardized death rate per 100 000 population for ovarian cancer due to high fasting plasma glucose by global and SDI regions during 1990–2019.

|  | 1990 | 1991 | 1992 | 1993 | 1994 | 1995 | 1996 | 1997 | 1998 | 1999 |
| --- | --- | --- | --- | --- | --- | --- | --- | --- | --- | --- |
| **Age-standardized death rate (per 100,000)** |  |  |  |  |  |  |  |  |  |  |
| Global | 0.3 (0.1 to 0.6) | 0.3 (0.1 to 0.6) | 0.3 (0.1 to 0.6) | 0.3 (0.1 to 0.6) | 0.3 (0.1 to 0.6) | 0.3 (0.1 to 0.6) | 0.3 (0.1 to 0.6) | 0.3 (0.1 to 0.6) | 0.3 (0.1 to 0.6) | 0.3 (0.1 to 0.7) |
| High SDI | 0.5 (0.1 to 1.1) | 0.4 (0.1 to 1.1) | 0.4 (0.1 to 1.0) | 0.4 (0.1 to 1.0) | 0.4 (0.1 to 1.0) | 0.4 (0.1 to 1.0) | 0.4 (0.1 to 1.0) | 0.4 (0.1 to 1.0) | 0.4 (0.1 to 1.0) | 0.4 (0.1 to 1.0) |
| High-middle SDI | 0.3 (0.1 to 0.6) | 0.3 (0.0 to 0.6) | 0.3 (0.1 to 0.6) | 0.3 (0.1 to 0.6) | 0.3 (0.1 to 0.6) | 0.3 (0.1 to 0.6) | 0.3 (0.1 to 0.6) | 0.3 (0.1 to 0.7) | 0.3 (0.1 to 0.7) | 0.3 (0.1 to 0.7) |
| Middle SDI | 0.1 (0.0 to 0.3) | 0.1 (0.0 to 0.4) | 0.1 (0.0 to 0.4) | 0.2 (0.0 to 0.4) | 0.2 (0.0 to 0.4) | 0.2 (0.0 to 0.4) | 0.2 (0.0 to 0.4) | 0.2 (0.0 to 0.4) | 0.2 (0.0 to 0.4) | 0.2 (0.0 to 0.4) |
| Low-middle SDI | 0.1 (0.0 to 0.3) | 0.1 (0.0 to 0.3) | 0.1 (0.0 to 0.3) | 0.1 (0.0 to 0.3) | 0.1 (0.0 to 0.3) | 0.1 (0.0 to 0.4) | 0.1 (0.0 to 0.4) | 0.1 (0.0 to 0.4) | 0.2 (0.0 to 0.4) | 0.2 (0.0 to 0.4) |
| Low SDI | 0.1 (0.0 to 0.3) | 0.1 (0.0 to 0.3) | 0.1 (0.0 to 0.3) | 0.1 (0.0 to 0.3) | 0.1 (0.0 to 0.3) | 0.1 (0.0 to 0.4) | 0.1 (0.0 to 0.4) | 0.1 (0.0 to 0.4) | 0.2 (0.0 to 0.4) | 0.2 (0.0 to 0.4) |

|  | 2000 | 2001 | 2002 | 2003 | 2004 | 2005 | 2006 | 2007 | 2008 | 2009 |
| --- | --- | --- | --- | --- | --- | --- | --- | --- | --- | --- |
| **Age-standardized death rate (per 100,000)** |  |  |  |  |  |  |  |  |  |  |
| Global | 0.3 (0.1 to 0.7) | 0.3 (0.1 to 0.7) | 0.3 (0.1 to 0.7) | 0.3 (0.1 to 0.7) | 0.3 (0.1 to 0.7) | 0.3 (0.1 to 0.7) | 0.3 (0.1 to 0.7) | 0.3 (0.1 to 0.8) | 0.3 (0.1 to 0.8) | 0.3 (0.1 to 0.8) |
| High SDI | 0.4 (0.1 to 1.0) | 0.4 (0.1 to 1.0) | 0.4 (0.1 to 1.0) | 0.4 (0.1 to 1.0) | 0.4 (0.1 to 1.0) | 0.4 (0.1 to 1.0) | 0.7 (0.2 to 1.3) | 0.7 (0.2 to 1.3) | 0.7 (0.2 to 1.3) | 0.7 (0.2 to 1.4) |
| High-middle SDI | 0.3 (0.1 to 0.7) | 0.3 (0.1 to 0.7) | 0.3 (0.1 to 0.7) | 0.3 (0.1 to 0.8) | 0.3 (0.1 to 0.8) | 0.3 (0.1 to 0.8) | 0.3 (0.1 to 0.8) | 0.3 (0.1 to 0.8) | 0.3 (0.1 to 0.8) | 0.3 (0.1 to 0.8) |
| Middle SDI | 0.2 (0.0 to 0.4) | 0.2 (0.0 to 0.4) | 0.2 (0.0 to 0.5) | 0.2 (0.0 to 0.5) | 0.2 (0.0 to 0.5) | 0.2 (0.0 to 0.5) | 0.2 (0.0 to 0.5) | 0.2 (0.0 to 0.5) | 0.2 (0.0 to 0.5) | 0.2 (0.0 to 0.5) |
| Low-middle SDI | 0.2 (0.1 to 0.4) | 0.2 (0.0 to 0.4) | 0.2 (0.0 to 0.5) | 0.2 (0.0 to 0.5) | 0.2 (0.0 to 0.5) | 0.2 (0.0 to 0.5) | 0.2 (0.0 to 0.5) | 0.2 (0.0 to 0.5) | 0.2 (0.0 to 0.5) | 0.2 (0.0 to 0.5) |
| Low SDI | 0.1 (0.0 to 0.4) | 0.1 (0.0 to 0.4) | 0.2 (0.0 to 0.4) | 0.2 (0.1 to 0.4) | 0.2 (0.1 to 0.4) | 0.2 (0.1 to 0.4) | 0.2 (0.1 to 0.4) | 0.2 (0.1 to 0.5) | 0.2 (0.0 to 0.5) | 0.2 (0.0 to 0.5) |

|  | 2010 | 2011 | 2012 | 2013 | 2014 | 2015 | 2016 | 2017 | 2018 | 2019 |
| --- | --- | --- | --- | --- | --- | --- | --- | --- | --- | --- |
| **Age-standardized death rate (per 100,000)** |  |  |  |  |  |  |  |  |  |  |
| Global | 0.3 (0.1 to 0.8) | 0.3 (0.1 to 0.8) | 0.3 (0.1 to 0.8) | 0.3 (0.1 to 0.8) | 0.3 (0.1 to 0.8) | 0.3 (0.1 to 0.8) | 0.3 (0.1 to 0.8) | 0.3 (0.1 to 0.8) | 0.3 (0.1 to 0.8) | 0.4 (0.1 to 0.8) |
| High SDI | 0.5 (0.1 to 1.1) | 0.5 (0.1 to 1.1) | 0.5 (0.1 to 1.1) | 0.5 (0.1 to 1.1) | 0.5 (0.1 to 1.1) | 0.5 (0.1 to 1.1) | 0.5 (0.1 to 1.1) | 0.5 (0.1 to 1.1) | 0.5 (0.1 to 1.1) | 0.5 (0.1 to 1.1) |
| High-middle SDI | 0.3 (0.1 to 0.8) | 0.3 (0.1 to 0.8) | 0.3 (0.1 to 0.8) | 0.3 (0.1 to 0.8) | 0.3 (0.1 to 0.8) | 0.3 (0.1 to 0.8) | 0.3 (0.1 to 0.8) | 0.3 (0.1 to 0.8) | 0.3 (0.1 to 0.8) | 0.3 (0.1 to 0.8) |
| Middle SDI | 0.2 (0.0 to 0.5) | 0.2 (0.0 to 0.5) | 0.2 (0.0 to 0.6) | 0.3 (0.1 to 0.7) | 0.3 (0.1 to 0.7) | 0.3 (0.1 to 0.7) | 0.3 (0.1 to 0.7) | 0.4 (0.1 to 0.7) | 0.4 (0.1 to 0.7) | 0.4 (0.1 to 0.8) |
| Low-middle SDI | 0.2 (0.0 to 0.6) | 0.2 (0.0 to 0.6) | 0.2 (0.1 to 0.6) | 0.3 (0.1 to 0.6) | 0.3 (0.1 to 0.6) | 0.3 (0.0 to 0.6) | 0.3 (0.1 to 0.6) | 0.3 (0.1 to 0.6) | 0.3 (0.1 to 0.6) | 0.3 (0.1 to 0.7) |
| Low SDI | 0.2 (0.0 to 0.5) | 0.2 (0.0 to 0.5) | 0.2 (0.0 to 0.5) | 0.2 (0.0 to 0.6) | 0.2 (0.0 to 0.6) | 0.2 (0.0 to 0.6) | 0.3 (0.0 to 0.6) | 0.3 (0.1 to 0.6) | 0.3 (0.1 to 0.6) | 0.3 (0.1 to 0.7) |

SDI=Sociodemographic index.
