# Supplementary material for: The global burden and associated factors of ovarian cancer in 1990–2019: findings from the Global Burden of Disease Study 2019
Source: BMC Public Health. 2022 Jul 30;22:1455. doi: 10.1186/s12889-022-13861-y (PMC9339194; doi:10.1186/s12889-022-13861-y)
Supplement: Supplementary file 10 — Additional file 10: Supplementary Table 10. Incident cases for ovarian cancer in three age groups by global and SDI regions during 1990–2019. [file 12889_2022_13861_MOESM10_ESM.docx]

Supplementary Table 10. Incident cases for ovarian cancer in three age groups by global and SDI regions during 1990–2019.

|  | Incident cases | | | | |
| --- | --- | --- | --- | --- | --- |
|  | 15 – 49 years |  | 50 – 69 years |  | ≥ 70 years |
| Global |  |  |  |  |  |
| 1990 | 39872 (34418 to 48766) |  | 65290 (60965 to 73424) |  | 35083 (32165 to 37477) |
| 1993 | 44165 (39364 to 51598) |  | 68216 (64589 to 75887) |  | 38751 (35496 to 41718) |
| 1995 | 48446 (43160 to 56583) |  | 70309 (67027 to 77226) |  | 41908 (38343 to 45061) |
| 1997 | 51402 (45076 to 59812) |  | 71836 (68304 to 78836) |  | 44449 (40571 to 47444) |
| 1999 | 54161 (47359 to 62854) |  | 74282 (70671 to 81080) |  | 47113 (43024 to 50649) |
| 2001 | 56963 (50684 to 65522) |  | 78567 (74872 to 85258) |  | 49960 (45098 to 53536) |
| 2003 | 58639 (53108 to 65808) |  | 83943 (79693 to 91203) |  | 52295 (47230 to 56551) |
| 2005 | 60591 (55358 to 67234) |  | 88598 (83703 to 96109) |  | 53970 (48746 to 58355) |
| 2007 | 62625 (57338 to 68861) |  | 93236 (87811 to 100528) |  | 56674 (50947 to 61127) |
| 2009 | 66058 (60060 to 72338) |  | 98646 (93363 to 105517) |  | 60159 (54025 to 64670) |
| 2011 | 68120 (61610 to 74730) |  | 103266 (96398 to 110968) |  | 62025 (55424 to 66733) |
| 2013 | 70489 (63802 to 76529) |  | 110610 (102909 to 119159) |  | 64988 (57359 to 69895) |
| 2015 | 72646 (64777 to 79409) |  | 118723 (107736 to 128326) |  | 67137 (59050 to 72481) |
| 2017 | 75900 (67332 to 84167) |  | 126981 (113573 to 138249) |  | 71287 (62371 to 77326) |
| 2019 | 79672 (68573 to 90845) |  | 135107 (118168 to 151221) |  | 77131 (66946 to 85958) |
| High SDI |  |  |  |  |  |
| 1990 | 12955 (11660 to 13353) |  | 28008 (25054 to 28813) |  | 21290 (18851 to 22379) |
| 1993 | 13595 (12678 to 13919) |  | 27999 (25810 to 28660) |  | 23166 (20501 to 24276) |
| 1995 | 14344 (13660 to 14734) |  | 28496 (26759 to 29279) |  | 24705 (21841 to 25936) |
| 1997 | 14500 (14145 to 14842) |  | 28556 (27468 to 29192) |  | 25673 (22825 to 26945) |
| 1999 | 14406 (14053 to 14725) |  | 29152 (28262 to 29740) |  | 26605 (23556 to 27885) |
| 2001 | 14432 (14112 to 14840) |  | 29959 (29065 to 30604) |  | 27345 (24119 to 28698) |
| 2003 | 14351 (14002 to 14920) |  | 30846 (29986 to 31691) |  | 27750 (24318 to 29241) |
| 2005 | 13969 (13612 to 14622) |  | 30863 (30006 to 31637) |  | 27638 (24063 to 29185) |
| 2007 | 13839 (13431 to 14653) |  | 31360 (30423 to 32366) |  | 28285 (24648 to 29924) |
| 2009 | 14062 (13597 to 14922) |  | 32296 (31107 to 33832) |  | 29462 (25583 to 31462) |
| 2011 | 13485 (13020 to 14306) |  | 31873 (30860 to 33307) |  | 29424 (25292 to 31394) |
| 2013 | 13176 (12647 to 14128) |  | 32367 (31313 to 34410) |  | 30394 (26043 to 32544) |
| 2015 | 12719 (12056 to 13648) |  | 32335 (30845 to 34769) |  | 30491 (26176 to 32947) |
| 2017 | 12757 (11838 to 13960) |  | 33237 (30907 to 36061) |  | 31732 (26988 to 35000) |
| 2019 | 12699 (11168 to 14532) |  | 33813 (29810 to 38581) |  | 33741 (28096 to 38375) |
| High-middle SDI |  |  |  |  |  |
| 1990 | 12535 (10591 to 13779) |  | 21957 (20544 to 23428) |  | 8723 (8127 to 9465) |
| 1993 | 13914 (12114 to 14909) |  | 22998 (21939 to 24483) |  | 9763 (9115 to 10614) |
| 1995 | 15412 (13814 to 16560) |  | 23140 (22117 to 24653) |  | 10758 (10040 to 11653) |
| 1997 | 16103 (14136 to 17358) |  | 22813 (21900 to 23750) |  | 11560 (10798 to 12428) |
| 1999 | 16848 (14754 to 18225) |  | 22998 (22080 to 24090) |  | 12462 (11613 to 13400) |
| 2001 | 17511 (15506 to 18870) |  | 24266 (23227 to 25500) |  | 13557 (12575 to 14429) |
| 2003 | 17681 (16087 to 18634) |  | 25914 (24685 to 27138) |  | 14402 (13325 to 15457) |
| 2005 | 18091 (16651 to 19169) |  | 27600 (26158 to 28777) |  | 14954 (13691 to 15932) |
| 2007 | 18212 (16697 to 19222) |  | 28686 (26735 to 29954) |  | 15728 (14238 to 16605) |
| 2009 | 18714 (17119 to 19856) |  | 29794 (27862 to 31019) |  | 16677 (15153 to 17552) |
| 2011 | 18809 (17002 to 20030) |  | 30854 (28565 to 32376) |  | 17122 (15415 to 18095) |
| 2013 | 18731 (16886 to 19905) |  | 32503 (29942 to 34078) |  | 17332 (15551 to 18339) |
| 2015 | 18914 (16978 to 20409) |  | 34616 (31449 to 36697) |  | 17498 (15551 to 18708) |
| 2017 | 19163 (16938 to 21047) |  | 36047 (31863 to 38961) |  | 18180 (15877 to 19817) |
| 2019 | 19717 (16578 to 22330) |  | 37733 (32039 to 42297) |  | 19512 (16591 to 21838) |
| Middle SDI |  |  |  |  |  |
| 1990 | 9162 (7157 to 12037) |  | 8692 (7605 to 11125) |  | 2922 (2588 to 3740) |
| 1993 | 10697 (8701 to 13076) |  | 9883 (8817 to 12530) |  | 3424 (3057 to 4447) |
| 1995 | 12079 (9828 to 14685) |  | 10710 (9715 to 13341) |  | 3830 (3444 to 4871) |
| 1997 | 13404 (10893 to 16106) |  | 11725 (10676 to 14242) |  | 4309 (3876 to 5384) |
| 1999 | 14727 (11973 to 17815) |  | 12745 (11714 to 15341) |  | 4806 (4342 to 5946) |
| 2001 | 15909 (13066 to 19109) |  | 14023 (12871 to 16642) |  | 5394 (4844 to 6583) |
| 2003 | 16677 (14174 to 19359) |  | 15733 (14310 to 18492) |  | 6032 (5443 to 7225) |
| 2005 | 17570 (15480 to 20324) |  | 17488 (15972 to 20385) |  | 6721 (6013 to 7932) |
| 2007 | 18632 (16570 to 21550) |  | 19297 (17585 to 22210) |  | 7445 (6625 to 8688) |
| 2009 | 20141 (17819 to 23053) |  | 21265 (19250 to 24262) |  | 8238 (7323 to 9548) |
| 2011 | 21417 (18875 to 24777) |  | 23578 (20774 to 27217) |  | 9067 (7891 to 10465) |
| 2013 | 22532 (19861 to 25681) |  | 26420 (22981 to 30101) |  | 9967 (8566 to 11408) |
| 2015 | 23305 (20246 to 26409) |  | 29778 (25045 to 33689) |  | 10951 (9158 to 12469) |
| 2017 | 24416 (20893 to 27904) |  | 33118 (27963 to 38180) |  | 12151 (10107 to 13867) |
| 2019 | 25734 (21011 to 30453) |  | 36400 (29825 to 42800) |  | 13611 (11163 to 15822) |
| Low-middle SDI |  |  |  |  |  |
| 1990 | 3762 (2782 to 6583) |  | 4649 (3635 to 7341) |  | 1545 (1176 to 2280) |
| 1993 | 4324 (3303 to 7264) |  | 5192 (4159 to 7899) |  | 1735 (1357 to 2490) |
| 1995 | 4833 (3811 to 7985) |  | 5682 (4682 to 8408) |  | 1907 (1518 to 2673) |
| 1997 | 5438 (4275 to 8648) |  | 6297 (5177 to 9180) |  | 2137 (1735 to 2940) |
| 1999 | 6013 (4799 to 9380) |  | 6771 (5682 to 9656) |  | 2397 (1961 to 3226) |
| 2001 | 6719 (5516 to 10055) |  | 7476 (6366 to 10364) |  | 2733 (2261 to 3630) |
| 2003 | 7289 (6052 to 10355) |  | 8308 (7203 to 11055) |  | 3079 (2606 to 4003) |
| 2005 | 8035 (6747 to 10998) |  | 9197 (8002 to 11874) |  | 3506 (3003 to 4499) |
| 2007 | 8752 (7419 to 11501) |  | 10143 (8792 to 12780) |  | 3950 (3387 to 5053) |
| 2009 | 9611 (8066 to 12069) |  | 11161 (9658 to 13765) |  | 4377 (3767 to 5609) |
| 2011 | 10507 (8804 to 13045) |  | 12406 (10603 to 15260) |  | 4862 (4155 to 6162) |
| 2013 | 11622 (9765 to 14176) |  | 14147 (11952 to 17192) |  | 5542 (4773 to 7041) |
| 2015 | 12719 (10504 to 15677) |  | 16154 (13377 to 20252) |  | 6241 (5355 to 7864) |
| 2017 | 13910 (11257 to 17290) |  | 18002 (14682 to 22757) |  | 7027 (5894 to 9054) |
| 2019 | 15179 (11950 to 19185) |  | 19825 (15987 to 25130) |  | 7820 (6471 to 10044) |
| Low SDI |  |  |  |  |  |
| 1990 | 1442 (936 to 3283) |  | 1959 (1320 to 3945) |  | 589 (411 to 1002) |
| 1993 | 1615 (1091 to 3610) |  | 2118 (1468 to 4198) |  | 647 (457 to 1078) |
| 1995 | 1756 (1171 to 3761) |  | 2251 (1577 to 4364) |  | 690 (493 to 1115) |
| 1997 | 1933 (1345 to 4100) |  | 2411 (1746 to 4550) |  | 748 (544 to 1194) |
| 1999 | 2138 (1501 to 4346) |  | 2580 (1891 to 4736) |  | 820 (601 to 1261) |
| 2001 | 2361 (1706 to 4613) |  | 2803 (2105 to 5007) |  | 906 (675 to 1388) |
| 2003 | 2607 (1910 to 4922) |  | 3100 (2362 to 5341) |  | 1006 (759 to 1511) |
| 2005 | 2890 (2158 to 5187) |  | 3406 (2653 to 5625) |  | 1123 (850 to 1615) |
| 2007 | 3151 (2430 to 5303) |  | 3703 (2912 to 5784) |  | 1237 (953 to 1738) |
| 2009 | 3490 (2741 to 5686) |  | 4080 (3245 to 6139) |  | 1374 (1073 to 1879) |
| 2011 | 3860 (3060 to 5907) |  | 4502 (3603 to 6490) |  | 1518 (1210 to 2017) |
| 2013 | 4387 (3496 to 6230) |  | 5118 (4158 to 7045) |  | 1720 (1394 to 2230) |
| 2015 | 4946 (3979 to 6647) |  | 5782 (4762 to 7559) |  | 1922 (1594 to 2452) |
| 2017 | 5609 (4528 to 7065) |  | 6514 (5420 to 8141) |  | 2162 (1794 to 2750) |
| 2019 | 6296 (4998 to 7975) |  | 7269 (5962 to 8989) |  | 2408 (2005 to 3078) |

SDI=Sociodemographic index.
