# Supplementary material for: The global burden and associated factors of ovarian cancer in 1990–2019: findings from the Global Burden of Disease Study 2019
Source: BMC Public Health. 2022 Jul 30;22:1455. doi: 10.1186/s12889-022-13861-y (PMC9339194; doi:10.1186/s12889-022-13861-y)
Supplement: Supplementary file 11 — Additional file 11: Supplementary Table 11. Age-standardized incidence rate, death rate and DALY rate per 100 000 population for ovarian cancer by global and SDI regions during 1990–2019. [file 12889_2022_13861_MOESM11_ESM.docx]

Supplementary Table 11. Age-standardized incidence rate, death rate and DALY rate per 100 000 population for ovarian cancer by global and SDI regions during 1990–2019.

|  | 1990 | 1991 | 1992 | 1993 | 1994 | 1995 | 1996 | 1997 | 1998 | 1999 |
| --- | --- | --- | --- | --- | --- | --- | --- | --- | --- | --- |
| **Age-standardized incident rate (per 100,000)** |  |  |  |  |  |  |  |  |  |  |
| Global | 6.5 (6.0 to 7.3) | 6.5 (6.0 to 7.3) | 6.5 (6.1 to 7.2) | 6.5 (6.1 to 7.3) | 6.6 (6.2 to 7.2) | 6.7 (6.3 to 7.4) | 6.7 (6.2 to 7.3) | 6.7 (6.2 to 7.3) | 6.7 (6.2 to 7.3) | 6.7 (6.2 to 7.3) |
| High SDI | 11.5 (10.4 to 11.8) | 11.4 (10.4 to 11.7) | 11.4 (10.5 to 11.7) | 11.4 (10.6 to 11.7) | 11.4 (10.6 to 11.7) | 11.5 (10.8 to 11.8) | 11.5 (10.8 to 11.7) | 11.4 (10.8 to 11.7) | 11.3 (10.8 to 11.6) | 11.3 (10.7 to 11.5) |
| High-middle SDI | 7.3 (6.7 to 7.8) | 7.4 (6.8 to 7.8) | 7.4 (7.0 to 7.8) | 7.5 (7.1 to 7.9) | 7.5 (7.1 to 8.0) | 7.7 (7.3 to 8.1) | 7.6 (7.2 to 7.9) | 7.6 (7.1 to 7.9) | 7.5 (7.1 to 7.9) | 7.5 (7.1 to 7.9) |
| Middle SDI | 3.4 (3.0 to 4.3) | 3.5 (3.1 to 4.3) | 3.6 (3.2 to 4.4) | 3.6 (3.3 to 4.4) | 3.7 (3.4 to 4.6) | 3.8 (3.5 to 4.5) | 3.9 (3.6 to 4.6) | 4.0 (3.6 to 4.7) | 4.1 (3.7 to 4.8) | 4.2 (3.8 to 4.8) |
| Low-middle SDI | 3.0 (2.3 to 4.7) | 3.0 (2.4 to 4.7) | 3.0 (2.4 to 4.7) | 3.1 (2.5 to 4.7) | 3.2 (2.6 to 4.7) | 3.2 (2.7 to 4.8) | 3.3 (2.8 to 4.8) | 3.4 (2.8 to 5.0) | 3.5 (2.9 to 5.1) | 3.6 (3.0 to 5.1) |
| Low SDI | 3.0 (2.0 to 5.9) | 3.0 (2.1 to 5.8) | 3.0 (2.1 to 6.0) | 3.0 (2.1 to 5.9) | 3.1 (2.2 to 5.9) | 3.1 (2.2 to 5.9) | 3.1 (2.3 to 5.9) | 3.2 (2.3 to 5.9) | 3.3 (2.4 to 6.0) | 3.3 (2.5 to 5.9) |
| **Age-standardized death rate (per 100,000)** |  |  |  |  |  |  |  |  |  |  |
| Global | 4.6 (4.2 to 5.2) | 4.6 (4.3 to 5.1) | 4.6 (4.3 to 5.1) | 4.6 (4.3 to 5.1) | 4.6 (4.3 to 5.1) | 4.7 (4.4 to 5.1) | 4.6 (4.4 to 5.1) | 4.6 (4.4 to 5.1) | 4.6 (4.3 to 5.1) | 4.6 (4.3 to 5.0) |
| High SDI | 7.5 (6.7 to 7.7) | 7.4 (6.7 to 7.6) | 7.3 (6.7 to 7.5) | 7.3 (6.7 to 7.5) | 7.3 (6.7 to 7.5) | 7.3 (6.8 to 7.5) | 7.3 (6.7 to 7.5) | 7.2 (6.7 to 7.4) | 7.1 (6.7 to 7.3) | 7.1 (6.6 to 7.3) |
| High-middle SDI | 5.0 (4.6 to 5.3) | 5.0 (4.7 to 5.3) | 5.0 (4.7 to 5.3) | 5.1 (4.8 to 5.4) | 5.1 (4.8 to 5.4) | 5.2 (4.9 to 5.5) | 5.1 (4.9 to 5.4) | 5.1 (4.8 to 5.3) | 5.1 (4.8 to 5.3) | 5.0 (4.8 to 5.3) |
| Middle SDI | 2.4 (2.2 to 3.0) | 2.5 (2.2 to 3.1) | 2.5 (2.3 to 3.1) | 2.6 (2.3 to 3.2) | 2.6 (2.4 to 3.2) | 2.7 (2.4 to 3.2) | 2.7 (2.5 to 3.3) | 2.8 (2.5 to 3.3) | 2.8 (2.6 to 3.4) | 2.8 (2.6 to 3.4) |
| Low-middle SDI | 2.3 (1.8 to 3.6) | 2.4 (1.9 to 3.6) | 2.4 (1.9 to 3.6) | 2.4 (1.9 to 3.6) | 2.5 (2.0 to 3.6) | 2.5 (2.1 to 3.6) | 2.6 (2.1 to 3.6) | 2.6 (2.2 to 3.8) | 2.7 (2.2 to 3.8) | 2.7 (2.3 to 3.8) |
| Low SDI | 2.4 (1.7 to 4.7) | 2.5 (1.7 to 4.7) | 2.5 (1.8 to 4.7) | 2.5 (1.8 to 4.7) | 2.5 (1.8 to 4.7) | 2.5 (1.8 to 4.7) | 2.6 (1.9 to 4.7) | 2.6 (1.9 to 4.7) | 2.7 (2.0 to 4.7) | 2.7 (2.0 to 4.7) |
| **Age-standardized DALY rate (per 100,000)** |  |  |  |  |  |  |  |  |  |  |
| Global | 124.1 (113.7 to 143.0) | 123.9 (113.6 to 142.1) | 123.9 (114.6 to 141.7) | 124.3 (115.8 to 141.5) | 124.5 (116.8 to 141.1) | 125.8 (118.0 to 141.5) | 125.1 (116.8 to 140.9) | 124.7 (116.1 to 140.1) | 124.2 (115.7 to 139.4) | 123.9 (115.9 to 138.4) |
| High SDI | 198.3 (178.8 to 204.3) | 196.2 (178.5 to 201.4) | 194.3 (178.5 to 199.1) | 192.5 (178.2 to 197.3) | 191.3 (178.5 to 196.4) | 192.6 (181.3 to 198.0) | 190.3 (180.3 to 195.2) | 188.2 (178.6 to 192.9) | 185.8 (177.8 to 190.3) | 183.7 (176.0 to 188.0) |
| High-middle SDI | 145.1 (132.9 to 155.3) | 145.5 (133.6 to 155.2) | 146.3 (136.1 to 155.1) | 147.9 (140.2 to 156.2) | 148.5 (141.0 to 157.8) | 150.3 (142.9 to 160.3) | 148.1 (140.8 to 155.7) | 146.4 (137.3 to 153.4) | 144.9 (135.3 to 151.7) | 144.4 (136.2 to 151.5) |
| Middle SDI | 73.9 (64.7 to 93.5) | 75.1 (66.3 to 92.8) | 76.3 (67.8 to 93.9) | 77.4 (69.3 to 94.0) | 78.9 (71.1 to 95.0) | 80.2 (72.7 to 95.8) | 81.6 (73.5 to 97.5) | 82.8 (74.8 to 97.8) | 84.1 (76.5 to 99.7) | 84.9 (77.3 to 99.3) |
| Low-middle SDI | 67.6 (53.3 to 107.7) | 68.2 (54.0 to 108.0) | 69.0 (54.8 to 108.2) | 70.0 (56.5 to 108.5) | 71.5 (57.4 to 108.8) | 72.9 (59.8 to 108.5) | 74.5 (61.5 to 110.0) | 76.5 (62.9 to 113.7) | 77.7 (64.6 to 115.1) | 79.0 (66.3 to 113.5) |
| Low SDI | 71.0 (48.9 to 144.9) | 71.6 (49.2 to 146.0) | 72.1 (50.3 to 146.0) | 72.7 (50.3 to 145.4) | 73.4 (51.9 to 143.8) | 74.1 (52.7 to 143.6) | 74.9 (54.2 to 143.5) | 75.9 (55.4 to 144.6) | 77.1 (56.9 to 144.5) | 78.1 (57.5 to 144.3) |

|  | 2000 | 2001 | 2002 | 2003 | 2004 | 2005 | 2006 | 2007 | 2008 | 2009 |
| --- | --- | --- | --- | --- | --- | --- | --- | --- | --- | --- |
| **Age-standardized incident rate (per 100,000)** |  |  |  |  |  |  |  |  |  |  |
| Global | 6.7 (6.3 to 7.3) | 6.7 (6.3 to 7.3) | 6.7 (6.3 to 7.3) | 6.7 (6.4 to 7.3) | 6.7 (6.3 to 7.3) | 6.7 (6.3 to 7.2) | 6.7 (6.2 to 7.2) | 6.7 (6.2 to 7.2) | 6.7 (6.3 to 7.2) | 6.7 (6.3 to 7.2) |
| High SDI | 11.2 (10.7 to 11.5) | 11.2 (10.7 to 11.4) | 11.1 (10.6 to 11.4) | 11.0 (10.5 to 11.3) | 10.8 (10.4 to 11.2) | 10.5 (10.1 to 10.9) | 10.4 (9.9 to 10.7) | 10.3 (9.8 to 10.7) | 10.3 (9.8 to 10.8) | 10.3 (9.8 to 10.8) |
| High-middle SDI | 7.7 (7.2 to 8.0) | 7.7 (7.2 to 8.0) | 7.7 (7.3 to 8.1) | 7.7 (7.3 to 8.1) | 7.7 (7.3 to 8.1) | 7.8 (7.3 to 8.2) | 7.7 (7.2 to 8.1) | 7.7 (7.2 to 8.1) | 7.7 (7.2 to 8.1) | 7.7 (7.2 to 8.0) |
| Middle SDI | 4.2 (3.8 to 4.9) | 4.3 (3.9 to 5.0) | 4.4 (4.0 to 5.1) | 4.4 (4.0 to 5.1) | 4.5 (4.1 to 5.2) | 4.6 (4.1 to 5.2) | 4.6 (4.2 to 5.3) | 4.7 (4.3 to 5.3) | 4.8 (4.3 to 5.5) | 4.9 (4.4 to 5.5) |
| Low-middle SDI | 3.7 (3.1 to 5.2) | 3.8 (3.2 to 5.3) | 3.9 (3.3 to 5.3) | 3.9 (3.4 to 5.2) | 4.0 (3.5 to 5.2) | 4.1 (3.6 to 5.3) | 4.2 (3.6 to 5.3) | 4.3 (3.7 to 5.4) | 4.4 (3.8 to 5.4) | 4.4 (3.9 to 5.4) |
| Low SDI | 3.4 (2.6 to 6.0) | 3.4 (2.6 to 6.1) | 3.5 (2.7 to 6.0) | 3.6 (2.8 to 6.1) | 3.7 (2.8 to 6.1) | 3.8 (2.9 to 6.1) | 3.8 (3.0 to 6.1) | 3.9 (3.1 to 6.1) | 3.9 (3.1 to 6.0) | 4.0 (3.2 to 6.0) |
| **Age-standardized death rate (per 100,000)** |  |  |  |  |  |  |  |  |  |  |
| Global | 4.6 (4.4 to 5.0) | 4.6 (4.3 to 5.0) | 4.6 (4.4 to 5.0) | 4.6 (4.3 to 5.0) | 4.6 (4.3 to 5.0) | 4.6 (4.3 to 4.9) | 4.6 (4.3 to 4.9) | 4.6 (4.2 to 4.9) | 4.6 (4.2 to 4.9) | 4.6 (4.2 to 4.9) |
| High SDI | 7.0 (6.6 to 7.2) | 7.0 (6.5 to 7.2) | 6.9 (6.5 to 7.1) | 6.8 (6.4 to 7.0) | 6.7 (6.3 to 6.9) | 6.5 (6.1 to 6.7) | 6.4 (6.0 to 6.6) | 6.4 (5.9 to 6.6) | 6.3 (5.9 to 6.6) | 6.3 (5.8 to 6.6) |
| High-middle SDI | 5.1 (4.9 to 5.3) | 5.1 (4.9 to 5.4) | 5.1 (4.9 to 5.4) | 5.1 (4.9 to 5.4) | 5.1 (4.9 to 5.4) | 5.2 (4.9 to 5.4) | 5.1 (4.8 to 5.3) | 5.1 (4.7 to 5.3) | 5.1 (4.7 to 5.3) | 5.0 (4.7 to 5.3) |
| Middle SDI | 2.9 (2.6 to 3.4) | 2.9 (2.7 to 3.4) | 3.0 (2.7 to 3.5) | 3.0 (2.7 to 3.5) | 3.0 (2.8 to 3.5) | 3.1 (2.8 to 3.5) | 3.1 (2.8 to 3.6) | 3.1 (2.9 to 3.6) | 3.2 (2.9 to 3.6) | 3.2 (2.9 to 3.7) |
| Low-middle SDI | 2.8 (2.4 to 3.8) | 2.9 (2.4 to 3.9) | 2.9 (2.5 to 4.0) | 3.0 (2.6 to 3.9) | 3.0 (2.6 to 3.9) | 3.1 (2.7 to 3.9) | 3.2 (2.8 to 4.0) | 3.2 (2.8 to 4.0) | 3.3 (2.8 to 4.0) | 3.3 (2.9 to 4.0) |
| Low SDI | 2.7 (2.0 to 4.7) | 2.8 (2.1 to 4.7) | 2.9 (2.2 to 4.8) | 2.9 (2.2 to 4.8) | 3.0 (2.3 to 4.8) | 3.0 (2.3 to 4.8) | 3.1 (2.4 to 4.8) | 3.1 (2.4 to 4.7) | 3.2 (2.5 to 4.8) | 3.2 (2.5 to 4.7) |
| **Age-standardized DALY rate (per 100,000)** |  |  |  |  |  |  |  |  |  |  |
| Global | 124.6 (116.6 to 138.4) | 124.6 (116.7 to 138.5) | 124.8 (117.1 to 138.1) | 124.3 (116.6 to 136.8) | 124.0 (116.0 to 136.2) | 123.5 (115.8 to 134.9) | 122.8 (115.0 to 134.3) | 122.4 (114.4 to 133.2) | 122.4 (114.4 to 133.1) | 122.4 (114.1 to 132.6) |
| High SDI | 182.1 (174.9 to 186.6) | 180.5 (173.2 to 184.9) | 178.5 (171.5 to 183.0) | 175.7 (169.2 to 180.9) | 173.4 (166.5 to 178.7) | 167.9 (161.0 to 172.7) | 165.6 (158.5 to 169.9) | 163.6 (156.3 to 168.5) | 162.1 (154.7 to 168.5) | 161.1 (153.5 to 168.7) |
| High-middle SDI | 146.4 (137.3 to 152.9) | 146.0 (137.5 to 153.0) | 146.2 (137.6 to 153.2) | 145.5 (137.4 to 152.7) | 144.8 (136.3 to 152.0) | 145.5 (136.8 to 152.6) | 143.5 (133.8 to 150.0) | 142.4 (132.2 to 148.2) | 141.7 (132.3 to 148.1) | 140.5 (130.6 to 146.6) |
| Middle SDI | 86.1 (78.3 to 100.8) | 87.0 (79.2 to 101.3) | 88.3 (80.0 to 103.0) | 89.0 (80.4 to 103.3) | 89.8 (81.1 to 103.2) | 90.7 (82.5 to 104.4) | 91.4 (83.1 to 105.5) | 92.2 (83.8 to 106.3) | 93.3 (84.4 to 107.0) | 94.4 (85.4 to 107.5) |
| Low-middle SDI | 81.0 (68.1 to 115.7) | 82.8 (70.2 to 116.0) | 84.9 (72.7 to 117.7) | 86.2 (73.6 to 116.6) | 87.5 (75.5 to 114.7) | 89.9 (77.7 to 115.6) | 91.5 (79.3 to 116.5) | 92.9 (80.9 to 117.6) | 94.4 (82.1 to 116.8) | 95.9 (83.0 to 116.9) |
| Low SDI | 79.3 (58.2 to 144.3) | 80.8 (60.4 to 144.9) | 82.4 (61.3 to 145.1) | 84.1 (64.7 to 146.1) | 85.7 (66.5 to 145.7) | 87.3 (67.0 to 145.0) | 88.5 (68.4 to 143.4) | 89.5 (70.7 to 141.4) | 90.8 (71.4 to 142.6) | 92.5 (73.5 to 140.9) |

|  | 2010 | 2011 | 2012 | 2013 | 2014 | 2015 | 2016 | 2017 | 2018 | 2019 |
| --- | --- | --- | --- | --- | --- | --- | --- | --- | --- | --- |
| **Age-standardized incident rate (per 100,000)** |  |  |  |  |  |  |  |  |  |  |
| Global | 6.6 (6.2 to 7.1) | 6.6 (6.2 to 7.1) | 6.6 (6.1 to 7.2) | 6.7 (6.2 to 7.1) | 6.7 (6.1 to 7.2) | 6.7 (6.1 to 7.2) | 6.7 (6.1 to 7.2) | 6.7 (6.0 to 7.3) | 6.8 (6.1 to 7.5) | 6.9 (6.1 to 7.7) |
| High SDI | 9.9 (9.4 to 10.3) | 9.8 (9.3 to 10.2) | 9.7 (9.2 to 10.1) | 9.7 (9.2 to 10.1) | 9.6 (9.1 to 10.1) | 9.3 (8.7 to 9.8) | 9.3 (8.7 to 9.8) | 9.3 (8.6 to 10.0) | 9.3 (8.3 to 10.4) | 9.3 (8.2 to 10.6) |
| High-middle SDI | 7.7 (7.1 to 8.1) | 7.6 (7.0 to 8.0) | 7.5 (6.9 to 7.9) | 7.5 (6.9 to 7.9) | 7.5 (6.8 to 7.9) | 7.5 (6.8 to 7.9) | 7.4 (6.7 to 7.9) | 7.5 (6.6 to 8.1) | 7.5 (6.6 to 8.3) | 7.6 (6.4 to 8.5) |
| Middle SDI | 4.9 (4.4 to 5.7) | 5.0 (4.4 to 5.8) | 5.1 (4.5 to 5.8) | 5.2 (4.5 to 5.9) | 5.3 (4.6 to 6.0) | 5.3 (4.5 to 6.0) | 5.4 (4.6 to 6.0) | 5.5 (4.7 to 6.3) | 5.6 (4.6 to 6.5) | 5.7 (4.7 to 6.6) |
| Low-middle SDI | 4.5 (3.9 to 5.5) | 4.6 (4.0 to 5.6) | 4.7 (4.1 to 5.7) | 4.9 (4.2 to 5.9) | 5.0 (4.3 to 6.0) | 5.2 (4.4 to 6.4) | 5.3 (4.4 to 6.5) | 5.4 (4.5 to 6.8) | 5.6 (4.5 to 6.9) | 5.6 (4.6 to 7.1) |
| Low SDI | 4.1 (3.3 to 5.9) | 4.2 (3.4 to 5.9) | 4.3 (3.5 to 6.0) | 4.4 (3.6 to 6.0) | 4.6 (3.8 to 6.0) | 4.7 (3.9 to 6.1) | 4.8 (4.0 to 6.1) | 4.9 (4.1 to 6.1) | 5.1 (4.2 to 6.3) | 5.1 (4.3 to 6.3) |
| **Age-standardized death rate (per 100,000)** |  |  |  |  |  |  |  |  |  |  |
| Global | 4.5 (4.2 to 4.8) | 4.5 (4.1 to 4.8) | 4.5 (4.1 to 4.8) | 4.5 (4.1 to 4.8) | 4.5 (4.1 to 4.9) | 4.5 (4.1 to 4.8) | 4.5 (4.0 to 4.8) | 4.5 (4.0 to 4.9) | 4.5 (4.0 to 5.0) | 4.6 (4.0 to 5.0) |
| High SDI | 6.0 (5.6 to 6.3) | 6.0 (5.6 to 6.2) | 5.9 (5.5 to 6.2) | 5.9 (5.5 to 6.1) | 5.8 (5.4 to 6.1) | 5.7 (5.2 to 6.0) | 5.7 (5.2 to 6.0) | 5.6 (5.2 to 6.0) | 5.6 (5.1 to 6.0) | 5.7 (5.2 to 6.1) |
| High-middle SDI | 5.0 (4.6 to 5.2) | 4.9 (4.5 to 5.2) | 4.9 (4.5 to 5.1) | 4.8 (4.4 to 5.1) | 4.8 (4.4 to 5.1) | 4.8 (4.3 to 5.1) | 4.7 (4.2 to 5.0) | 4.7 (4.2 to 5.1) | 4.7 (4.2 to 5.1) | 4.7 (4.1 to 5.2) |
| Middle SDI | 3.3 (2.9 to 3.7) | 3.3 (2.9 to 3.8) | 3.3 (2.9 to 3.8) | 3.4 (3.0 to 3.8) | 3.5 (3.0 to 3.9) | 3.5 (3.0 to 4.0) | 3.5 (2.9 to 4.0) | 3.6 (3.0 to 4.1) | 3.6 (3.0 to 4.2) | 3.7 (3.0 to 4.3) |
| Low-middle SDI | 3.4 (2.9 to 4.1) | 3.4 (3.0 to 4.1) | 3.5 (3.0 to 4.2) | 3.6 (3.1 to 4.3) | 3.7 (3.2 to 4.5) | 3.8 (3.2 to 4.6) | 3.9 (3.3 to 4.8) | 4.0 (3.3 to 4.9) | 4.0 (3.3 to 5.0) | 4.1 (3.4 to 5.1) |
| Low SDI | 3.3 (2.6 to 4.7) | 3.3 (2.7 to 4.7) | 3.4 (2.8 to 4.7) | 3.5 (2.8 to 4.7) | 3.6 (3.0 to 4.7) | 3.7 (3.1 to 4.8) | 3.8 (3.2 to 4.8) | 3.9 (3.3 to 4.8) | 4.0 (3.3 to 4.9) | 4.0 (3.4 to 4.9) |
| **Age-standardized DALY rate (per 100,000)** |  |  |  |  |  |  |  |  |  |  |
| Global | 121.2 (112.1 to 130.9) | 120.8 (112.1 to 131.0) | 121.0 (111.4 to 130.8) | 121.2 (111.3 to 130.5) | 122.1 (111.3 to 131.6) | 121.5 (109.7 to 132.1) | 121.9 (110.4 to 132.4) | 122.9 (110.4 to 134.8) | 124.0 (110.0 to 136.6) | 124.7 (109.1 to 138.7) |
| High SDI | 154.6 (147.5 to 160.8) | 152.9 (146.1 to 158.4) | 151.4 (144.6 to 157.2) | 149.7 (143.0 to 156.0) | 148.4 (140.9 to 156.0) | 143.8 (135.5 to 151.7) | 143.6 (135.2 to 151.4) | 143.5 (133.7 to 152.4) | 143.5 (133.1 to 153.6) | 143.8 (132.6 to 154.5) |
| High-middle SDI | 139.4 (128.5 to 146.1) | 137.3 (126.8 to 144.2) | 135.8 (125.0 to 143.0) | 134.6 (123.2 to 141.7) | 134.4 (122.7 to 141.8) | 133.2 (120.9 to 141.2) | 132.0 (118.4 to 140.1) | 132.0 (117.0 to 142.2) | 132.7 (115.8 to 144.8) | 133.0 (114.8 to 147.5) |
| Middle SDI | 95.5 (84.5 to 108.7) | 96.4 (86.3 to 109.5) | 97.6 (85.9 to 110.6) | 98.9 (85.9 to 111.9) | 100.7 (87.1 to 114.3) | 101.4 (86.1 to 115.2) | 102.1 (85.8 to 115.7) | 103.9 (87.8 to 118.7) | 105.5 (88.1 to 121.0) | 106.4 (87.7 to 124.0) |
| Low-middle SDI | 97.4 (84.6 to 117.4) | 99.1 (85.2 to 120.4) | 101.9 (87.4 to 123.9) | 104.7 (89.0 to 126.8) | 107.5 (90.3 to 130.3) | 110.3 (92.2 to 135.0) | 112.7 (94.2 to 139.7) | 114.9 (94.8 to 143.2) | 117.0 (95.5 to 146.9) | 118.4 (95.4 to 150.2) |
| Low SDI | 93.8 (75.4 to 138.5) | 95.3 (77.3 to 138.3) | 98.1 (79.3 to 139.7) | 100.9 (81.3 to 138.5) | 103.7 (85.6 to 138.1) | 106.0 (88.0 to 139.6) | 108.7 (90.5 to 137.7) | 111.2 (92.8 to 140.5) | 113.8 (95.3 to 142.0) | 115.2 (96.4 to 141.8) |

SDI=Sociodemographic index.
