# Supplementary material for: The global burden and associated factors of ovarian cancer in 1990–2019: findings from the Global Burden of Disease Study 2019
Source: BMC Public Health. 2022 Jul 30;22:1455. doi: 10.1186/s12889-022-13861-y (PMC9339194; doi:10.1186/s12889-022-13861-y)
Supplement: Supplementary file 12 — Additional file 12: Supplementary Figure 1. The correlation of ovarian cancer incident cases and SDI (a), the correlation of ovarian cancer DALYs and SDI (b), 1990-2019. [file 12889_2022_13861_MOESM12_ESM.docx]

Supplementary Figure 1. The correlation of ovarian cancer incident cases and SDI (a), the correlation of ovarian cancer DALYs and SDI (b), 1990-2019.


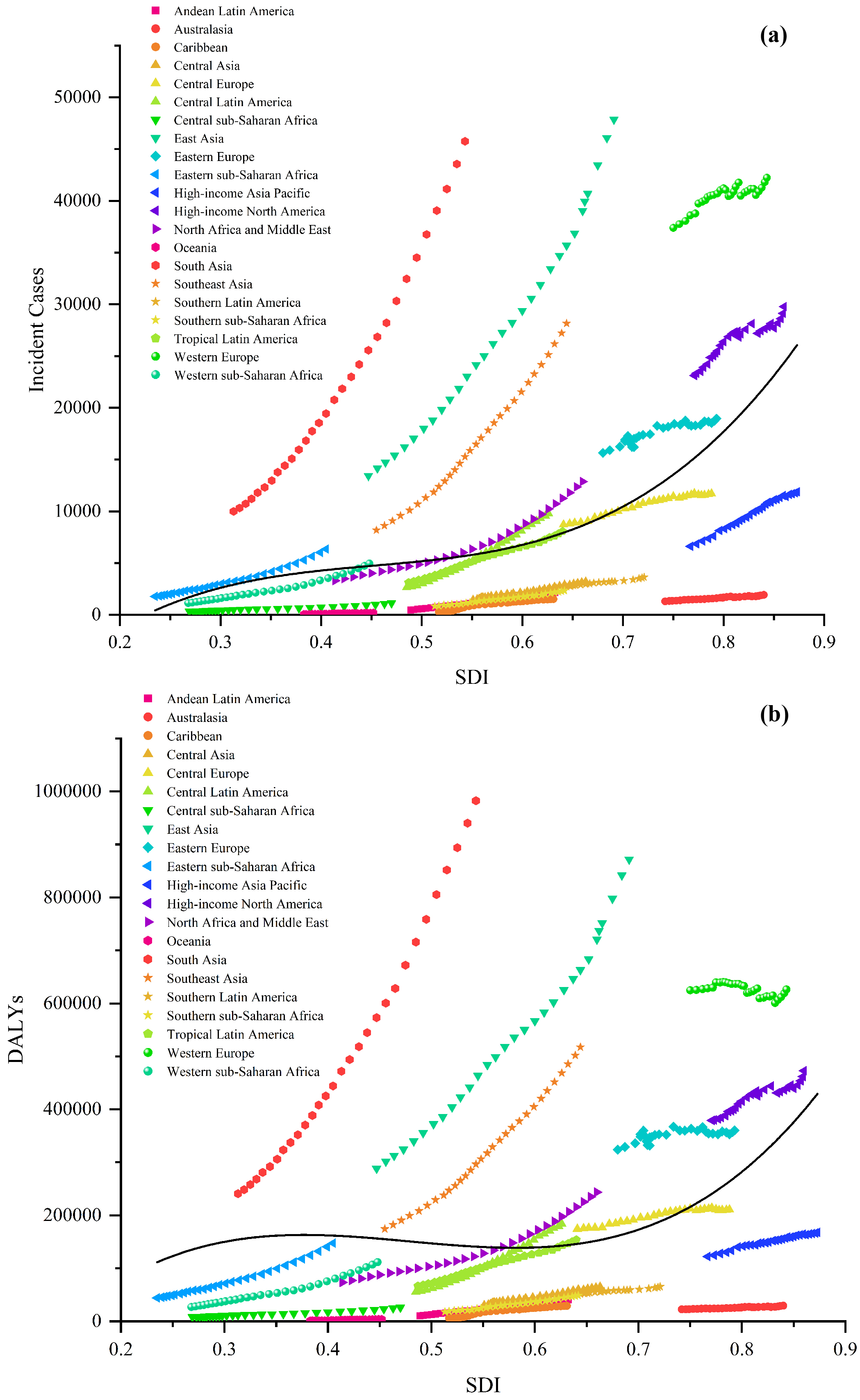


*The black line represents the average expected relationship between SDIs and cases or DALYs for ovarian cancer based on values from all countries from 1990 to 2019. SDI, social-demographic index.
