# Supplementary figures and images for: The global burden and associated factors of ovarian cancer in 1990–2019: findings from the Global Burden of Disease Study 2019
Source: BMC Public Health. 2022 Jul 30;22:1455. doi: 10.1186/s12889-022-13861-y (PMC9339194; doi:10.1186/s12889-022-13861-y)

Supplementary Figure 2. Map of percentage change of deaths (a) and DALYs (b) due to ovarian cancer, 1990-2019.


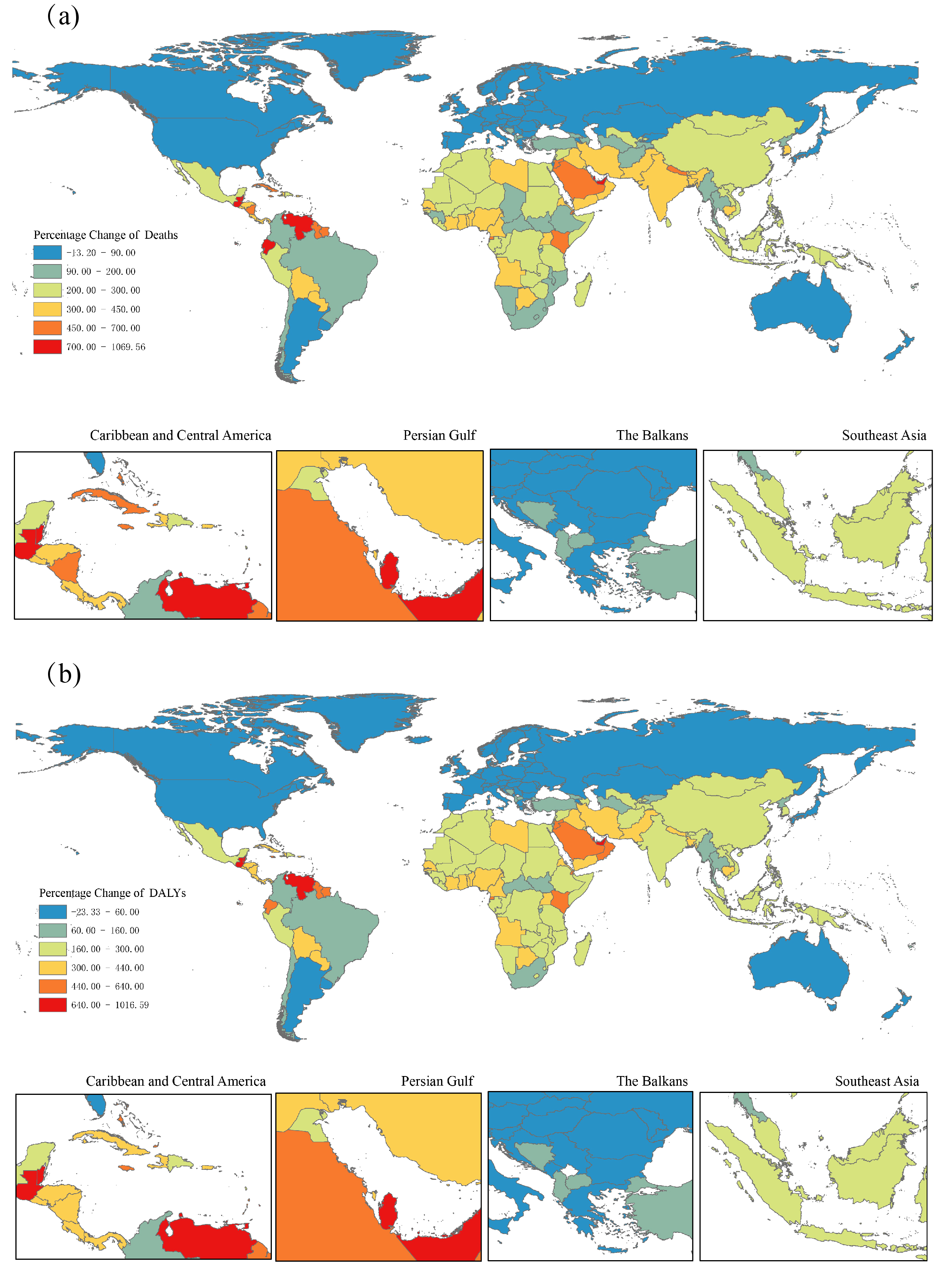

Supplement: Supplementary file 13 — Additional file 13: Supplementary Figure 2. Map of percentage change of deaths (a) and DALYs (b) due to ovarian cancer, 1990-2019. [file 12889_2022_13861_MOESM13_ESM.docx]
