# Supplementary material for: The global burden and associated factors of ovarian cancer in 1990–2019: findings from the Global Burden of Disease Study 2019
Source: BMC Public Health. 2022 Jul 30;22:1455. doi: 10.1186/s12889-022-13861-y (PMC9339194; doi:10.1186/s12889-022-13861-y)

Supplementary Figure 3. Map of age-standardized mortality (a) and DALY (b) rate due to ovarian cancer in 2019.


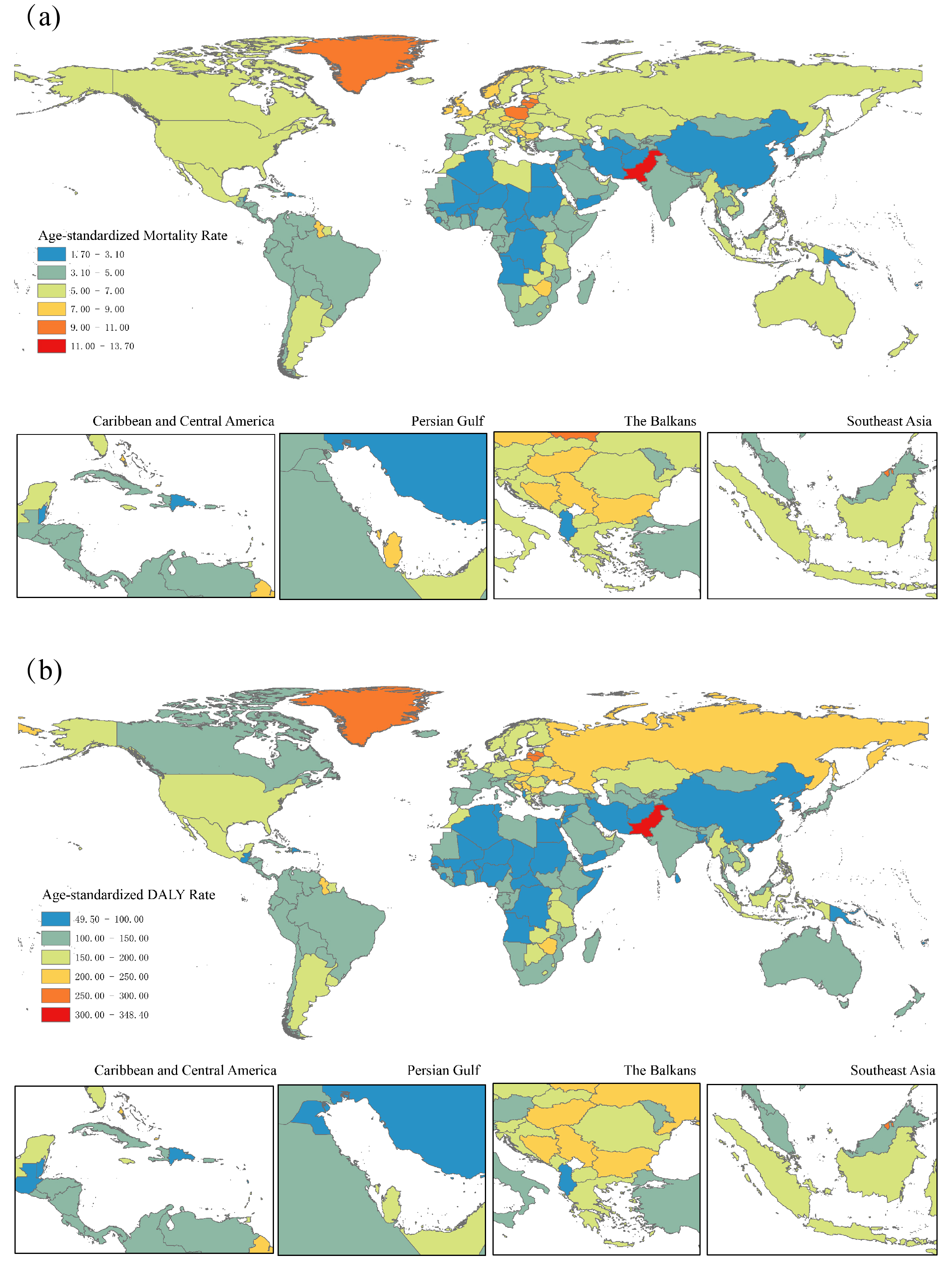

Supplement: Supplementary file 14 — Additional file 14: Supplementary Figure 3. Map of age-standardized mortality (a) and DALY (b) rate due to ovarian cancer in 2019. [file 12889_2022_13861_MOESM14_ESM.docx]
